# Supplementary material for: Rational Design 2-Hydroxypropylphosphonium Salts as Cancer Cell Mitochondria-Targeted Vectors: Synthesis, Structure, and Biological Properties
Source: Molecules. 2021 Oct 20;26(21):6350. doi: 10.3390/molecules26216350 (PMC8588467; doi:10.3390/molecules26216350)
Supplement: Supplementary file 1 [file molecules-26-06350-s001.zip › molecules-1415322-supplementary/Supporting Information-highlighted/Supporting Information-highlighted.pdf]

Supporting Information

Molecules. 2021

Crystallographic and spectral data

Rational Design 2-hydroxypropylphosphonium Salts as Cancer Cell Mitochondria-targeted Vectors. Synthesis, Structure and Biological Properties

Vladimir F. Mironov[a,], Andrey V. Nemtarev[a], Olga V. Tsepaeva[a], Mudaris N. Dimukhametov[a], Igor A. Litvinov[a], Alexandra D. Voloshina[a], Tatyana N. Pashirova[a], Eugenii A. Titov[b], Anna P. Lyubina[a], Syumbelya K. Amerhanova[a], Aidar T. Gubaidullin[a], Daut R. Islamov[a]

[a]Arbuzov Institute of Organic and Physical Chemistry, FRC Kazan Scientific Center, Russian Academy of Sciences, 8 Arbuzov St., 420088 Kazan, Russia, E-mail: mironov@iopc.ru

[b]Alexander Butlerov Institute of Chemistry, Kazan (Volga Region) Federal University, 18 Kremlevskaya St., 420008 Kazan, Russia

Table of contents

Table of contents	S1
Investigated compounds numbers	S7
General remarks for XRD investigation	S7
Table 1. Crystal Data and Refinement Details for compounds (**3b**, **3c**, **3d**, **3e** (***S***)).	S8
Table 2. Crystal Data and Refinement Details for compounds (**3a**), (**3e**, ***S***), (**3e**, ***R***)).	S9
Table 3.Crystal Data and Refinement Details for compounds (**3f**, **3g**, **3i**, **3j**).	S10
Table 4. Crystal Data and Refinement Details for compounds (**4a**, **7**).	S12
Figure. 1. Molecular geometry of independent part of crystal (**3b**).	S13
Figure 2. Molecular geometry (**3c**) in crystal.	S13
Figure 3. Molecular geometry (**3d**) in crystal.	S13
Figure 4. Molecular geometry (**3f**) in crystal.	S13
Figure 5. Molecular geometry (**3g**) in crystal. The cell contains two independent molecules, but to simplify only one molecule is shown.	S13
Figure 6. Molecular geometry (**3i**) in crystal.	S13
Figure 7. Molecular geometry (**3j**) in crystal.	S14
Figure 8. Molecular geometry (**4a**) in crystal.	S14
Figure 9 Molecular geometry (**7**) in crystal.	S14

Conclusions on the spatial structure of the studied compounds	S14
Figure 10. ^{1}H NMR spectrum (400.0 MHz, $CDCl_3$) of 1-chloro-3-methoxypropan-2-ol (**5a**).	S15
Figure 11. ^{1}H NMR spectrum (400.0 MHz, $CDCl_3$) of 1-iodo-3-methoxypropan-2-ol (**6a**).	S16
Figure 12. ^{1}H NMR spectrum (400.0 MHz, $CDCl_3$) of 1-chloro-3-ethoxypropan-2-ol (**5b**).	S17
Figure 13. ^{1}H NMR spectrum (400.0 MHz, $CDCl_3$) of 1-ethoxy-3-iodopropan-2-ol (**6b**).	S18
Figure 14. ^{1}H NMR spectrum (400.0 MHz, $CDCl_3$) of 1-(allyloxy)-3-iodopropan-2-ol (**6c**).	S19
Figure 15. ^{1}H NMR spectrum (400.0 MHz, $CDCl_3$) of 1-chloro-3-(decyloxy)propan-2-ol (**5d**).	S20
Figure 16. Fragment of ^{1}H NMR spectrum (400.0 MHz, $CDCl_3$) of 1-chloro-3-(decyloxy)propan-2-ol (**5d**).	S21
Figure 17. ^{1}H NMR spectrum (400.0 MHz, $CDCl_3$) of 1-(decyloxy)-3-iodopropan-2-ol (**6d**).	S22
Figure 18. ^{1}H NMR spectrum (400.0 MHz, $CDCl_3$) of 2-(methoxymethyl)oxirane (**2f**).	S23
Figure 19. ^{1}H NMR spectrum (400.0 MHz, $CDCl_3$) of 2-(ethoxymethyl)oxirane (**2g**).	S24
Figure 20. ^{1}H NMR spectrum (400.0 MHz, $CDCl_3$) of 2-((2,2,3,3-tetrafluoropropoxy)methyl)oxirane (**2h**).	S25
Figure 21. ^{1}H NMR spectrum (400.0 MHz, $CDCl_3$) of 2-((allyloxy)methyl)oxirane (**2i**).	S26
Figure 22. ^{1}H NMR spectrum (400.0 MHz, $CDCl_3$) of 2-((decyloxy)methyl)oxirane (**2j**).	S27
Figure 23. Fragments of ^{1}H NMR spectrum (400.0 MHz, $CDCl_3$) of 2-((decyloxy)methyl)oxirane (**2j**).	S28
Figure 24. ^{13}C-{^{1}H} NMR spectrum (100.6 MHz, $CDCl_3$) of 2-((decyloxy)methyl)oxirane (**2j**).	S29
Figure 25. ^{13}C-{^{1}H} and ^{13}C-{^{1}H}-dept NMR spectra (100.6 MHz, $CDCl_3$) 2-((decyloxy)methyl)oxirane (**2j**).	S30
Figure 26. ^{1}H NMR spectrum (400 MHz, $CDCl_3$) of oxiran-2-ylmethyl stearate (**2l**).	S31
Figure 27. Fragments of ^{1}H NMR spectrum (100.6 MHz, $CDCl_3$) of oxiran-2-ylmethyl stearate (**2l**).	S32
Figure 28. ^{13}C-{^{1}H} NMR spectrum (100.6 MHz, $CDCl_3$) of oxiran-2-ylmethyl stearate (**2l**).	S33
Figure 29. ^{13}C-{^{1}H} and ^{13}C-{^{1}H}-APT NMR spectra (100.6 MHz, $CDCl_3$) of oxiran-2-ylmethyl stearate (**2l**).	S34
Figure 30. ^{31}P-{^{1}H} NMR spectrum (162.0 MHz, CH_2Cl_2) of compound (**3a**).	S35
Figure 31. ^{1}H NMR spectrum (400 MHz, $CDCl_3$) of compound (**3e**) (*R*).	S36
Figure 32. ^{31}P-{^{1}H} NMR spectrum (162.0 MHz, $CDCl_3$) of compound (**3e**) (*R*).	S37
Figure 33. ^{1}H NMR spectrum (400 MHz, $CDCl_3$) of compound (**3e**) (*S*).	S38
Figure 34. ^{31}P-{^{1}H} NMR spectrum (162.0 MHz, $CDCl_3$) of compound (**3e**) (*S*).	S39
Figure 35. ^{13}C-{^{1}H} NMR spectrum (100.6 MHz, acetone-d_6 + 10% $CDCl_3$) of compound (**3a**).	S40
Figure 36. ^{13}C NMR spectrum (100.6 MHz, acetone-d_6 + 10% $CDCl_3$) of compound (**3a**).	S41
Figure 37. Aromatic carbons region of ^{13}C NMR spectrum (100.6 MHz, acetone-d_6 + 10% $CDCl_3$) of compound (**3a**).	S42
Figure 38. ^{13}C-{^{1}H} and ^{13}C NMR spectra (100.6 MHz, acetone-d_6 + 10% $CDCl_3$) of compound (**3a**).	S43
Figure 39. Aromatic carbon region of ^{13}C-{^{1}H} and ^{13}C NMR spectra (100.6 MHz, acetone-d_6 + 10% $CDCl_3$) of compound (**3a**).	S44
Figure 40. The 66-68 and 48-52 ppm fragments of ^{13}C-{^{1}H} and ^{13}C NMR spectra (100.6 MHz, acetone-d_6 + 10% $CDCl_3$) of compound (**3a**).	S45
Figure 41. ^{1}H NMR spectrum (500 MHz, $CDCl_3$ + 10% acetone-d_6) of compound (**3b**).	S46

Figure 42. ^{31}P-{^{1}H} NMR spectrum (162.0 MHz, $CDCl_3$) of compound (**3b**).	S47
Figure 43. ^{13}C-{^{1}H} NMR spectrum (125.76 MHz, CD_3CN) of compound (**3b**).	S48
Figure 44. ^{13}C-{^{1}H} and ^{13}C-{^{1}H}-dept NMR spectra (125.76 MHz, CD_3CN) of compound (**3b**).	S49
Figure 45. ^{13}C-{^{1}H} NMR spectrum (100.6 MHz, $CDCl_3$ + 10% acetone-d_6) of compound (**3b**).	S50
Figure 46. ^{13}C NMR spectrum (100.6 MHz, $CDCl_3$ + 10% acetone-d_6) of compound (**3b**).	S51
Figure 47. High-field fragments of ^{13}C NMR spectrum (100.6 MHz, $CDCl_3$ + 10% acetone-d_6) of compound (**3b**).	S52
Figure 48. ^{13}C-{^{1}H} and ^{13}C NMR spectra (100.6 MHz, $CDCl_3$ + 10% acetone-d_6) of compound (**3b**).	S53
Figure 49. Fragments of ^{13}C-{^{1}H} and ^{13}C NMR spectra (100.6 MHz, $CDCl_3$ + 10% acetone-d_6) of compound (**3b**).	S54
Figure 50. ^{1}H NMR spectrum (400 MHz, $CDCl_3$) of compound (**3c**).	S55
Figure 51. ^{1}H-{^{31}P} NMR spectrum (400 MHz, $CDCl_3$) of compound (**3c**).	S56
Figure 52. High field fragment of ^{1}H and ^{1}H-{^{31}P} NMR spectra (400 MHz, $CDCl_3$) of compound (**3c**).	S57
Figure 53. ^{31}P-{^{1}H} NMR spectrum (162.0 MHz, $CDCl_3$) of compound (**3c**).	S58
Figure 54. ^{13}C-{^{1}H} NMR spectrum (100.6 MHz, $CDCl_3$) of compound (**3c**).	S59
Figure 55. ^{13}C-{^{1}H} and ^{13}C-{^{1}H}-dept NMR spectra (100.6 MHz, $CDCl_3$) of compound (**3c**).	S60
Figure 56. ^{31}P-{^{1}H} NMR spectrum (400 MHz, CD_3CN) of compound (**3d**).	S61
Figure 57. ^{31}P-{^{1}H} NMR spectrum (162.0 MHz, CD_3CN/ $CDCl_3$ = 1/1) of compound (**3d**).	S62
Figure 58. ^{31}F NMR spectrum (376.5 MHz, CD_3CN/ $CDCl_3$ = 1/1) of compound (**3d**).	S63
Figure 59. ^{13}C-{^{1}H} NMR spectrum (100.6 MHz, CD_3CN/ $CDCl_3$ = 1/1) of compound (**3d**).	S64
Figure 60. ^{13}C NMR spectrum (100.6 MHz, CD_3CN/ $CDCl_3$ = 1/1) of compound (**3d**).	S65
Figure 61. Low-field fragments of ^{13}C NMR spectrum (100.6 MHz, CD_3CN/ $CDCl_3$ = 1/1) of compound (**3d**).	S66
Figure 62. ^{13}C, ^{13}C-{^{1}H} and ^{13}C-{^{1}H}-dept NMR spectra (100.6 MHz, CD_3CN/ $CDCl_3$ = 1/1) of compound (**3d**).	S67
Figure 63. Low-field fragment of ^{13}C, ^{13}C-{^{1}H} and ^{13}C-{^{1}H}-dept NMR spectra (100.6 MHz, CD_3CN/ $CDCl_3$ = 1/1) of compound (**3d**).	S68
Figure 64. High-field fragment of ^{13}C, ^{13}C-{^{1}H} and ^{13}C-{^{1}H}-dept NMR spectra (100.6 MHz, CD_3CN/ $CDCl_3$ = 1/1) of compound (**3d**).	S69
Figure 65. ^{1}H NMR spectrum (400 MHz, $CDCl_3$) of compound (**3f**).	S70
Figure 66. ^{31}P-{^{1}H} NMR spectrum (162.0 MHz, $CDCl_3$) of compound (**3f**).	S71
Figure 67. ^{19}F NMR spectrum (376.5 MHz, $CDCl_3$) of compound (**3f**).	S72
Figure 68. ^{13}C-{^{1}H} NMR spectrum (100.6 MHz, $CDCl_3$) of compound (**3f**).	S73
Figure 69. ^{13}C NMR spectrum (100.6 MHz, $CDCl_3$) of compound (**3f**).	S74
Figure 70. The 25-78 and 56-67 ppm regions of ^{13}C NMR spectrum (100.6 MHz, $CDCl_3$) of compound (**3f**).	S75
Figure 71. ^{13}C and ^{13}C -{^{1}H} NMR spectra (100.6 MHz, $CDCl_3$) of compound (**3f**).	S76
Figure 72. The 72-78 and 114-127 ppm regions of ^{13}C-{^{1}H} and ^{13}C NMR spectra (100.6 MHz, $CDCl_3$) of compound (**3f**).	S77
Figure 73. The 25-31 and 56-68 ppm regions of ^{13}C-{^{1}H} and ^{13}C NMR spectra (100.6 MHz, $CDCl_3$) of compound (**3f**).	S78
Figure 74. ^{13}C-{^{1}H} and ^{13}C-{^{1}H}-dept NMR spectra (100.6 MHz, $CDCl_3$) of compound (**3f**).	S79

Figure 75. ^{1}H NMR spectrum (400 MHz, $CDCl_3$) of compound (**3g**).	S80
Figure 76. ^{31}P-{^{1}H} NMR spectrum (162.0 MHz, $CDCl_3$) of compound (**3g**).	S81
Figure 77. ^{19}F NMR spectrum (376.5 MHz, $CDCl_3$) of compound (**3g**).	S82
Figure 78. ^{13}C-{^{1}H} NMR spectrum (100.6 MHz, $CDCl_3$) of compound (**3g**).	S83
Figure 79. ^{13}C NMR spectrum (100.6 MHz, $CDCl_3$) of compound (**3g**).	S84
Figure 80. The 13-17, 26-30, 64-69 and 72-75 ppm regions of ^{13}C NMR spectrum (100.6 MHz, $CDCl_3$) of compound (**3g**).	S85
Figure 81. ^{13}C and ^{13}C -{^{1}H} NMR spectra (100.6 MHz, $CDCl_3$) of compound (**3g**) ($MeOCH_2CH(OH)CH_2$-$PPh_3CF_3SO_3$).	S86
Figure 82. The 64-69, 70-75 and 115-127 ppm regions of ^{13}C-{^{1}H} and ^{13}C NMR spectra (100.6 MHz, $CDCl_3$) of compound (**3g**).	S87
Figure 83. The 12-17 and 26-31 ppm regions of ^{13}C-{^{1}H} and ^{13}C NMR spectra (100.6 MHz, $CDCl_3$) of compound (**3g**).	S88
Figure 84. ^{13}C-{^{1}H} and ^{13}C-{^{1}H}-dept NMR spectra (100.6 MHz, $CDCl_3$) of compound (**3g**).	S89
Figure 85. ^{1}H NMR spectrum (400 MHz, $CDCl_3$) of compound (**3h**).	S90
Figure 86. The 3-6 ppm region of ^{1}H NMR spectrum (400 MHz, $CDCl_3$) of compound (**3h**).	S91
Figure 87. ^{31}P-{^{1}H} NMR spectrum (162.0 MHz, CH_3CN) of compound (**3h**).	S92
Figure 88. ^{19}F NMR spectrum (376.5 MHz, $CDCl_3$) of compound (**3h**).	S93
Figure 89. ^{13}C-{^{1}H} NMR spectrum (100.6 MHz, $CDCl_3$) of compound (**3h**).	S94
Figure 90. ^{13}C NMR spectrum (100.6 MHz, $CDCl_3$) of compound (**3h**).	S95
Figure 91. The 105-126 ppm region of ^{13}C NMR spectrum (100.6 MHz, $CDCl_3$) of compound (**3h**)	S96
Figure 92. The 63-79 ppm region of ^{13}C NMR spectrum (100.6 MHz, $CDCl_3$) of compound (**3h**).	S97
Figure 93. ^{13}C and ^{13}C -{^{1}H} NMR spectra (100.6 MHz, $CDCl_3$) of compound (**3h**).	S98
Figure 94. The 104-126 ppm region of ^{13}C-{^{1}H} and ^{13}C NMR spectra (100.6 MHz, $CDCl_3$) of compound (**3h**).	S99
Figure 95. The 106-118 ppm region of ^{13}C-{^{1}H} and ^{13}C NMR spectrum (100.6 MHz, $CDCl_3$) of compound (**3h**).	S100
Figure 96. High-field fragments of of ^{13}C-{^{1}H} and ^{13}C NMR spectra (100.6 MHz, $CDCl_3$) of compound (**3h**).	S101
Figure 97. ^{13}C-{^{1}H} and ^{13}C-{^{1}H}-dept NMR spectra (100.6 MHz, $CDCl_3$) of compound (**3h**).	S102
Figure 98. ^{1}H NMR spectrum (400 MHz, $CDCl_3$) of compound (**3i**).	S103
Figure 99. The 7.5-7.8 and 3.9-4.2 ppm regions of ^{1}H NMR spectrum (400 MHz, $CDCl_3$) of compound (**3i**).	S104
Figure 100. The 4.1-4.2 and 3.3-3.7 ppm regions of ^{1}H NMR spectrum (400 MHz, $CDCl_3$) of compound (**3i**).	S105
Figure 101. ^{1}H NMR spectrum (600 MHz, $CDCl_3$) of compound (**3i**).	S106
Figure 102. High-field fragment of ^{1}H NMR spectrum (600 MHz, $CDCl_3$) of compound (**3i**).	S107
Figure 103. ^{31}P-{^{1}H} NMR spectrum (242.94 MHz, $CDCl_3$) of compound (**3i**).	S108
Figure 104. ^{13}C-{^{1}H} NMR spectrum (150.9 MHz, $CDCl_3$) of compound (**3i**).	S109
Figure 105. ^{13}C NMR spectrum (150.9 MHz, $CDCl_3$) of compound (**3i**).	S110
Figure 106. The 72-75 and 116-124 ppm regions of ^{13}C NMR spectrum (150.9 MHz, $CDCl_3$) of compound (**3i**).	S111
Figure 107. ^{13}C and ^{13}C -{^{1}H} NMR spectra (150.9 MHz, $CDCl_3$) of compound (**3i**).	S112

Figure 108. Aromatic carbons region of ^{13}C-{^{1}H} and ^{13}C NMR spectra (150.9 MHz, $CDCl_3$) of compound (**3i**).	S113
Figure 109. The 117-124 ppm region of ^{13}C-{^{1}H} and ^{13}C NMR spectra (150.9 MHz, $CDCl_3$) of compound (**3i**).	S114
Figure 110. The 64-75 ppm region of ^{13}C-{^{1}H} and ^{13}C NMR spectra (150.9 MHz, $CDCl_3$) of compound (**3i**).	S115
Figure 111. High-field fragment of ^{13}C-{^{1}H} and ^{13}C NMR spectra (150.9 MHz, $CDCl_3$) of compound (**3i**).	S116
Figure 112. ^{13}C-{^{1}H} and ^{13}C-{^{1}H}-dept NMR spectra (150.9 MHz, $CDCl_3$) of compound (**3i**).	S117
Figure 113. ^{1}H NMR spectrum (400 MHz, $CDCl_3$) of compound (**3j**).	S118
Figure 114. ^{31}P-{^{1}H} NMR spectrum (162.0 MHz, $CDCl_3$) of compound (**3j**).	S119
Figure 115. ^{19}F NMR spectrum (376.5 MHz, $CDCl_3$) of compound (**3j**).	S120
Figure 116. ^{13}C-{^{1}H} NMR spectrum (100.6 MHz, $CDCl_3$) of compound (**3j**).	S121
Figure 117. ^{13}C NMR spectrum (100.6 MHz, $CDCl_3$) of compound (**3j**).	S122
Figure 118. The 11-16, 63-67 and 70-76 ppm regions of ^{13}C NMR spectrum (100.6 MHz, $CDCl_3$) of compound (**3j**).	S123
Figure 119. The 20-28 and 27-34 ppm regions of ^{13}C and ^{13}C -{^{1}H} NMR spectra (150.9 MHz, $CDCl_3$) of compound (**3j**).	S124
Figure 120. ^{13}C-{^{1}H} and ^{13}C NMR spectra (100.6 MHz, $CDCl_3$) of compound (**3j**).	S125
Figure 121. The 70-76 and 115-126 ppm regions of ^{13}C-{^{1}H} and ^{13}C NMR spectra (150.9 MHz, $CDCl_3$) of compound (**3j**).	S126
Figure 122. The 11-17 and 64-67 ppm regions of ^{13}C-{^{1}H} and ^{13}C NMR spectra (100.6 MHz, $CDCl_3$) of compound (**3j**).	S127
Figure 123. High-field fragment of ^{13}C-{^{1}H} and ^{13}C NMR spectra (100.6 MHz, $CDCl_3$) of compound (**3j**).	S128
Figure 124. ^{13}C-{^{1}H} and ^{13}C-{^{1}H}-dept NMR spectra (100.6 MHz, $CDCl_3$) of compound (**3j**).	S129
Figure 125. ^{1}H NMR spectrum (400 MHz, $CDCl_3$) of compound (**3k**).	S130
Figure 126. ^{31}P-{^{1}H} NMR spectrum (162.0 MHz, $CDCl_3$) of compound (**3k**).	S131
Figure 127. ^{13}C-{^{1}H} NMR spectrum (100.6 MHz, $CDCl_3$) of compound (**3k**).	S132
Figure 128. High-field fragment of ^{13}C-{^{1}H} NMR spectrum (100.6 MHz, $CDCl_3$) of compound (**3k**).	S133
Figure 129. ^{1}H NMR spectrum (400 MHz, $CDCl_3$) of compound (**3l**).	S134
Figure 130. ^{31}P-{^{1}H} NMR spectrum (162.0 MHz, $CDCl_3$) of compound (**3l**).	S135
Figure 131. ^{13}C-{^{1}H} NMR spectrum (100.6 MHz, $CDCl_3$) of compound (**3l**).	S136
Figure 132. High-field fragment of ^{13}C-{^{1}H} NMR spectrum (100.6 MHz, $CDCl_3$) of compound (**3l**).	S137
Figure 133. ^{1}H NMR spectrum (400 MHz, $CDCl_3$) of compound (**4a**).	S138
Figure 134. The fragment of ^{1}H NMR spectrum (400 MHz, $CDCl_3$) of compound (**4a**).	S139
Figure 135. ^{31}P-{^{1}H} NMR spectrum (162.0 MHz, CH_3CN) of compound (**4a**).	S140
Figure 136. ^{13}C-{^{1}H} NMR spectrum (100.6 MHz, $CDCl_3$) of compound (**4a**).	S141
Figure 137. ^{13}C NMR spectrum (100.6 MHz, $CDCl_3$) of compound (**4a**).	S142
Figure 138. The 56-66 ppm region of ^{13}C NMR spectrum (100.6 MHz, $CDCl_3$) of compound (**4a**).	S143
Figure 139. ^{13}C and ^{13}C -{^{1}H} NMR spectra (100.6 MHz, $CDCl_3$) of compound (**4a**).	S144
Figure 140. The 104-126 ppm region of ^{13}C-{^{1}H} and ^{13}C NMR spectra (100.6 MHz, $CDCl_3$) of compound (**4a**).	S145

Figure 141. ^{13}C-{^{1}H} and ^{13}C-{^{1}H}-dept NMR spectra (100.6 MHz, $CDCl_3$) of compound (**4a**).	S146
Figure 142. ^{1}H NMR spectrum (400 MHz, $CDCl_3$) of compound (**4b**).	S147
Figure 143. ^{31}P-{^{1}H} NMR spectrum (162.0 MHz, CH_3CN) of compound (**4b**).	S148
Figure 144. ^{13}C-{^{1}H} NMR spectrum (100.6 MHz, $CDCl_3$) of compound (**4b**).	S149
Figure 145. ^{13}C NMR spectrum (100.6 MHz, $CDCl_3$) of compound (**4b**).	S150
Figure 146. The 26-30, 64-69 and 72-76 ppm regions of ^{13}C NMR spectrum (100.6 MHz, $CDCl_3$) of compound (**4b**).	S151
Figure 147. ^{13}C and ^{13}C-{^{1}H} NMR spectra (100.6 MHz, $CDCl_3$) of compound (**4b**).	S152
Figure 148. The 117-136 ppm region of ^{13}C-{^{1}H} and ^{13}C NMR spectra (100.6 MHz, $CDCl_3$) of compound (**4b**).	S153
Figure 149. ^{13}C-{^{1}H} and ^{13}C-{^{1}H}-dept NMR spectra (100.6 MHz, $CDCl_3$) of compound (**4b**).	S154
Figure 150. ^{1}H NMR spectrum (400 MHz, $CDCl_3$) of compound (**4c**).	S155
Figure 151. ^{31}P-{^{1}H} NMR spectrum (162.0 MHz, CD_3CN) of compound (**4c**).	S156
Figure 152. ^{13}C-{^{1}H} NMR spectrum (100.6 MHz, $CDCl_3$) of compound (**4c**).	S157
Figure 153. ^{13}C NMR spectrum (100.6 MHz, $CDCl_3$) of compound (**4c**)	S158
Figure 154. High-field fragments of ^{13}C NMR spectrum (100.6 MHz, $CDCl_3$) of compound (**4c**).	S159
Figure 155. ^{13}C and ^{13}C -{^{1}H} NMR spectra (100.6 MHz, $CDCl_3$) of compound (**4c**).	S160
Figure 156. High-field region of ^{13}C and ^{13}C -{^{1}H} NMR spectra (100.6 MHz, $CDCl_3$) of compound (**4c**).	S161
Figure 157. ^{13}C-{^{1}H} and ^{13}C-{^{1}H}-dept NMR spectra (100.6 MHz, $CDCl_3$) of compound (**4c**).	S162
Figure 158. ^{1}H NMR spectrum (400 MHz, $CDCl_3$) of compound (**4d**).	S163
Figure 159. ^{31}P-{^{1}H} NMR spectrum (162.0 MHz, CD_3CN) of compound (**4d**).	S164
Figure 160. ^{13}C-{^{1}H} NMR spectrum (100.6 MHz, CD_3CN) of compound (**4d**).	S165
Figure 161. ^{13}C NMR spectrum (100.6 MHz, CD_3CN) of compound (**4d**).	S166
Figure 162. High-field fragments of ^{13}C NMR spectrum (100.6 MHz, CD_3CN) of compound (**4d**).	S167
Figure 163. ^{13}C and ^{13}C -{^{1}H} NMR spectra (100.6 MHz, CD_3CN) of compound (**4d**).	S168
Figure 164. ^{13}C and ^{13}C -{^{1}H} NMR spectra (100.6 MHz, CD_3CN) of compound (**4d**).	S169
Figure 165. High-field region of ^{13}C-{^{1}H} and ^{13}C NMR spectra (100.6 MHz, CD_3CN) of compound (**4d**).	S170
Figure 166. ^{13}C-{^{1}H} and ^{13}C-{^{1}H}-dept NMR spectra (100.6 MHz, CD_3CN) of compound (**4d**).	S171
Figure 167. ^{31}P-{^{1}H} spectrum (242.94 MHz, $CDCl_3$ + 10% MeCN) of compound (**7**).	S172
Figure 168. ^{1}H NMR spectrum (242.94 MHz, CD_3CN) of compound (**7**).	S173
Figure 169. High-field fragment of ^{1}H NMR spectrum (242.94 MHz, CD_3CN) of compound (**7**).	S174
Figure 170. ^{13}C-{^{1}H} NMR spectrum (100.6 MHz, $CDCl_3$+ 20% DMSO-d_6) of compound (**7**).	S175
Figure 171. ^{13}C NMR spectrum (100.6 MHz, $CDCl_3$+ 20% DMSO-d_6) of compound (**7**).	S176
Figure 172. ^{13}C-{^{1}H} and ^{13}C-{^{1}H}-dept NMR spectra (100.6 MHz, $CDCl_3$+ 20% DMSO-d_6) of compound (**7**).	S177
Figure 173. ^{1}H NMR spectrum (600 MHz, $CDCl_3$ + 10% MeCN) of compound (**7**).	S178

Figure 174. ^{13}C-{^{1}H} NMR spectrum (150.9 MHz, $CDCl_3$+ 10% MeCN) of compound (**7**).	S179
Figure 175. ^{13}C NMR spectrum (150.9 MHz, $CDCl_3$+ 10% MeCN) of compound (**7**).	S180
Figure 176. ^{13}C and ^{13}C-{^{1}H} NMR spectra (150.9 MHz, $CDCl_3$+ 10% MeCN) of compound (**7**).	S181
Figure 177. Fragments of ^{13}C and ^{13}C-{^{1}H} NMR spectra (150.9 MHz, $CDCl_3$+ 10% MeCN) of compound (**7**).	S182
Figure 178. ^{13}C-{^{1}H} and ^{13}C-{^{1}H}-dept NMR spectra (150.9 MHz, $CDCl_3$+ 10% MeCN) of compound (**7**).	S183

Numbers of investigated compounds

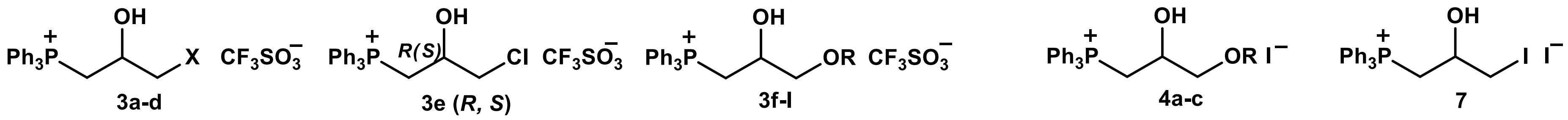

X = Cl (a), Br (b), F (c), I (d)

R = Me (f), Et (g), $CHF_2CF_2CH_2$ (h), CH_2=$CHCH_2$ (i), $C_{10}H_{21}$ (j), $C_{15}H_{31}CO$ (k), $C_{17}H_{35}CO$ (l)

R = Me (a), Et (b), $C_{10}H_{21}$ (c)

General remarks for XRD investigation

The X-ray diffraction data for the crystals (**3b**, **3c**, **3d**, **3f-3j**, **4a**) were collected on a Bruker D8 QUEST CCD diffractometer at temperature 100(2) K, (**3a**) and (**3e**) were collected on a Bruker Kappa Apex II CCD diffractometer at room temperature (296(2) K),in the ω and φ-scan modes using graphite monochromated MoK_α (λ = 0.71073 Å) radiation. Data for the crystals (**3**, **4a**) were corrected for the absorption effect using SADABS program [1]. Data collection: images were indexed and integrated using the APEX2 data reduction package [2].The structures were solved by direct method using SHELXT [3] and refined by the full matrix least-squares using SHELXL [4] programs. The X-ray diffraction data for the crystals (**3e**, ***S***) and (**3e**, ***R***) were collected on a Enraf-Nonius CAD-4 diffractometer at room temperature (296(2) K), in the ω/2θ-scan modes using graphite monochromated MoK_α (λ = 0.71073 Å) radiation. The structures were solved by direct method and refined by the full matrix least-squares using MOLEN [5] programs.

The X-ray diffraction data for the crystal **(7)** were collected on a XtaLAB Synergy, Single source at home/near, HyPix diffractometer at temperature 100(2) K using graphite monochromated CuK_α (λ = 1.54184Å) radiation. The structure was solved by direct method using SHELXS and refined by the full matrix least-squares using SHELXL programs. Hydrogen atoms in all structures were inserted at calculated positions and refined as riding atoms. Hydrogen atoms in the hydroxyl-groups were solved from difference Fourier maps and refined with fixed bond length and angles with rotation around C-O bonds.

Analysis of the intermolecular interactions was performed using the program PLATON [7]. Mercury program package [8] was used for figures preparation. Crystallographic data and detail of the structures refinement are shown in the Tables 1-4. Crystals of compounds (**3b**, **3c**) and (**3d**) are isostructural, and crystals of the compounds (**4**) and (**7**) are isostructural.

Crystallographic data for the investigated structures see Supplementary Materials and have been deposited in the Cambridge Crystallographic Data Centre as supplementary publication nos. CCDC 2110389-2110402, respectively. Copies of the data can be obtained free of charge upon application to the CCDC (12 Union Road, Cambridge CB2 1EZ UK. Fax: (internat.) +44-1223/336-033; E-mail: deposit@ccdc.cam.ac.uk).

1. Sheldrick, G. SADABS, Program for empirical X-ray absorption correction, Bruker-Nonius, 2004.
2. APEX2 (Version 2.1), SAINTPlus, Data Reduction and Correction Program (Version 7.31A), BrukerAXS Inc., Madison, Wisconsin, USA, 2006.
3. G.M.Sheldrick. Acta Crystallogr. Section A. 2015, **7**, Part 1, 3-8.
4. G.M.Sheldrick. Acta Crystallogr. Sect. C. 2015, **71**, 3-8.
5. L.H. Straver, A.J. Schierbeek, MOLEN. Structure Determination System. Program Description, Nonius B.V., 1994, 1, 180 p.
6. L.J.Farrugia, J. Appl. Crystallogr. 2012, **45**, 849-854.
7. A.L.Spek. Acta Crystallogr. Sect. D. 2009, **65**, 148-155.
8. C.F.Macrae, P.R.Edgington, P.McCabe, E.Pidcock, G.P.Shields, R.Taylor, M.Towler, J. van de Streek. J. Appl. Crystallogr.2006, **39**, 453-459.

Table 1. Crystal Data and Refinement Details for compounds (**3b**, **3c**, **3d**, **3e** (***S***)).

Parameter	**3b**	**3c**	**3d**	**3e**, ***S***
Color, habitus	Yellow, prismatic	Colorless, prismatic	Yellow, prismatic	Colorless, plates
Crystal dimensions, mm	0.30 × 0.10 × 0.10	0.40 × 0.30 × 0.10	0.20 × 0.10× 0.05	0.50 × 0.20 × 0.05
Moiety Formula<br>Sum Formula	$C_{21}H_{21}BrOP^+$, $CF_3O_3S^-$<br>$C_{22}H_{21}F_3BrO_4PS$	$C_{21}H_{21}FOP^+$, $CF_3O_3S^-$<br>$C_{22}H_{21}F_4O_4PS$	$C_{21}H_{21}IOP^+$, $CF_3O_3S^-$<br>$C_{22}H_{21}F_3IO_4PS$	$C_{21}H_{21}ClOP^+$, $CF_3O_3S^-$<br>$C_{22}H_{21}F_3ClO_4PS$
Molecular mass	549.32	488.42	596.32	504.89
Melting point (°C)	161-162	137	178-180	146.147
Molecular mass	549.32	488.42	596.32	504.87
Temperature	100(2) K	100(2) K	100(2) K	296(2) K
Crystal system	Monoclinic	Monoclinic	Monoclinic	Monoclinic
Space group	$P2_1/n$	$P2_1/n$	$P2_1/n$	$P2_1$
Cell parameters, Å, angles, degrees	*a* = 9.5904(8),<br>*b* = 18.0823 (12),<br>*c* = 14.0321(12)<br>β = 109.527(3)°	*a* = 10.0953(3),<br>*b* = 16.3635(4),<br>*c* = 13.6011(3)<br>β = 106.271(1)°	*a* = 9.767(3),<br>*b* = 17.759(6),<br>*c* = 14.183(5)<br>β = 109.371(9)°	*a* = 9.2585(17),<br>*b* = 25.998(5),<br>*c* = 10.0176(18)<br>β = 107.135(2)°
Cell volume, $Å^3$	2293.4(3)	2156.8(1)	2320.8(13)	2304.2(7)
Z	4	4	4	4 (two independent molecules)
D(calc), g/см^3	1.591	1.504	1.707	1.455
μMo, мм$^{-1}$	2.004	0.286	1.590	0.377

Absorption correction	multi-scan	multi-scan	multi-scan	multi-scan
Radiation (λ, Å)	MoK_{α}, 0.71073	MoK_{α}, 0.71073	MoK_{α}, 0.71073	MoK_{α}, 0.71073
F(000)	1112	1008	1184	1040
Total reflections, independent reflections	47675, 6086	88186, 5697	15093, 4100	21481, 11344
R(int)	0.057	0.073	0.061	0.040
Observed reflections, I > 2σ(I)	5351	5173	3056	9128
R-factors, I > 2σ(I)	R^1 = 0.0325, wR^2 = 0.0829	R^1 = 0.0327, wR^2 = 0.0908	R^1 = 0.0723, wR^2 = 0.1839	R^1 = 0.0533, wR^2 = 0.1197
R-factors, (all)	R^1 = 0.0388, wR^2 = 0.0858	R^1 = 0.0362, wR^2 = 0.0935	R^1 = 0.0977, wR^2 = 0.2009	R^1 = 0.0707, wR^2 = 0.1305
goodness of fit	1.029	1.006	1.049	1.019
Refines number parameters, N par	293	293	290	579
Data set	$-11 \le h \le 13$, $-4 \le k \le 24$, $-19 \le l \le 16$	$-13 \le h \le 13$, $-22 \le k \le 22$, $-18 \le l \le 18$	$-11 \le h \le 9$, $-21 \le k \le 21$, $-7 \le l \le 17$	$-12 \le h \le 12$, $-35 \le k \le 35$, $-13 \le l \le 13$
Theta Min-Max [Deg]	1.9, 29.0	2.0, 29.0	1.9, 26.0	2.1, 29.0
Completeness to theta	0.999	0.994	0.904	0.999
Min. and Max. Resd. Dens., e·Å^{-3}	–0.96, 1.11	–0.38, 0.43	–1.19, 1.43	–0.38, 0.98
Flack parameter	–	–	–	0.00(4)
CCDC deposit	2110391	2110393	2110394	2110390

Table 2. Crystal Data and Refinement Details for compounds (**3a**) (anomalous conglomerate), (**3e**, ***S***), (**3e**, ***R***)).

Parameter	**3e**, ***R***	**3e**, ***S***	**3e**, ***S***	**3a**
Color, habitus	Colorless, prismatic	Yellow, prismatic	Colorless, plates	Yellow, prismatic
Crystal dimensions, мм	0.40 × 0.30 × 0.10	0.30 × 0.10 × 0.10	0.50 × 0.20 × 0.05	0.20 × 0.10 × 0.05
Moiety Formula; Sum Formula	$C_{21}H_{21}ClOP^+$, $CF_3O_3S^-$; $C_{22}H_{21}F_3ClO_4PS$			
Melting point (°C)	137-138	137-138	137-138	146-147
Molecular mass	504.87			
Temperature	296(2) K	296(2) K	296(2) K	296(2) K
Crystal system	Monoclinic			
Space group	$P2_1$			

Cell parameters, Å, angles, degrees	*a* = 9.314(2), *b* = 26.328(6), *c* = 10.089(2) β = 106.327(2)°	*a* = 9.2964(9), *b* = 26.293(2), *c* = 10.0754(9) β = 106.378(1)°	*a* = 9.2585(17), *b* = 25.998(5), *c* = 10.0176(18) β = 107.135(2)°	*a* = 9.5483(17), *b* = 17.900(3), *c* = 13.970(2) β = 108.873(2)°
Cell volume, $Å^3$	2374.3(9)	2362.8(4)	2304.2(7)	2259.3(6)
Z	4 (two independent molecules)			
D(calc), g/см^3	1.412	1.419	1.455	1.484
μMo, mm^{-1}	0.366	0.367	0.377	0.384
Absorption correction	multi-scan			
Radiation λ, Å)	MoK$_\alpha$, 0.71073			
F(000)	1040			
Total reflections, independent reflections	32388, 10304	32412, 10138	21481, 11344	10385, 8537
R(int)	0.0288	0.0425	0.040	0.037
Observed reflections, I > 2σ(I)	7720	6462	9128	6316
R-factors, I > 2σ(I)	R^1 = 0.0605, wR^2 = 0.1643	R^1 = 0.0619, wR^2 = 0.1565	R^1 = 0.0533, wR^2 = 0.1197	R^1 = 0.0723, wR^2 = 0.1839
R-factors, (all)	R^1 = 0.0814, wR^2 = 0.1819	R^1 = 0.1048, wR^2 = 0.1884	R^1 = 0.0707, wR^2 = 0.1305	R^1 = 0.0977, wR^2 = 0.2009
goodness of fit	1.121	0.987	1.019	1.049
Refines number parameters, N par	585	577	579	290
Dataset	$-11 \le h \le 11$, $-33 \le k \le 33$, $-12 \le l \le 12$	$-11 \le h \le 11$, $-33 \le k \le 33$, $-12 \le l \le 12$	$-12 \le h \le 12$, $-35 \le k \le 35$, $-13 \le l \le 13$	$-12 \le h \le 11$, $-22 \le k \le 22$, $-17 \le l \le 15$
Theta Min-Max [Deg]	2.1, 27.0	2.1, 27.0	2.1, 29.0	1.9, 27.1
Completeness to theta	0.994	0.999	0.999	1.01
Min. and Max. Resd. Dens., e·Å^{-3}	–0.38, 0.43	–0.39, 0.47	–0.38, 0.98	–1.19, 1.43
Flack parameter	–0.01(8)	0.06(9)	0.00(4)	–0.11(9)
CCDC deposit	2110401	2110402	2110390	2110389

Table 3. Crystal Data and Refinement Details for compounds (**3f**, **3g**, **3i**, **3j**).

Parameter	**3f**	**3g**	**3i**	**3j**
Color, habitus	Colorless, prismatic	Colorless, formless	Colorless, formless	Colorless, slides
Crystal dimensions, mm	0.50 × 0.30 × 0.20	0.10 × 0.10 × 0.05	0.20 × 0.20 × 0.05	0.20 × 0.10 × 0.04

Moiety Formula<br>Sum Formula	$C_{22}H_{24}O_2P^+$, $CF_3O_3S^-$<br>$C_{23}H_{24}F_3O_5PS$	$C_{23}H_{25}O_2P^+$, $CF_3O_3S^-$<br>$C_{24}H_{25}F_3ClO_5PS$	$C_{24}H_{26}O_2P^+$, $CF_3O_3S^-$<br>$C_{25}H_{26}F_3O_5PS$	$C_{31}H_{42}O_2P^+$, $CF_3O_3S^-$<br>$C_{32}H_{42}F_3O_5PS$
Melting point (°C)	122-124	99-101	124-125	78-79
Molecular mass	500.45	513.47	526.49	626.69
Temperature	100(2) K	100(2) K	293(2) K	100(2) K
Crystal system	Monoclinic	Monoclinic	Monoclinic	Monoclinic
Space group	$P2_1/n$	Pn	Cc	$P2_1/c$
Cell parameters, Å, angles, degrees	*a* = 9.5710(7),<br>*b* = 18.4248(13),<br>*c* = 13.6886(8)<br>β = 106.439(3)°	*a* = 9.5757(5),<br>*b* = 19.9551(11),<br>*c* = 13.5883(8)<br>β = 107.202(2)°	*a* = 13.5147(18),<br>*b* = 20.753(3),<br>*c* = 9.3742(12)<br>β = 108.067(5)°	*a* = 9.4703(3),<br>*b* = 26.8164(9),<br>*c* = 13.5705(5)<br>β = 110.173(2)°
Cell volume, $Å^3$	2315.2(3)	2480.4(2)	2499.5(6)	3234.9(2)
Z	4	4 (two independent molecules)	4	4
D(calc), g/см^3	1.436	1.375	1.399	1.287
μMo, мм$^{-1}$	0.266	0.250	0.250	0.204
Absorption correction	multi-scan	multi-scan	multi-scan	multi-scan
Radiation (λ, Å)	MoK_α, 0.71073	MoK_α, 0.71073	MoK_α, 0.71073	MoK_α, 0.71073
F(000)	1040	1068	1096	1328
Total reflections, independent reflections	83972, 6737	59109, 12194	58503, 5387	82297, 8018
R(int)	0.100	0.156	0.0839	0.254
Observed reflections, I > 2σ(I)	5458	7836	4969	3776
R-factors, I > 2σ(I)	R^1 = 0.0839,<br>wR^2 = 0.2220	R^1 = 0.0844,<br>wR^2 = 0.1597	R^1 = 0.0725,<br>wR^2 = 0.1854	R^1 = 0.0984,<br>wR^2 = 0.2272
R-factors, (all)	R^1 = 0.0996,<br>wR^2 = 0.2352	R^1 = 0.1420,<br>wR^2 = 0.1815	R^1 = 0.0779,<br>wR^2 = 0.1887	R^1 = 0.2049,<br>wR^2 = 0.2890
goodness of fit	1.040	1.039	1.045	1.019
Refines number parameters, N par	300	615	318	380
Dataset	$-13 \le h \le 13$, $-25 \le k \le 25$,<br>$-19 \le l \le 19$	$-12 \le h \le 12$, $-26 \le k \le 26$,<br>$-18 \le l \le 18$	$-17 \le h \le 17$, $26 \le k \le 26$,<br>$-11 \le l \le 11$	$-12 \le h \le 12$, $-35 \le k \le 35$,<br>$-18 \le l \le 18$
Theta Min-Max [Deg]	1.9, 30.0	1.9, 28.4	1.864, 26.998	1.8, 28.3
Completeness to theta	0.998	1.000	0.9985	0.997

Min. and Max. Resd. Dens., e·Å^{-3}	–1.47, 1.48	–0.66, 0.73	–0.763, 0.750	–0.58, 0.93
Flack parameter	–	0.06(6)	0.24(19)	–
CCDC deposit	2110395	2110396	2110397	2110398

Table 4. Crystal Data and Refinement Details for compounds (**4a**, **7**).

Parameter	**4a**	**7**
Color, habitus	Colorless, prismatic	Yellow, formless
Crystal dimensions, mm	0.30 × 0.30 × 0.10	0.30 × 0.20 × 0.15
Moiety Formula<br>Sum Formula	$C_{22}H_{24}O_2P^+$, I^-	$C_{21}H_{21}IOP^+$, I^-<br>$C_{21}H_{21}I_2OP$
Melting point (°C)	172-174	197-198
Molecular mass	478.28	574.15
Temperature	100(2) K	100(2) K
Crystal system	Monoclinic	Monoclinic
Space group	$P2_1/c$	$P2_1/c$
Cell parameters, Å, angles, degrees	a = 12.0217(5), b = 10.1838(4), c = 17.7359(7)<br>β = 107.512(2)°	a = 12.4823(2), b = 9.9826(2), c = 17.5396(3)<br>β = 108.489(2)°
Cell volume, Å^3	2070.71(15)	2072.73(7)
Z	4	4
D(calc), g/см^3	1.534	1.840
μMo, мм$^{-1}$	1.637	24.602 (μCu)
Absorption correction	multi-scan	multi-scan
Radiation(λ, Å)	MoK_α, 0.71073	CuK_α, 1.54184
F(000)	960	1104
Total reflections, independent reflections	76826, 5487	14038, 3896
R(int)	0.068	0.112
Observed reflections, I > 2σ (I)	4978	3688
R-factors, I > 2σ (I)	R^1 = 0.0313, wR^2 = 0.0773	R^1 = 0.0653, wR^2 = 0.1652
R-factors, (all)	R^1 = 0.0352, wR^2 = 0.0791	R^1 = 0.0670, wR^2 = 0.1681
goodness of fit	1.057	1.071
Refines number parameters, N par	236	227
Data set	$-16 \le h \le 16$, $-13 \le k \le 13$, $-24 \le l \le 24$	$-14 \le h \le 15$, $-11 \le k \le 12$, $-21 \le l \le 21$
Theta Min-Max [Deg]	1.8, 29.0	5.2, 70.1

Completeness to theta	0.997	0.991
Min. and Max. Resd. Dens., e·Å^{-3}	–0.87, 1.76	–1.72, 3.34
Flack parameter	–	–
CCDC deposit	2110399	2110400

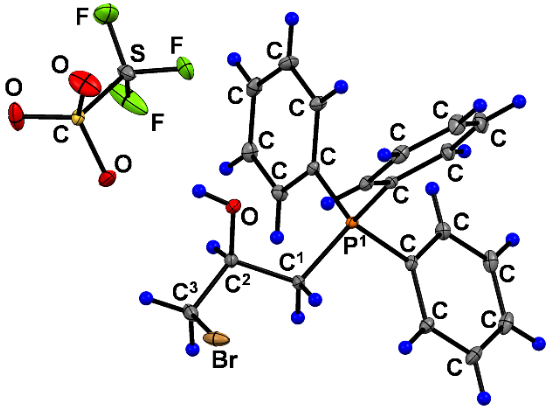

Figure. 1. Molecular geometry of independent part of crystal (**3b**).

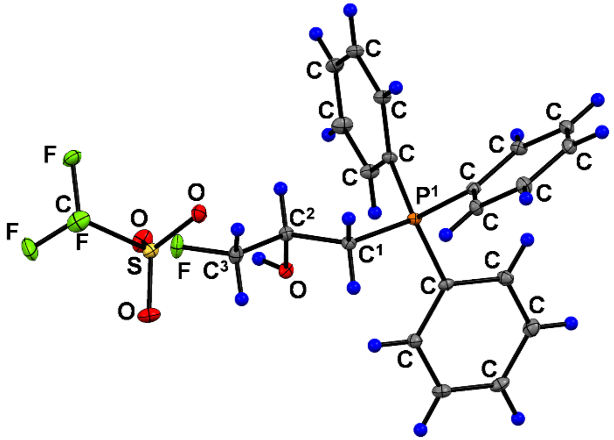

Figure 2. Molecular geometry (**3c**) in crystal.

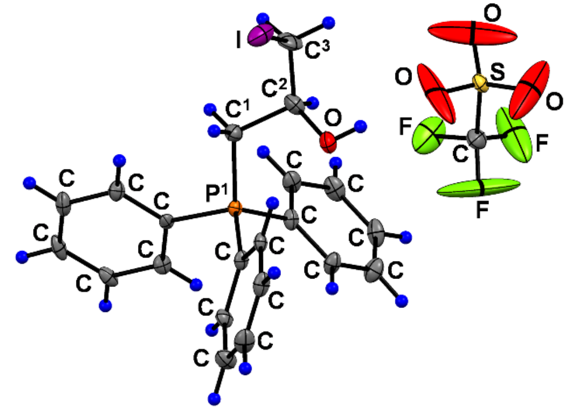

Figure 3. Molecular geometry (**3d**) in crystal.

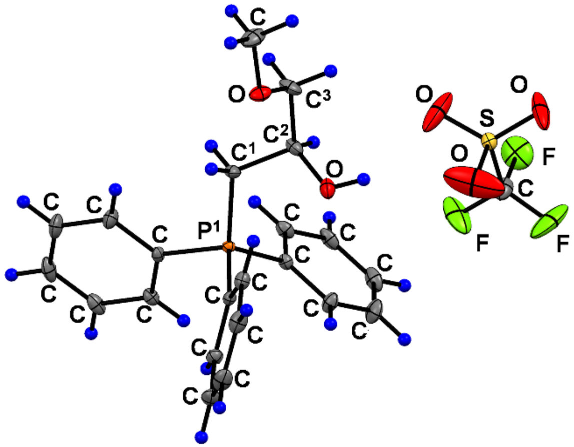

Figure 4. Molecular geometry (**3f**) in crystal.

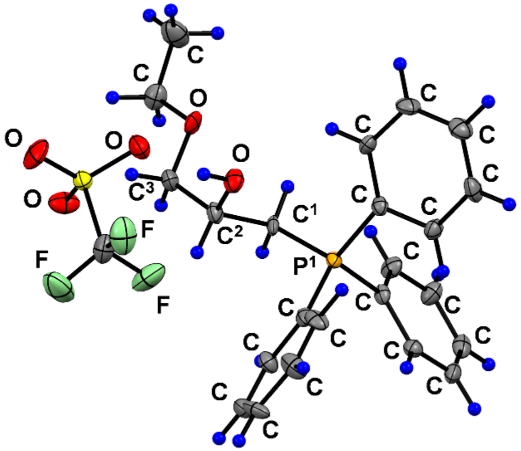

Figure 5. Molecular geometry (**3g**) in crystal. The cell contains two independent molecules, but to simplify only one molecule is shown.

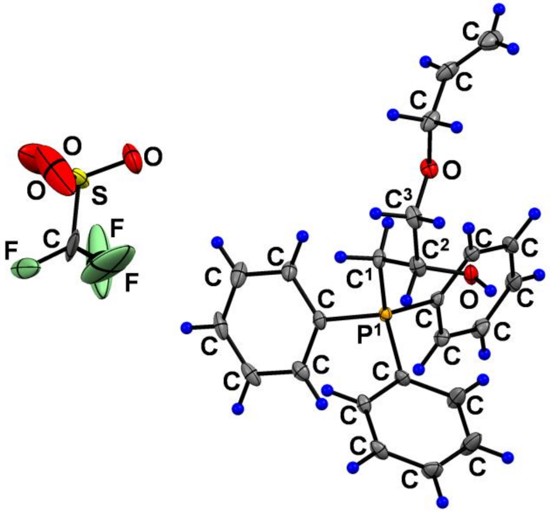

Figure 6. Molecular geometry (**3i**) in crystal.

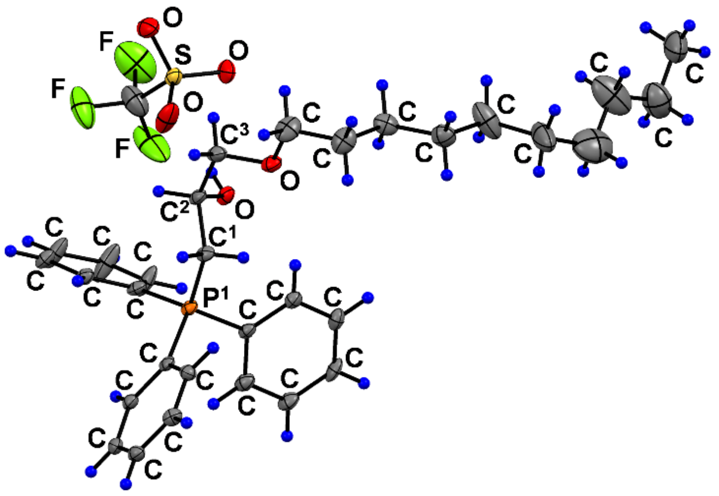

Figure 7. Molecular geometry (**3j**) in crystal.

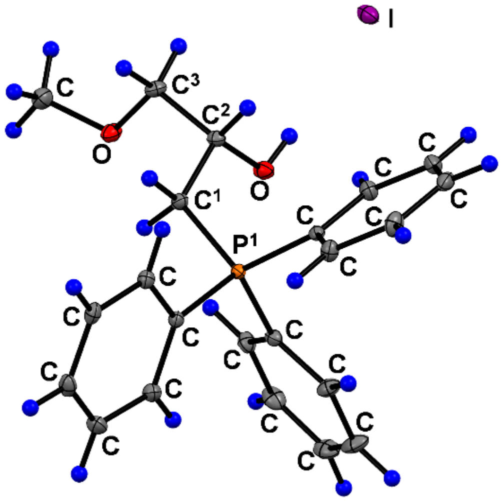

Figure 8. Molecular geometry (**4a**) in crystal.

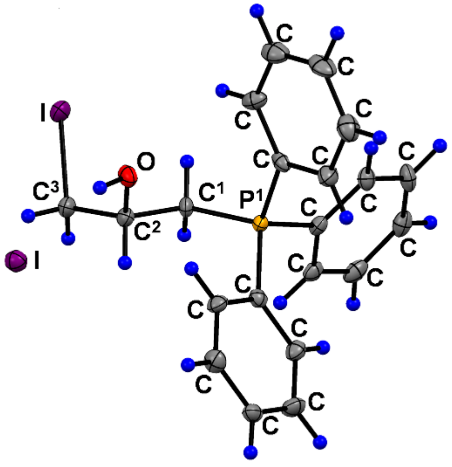

Figure 9 Molecular geometry (**7**) in crystal.

Conclusions on the spatial structure of the studied compounds

Some regularities in the spatial structure of investigated 10 compounds were formulated. The conformation and geometry of isopropanoltriphenyl phosphonium cations in crystals is the same. The propanol fragment has a transoidal conformation (the P–C^1–C^2–C^3 torsion angles are close to 180°). Hydroxyl groups of cations form hydrogen bonds with triflate or iodide anions. Closed 0D-hydrogen bond motifs were formed. Crystals of studied compounds are formed due to conventional dispersion interactions, π-stacking interactions between benzene rings are not observed. All interplanar distances are more than 3.2 Å. Distances between the benzene rings are more than 5 Å. Bi- and trifurcate hydrogen bonds with two oxygen atoms and one fluorine atom of anion are observed in crystals with triflate anions. The increase in the ellipsoid size of anisotropic displacements of oxygen atoms can be explained by the "switching" of hydrogen bond from one oxygen atom to another (disorder of hydrogen bond).

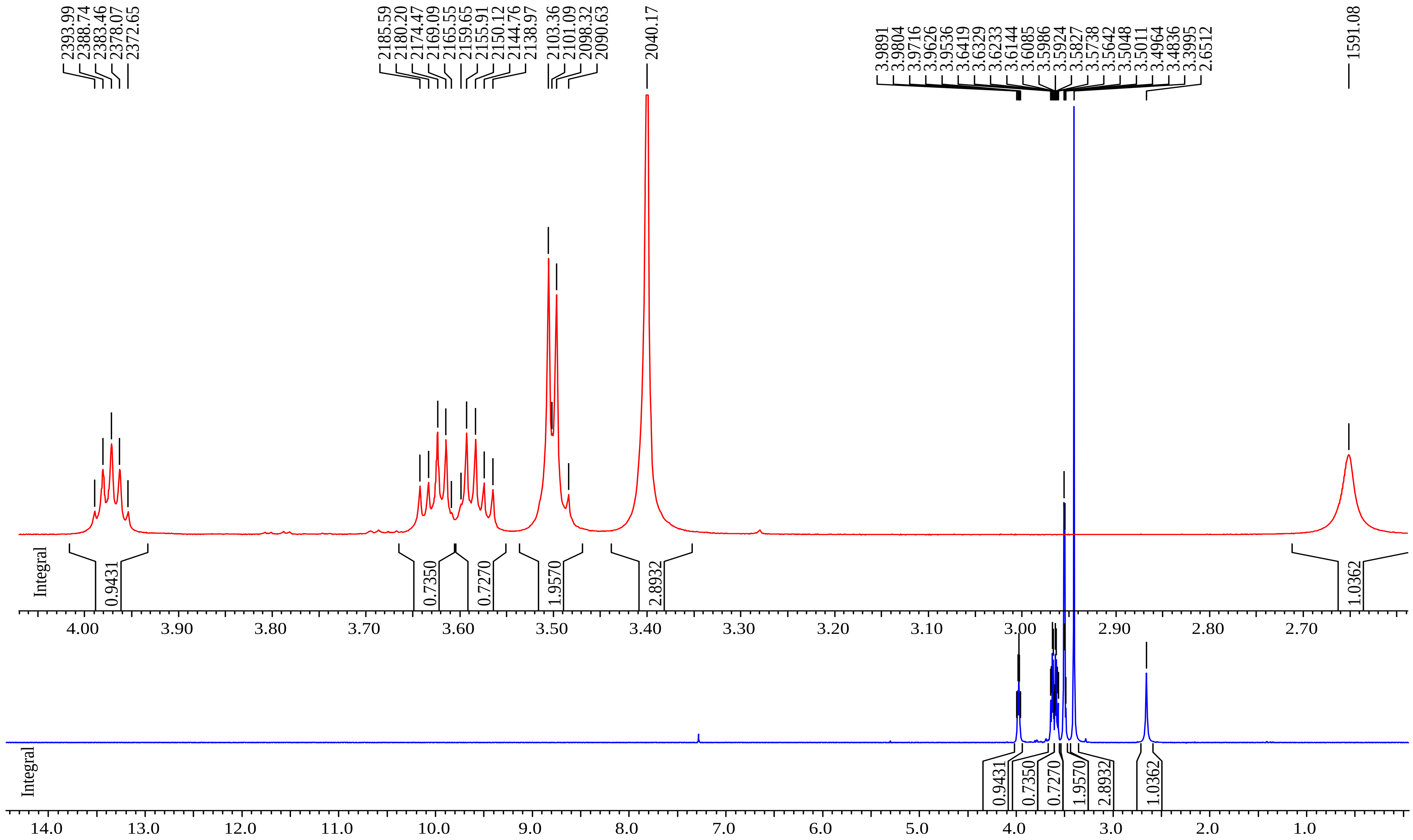

Figure 10. ^{1}H NMR spectrum (400.0 MHz, $CDCl_3$) of 1-chloro-3-methoxypropan-2-ol **(5a)**.

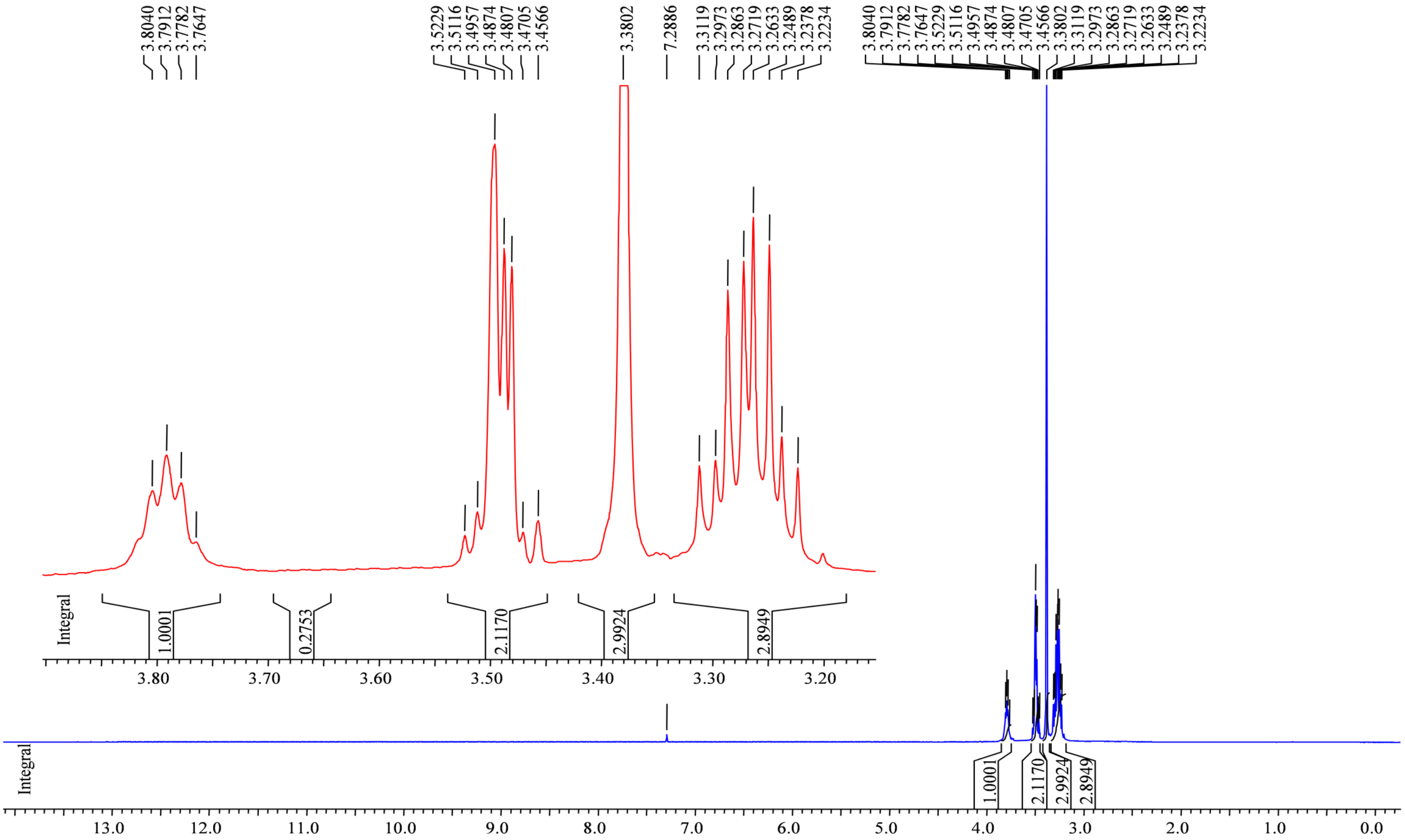

Figure 11. ^{1}H NMR spectrum (400.0 MHz, $CDCl_3$) of 1-iodo-3-methoxypropan-2-ol **(6a)**.

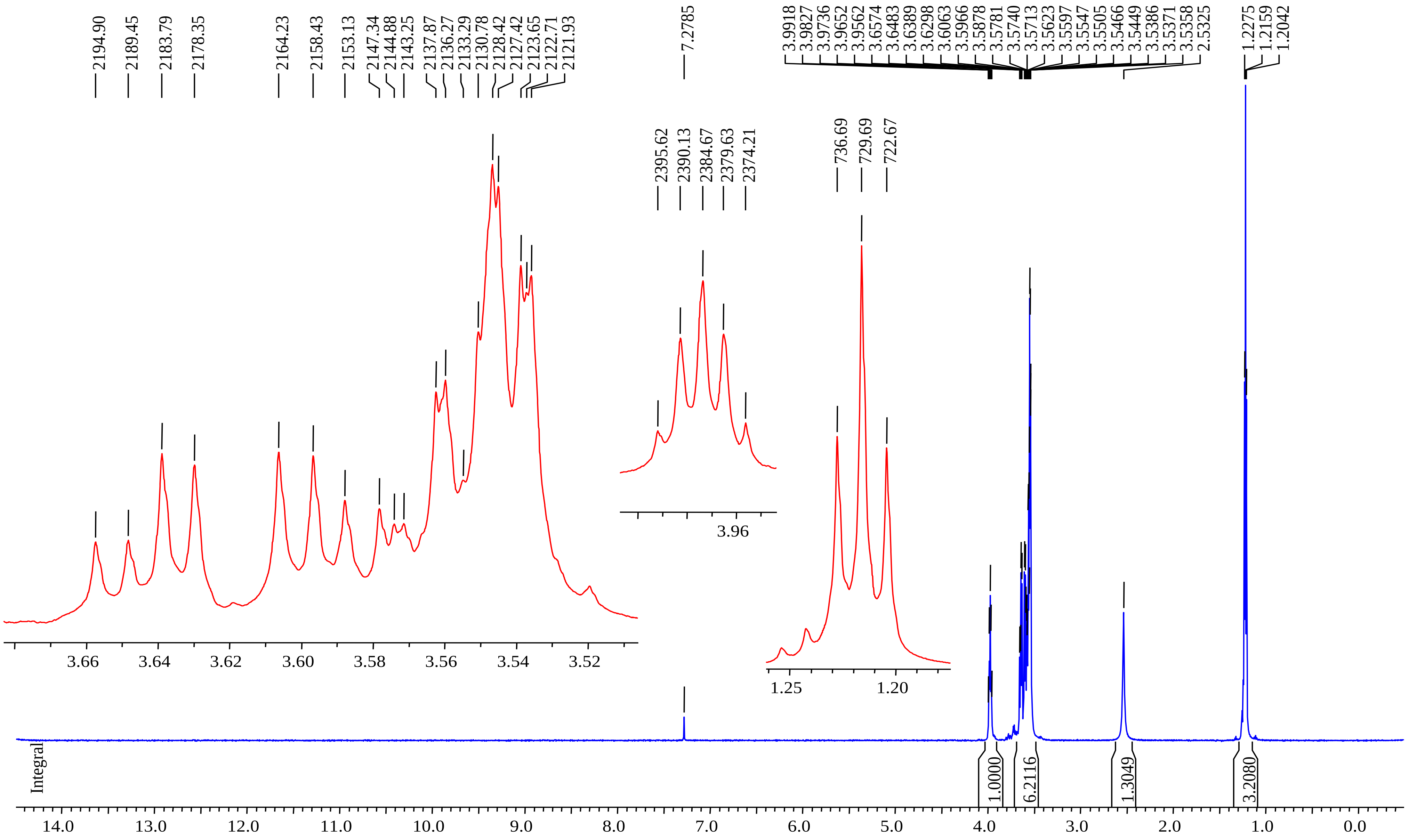

Figure 12. ^{1}H NMR spectrum (400.0 MHz, $CDCl_3$) of 1-chloro-3-ethoxypropan-2-ol (**5b**).

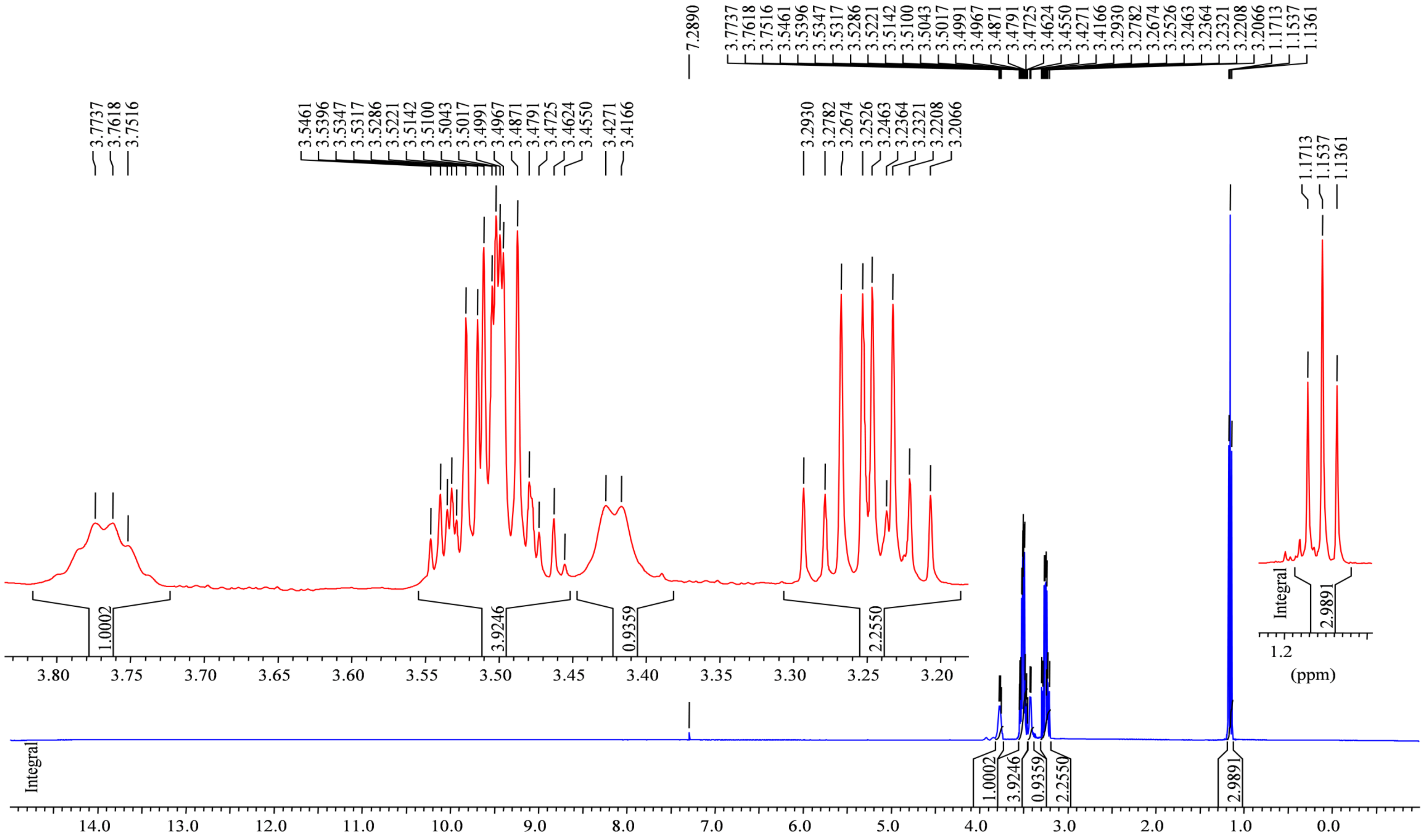

Figure 13. ^{1}H NMR spectrum (400.0 MHz, $CDCl_3$) of 1-ethoxy-3-iodopropan-2-ol (**6b**).

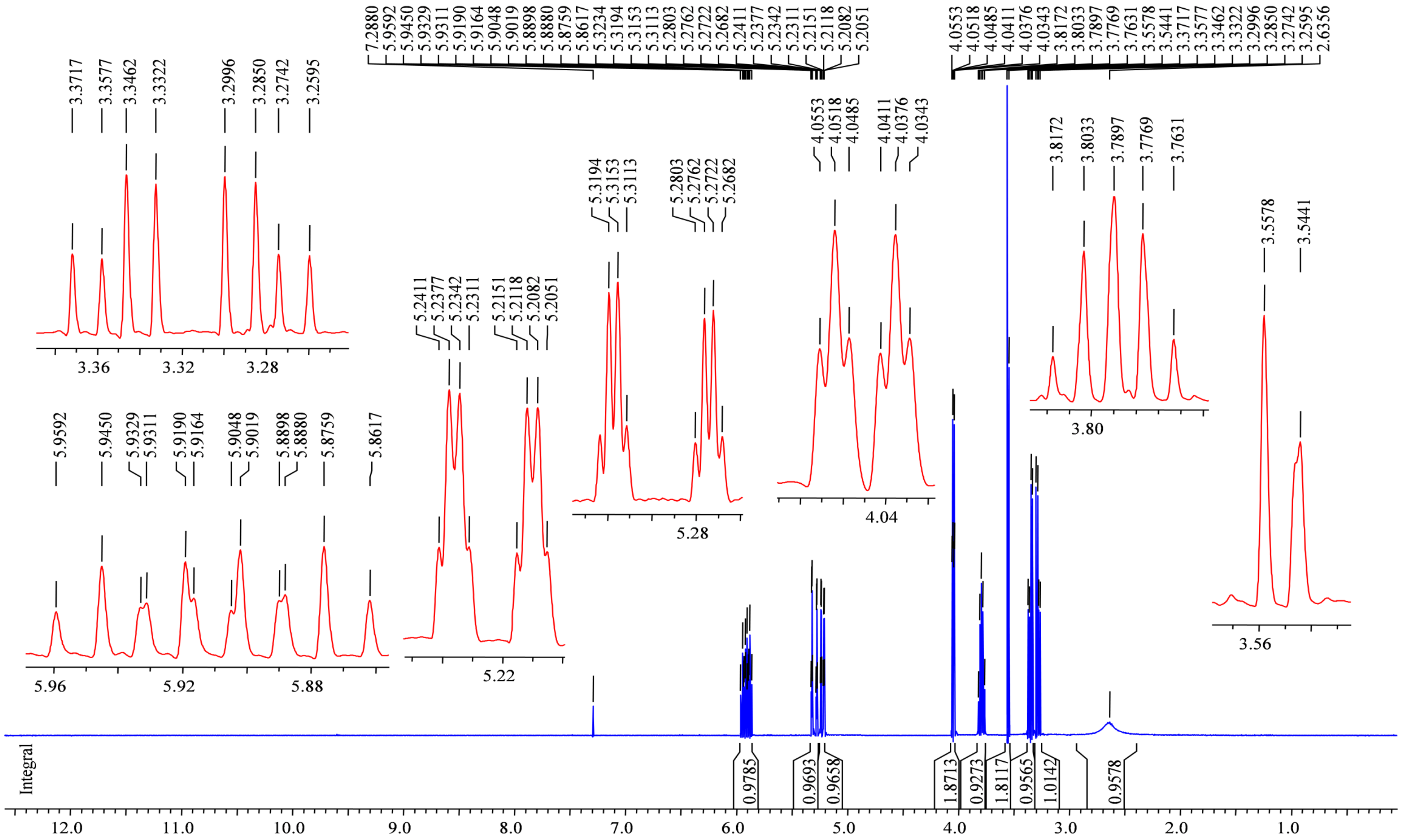

Figure 14. ^{1}H NMR spectrum (400.0 MHz, $CDCl_3$) of 1-(allyloxy)-3-iodopropan-2-ol (**6c**).

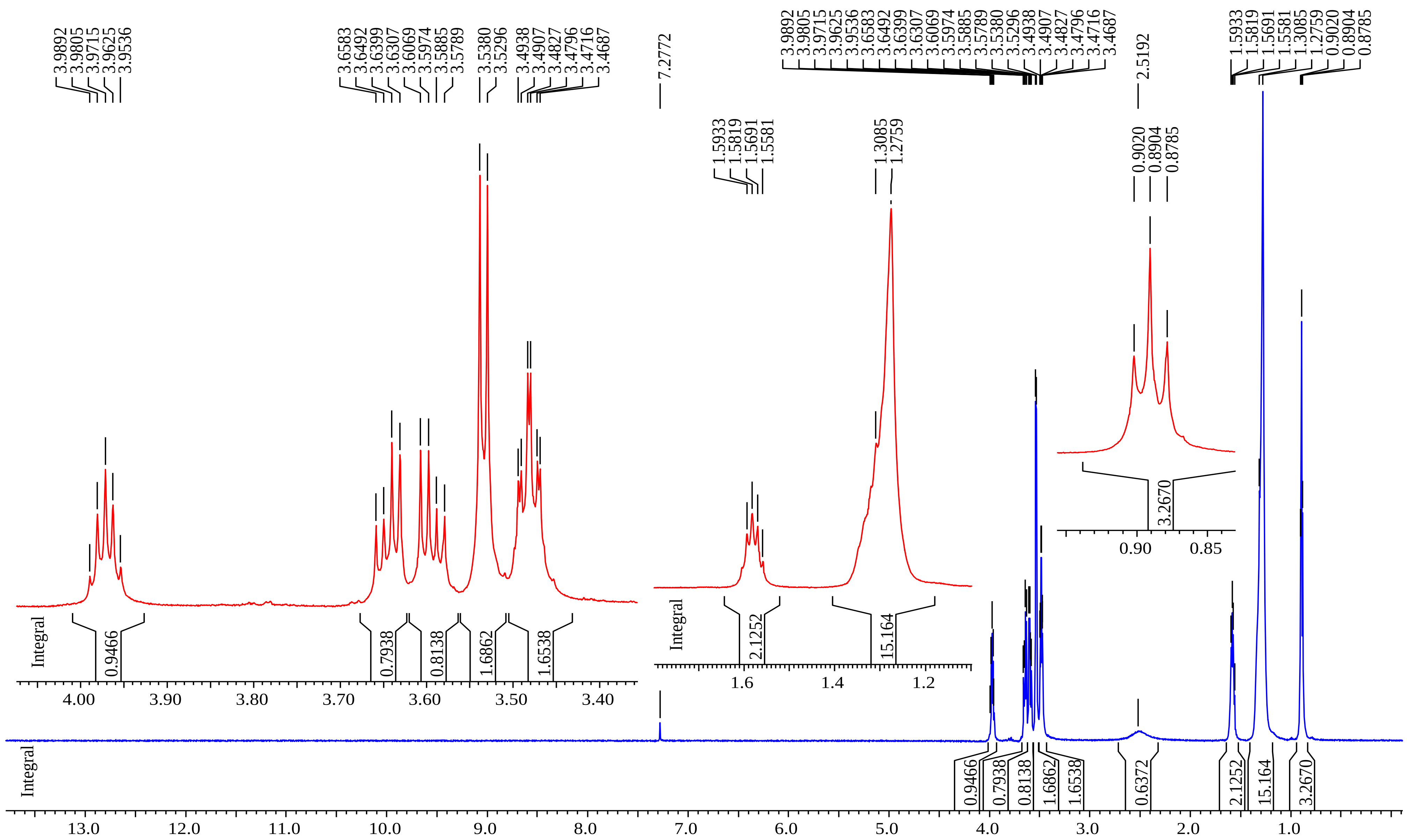

Figure 15. ^{1}H NMR spectrum (400.0 MHz, $CDCl_3$) of 1-chloro-3-(decyloxy)propan-2-ol (**5d**).

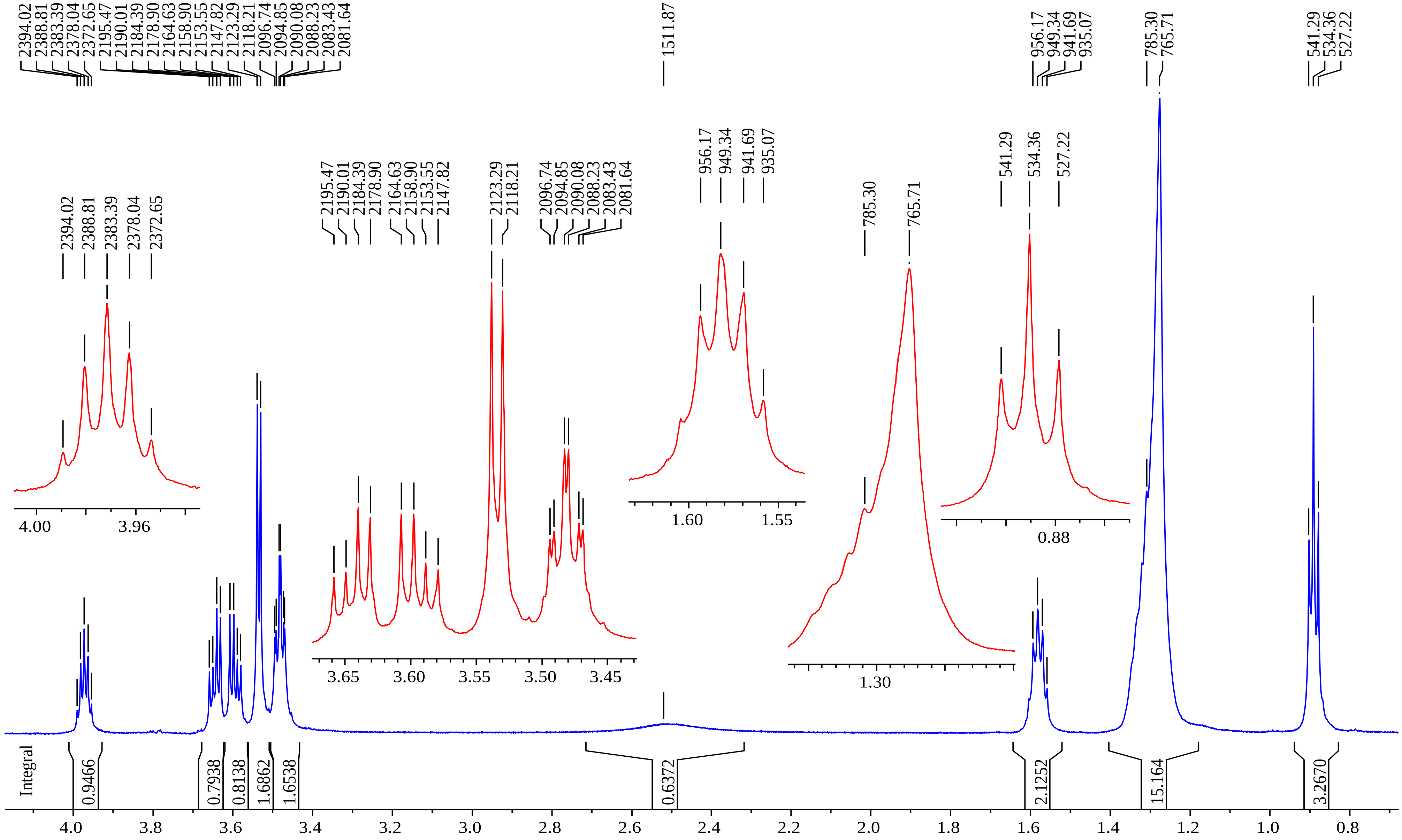

Figure 16. Fragment of ^{1}H NMR spectrum (400.0 MHz, $CDCl_3$) of 1-chloro-3-(decyloxy)propan-2-ol (**5d**).

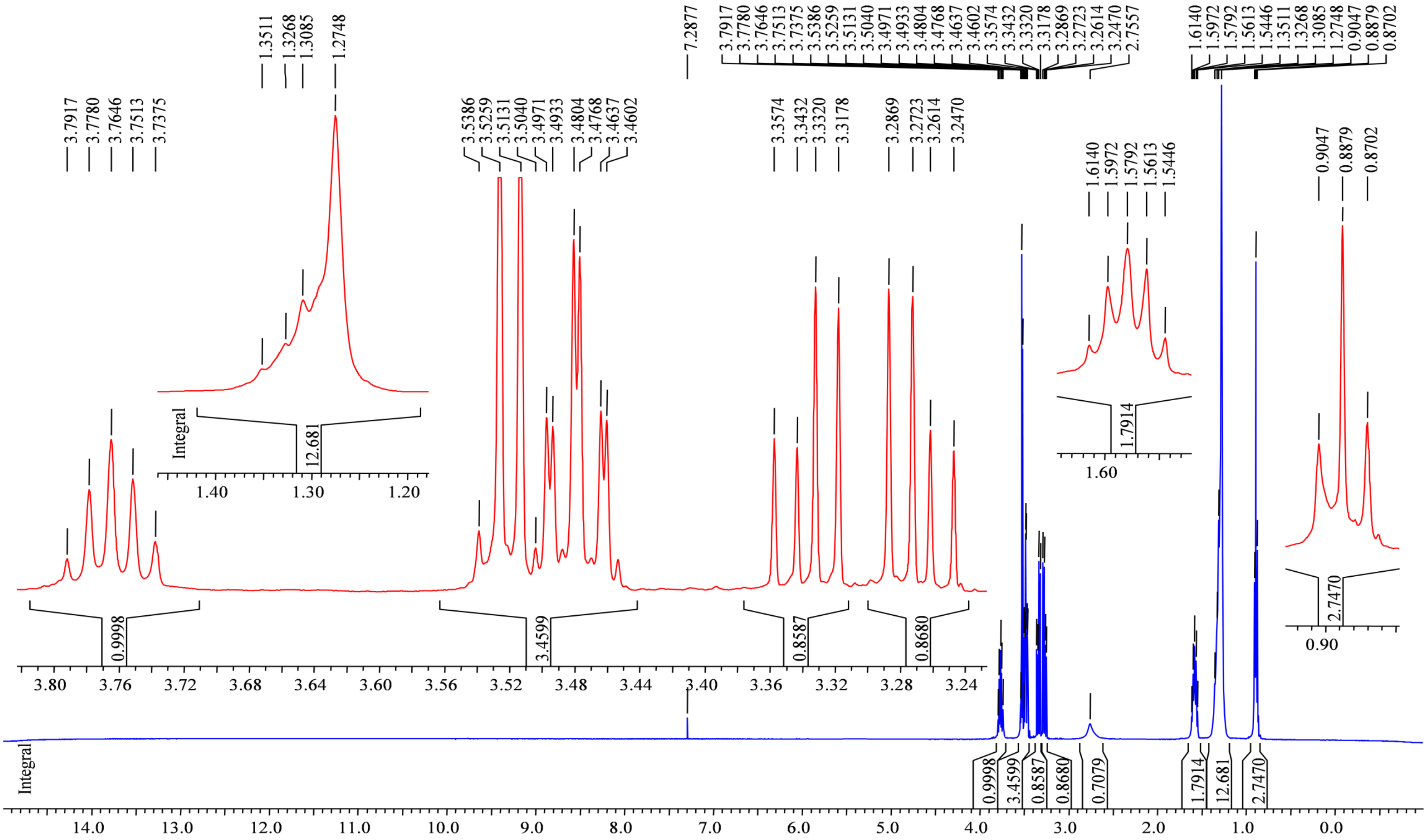

Figure 17. ^{1}H NMR spectrum (400.0 MHz, $CDCl_3$) of 1-(decyloxy)-3-iodopropan-2-ol (**6d**).

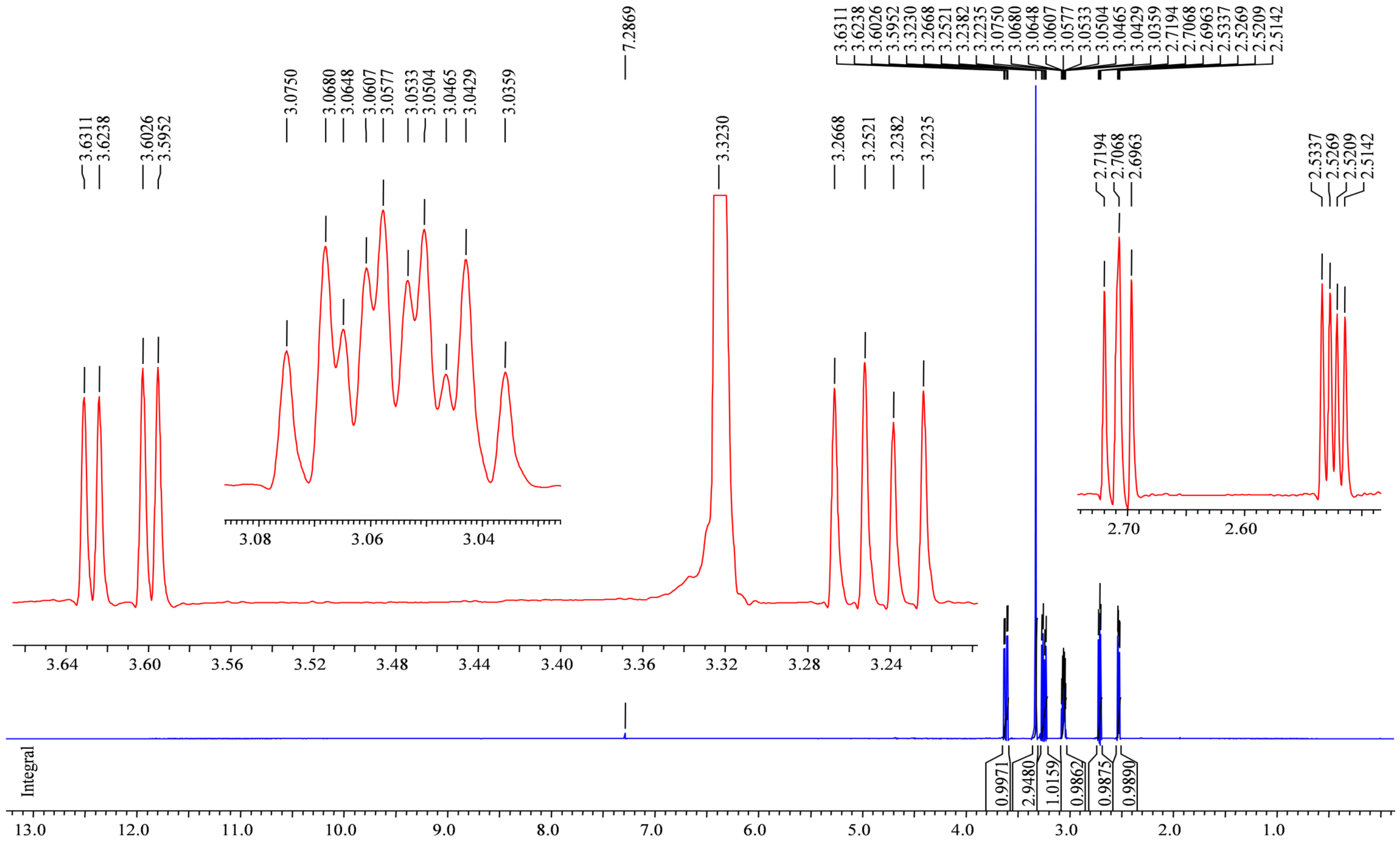

Figure 18. ^{1}H NMR spectrum (400.0 MHz, $CDCl_3$) of 2-(methoxymethyl)oxirane (**2f**).

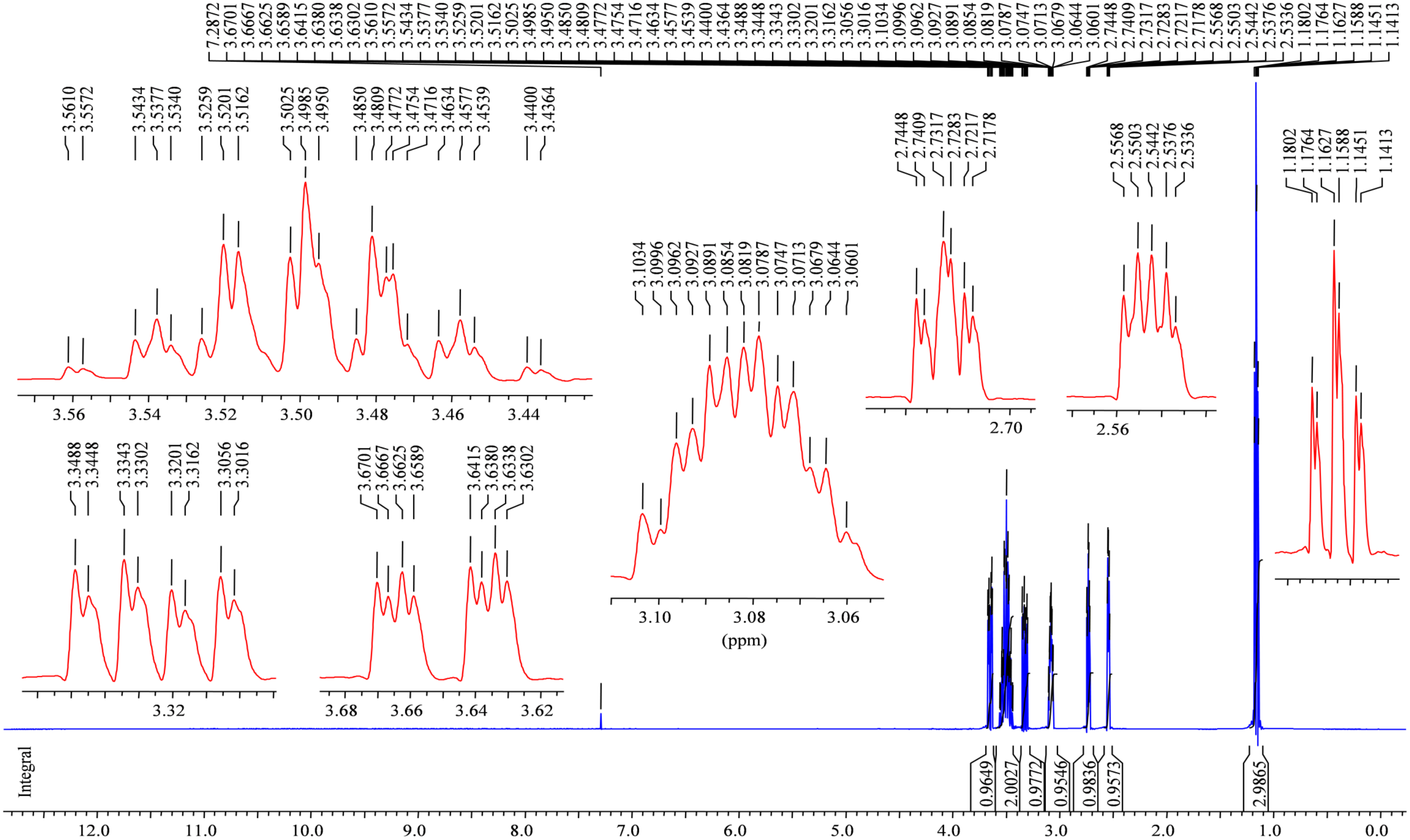

Figure 19. ^{1}H NMR spectrum (400.0 MHz, $CDCl_3$) of 2-(ethoxymethyl)oxirane (**2g**).

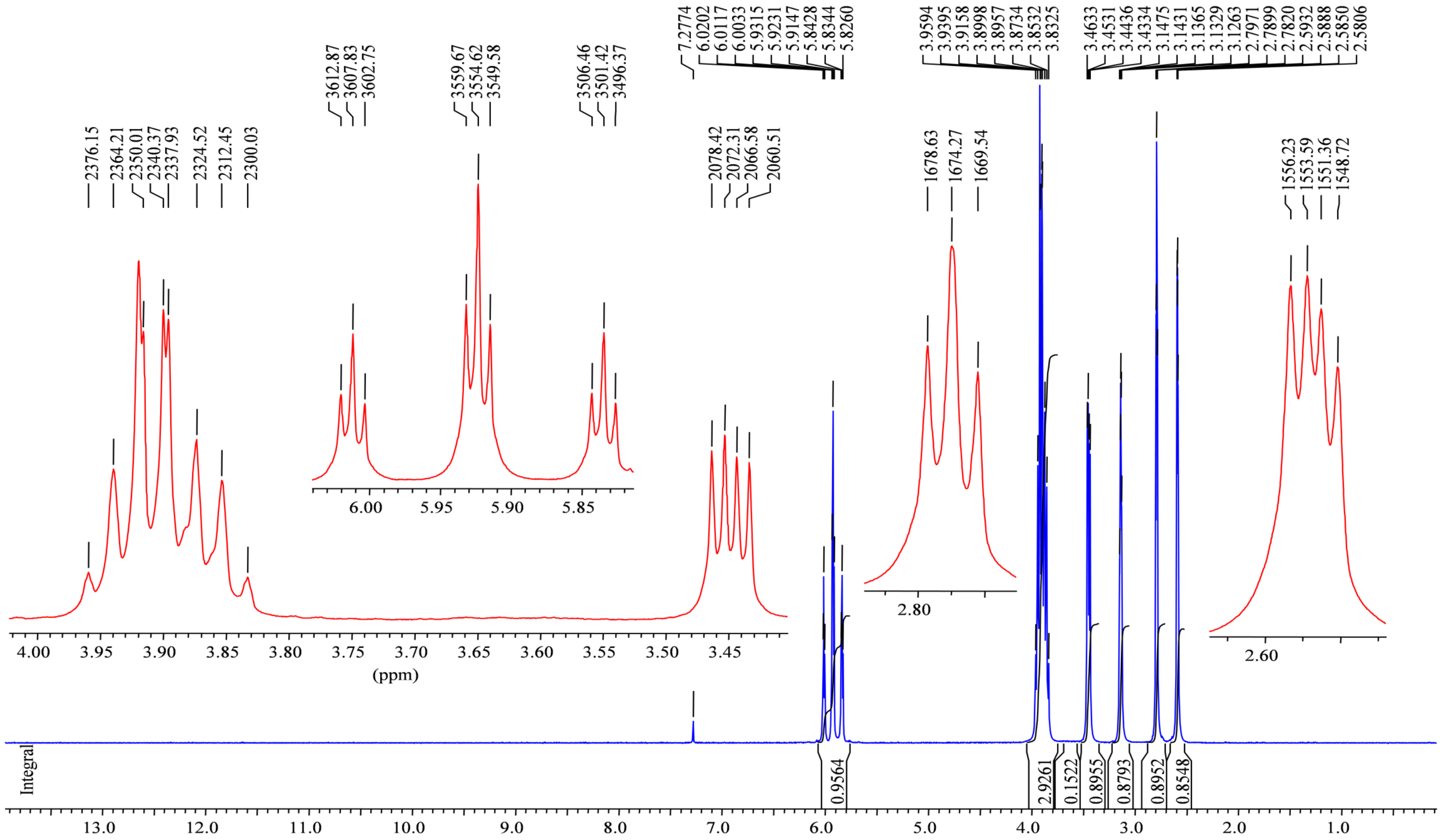

Figure 20. ^{1}H NMR spectrum (400.0 MHz, $CDCl_3$) of 2-((2,2,3,3-tetrafluoropropoxy)methyl)oxirane (**2h**).

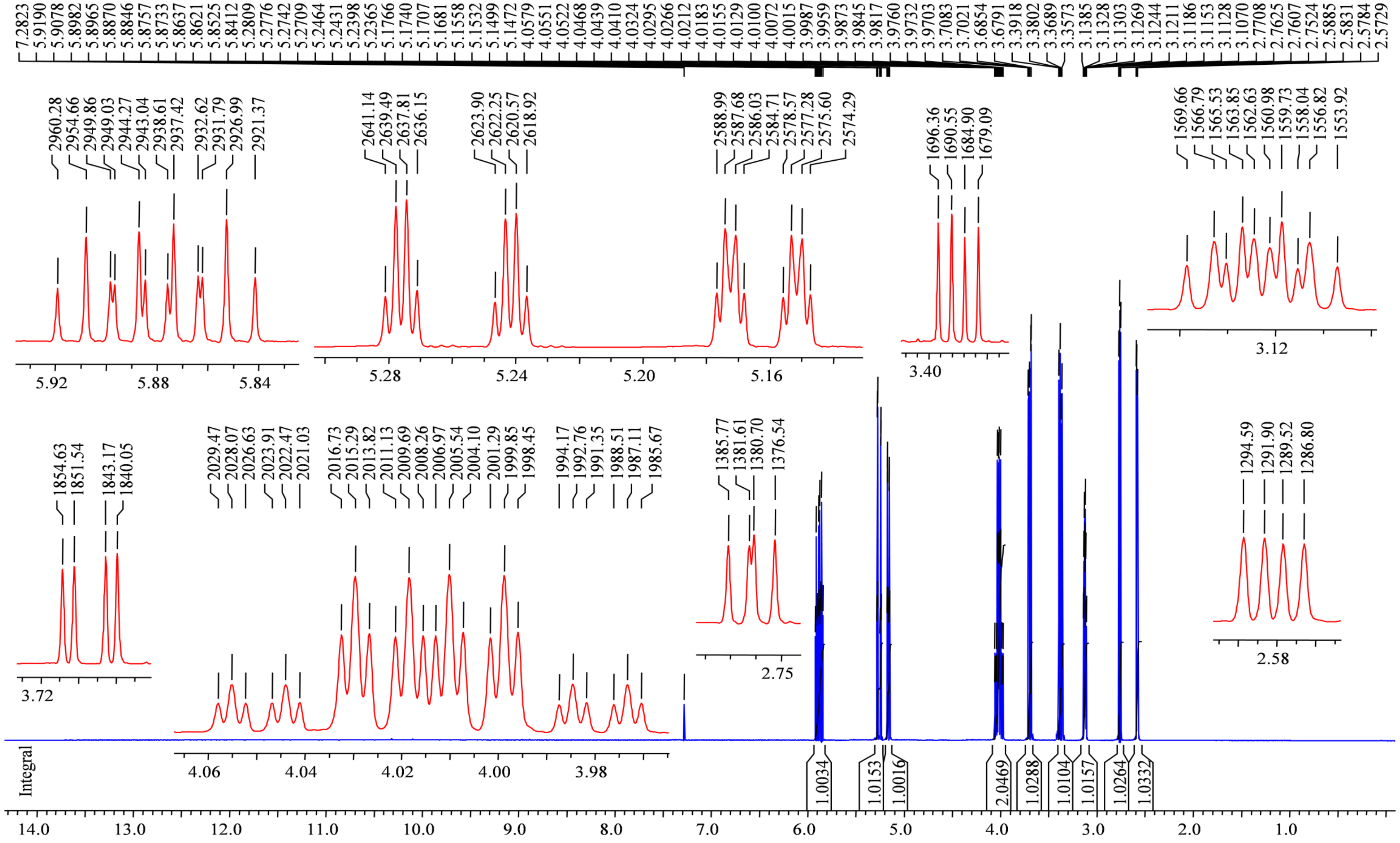

Figure 21. ^{1}H NMR spectrum (400.0 MHz, $CDCl_3$) of 2-((allyloxy)methyl)oxirane (**2i**).

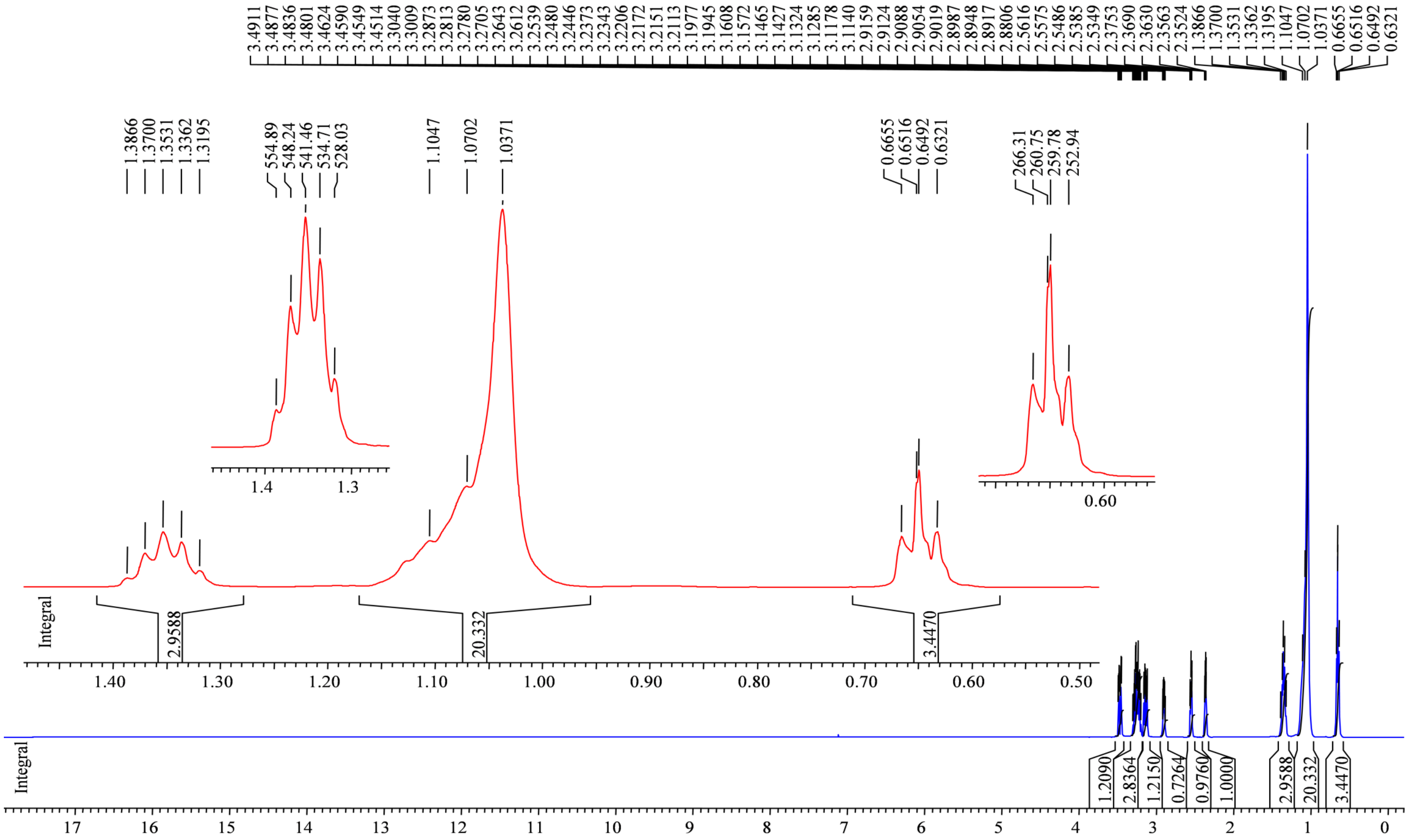

Figure 22. ^{1}H NMR spectrum (400.0 MHz, $CDCl_3$) of 2-((decyloxy)methyl)oxirane (**2j**).

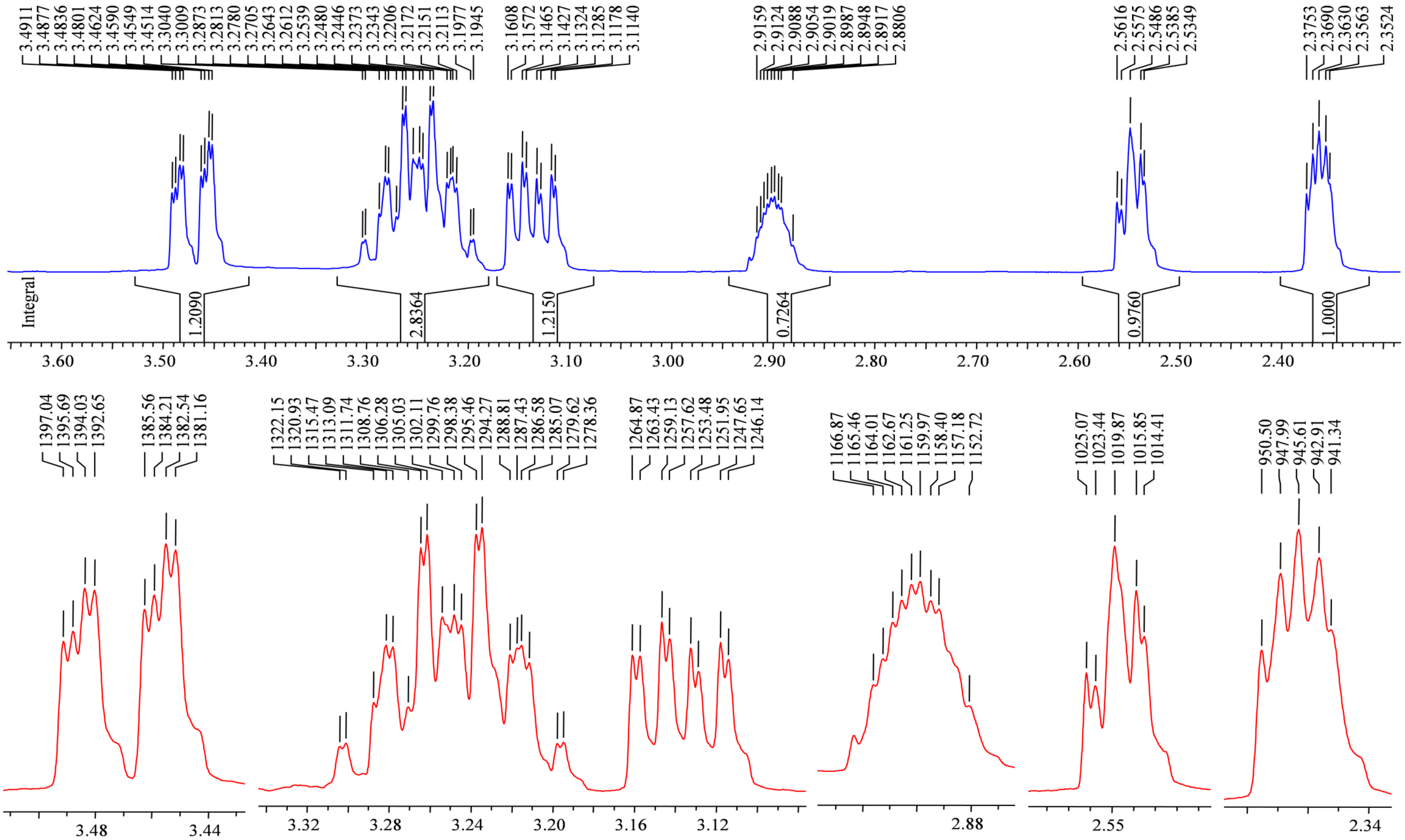

Figure 23. Fragments of ^{1}H NMR spectrum (400.0 MHz, $CDCl_3$) of 2-((decyloxy)methyl)oxirane (**2j**).

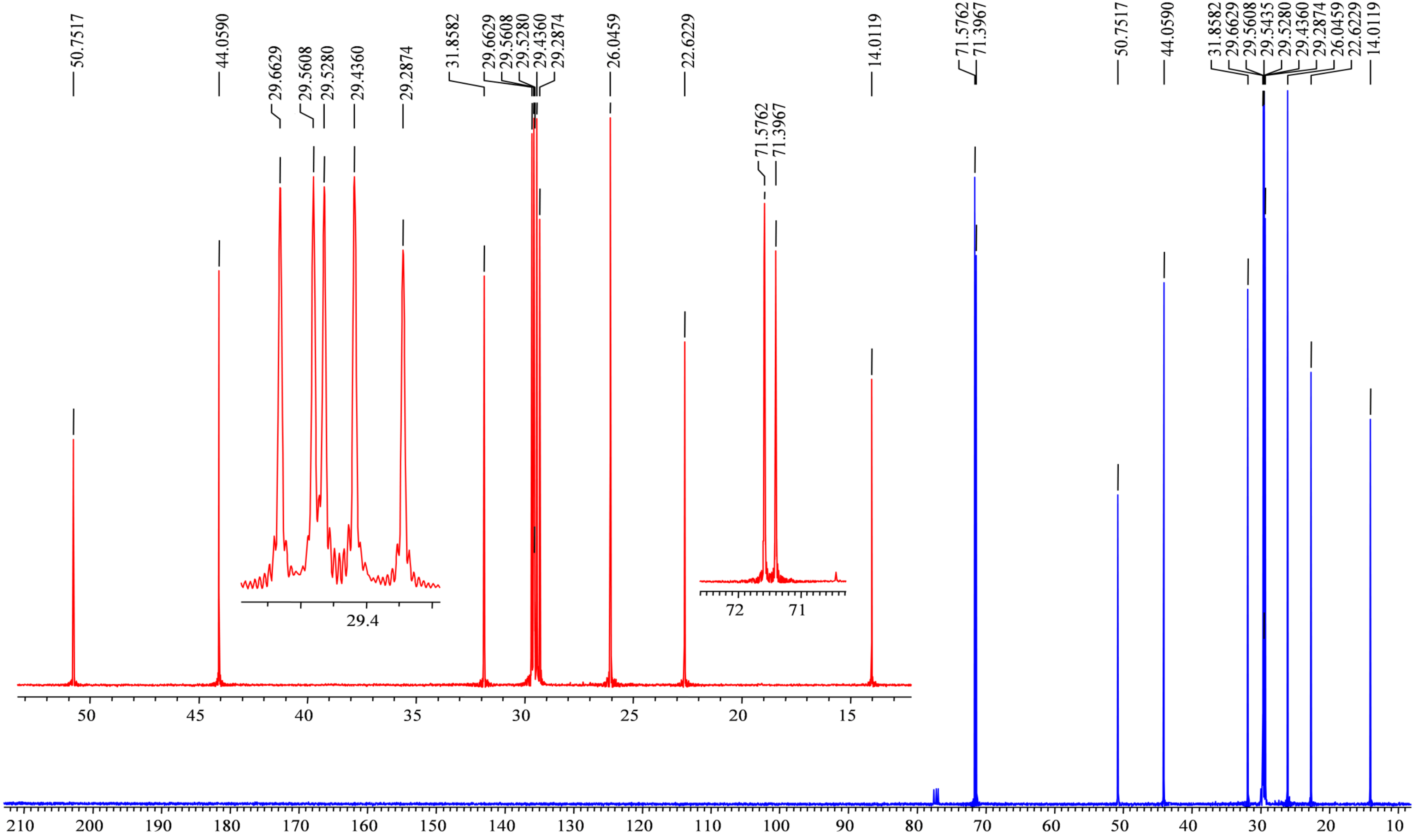

Figure 24. ^{13}C-{^{1}H} NMR spectrum (100.6 MHz, $CDCl_3$) of 2-((decyloxy)methyl)oxirane (**2j**).

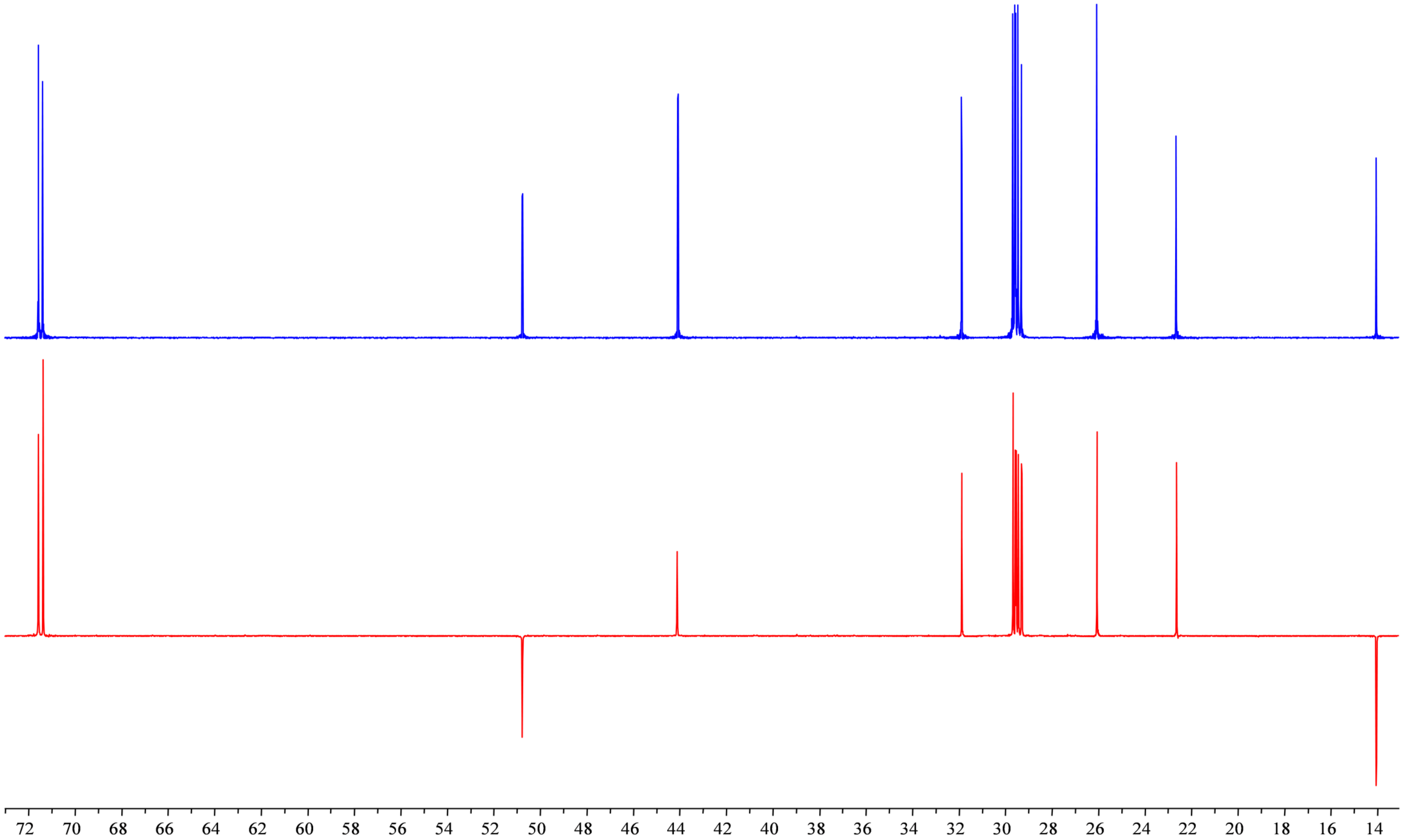

Figure 25. ^{13}C-{^{1}H} and ^{13}C-{^{1}H}-dept NMR spectra (100.6 MHz, $CDCl_3$) of 2-((decyloxy)methyl)oxirane (**2j**).

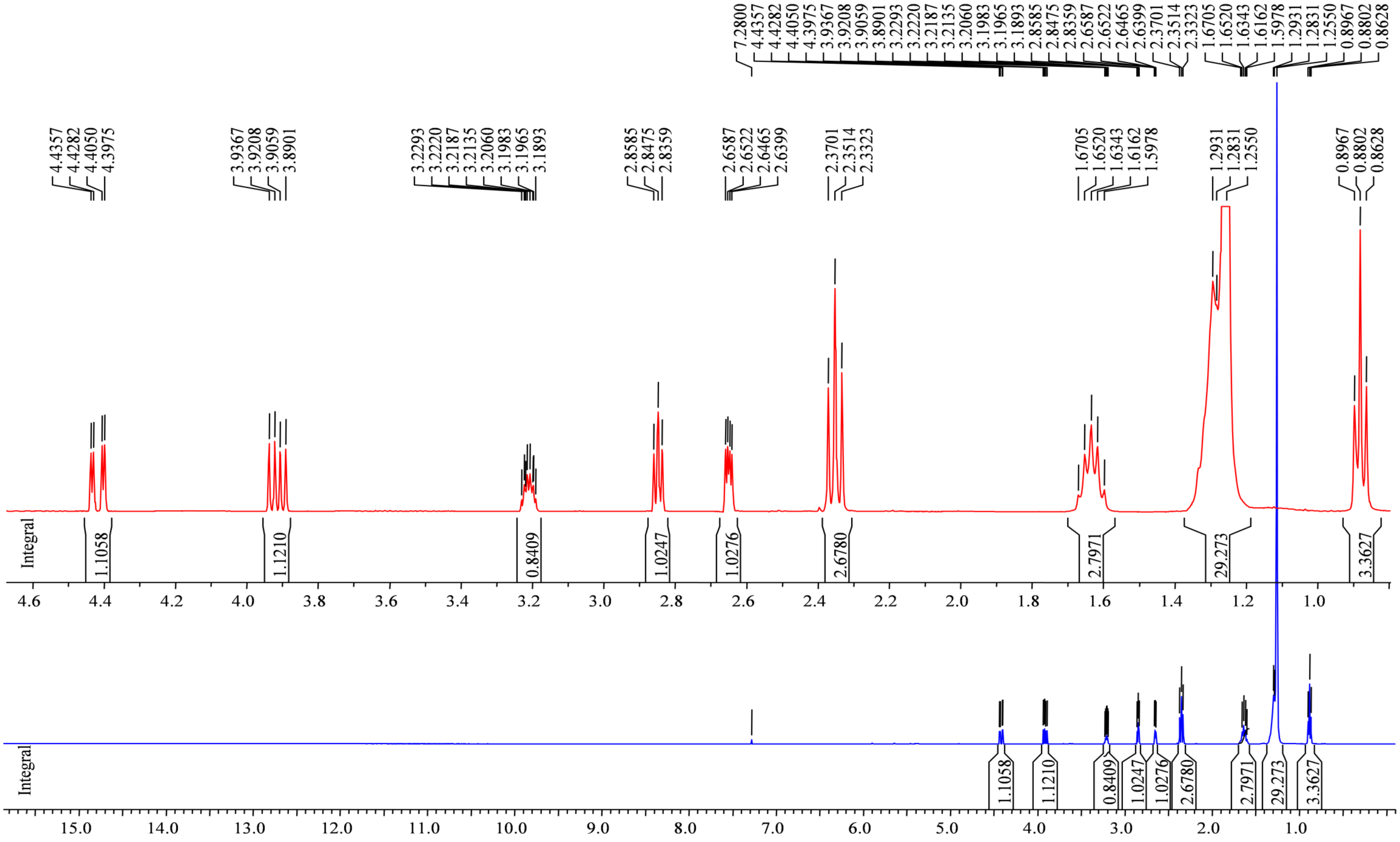

7.2800
4.4357
4.4282
4.4050
4.3975
3.9367
3.9208
3.9059
3.8901
3.2293
3.2220
3.2187
3.2135
3.2060
3.1983
3.1965
3.1893
2.8585
2.8475
2.8359
2.6587
2.6522
2.6465
2.6399
2.3701
2.3514
2.3323
1.6705
1.6520
1.6343
1.6162
1.5978
1.2931
1.2831
1.2550
0.8967
0.8802
0.8628
Integral
1.1058
1.1210
0.8409
1.0247
1.0276
2.6780
2.7971
29.273
3.3627

Figure 26. ^{1}H NMR spectrum (400 MHz, $CDCl_3$) of oxiran-2-ylmethyl stearate (**2l**).

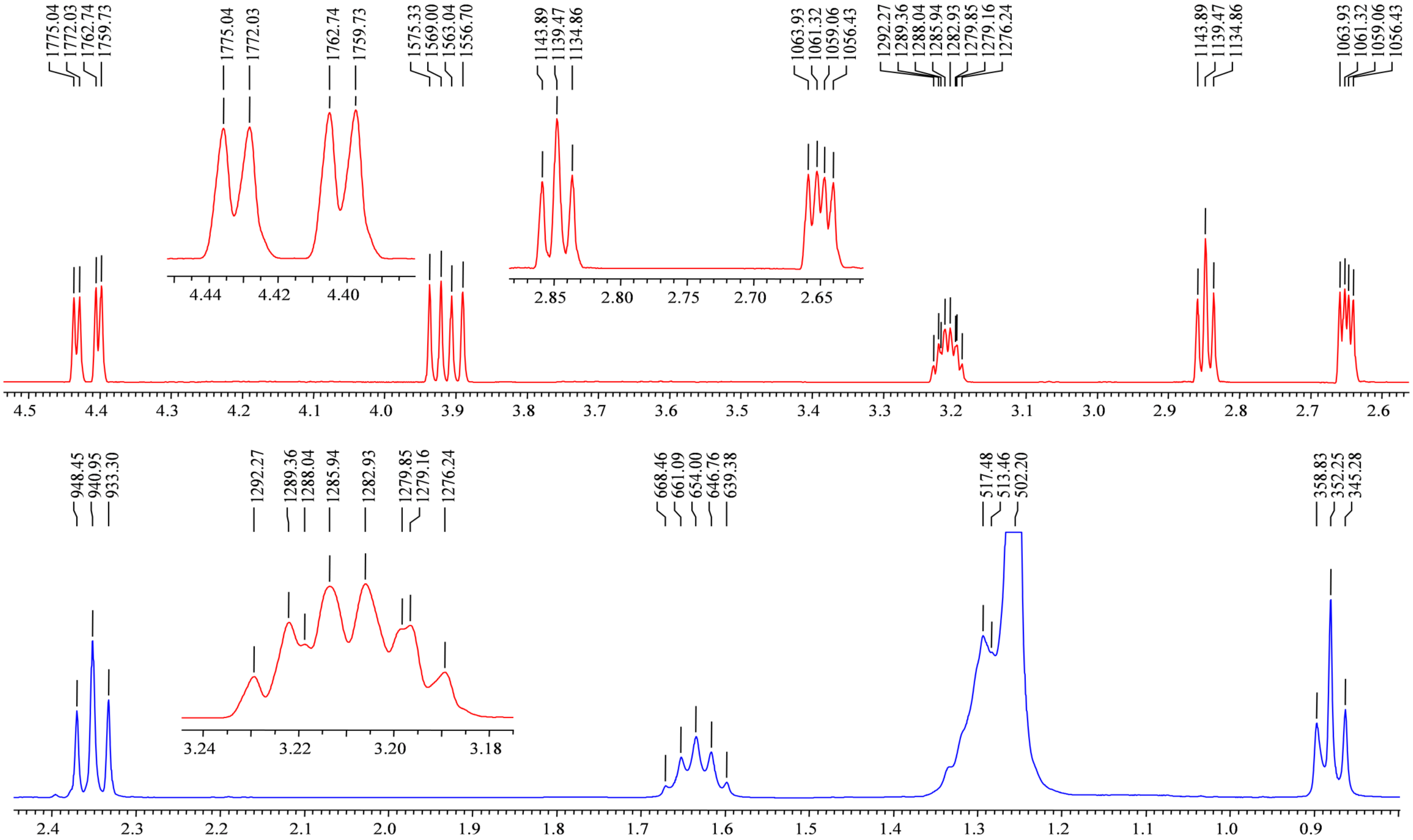

Figure 27. Fragments of ^{1}H NMR spectrum (100.6 MHz, $CDCl_3$) of oxiran-2-ylmethyl stearate (**2l**).

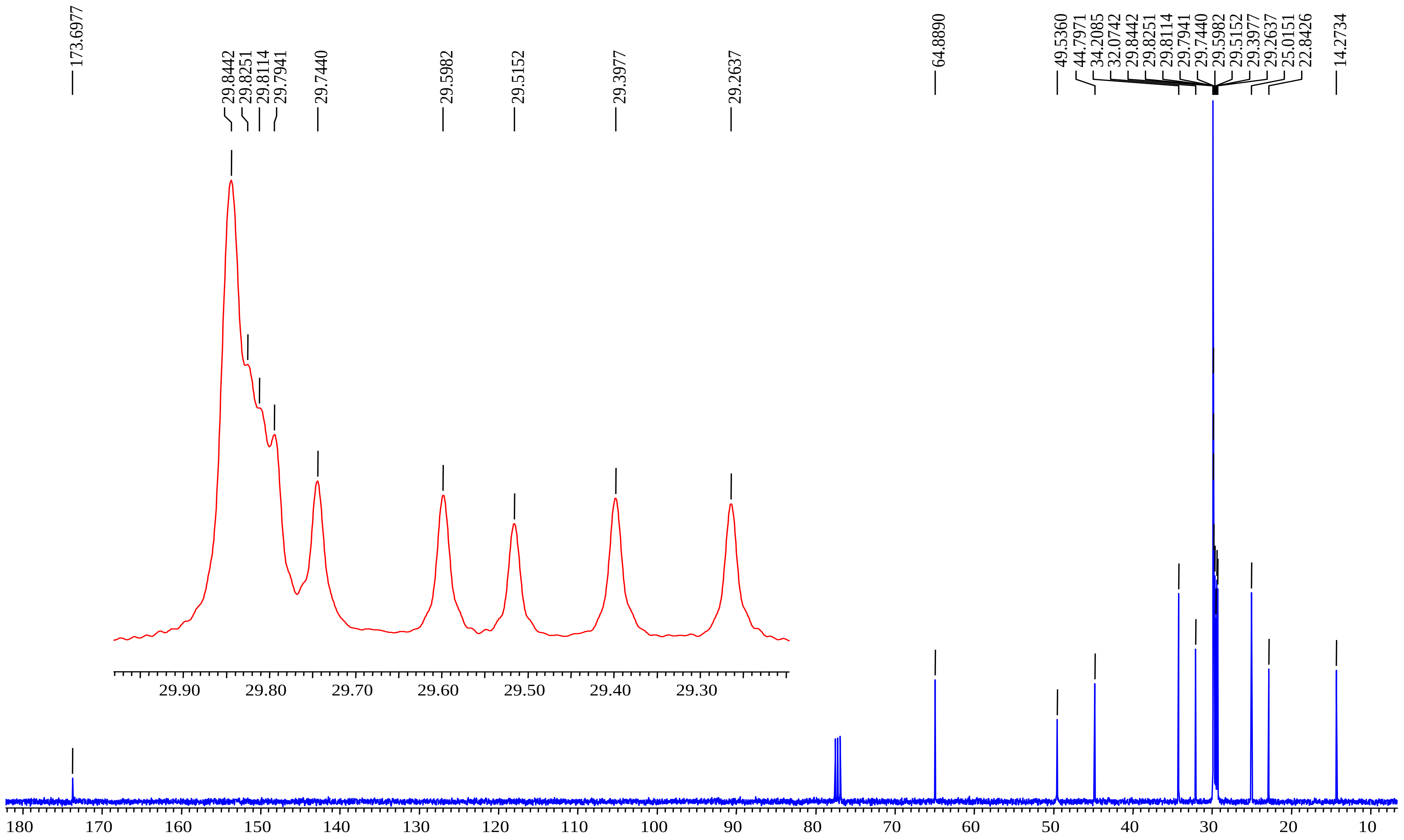

Figure 28. ^{13}C-{^{1}H} NMR spectrum (100.6 MHz, $CDCl_3$) of oxiran-2-ylmethyl stearate (**2l**).

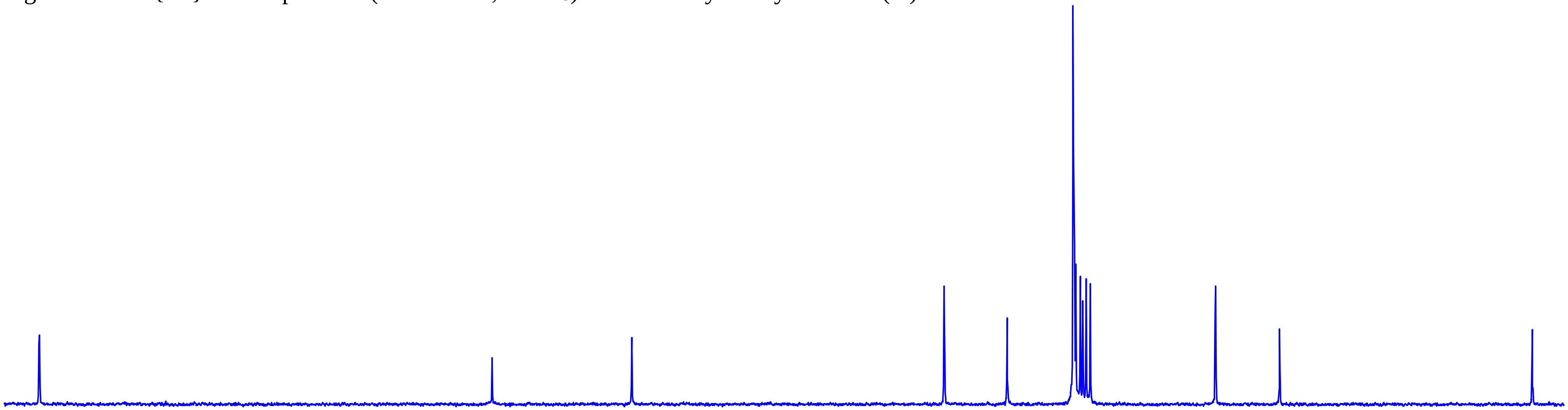

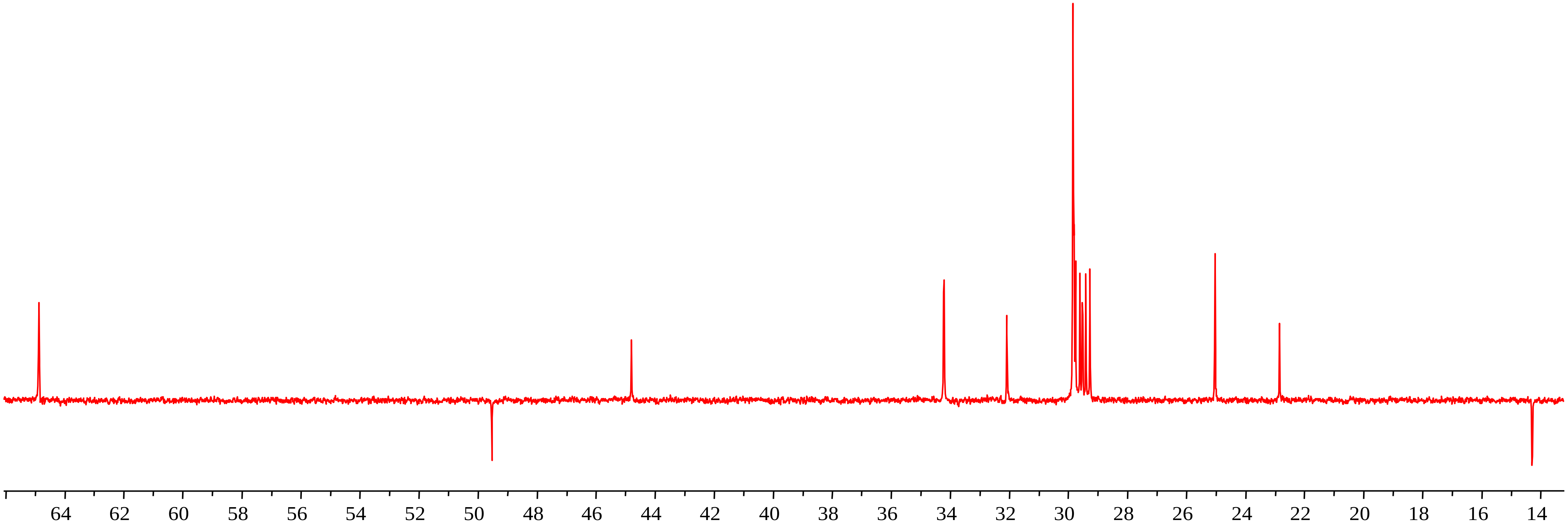

Figure 29. ^{13}C-{^{1}H} and ^{13}C-{^{1}H}-APT NMR spectra (100.6 MHz, $CDCl_3$) of oxiran-2-ylmethyl stearate (**2l**).

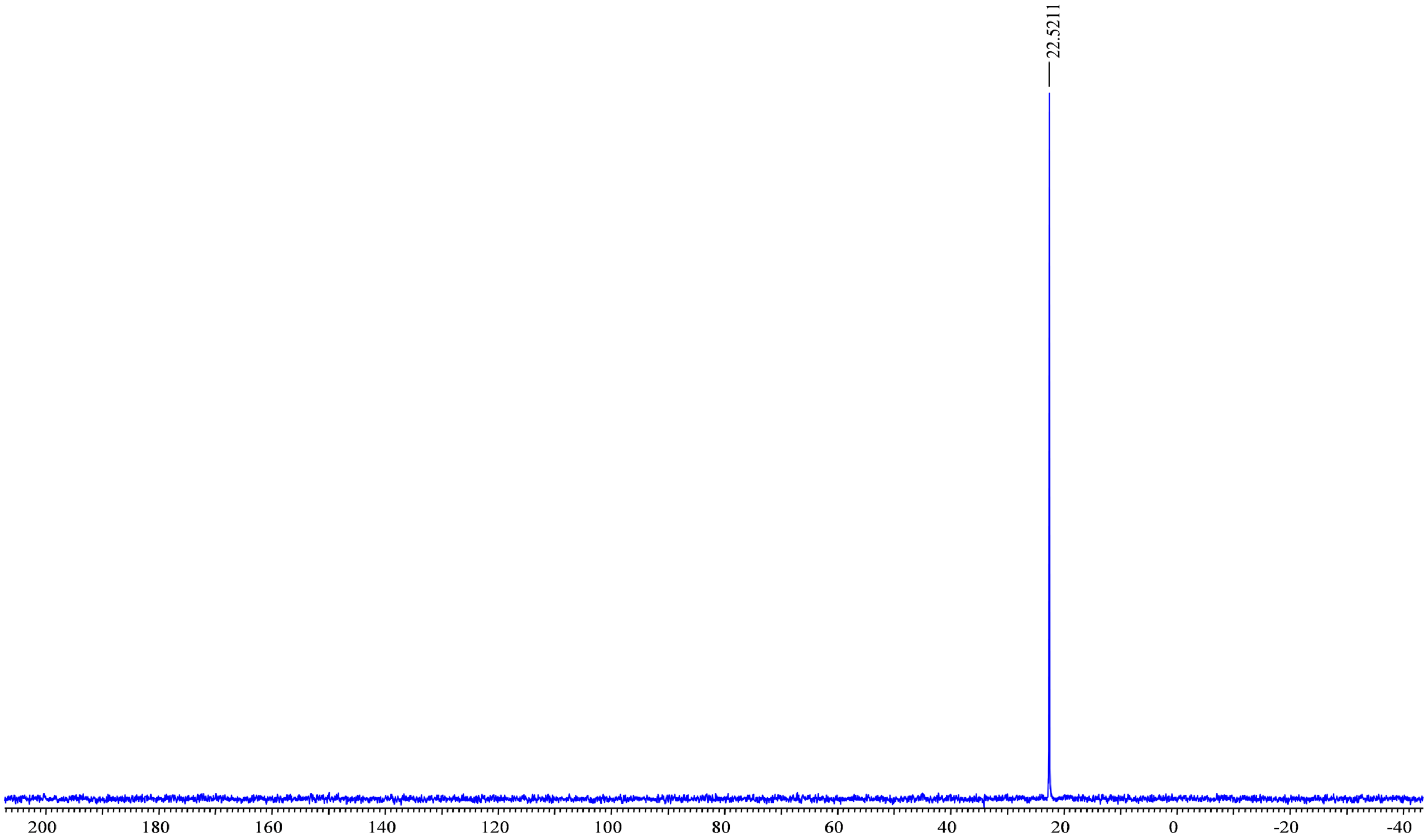

Figure 30. ^{31}P-{^{1}H} NMR spectrum (162.0 MHz, CH_2Cl_2) of compound (**3a**).

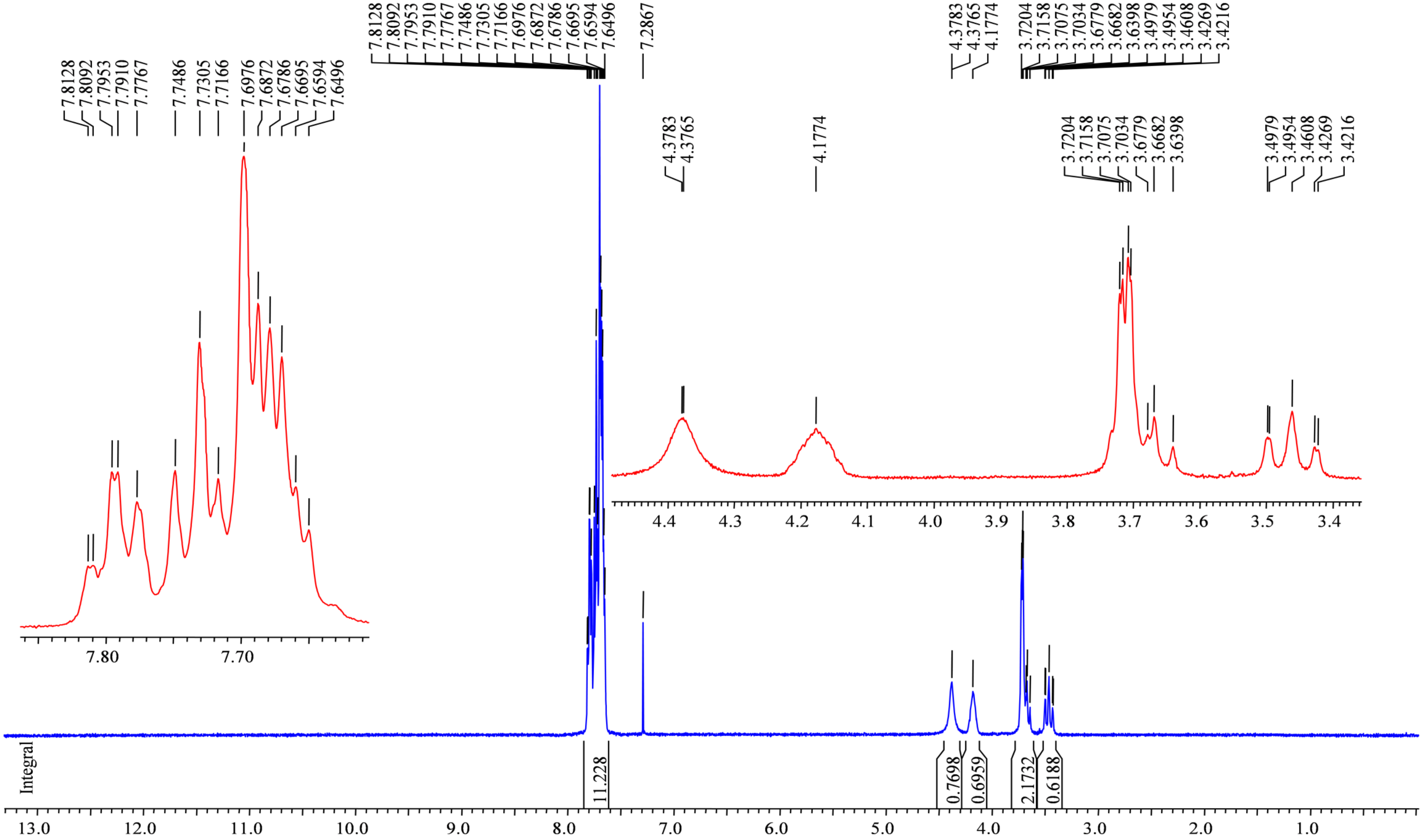

Figure 31. ^{1}H NMR spectrum (400 MHz, $CDCl_3$) of compound (**3e**) (*R*).

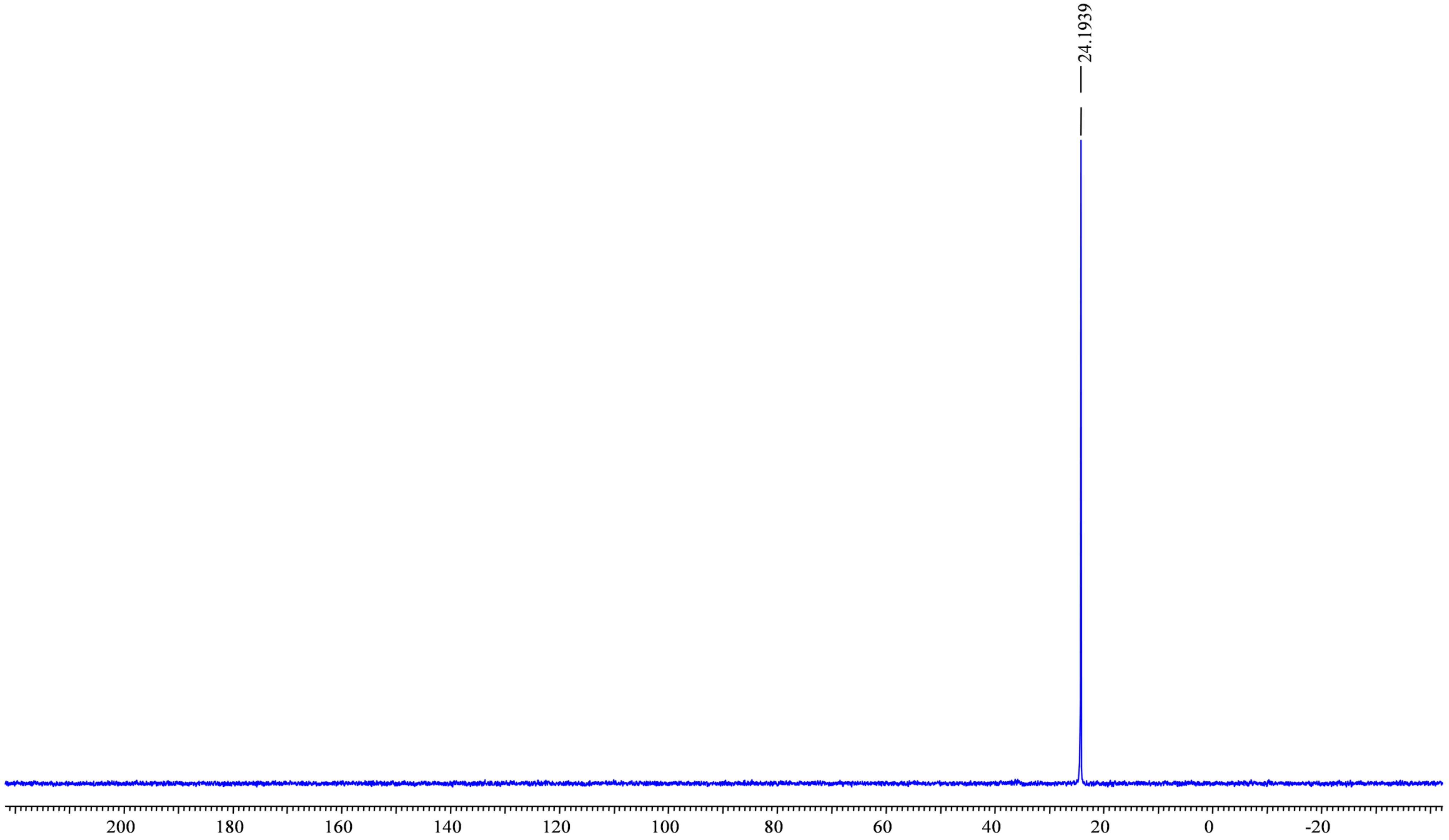

Figure 32. ^{31}P-{^{1}H} NMR spectrum (162.0 MHz, $CDCl_3$) of compound (**3e**) (*R*).

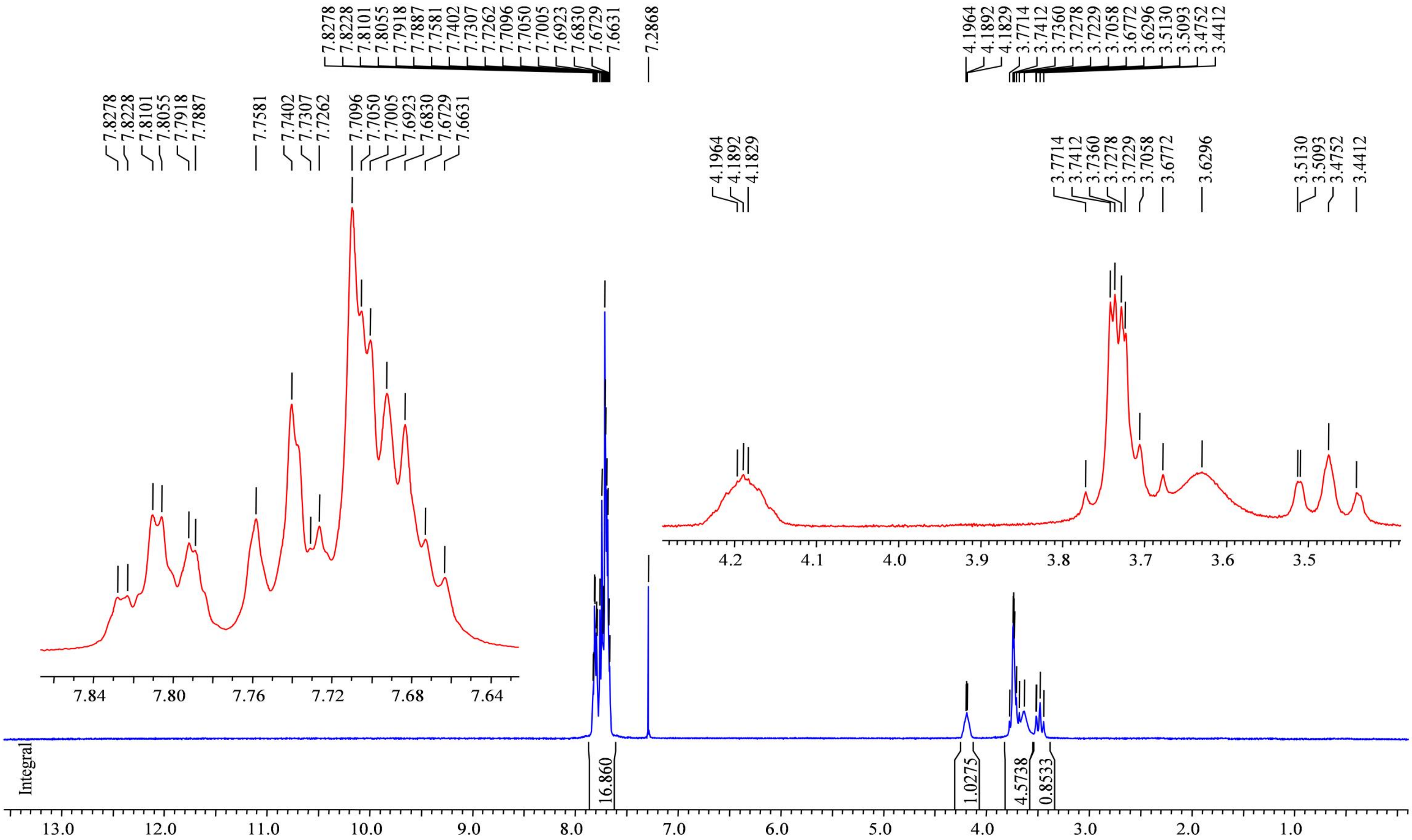

Figure 33. ^{1}H NMR spectrum (400 MHz, $CDCl_3$) of compound (**3e**) (*S*).

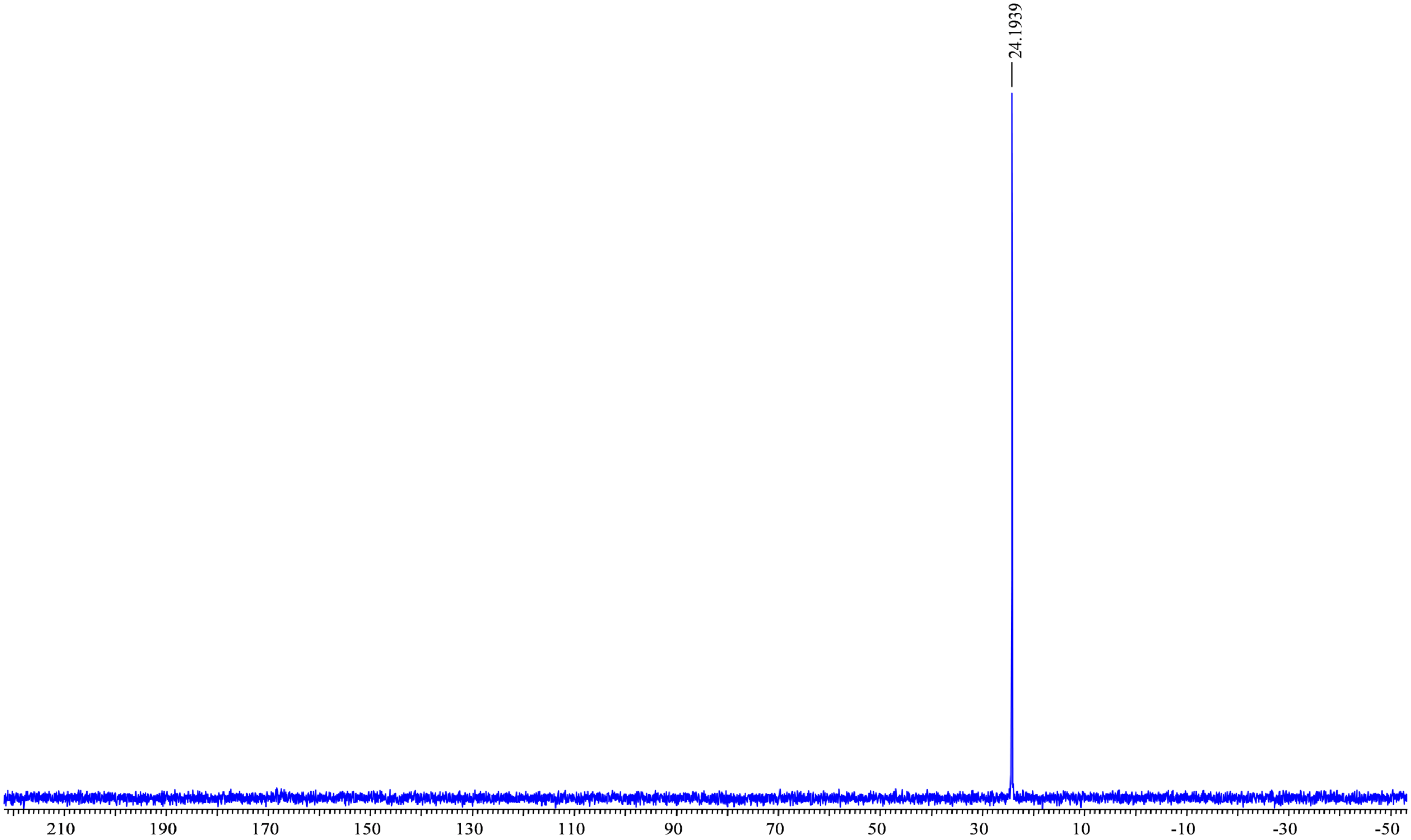

Figure 34. ^{31}P-{^{1}H} NMR spectrum (162.0 MHz, $CDCl_3$) of compound (**3e**) (*S*).

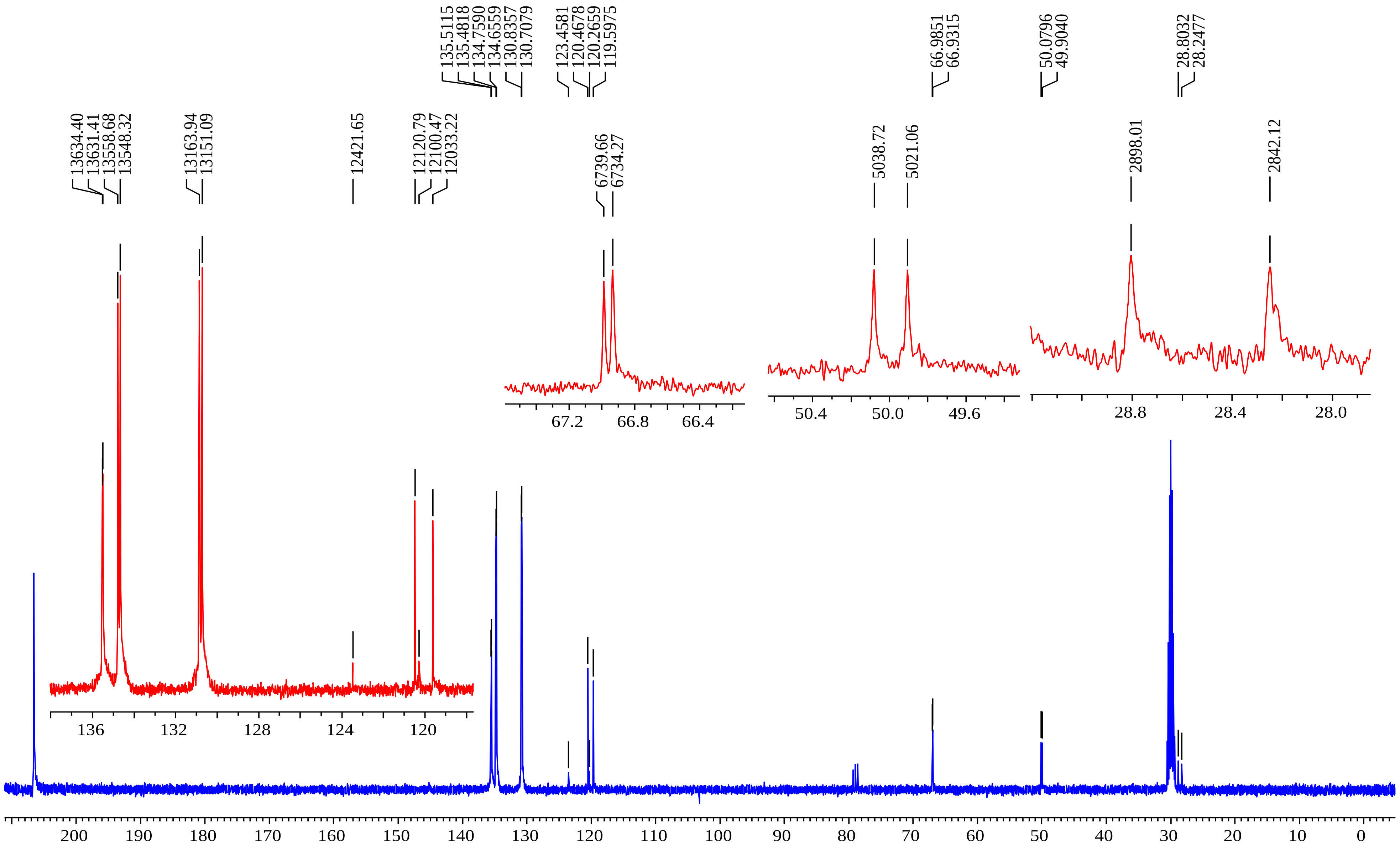

Figure 35. $^{13}C-\{^{1}H\}$ NMR spectrum (100.6 MHz, acetone-d_6 + 10% $CDCl_3$) of compound (**3a**).

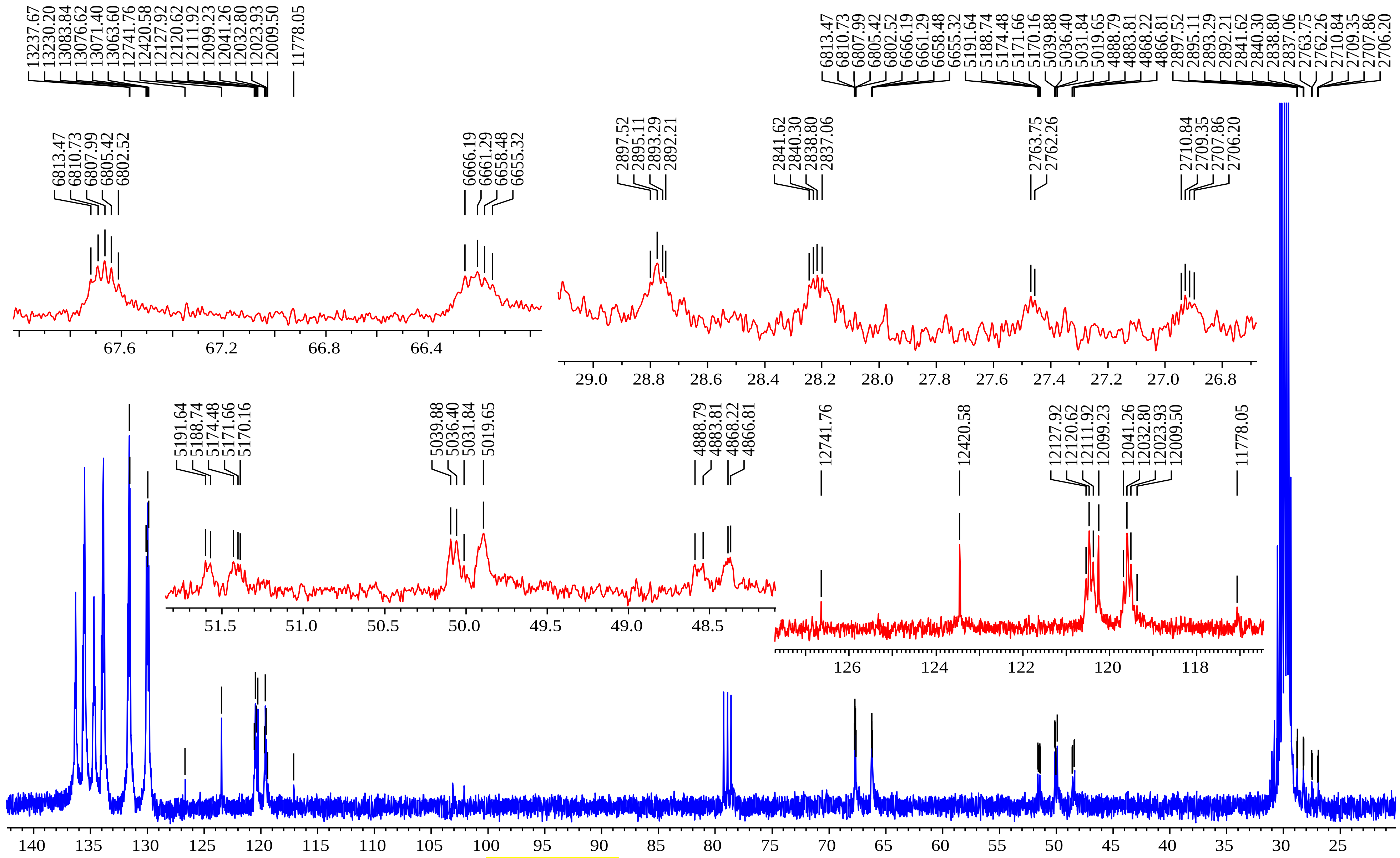

Figure 36. ^{13}C NMR spectrum (100.6 MHz, acetone-d_6 + 10% $CDCl_3$) of compound (**3a**).

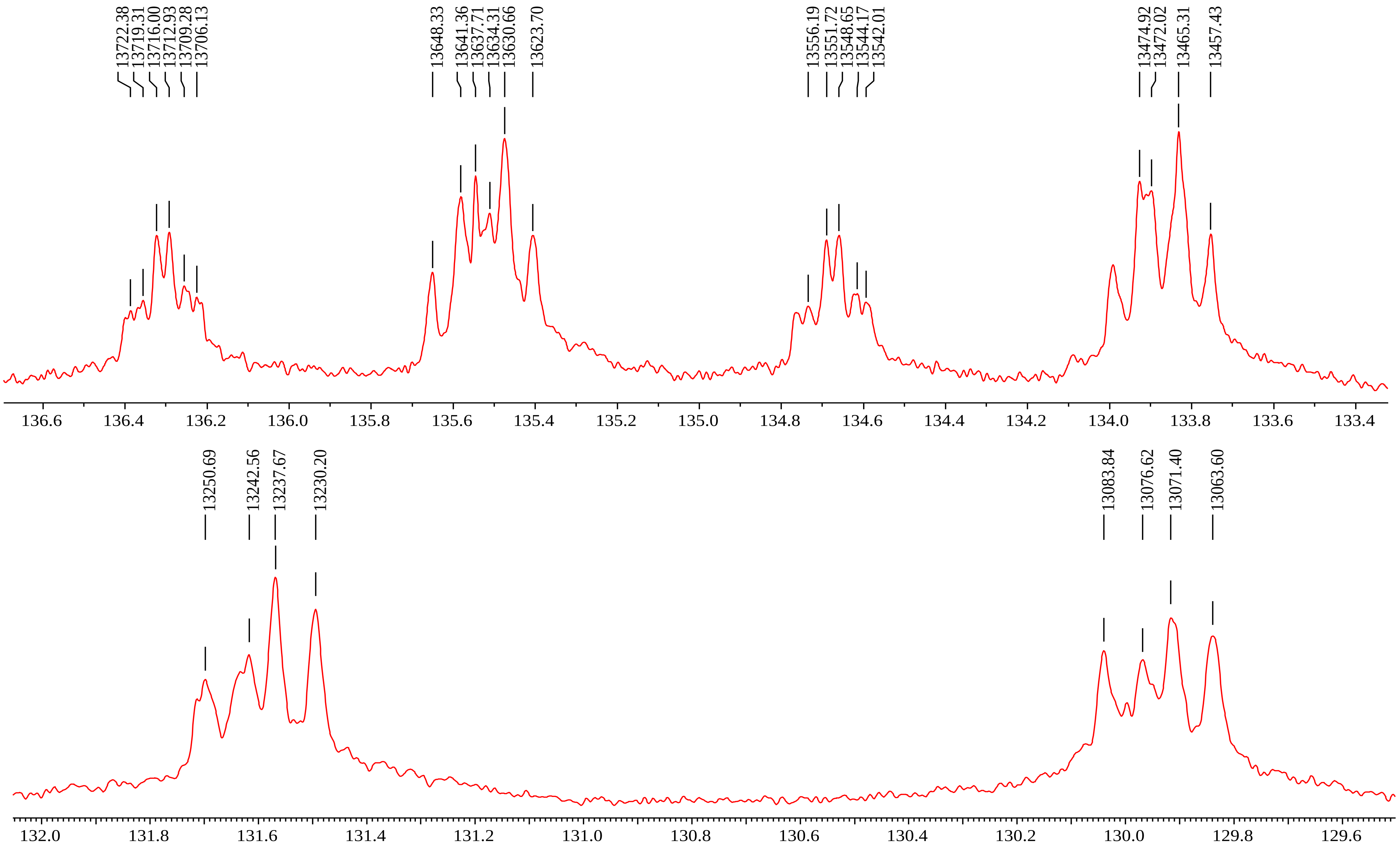

Figure 37. Aromatic carbons region of ^{13}C NMR spectrum (100.6 MHz, acetone-d_6 + 10% $CDCl_3$) of compound (**3a**).

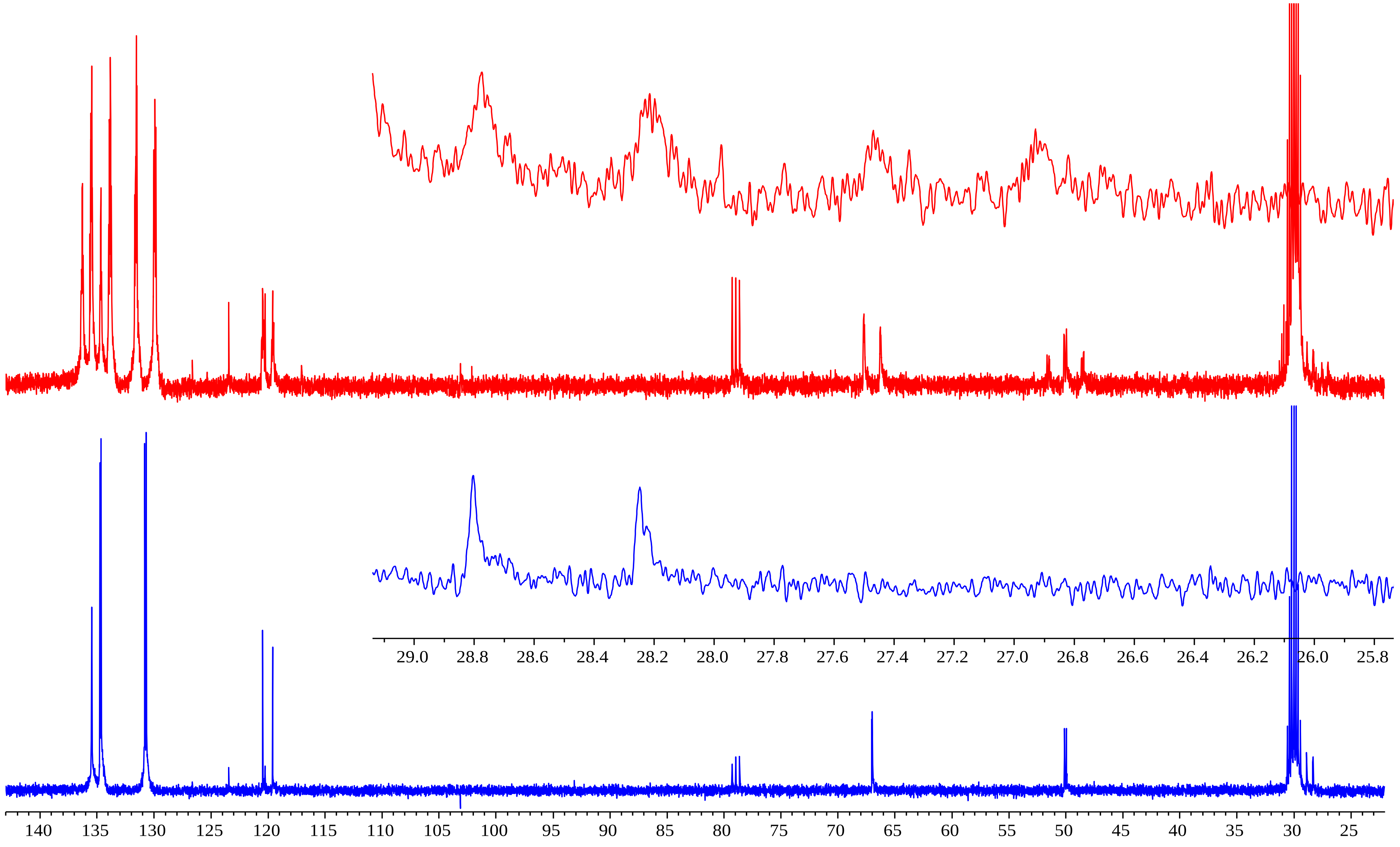

Figure 38. ^{13}C-{^{1}H} and ^{13}C NMR spectra (100.6 MHz, acetone-d_6 + 10% $CDCl_3$) of compound (**3a**).

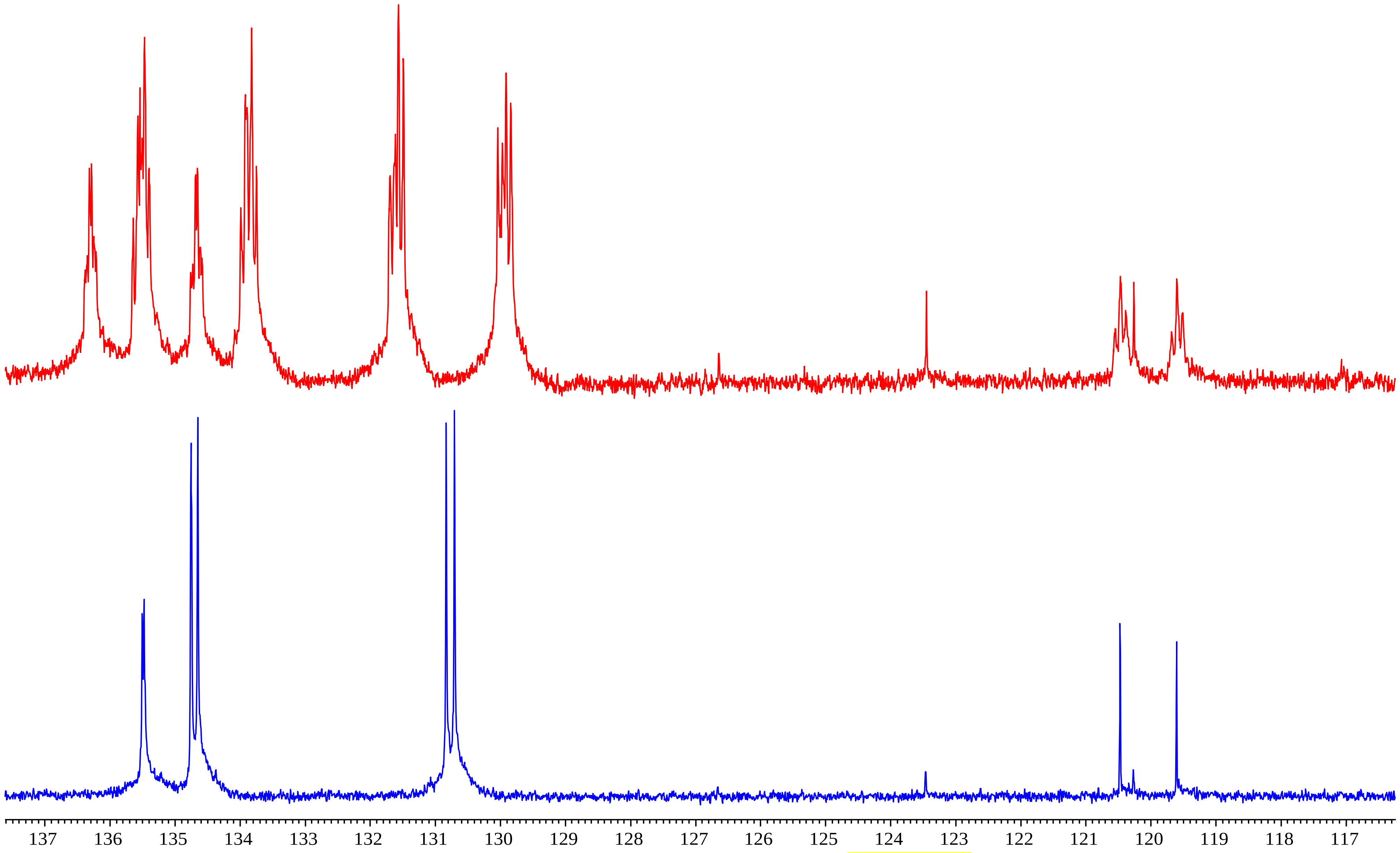

Figure 39. Aromatic carbon region of ^{13}C-{^{1}H} and ^{13}C NMR spectra (100.6 MHz, acetone-d_6 + 10% $CDCl_3$) of compound (**3a**).

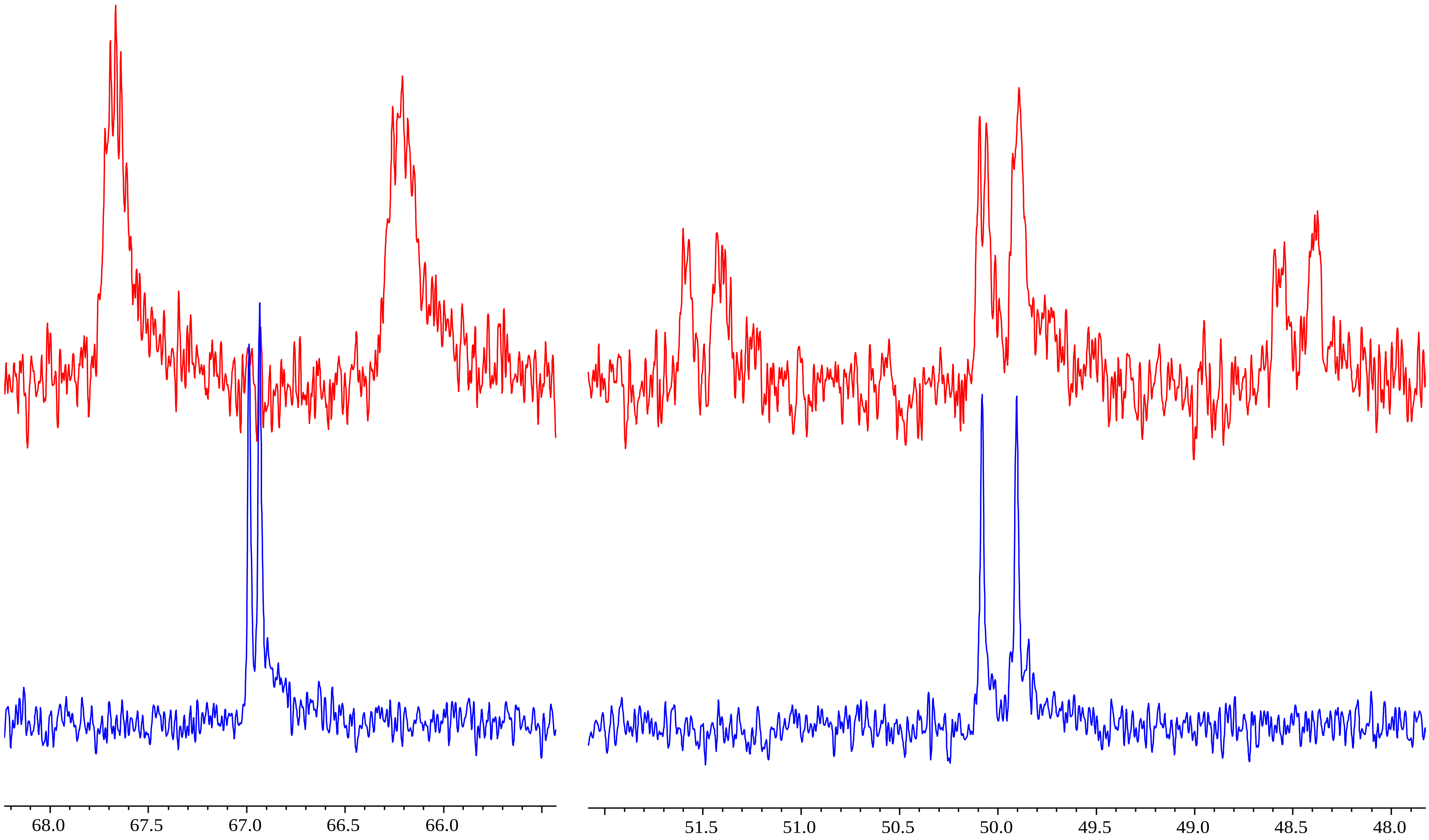

Figure 40. The 66-68 and 48-52 ppm fragments of ^{13}C-{^{1}H} and ^{13}C NMR spectra (100.6 MHz, acetone-d_6 + 10% $CDCl_3$) of compound (**3a**).

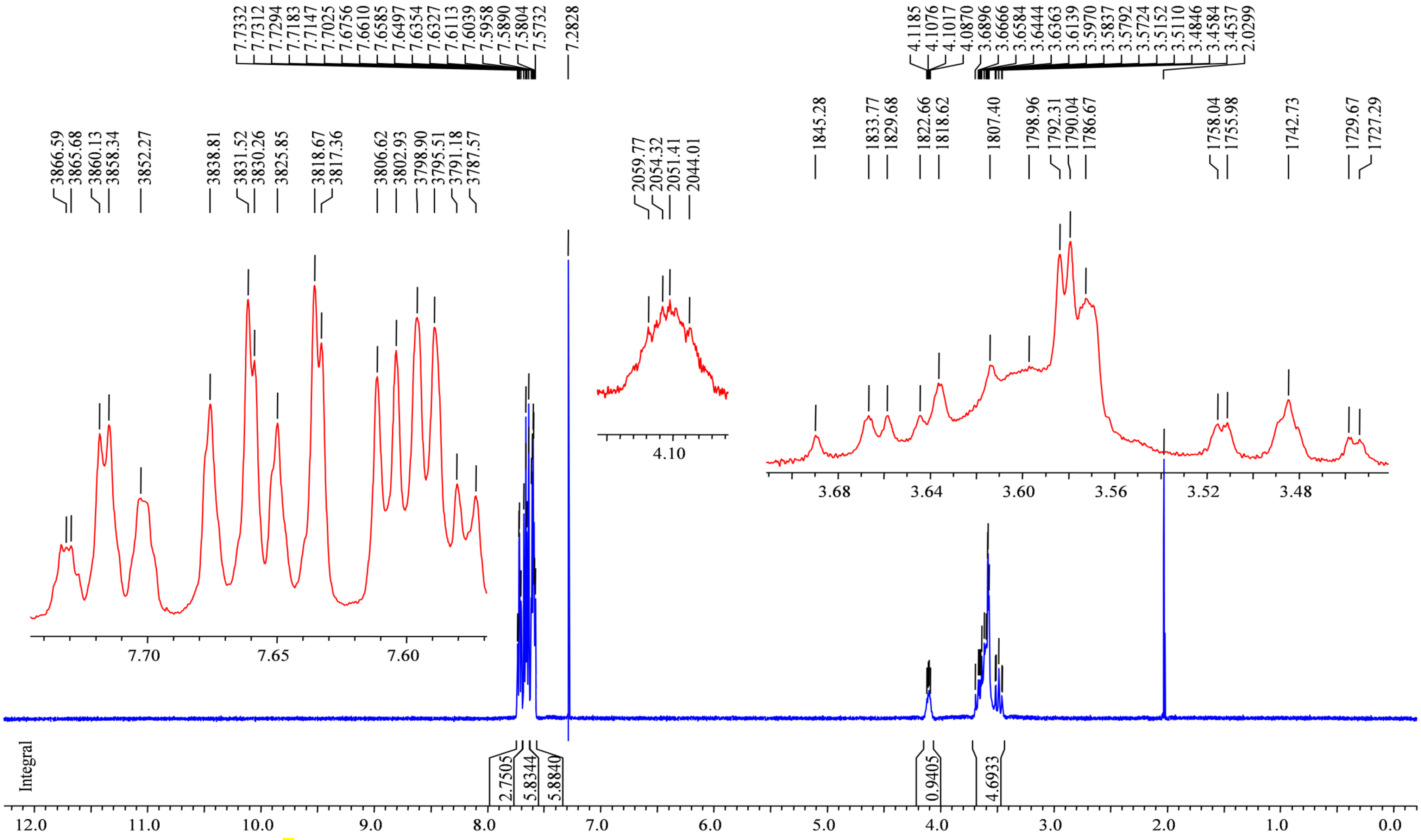

Figure 41. ^{1}H NMR spectrum (400 MHz, $CDCl_3$ + 10% acetone-d_6) of compound (**3b**).

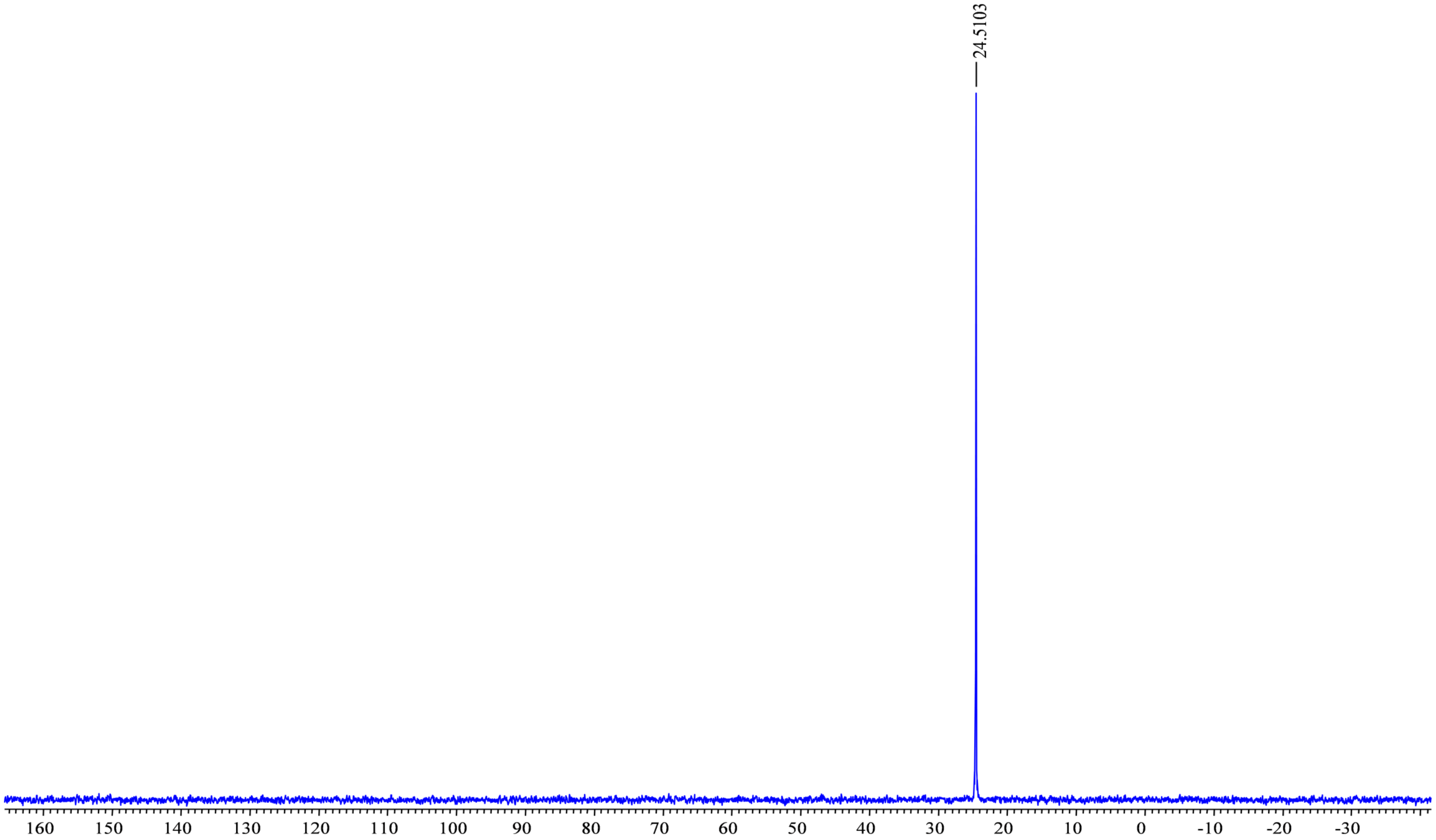

Figure 42. ^{31}P-{^{1}H} NMR spectrum (162.0 MHz, $CDCl_3$) of compound (**3b**).

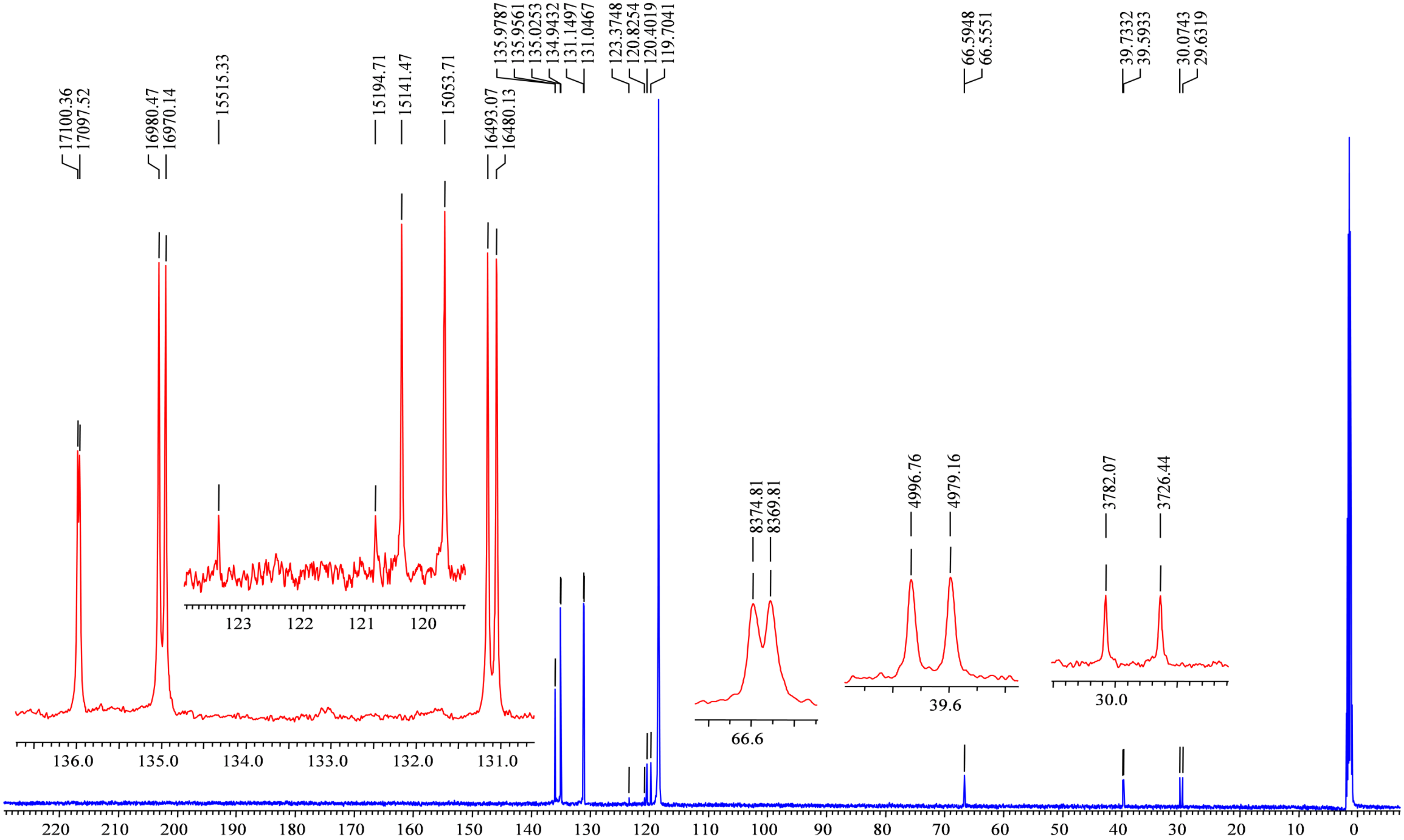

Figure 43. ^{13}C-{^1H} NMR spectrum (125.76 MHz, CD_3CN) of compound (**3b**).

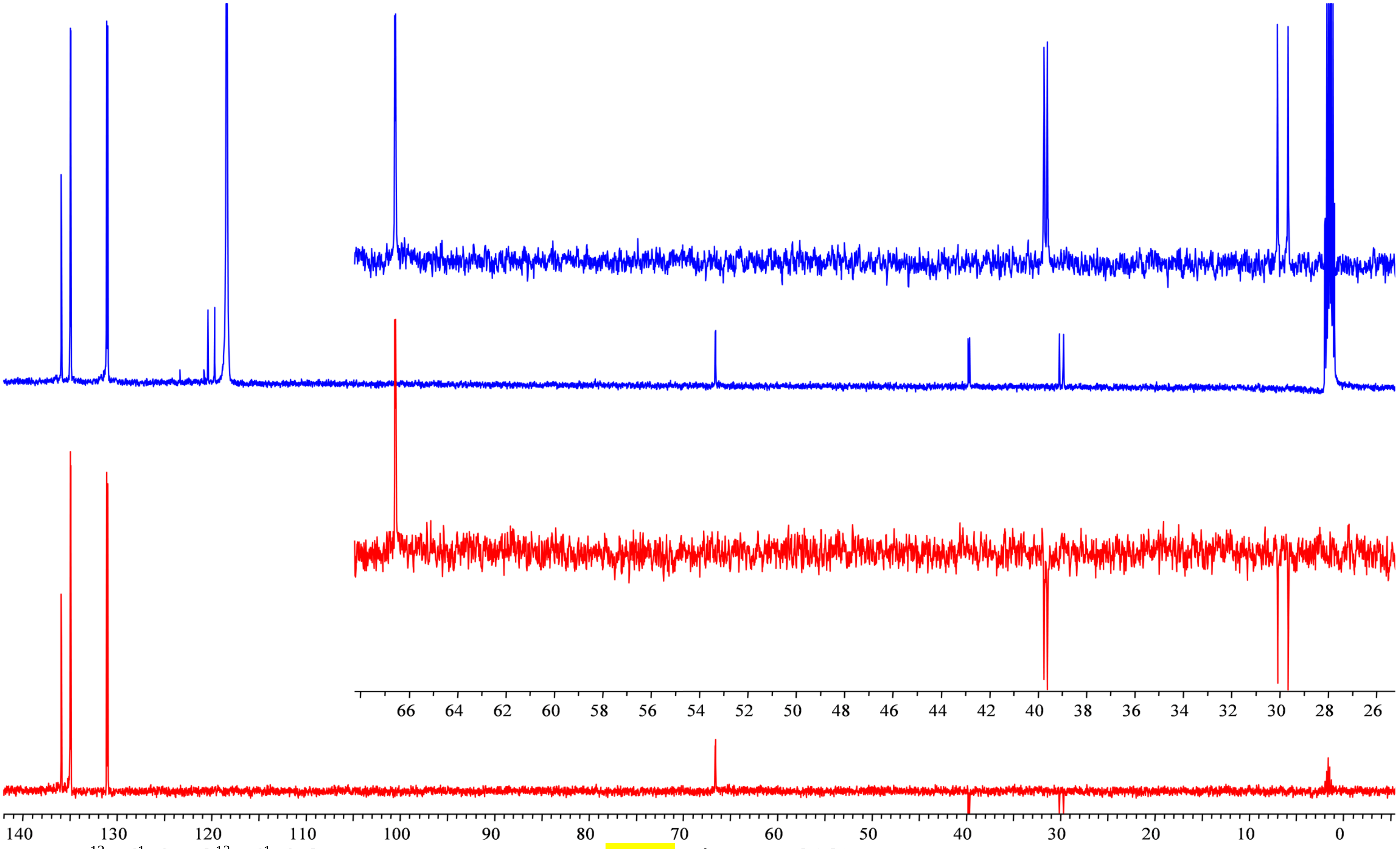

Figure 44. ^{13}C-{^{1}H} and ^{13}C-{^{1}H}-dept NMR spectra (125.76 MHz, CD_3CN) of compound (**3b**).

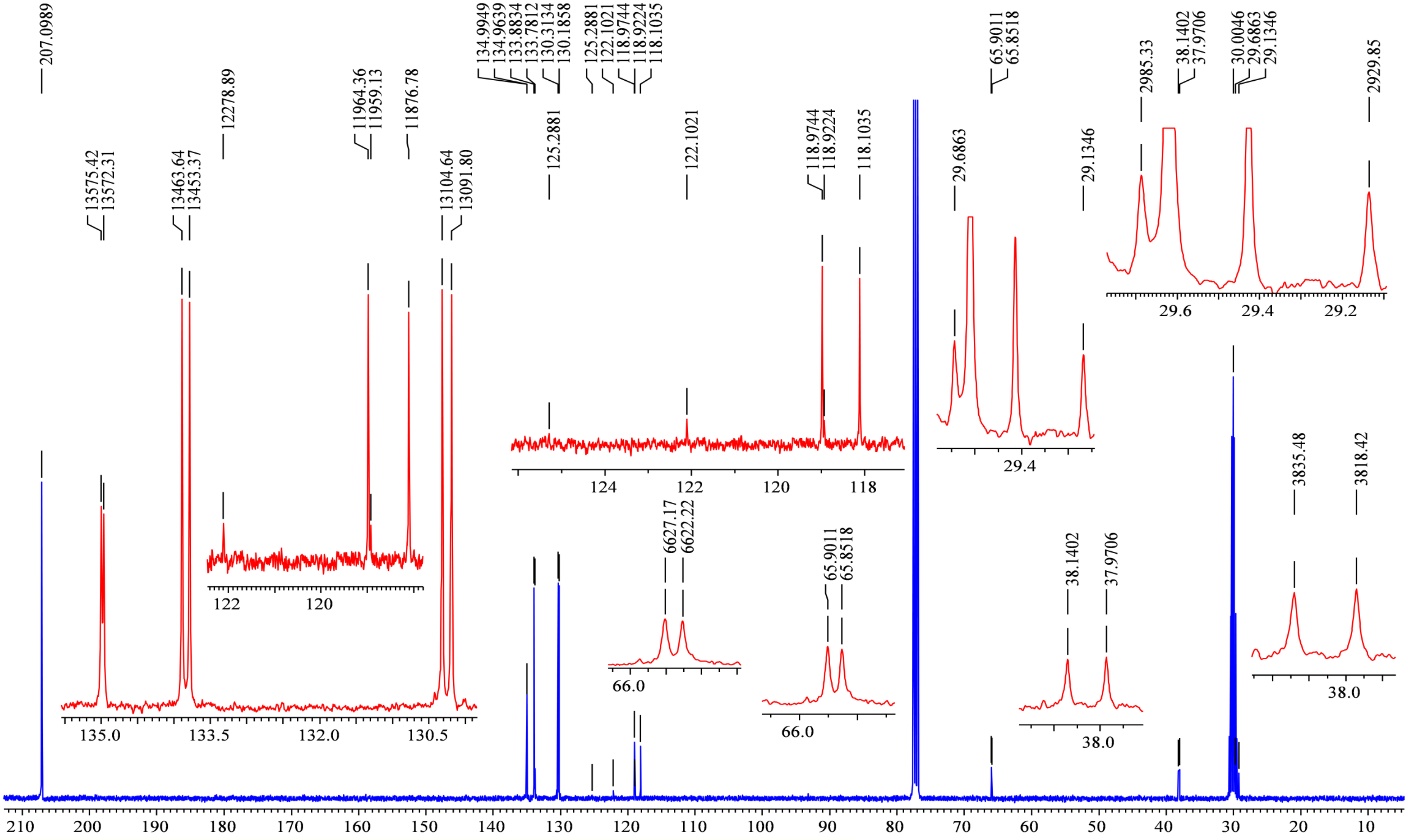

Figure 45. $^{13}C-\{^{1}H\}$ NMR spectrum (100.6 MHz, $CDCl_3$ + 10% acetone-d_6) of compound (**3b**).

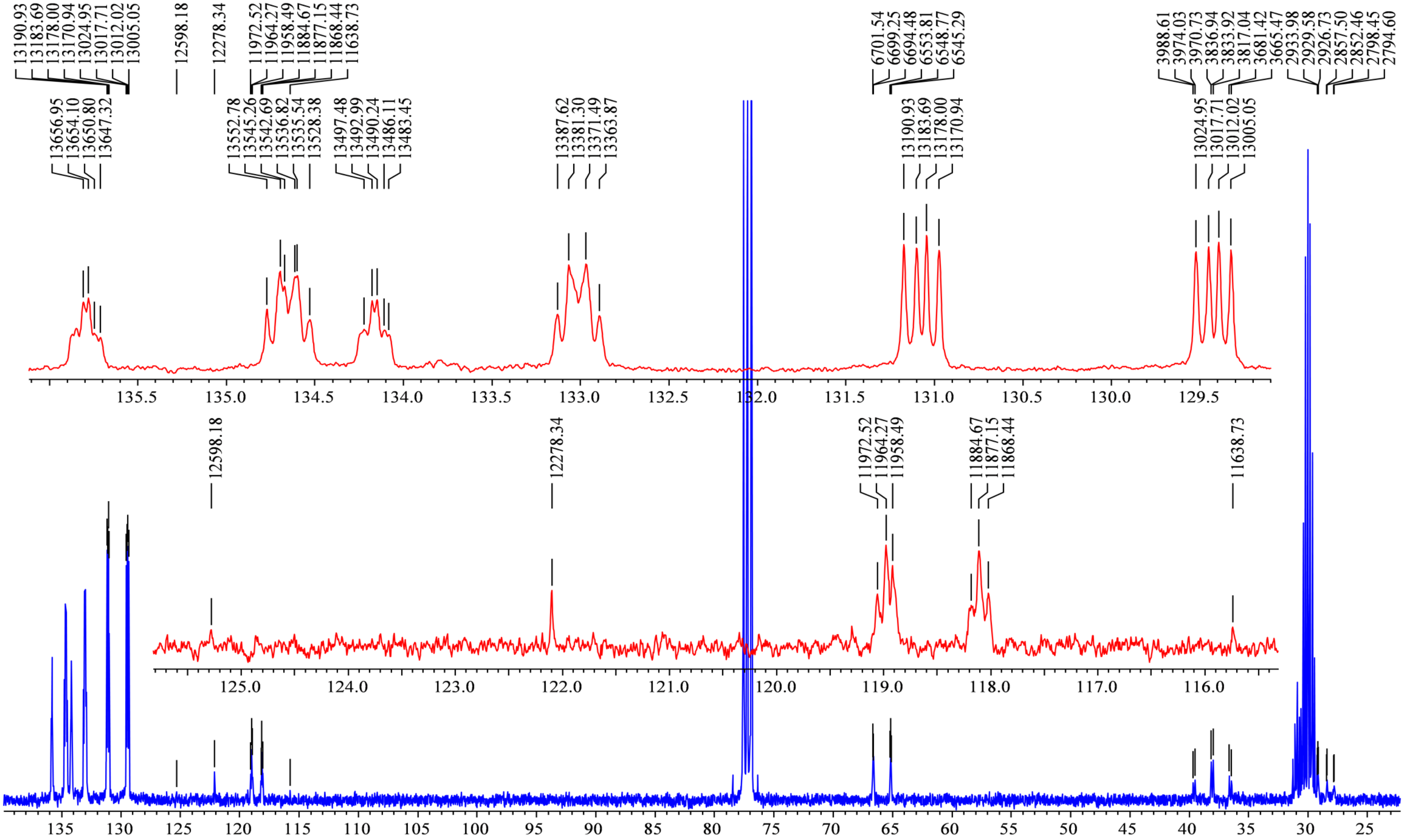

Figure 46. ^{13}C NMR spectrum (100.6 MHz, $CDCl_3$ + 10% acetone-d_6) of compound (**3b**).

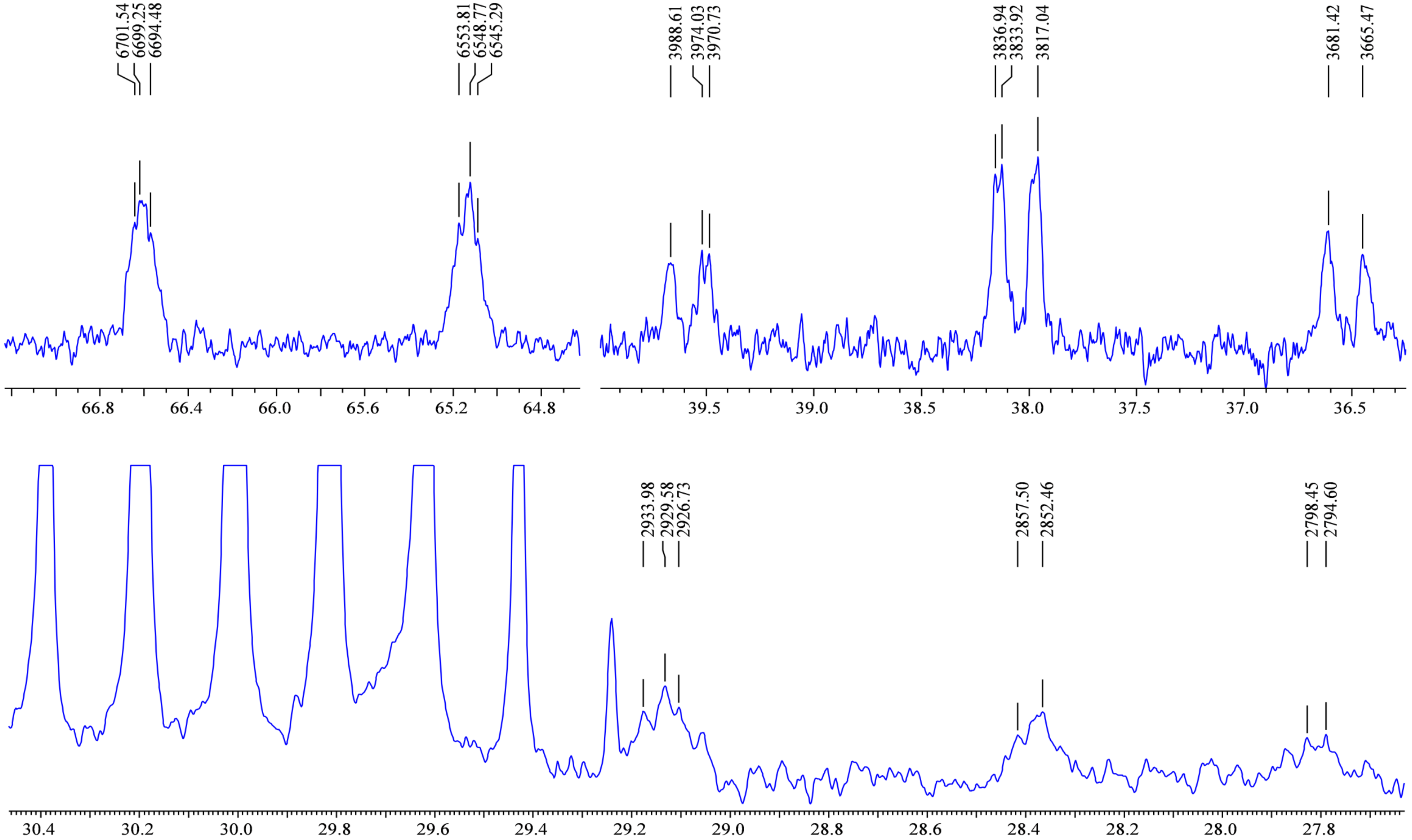

Figure 47. High-field fragments of ^{13}C NMR spectrum (100.6 MHz, $CDCl_3$ + 10% acetone-d_6) of compound (**3b**).

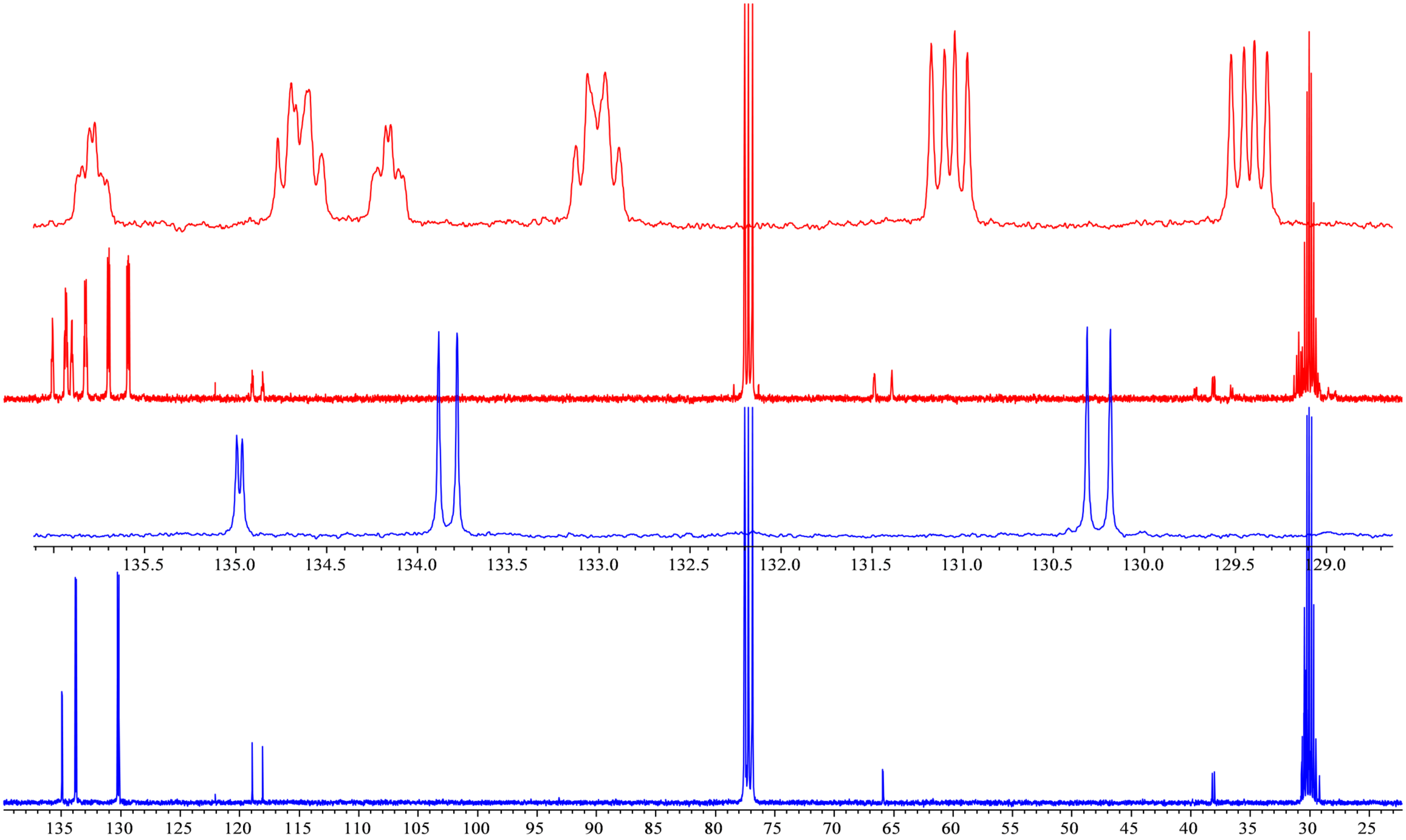

Figure 48. ^{13}C-{^{1}H} and ^{13}C NMR spectra (100.6 MHz, $CDCl_3$ + 10% acetone-d_6) of compound (**3b**).

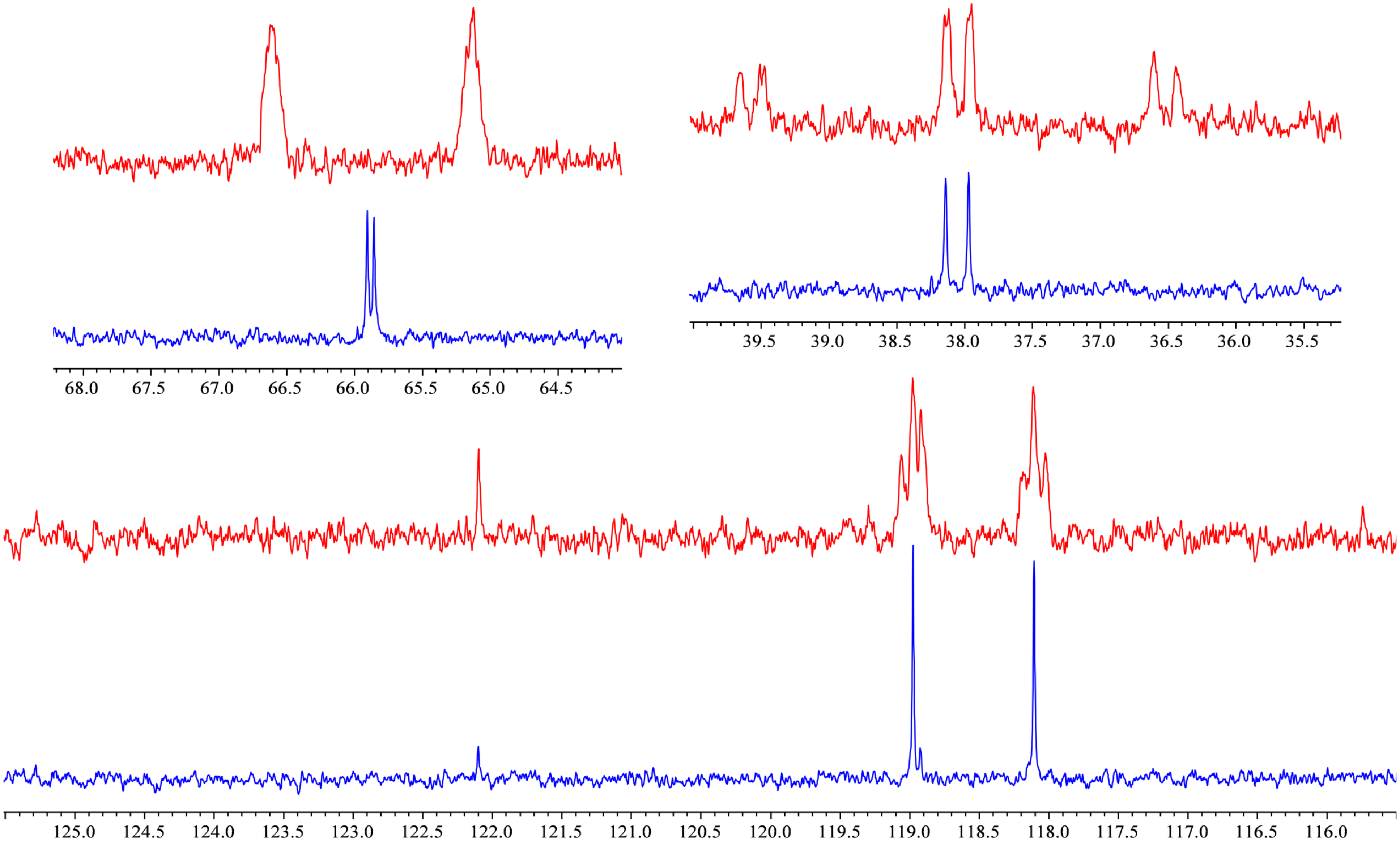

Figure 49. Fragments of ^{13}C-{^{1}H} and ^{13}C NMR spectra (100.6 MHz, $CDCl_3$ + 10% acetone-d_6) of compound (**3b**).

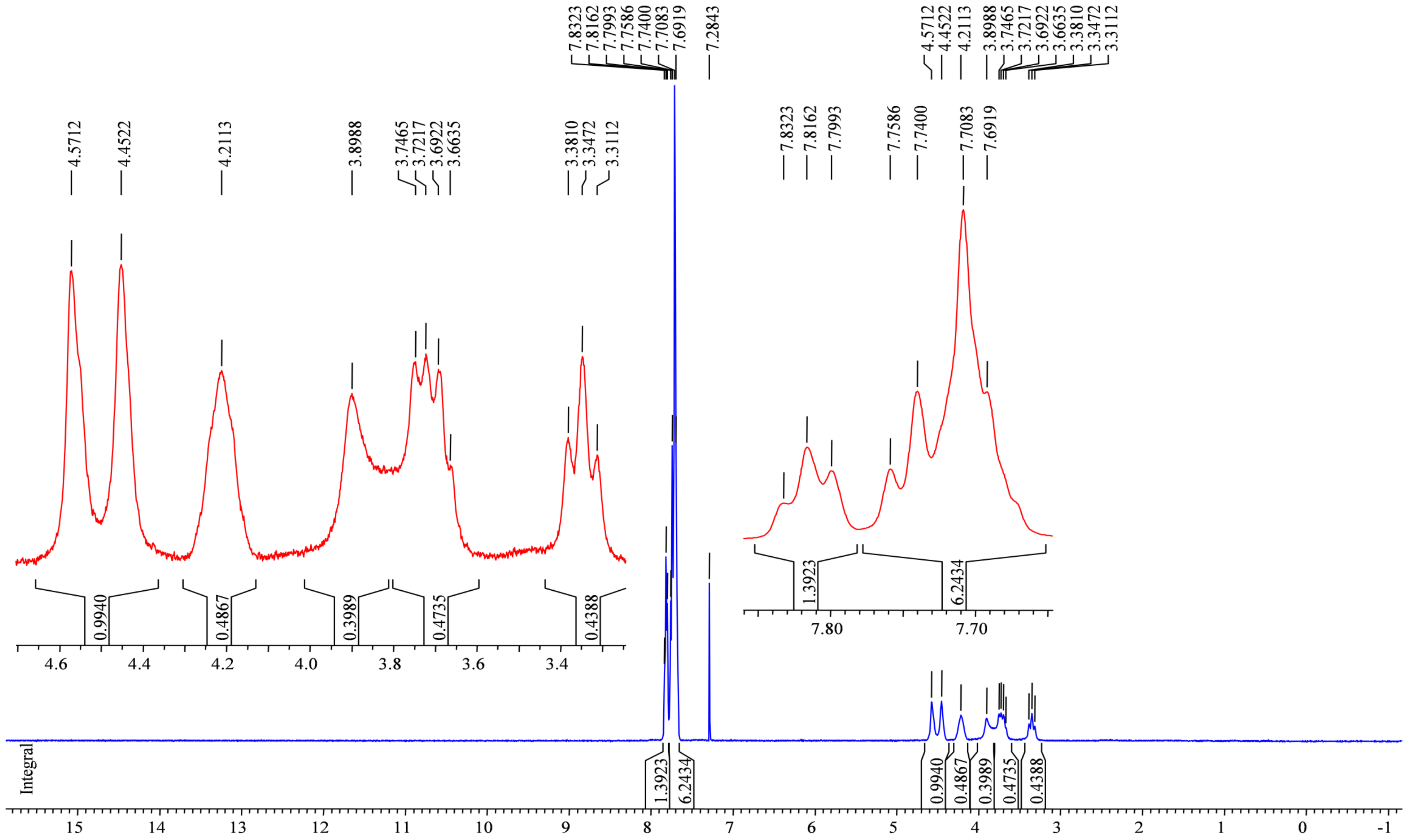

Figure 50. ^{1}H NMR spectrum (400 MHz, $CDCl_3$) of compound (**3c**).

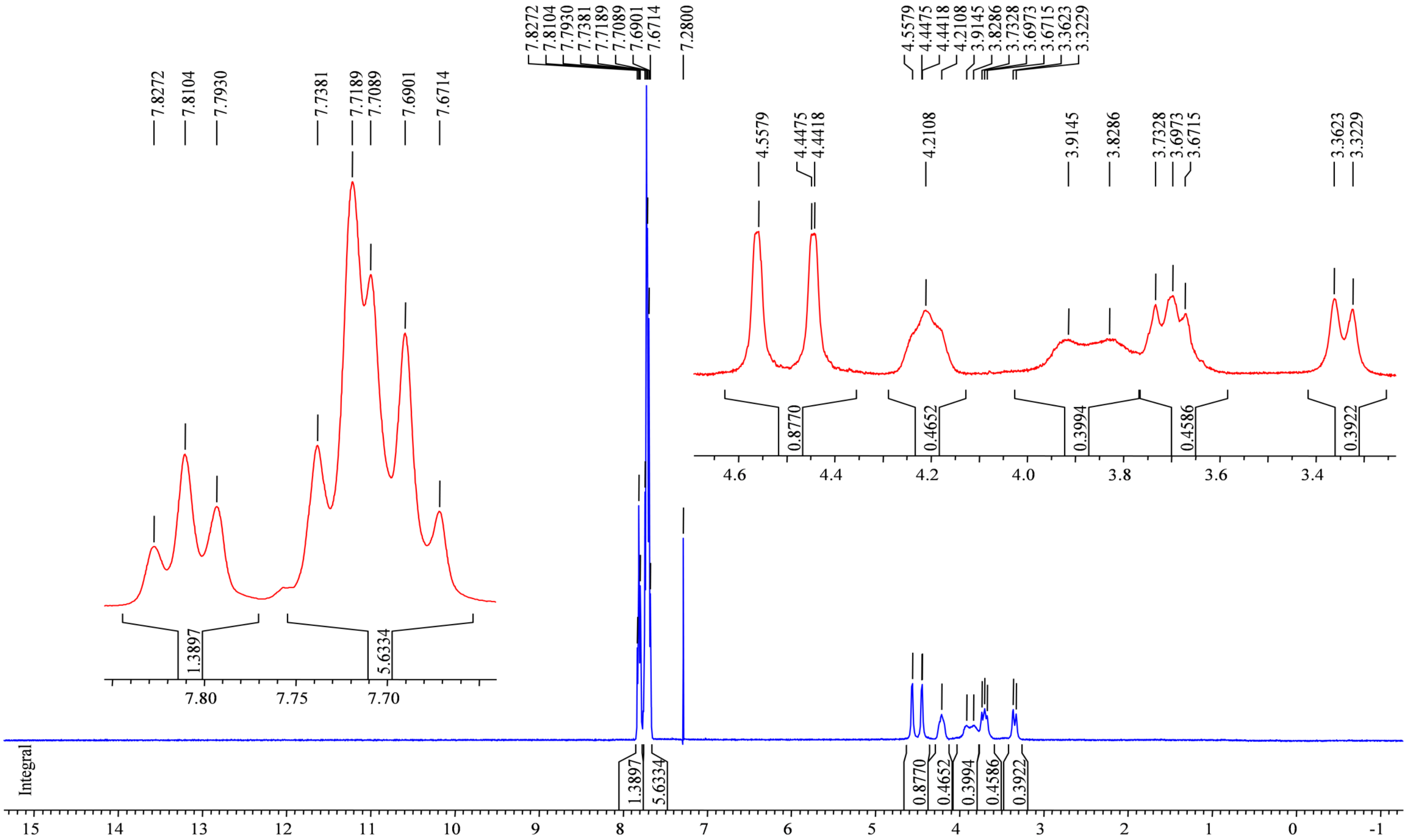

Figure 51. ^{1}H-{^{31}P} NMR spectrum (400 MHz, $CDCl_3$) of compound (**3c**).

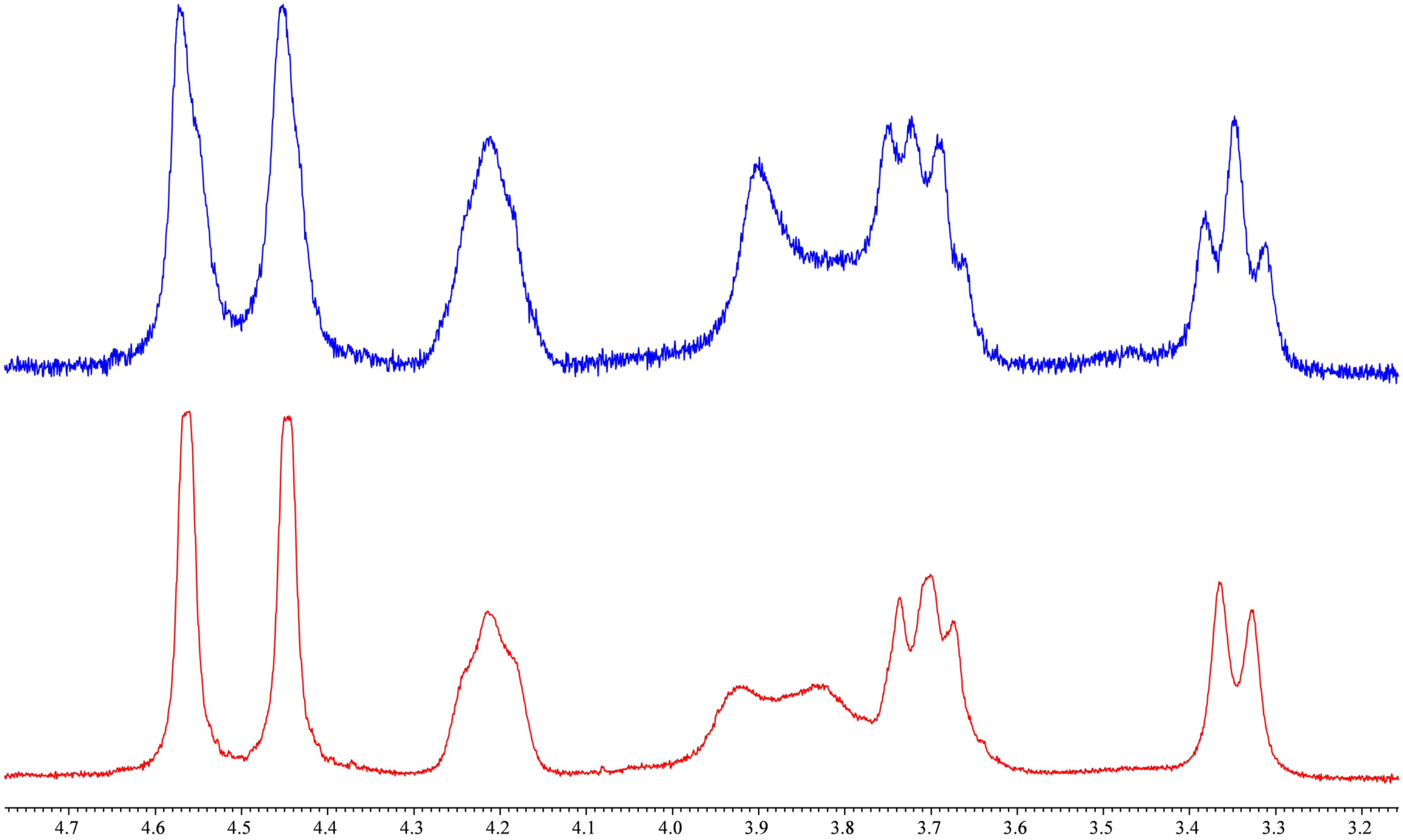

Figure 52. High field fragment of ^{1}H and ^{1}H-{^{31}P} NMR spectra (400 MHz, $CDCl_3$) of compound (**3c**).

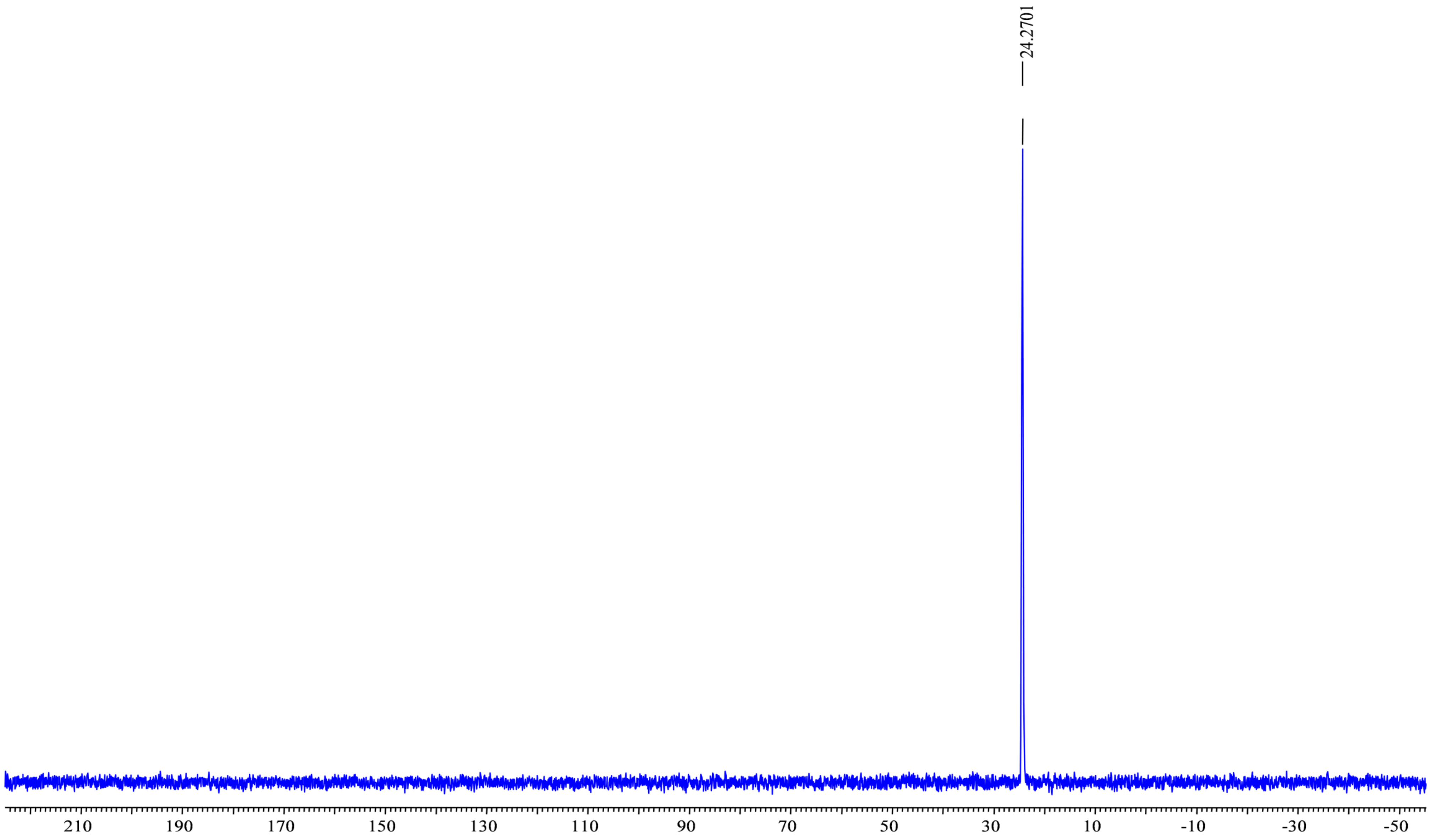

Figure 53. ^{31}P-{^{1}H} NMR spectrum (162.0 MHz, $CDCl_3$) of compound (**3c**).

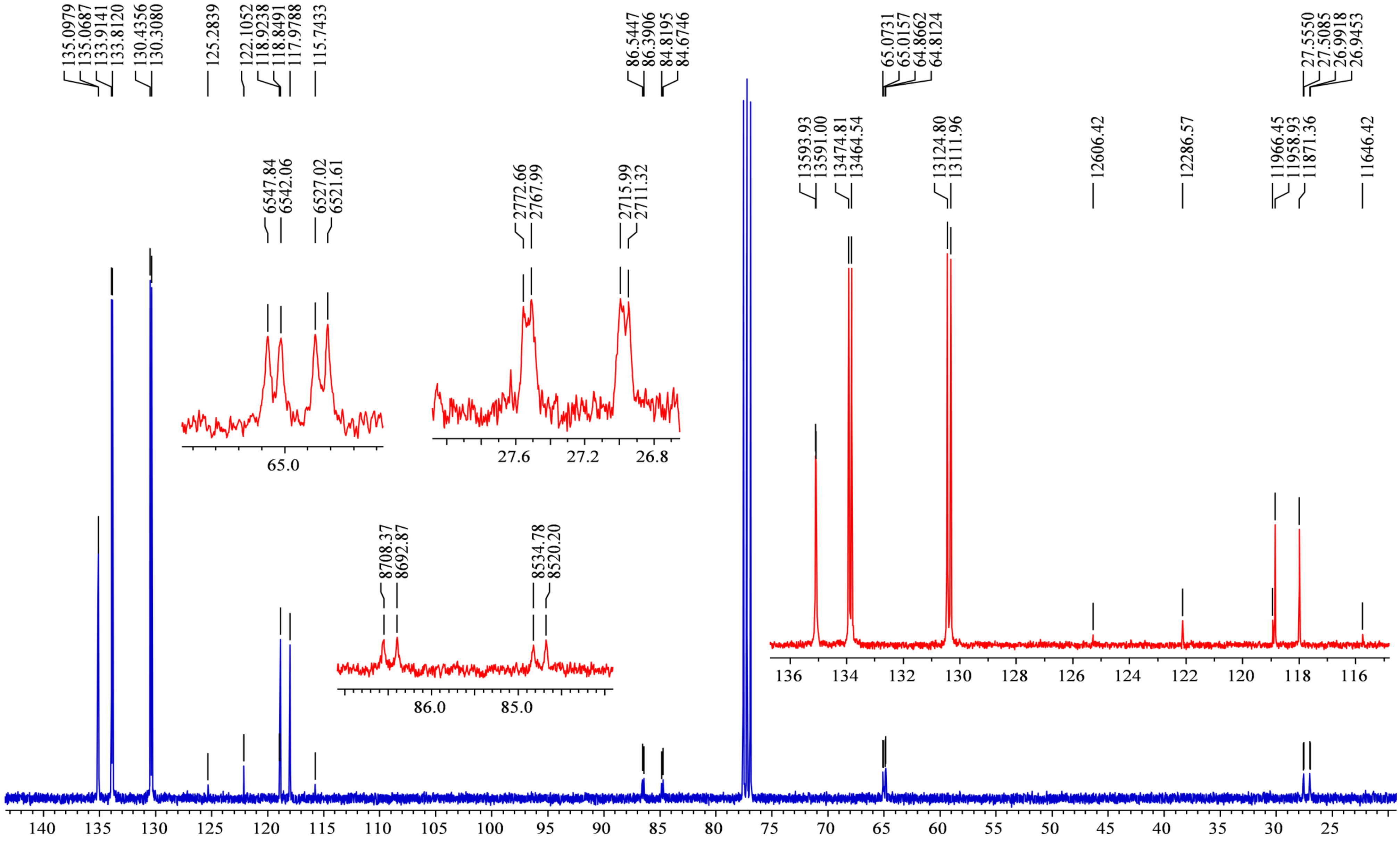

Figure 54. ^{13}C-{^{1}H} NMR spectrum (100.6 MHz, $CDCl_3$) of compound (**3c**).

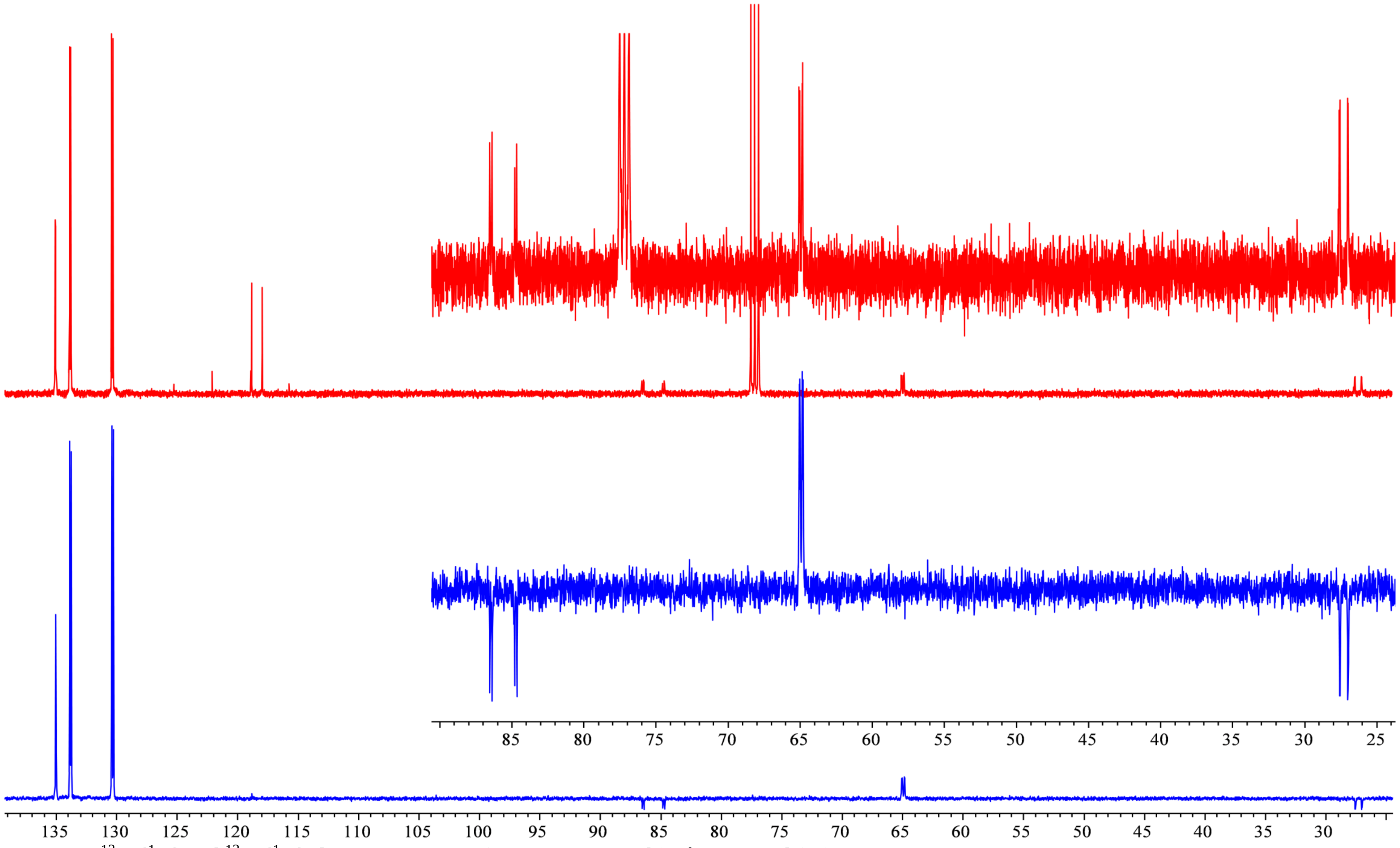

Figure 55. $^{13}C-\{^1H\}$ and $^{13}C-\{^1H\}$-dept NMR spectra (100.6 MHz, $CDCl_3$) of compound (**3c**).

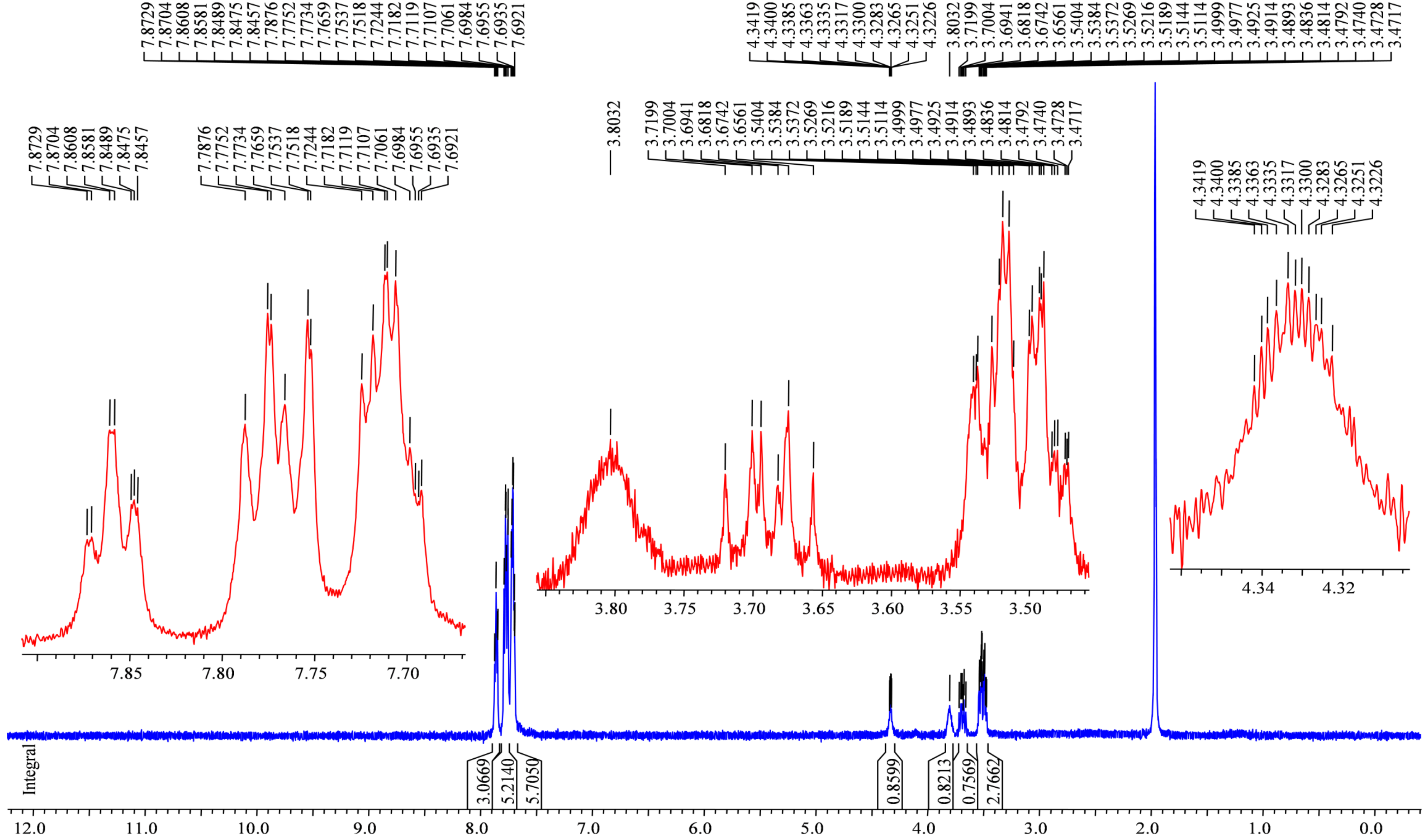

Figure 56. ^{31}P-{^{1}H} NMR spectrum (400 MHz, CD_3CN) of compound (**3d**).

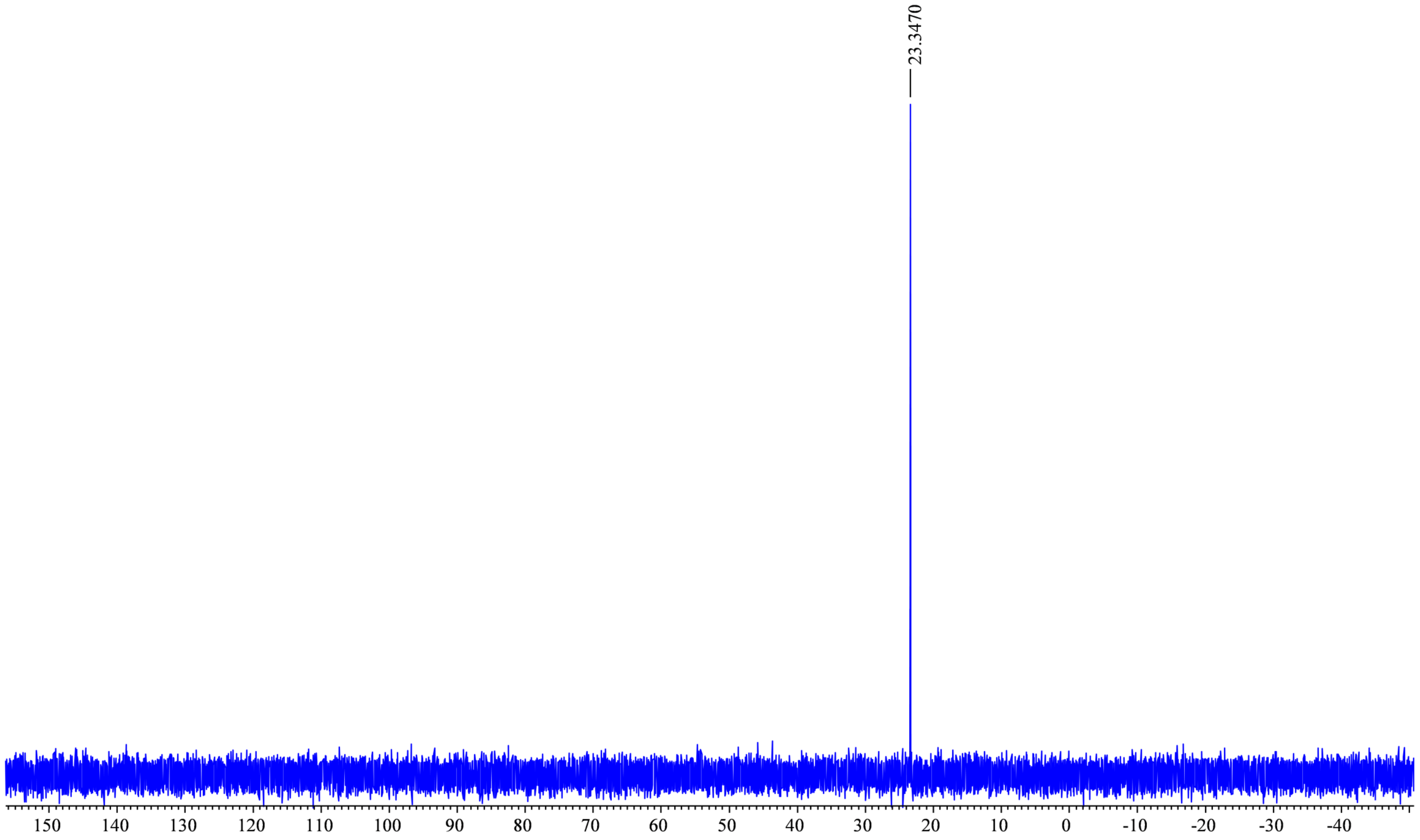

Figure 57. ^{31}P-{^{1}H} NMR spectrum (162.0 MHz, CD_3CN/ $CDCl_3$ = 1/1) of compound (**3d**).

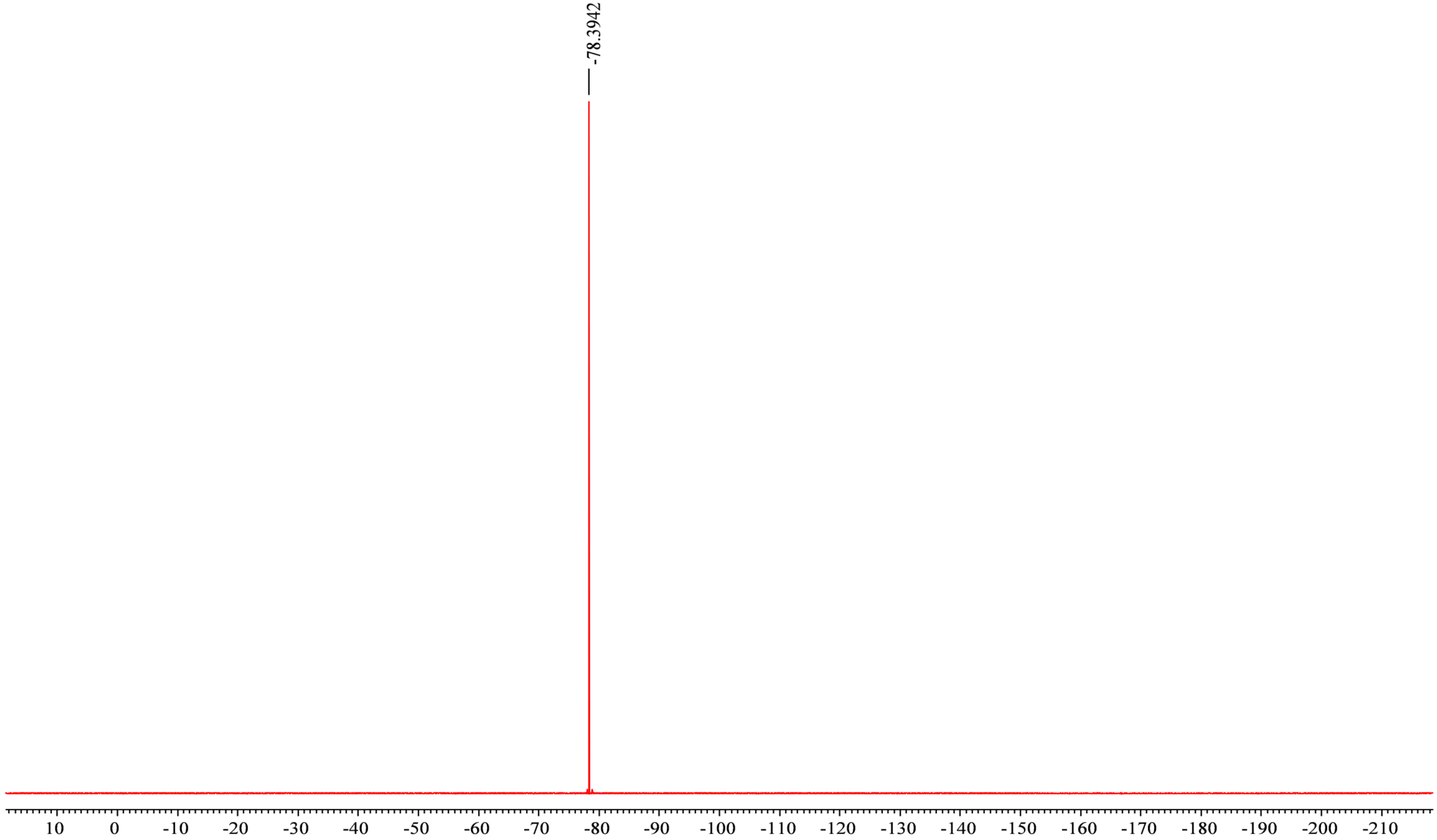

Figure 58. ^{31}F NMR spectrum (376.5 MHz, CD_3CN/ $CDCl_3$ = 1/1) of compound (**3d**).

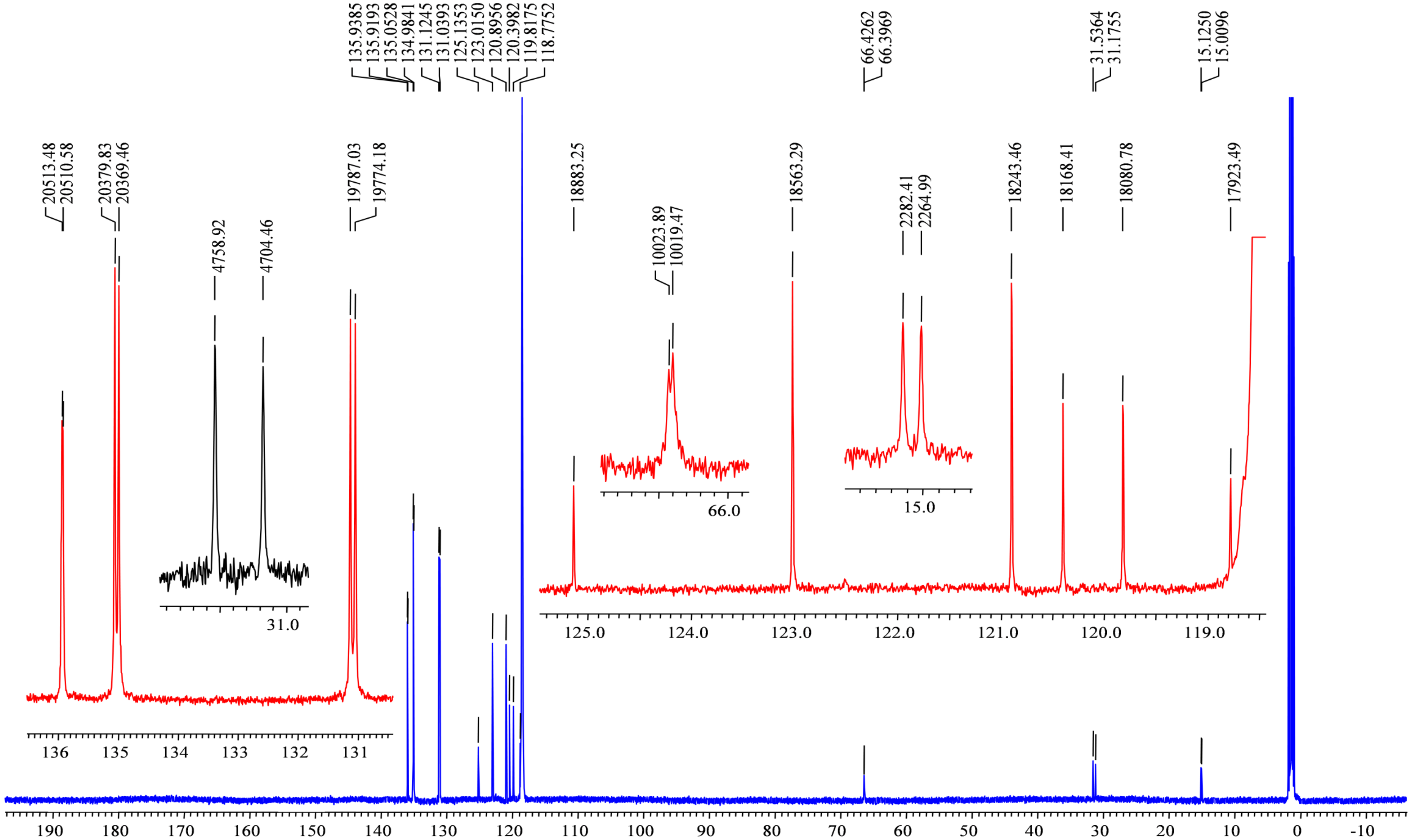

Figure 59. ^{13}C-{^{1}H} NMR spectrum (100.6 MHz, CD_3CN/ $CDCl_3$ = 1/1) of compound (**3d**).

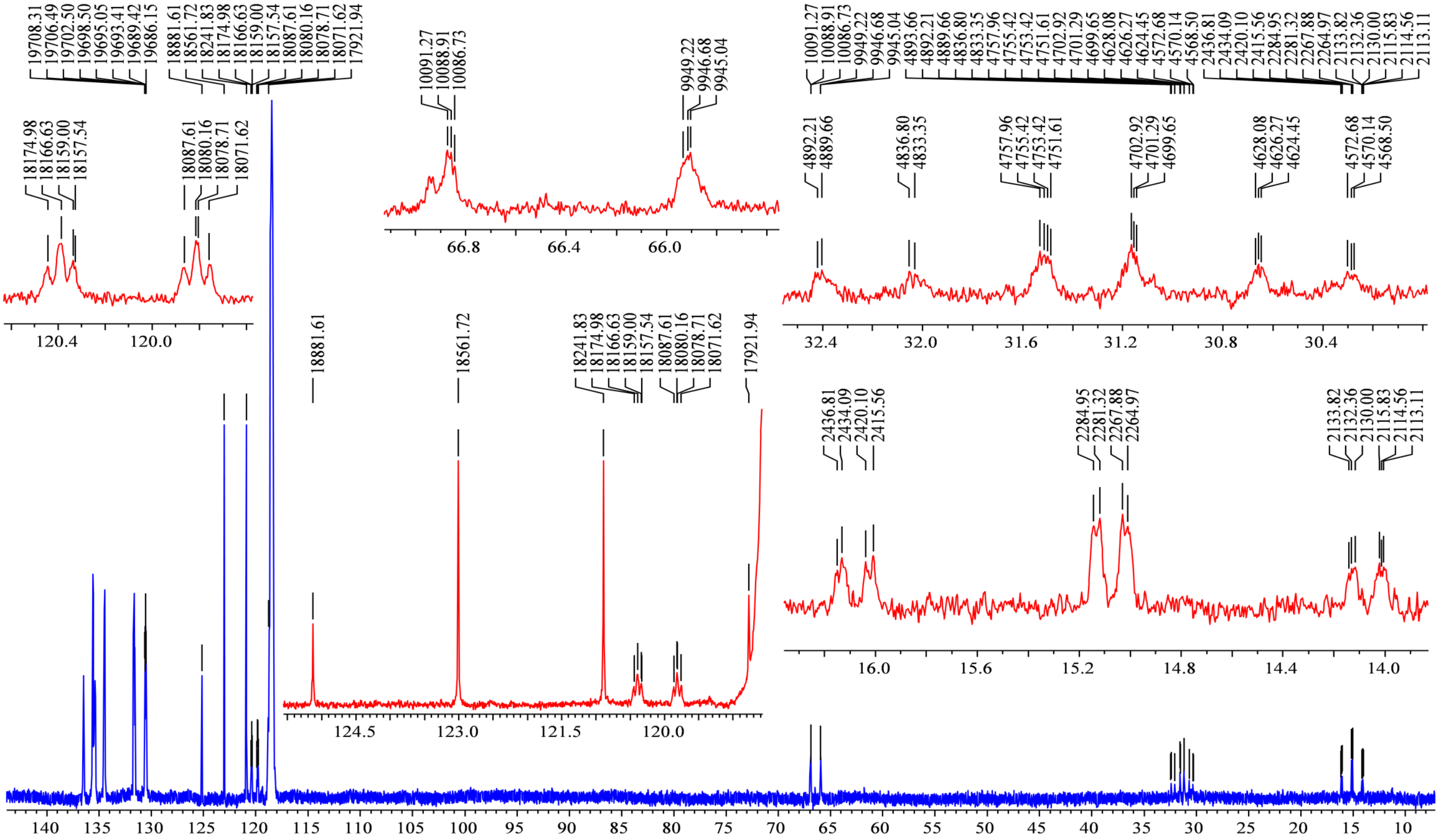

Figure 60. ^{13}C NMR spectrum (100.6 MHz, CD_3CN/ $CDCl_3$ = 1/1) of compound (**3d**).

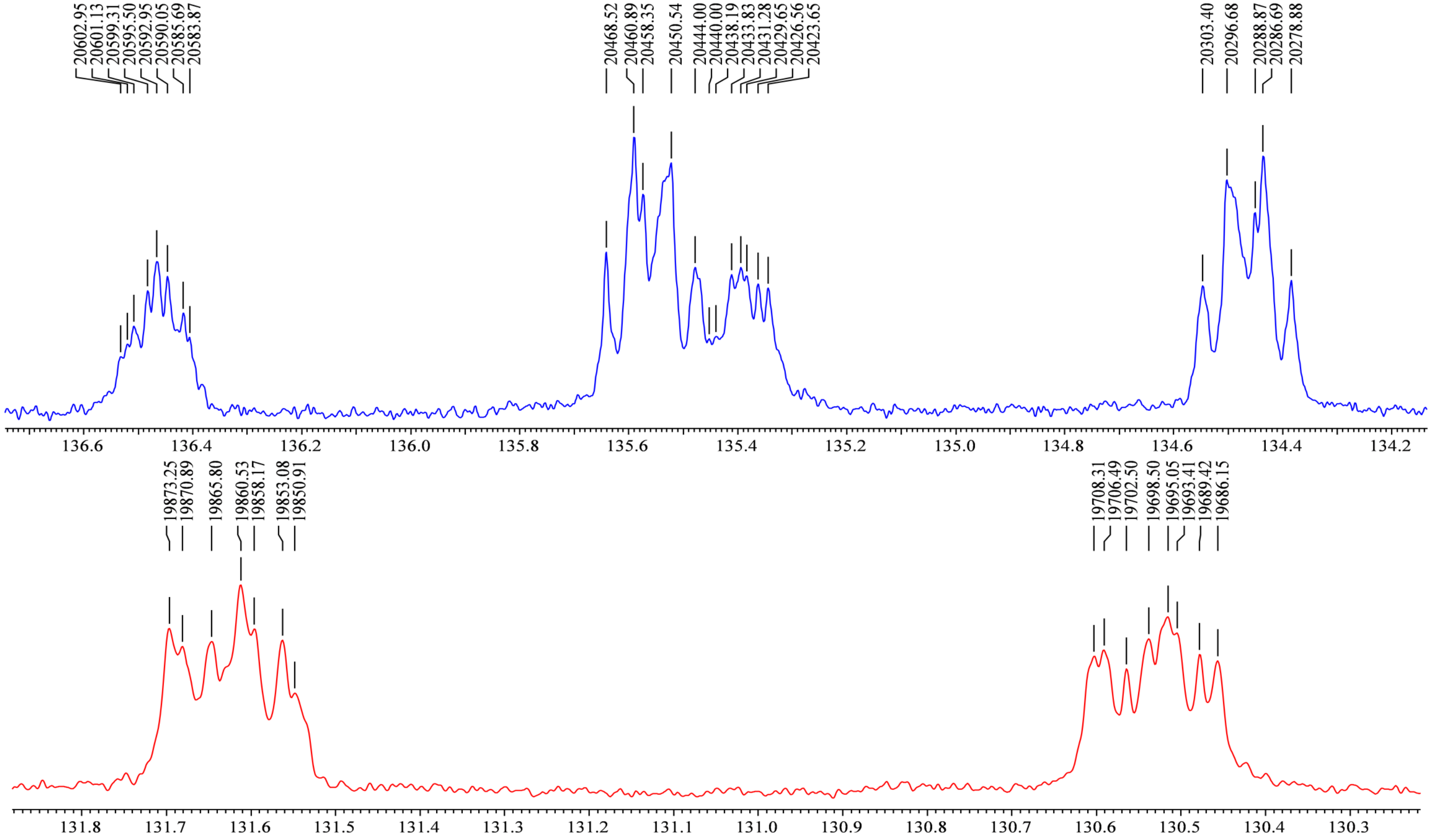

Figure 61. Low-field fragments of ^{13}C NMR spectrum (100.6 MHz, CD_3CN/ $CDCl_3$ = 1/1) of compound (**3d**).

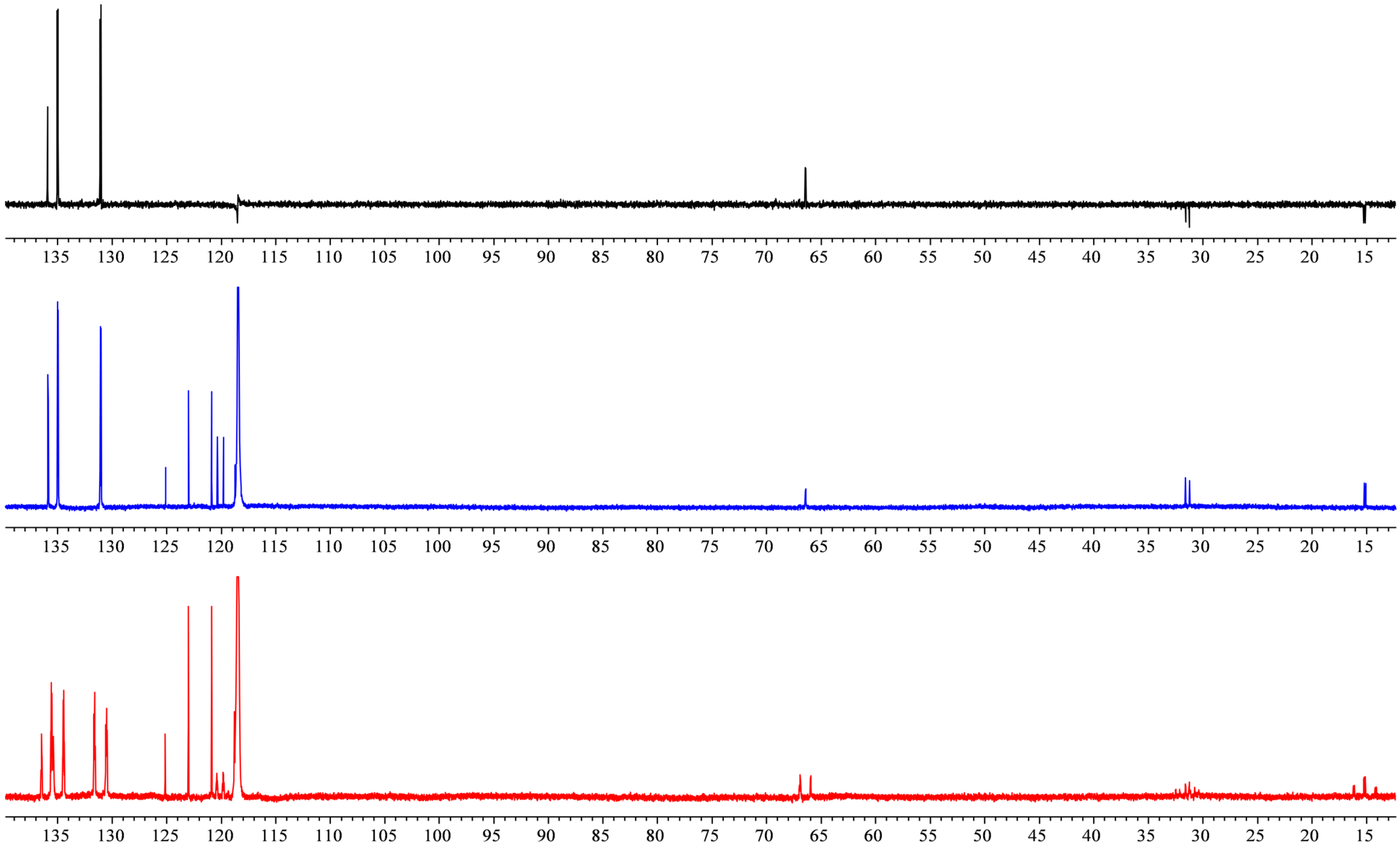

Figure 62. ^{13}C, ^{13}C-{^{1}H} and ^{13}C-{^{1}H}-dept NMR spectra (100.6 MHz, CD_3CN/ $CDCl_3$ = 1/1) of compound (**3d**).

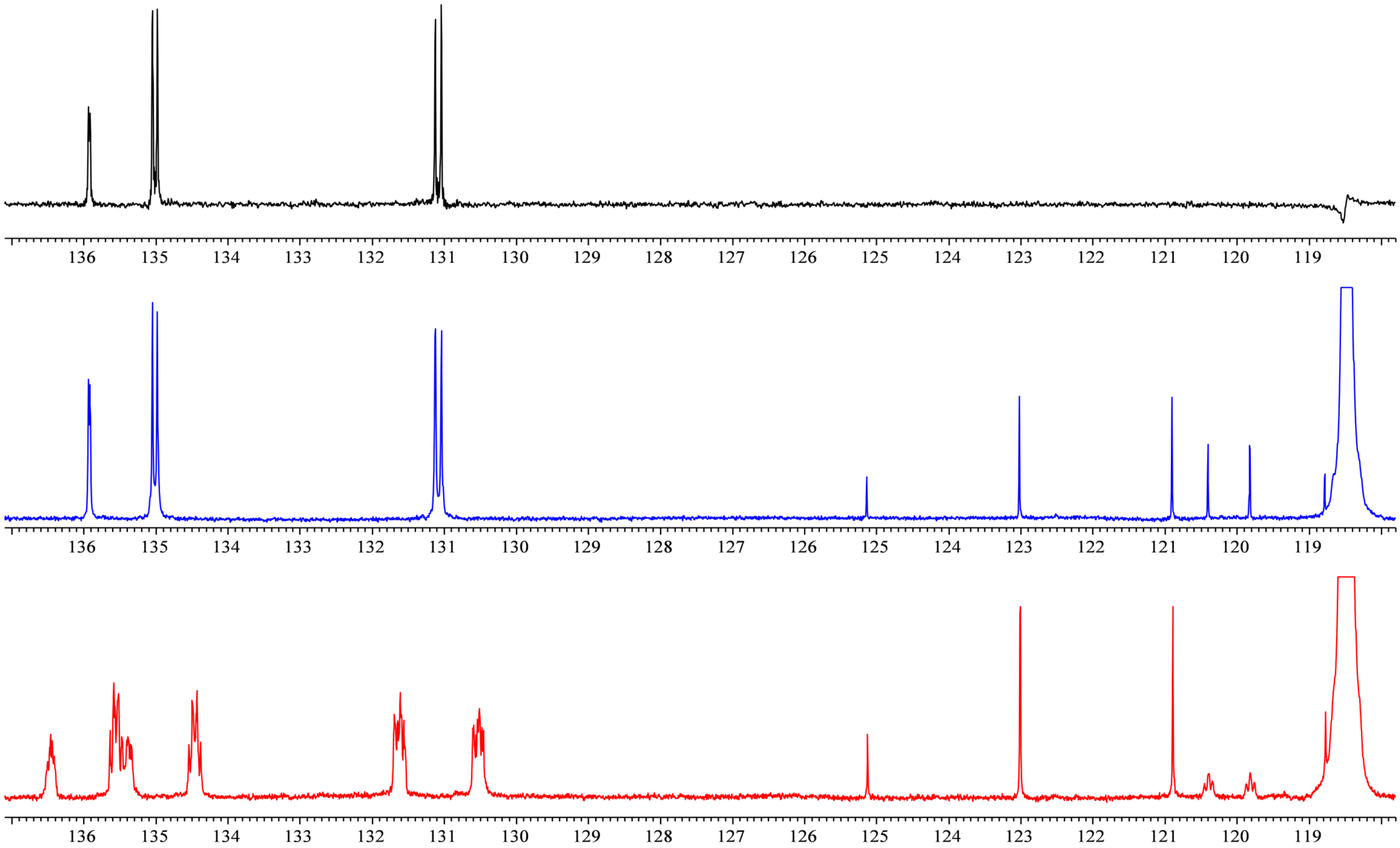

Figure 63. Low-field fragment of ^{13}C, ^{13}C-{^{1}H} and ^{13}C-{^{1}H}-dept NMR spectra (100.6 MHz, CD_3CN/ $CDCl_3$ = 1/1) of compound (**3d**).

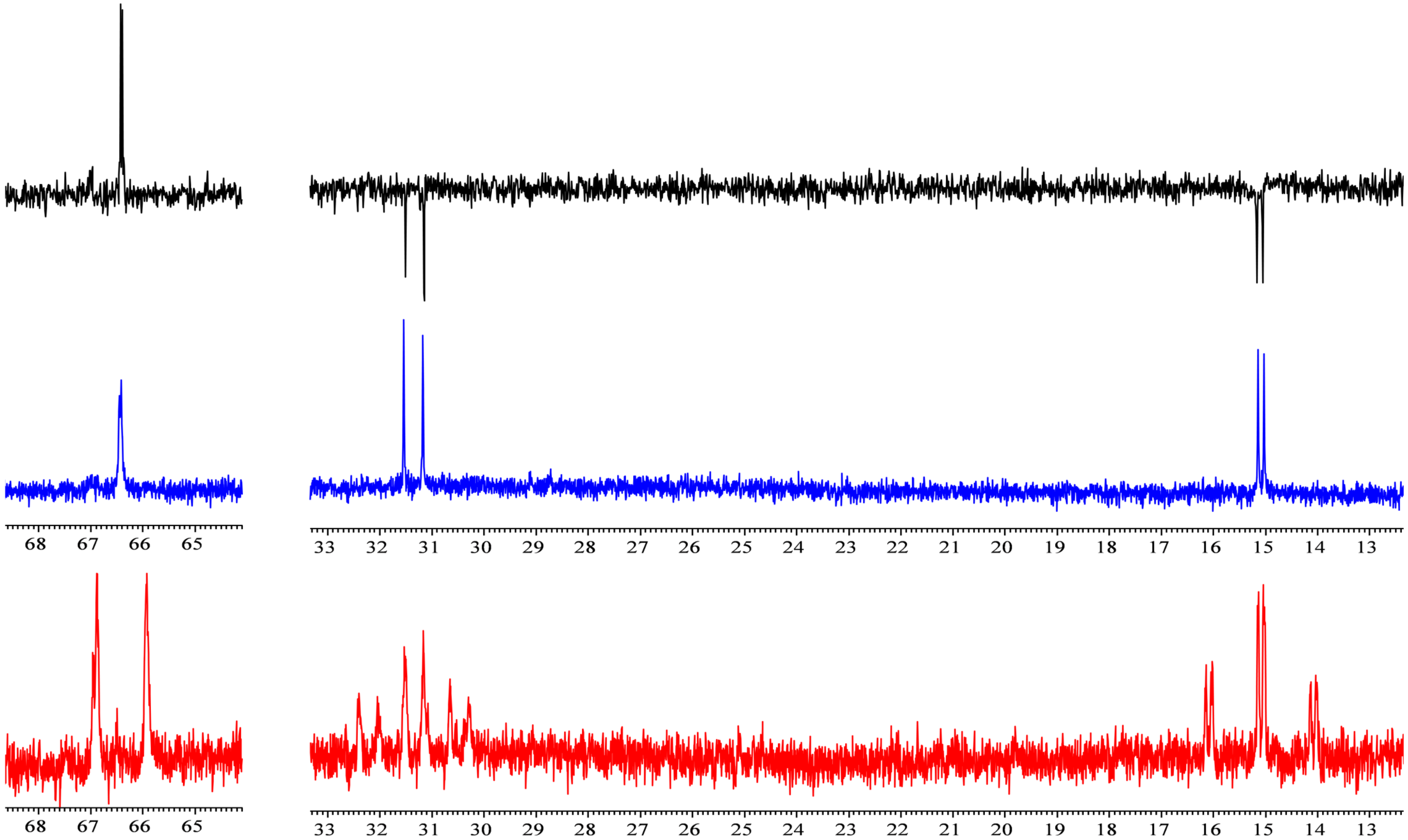

Figure 64. High-field fragment of ^{13}C, ^{13}C-{^{1}H} and ^{13}C-{^{1}H}-dept NMR spectra (100.6 MHz, CD_3CN/ $CDCl_3$ = 1/1) of compound (**3d**).

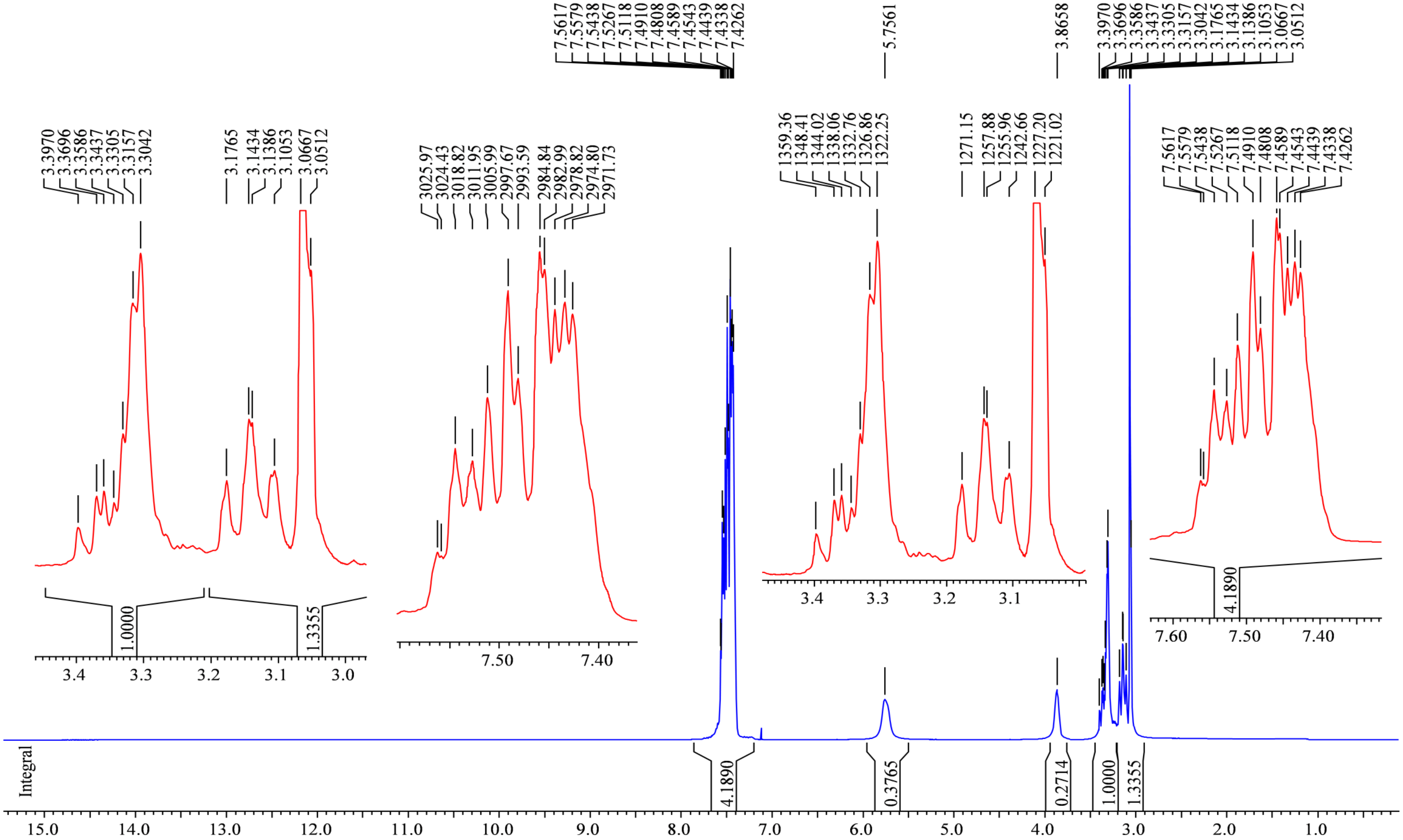

Figure 65. ^{1}H NMR spectrum (400 MHz, $CDCl_3$) of compound (**3f**).

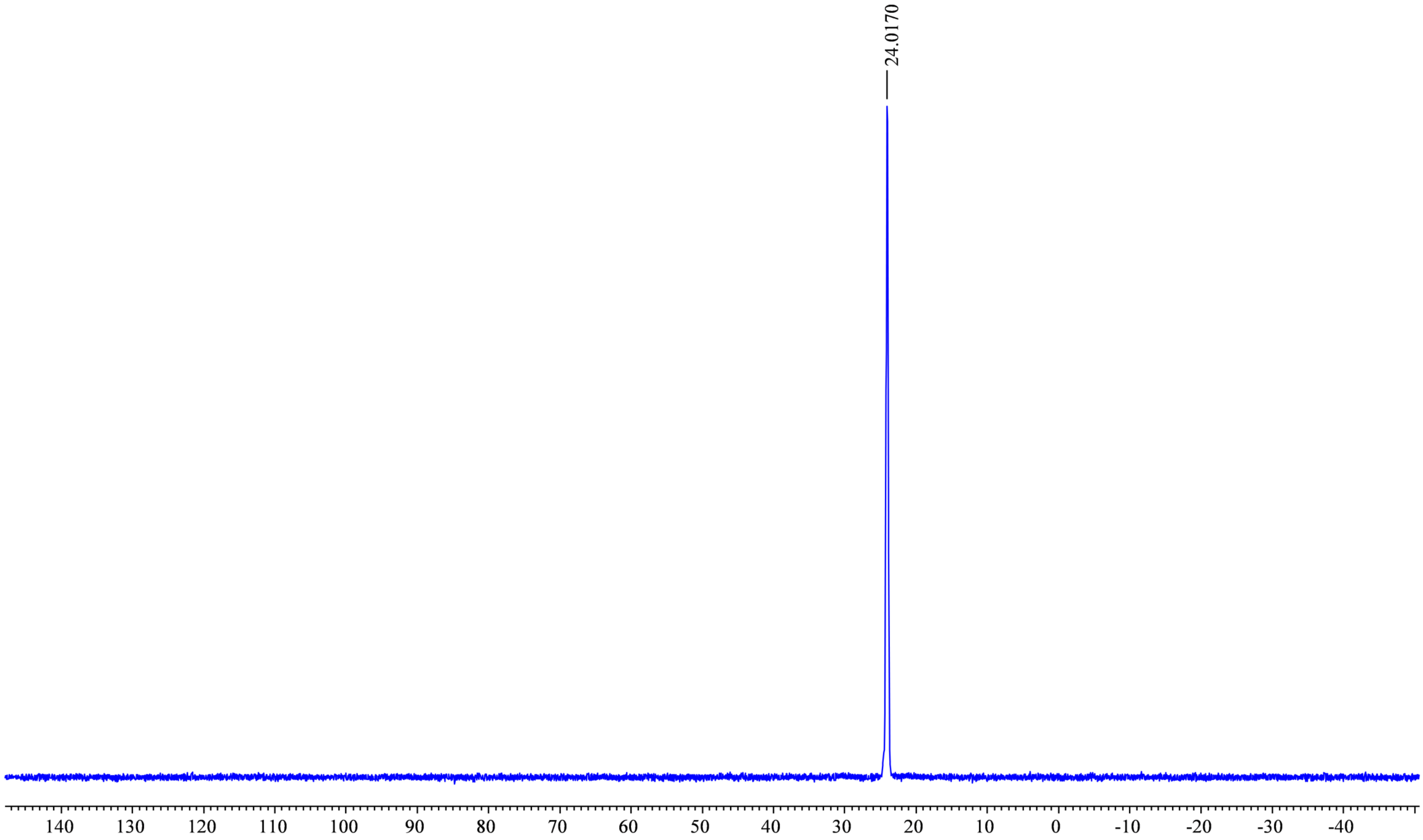

Figure 66. ^{31}P-{^{1}H} NMR spectrum (162.0 MHz, $CDCl_3$) of compound (**3f**).

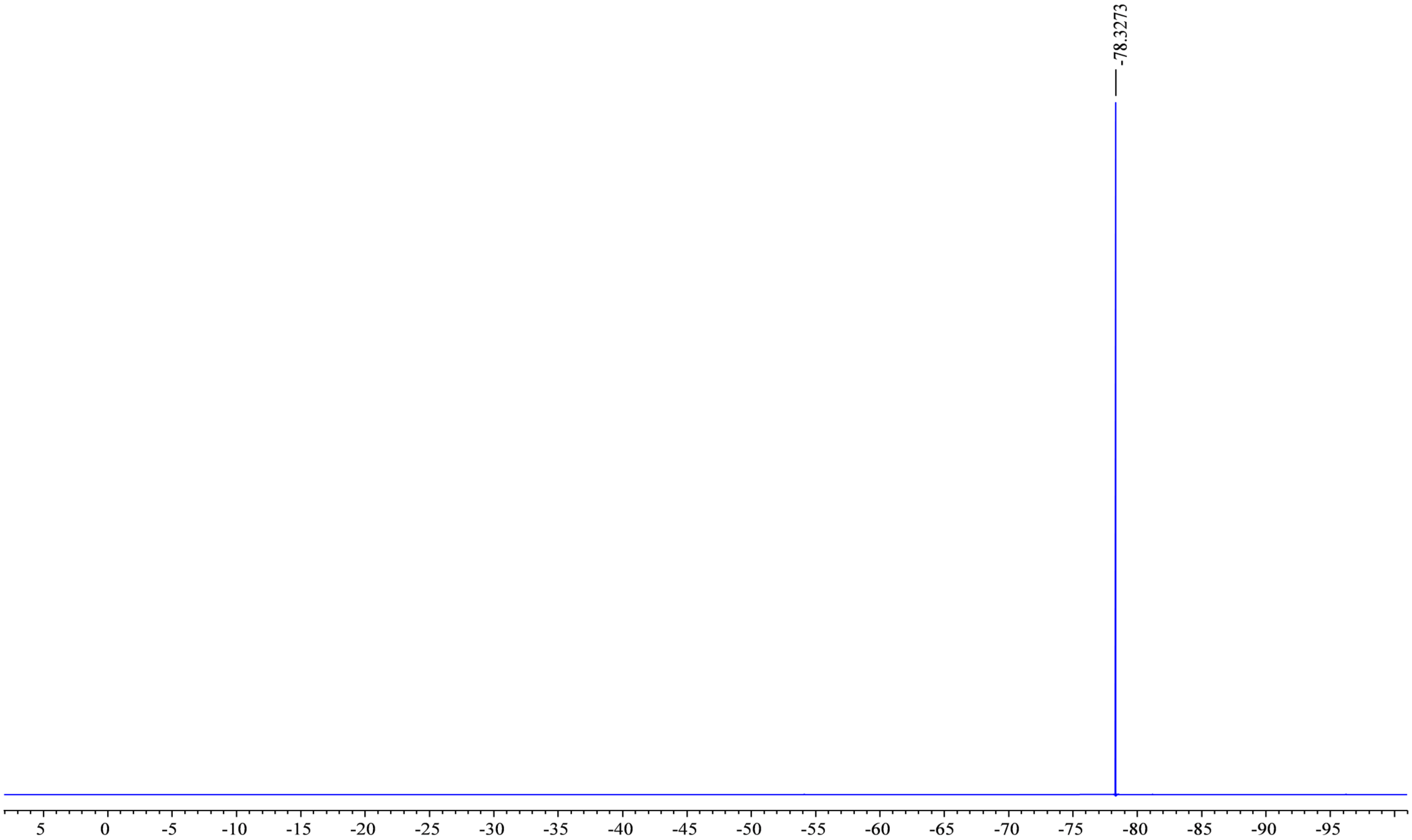

Figure 67. ^{19}F NMR spectrum (376.5 MHz, $CDCl_3$) of compound (**3f**).

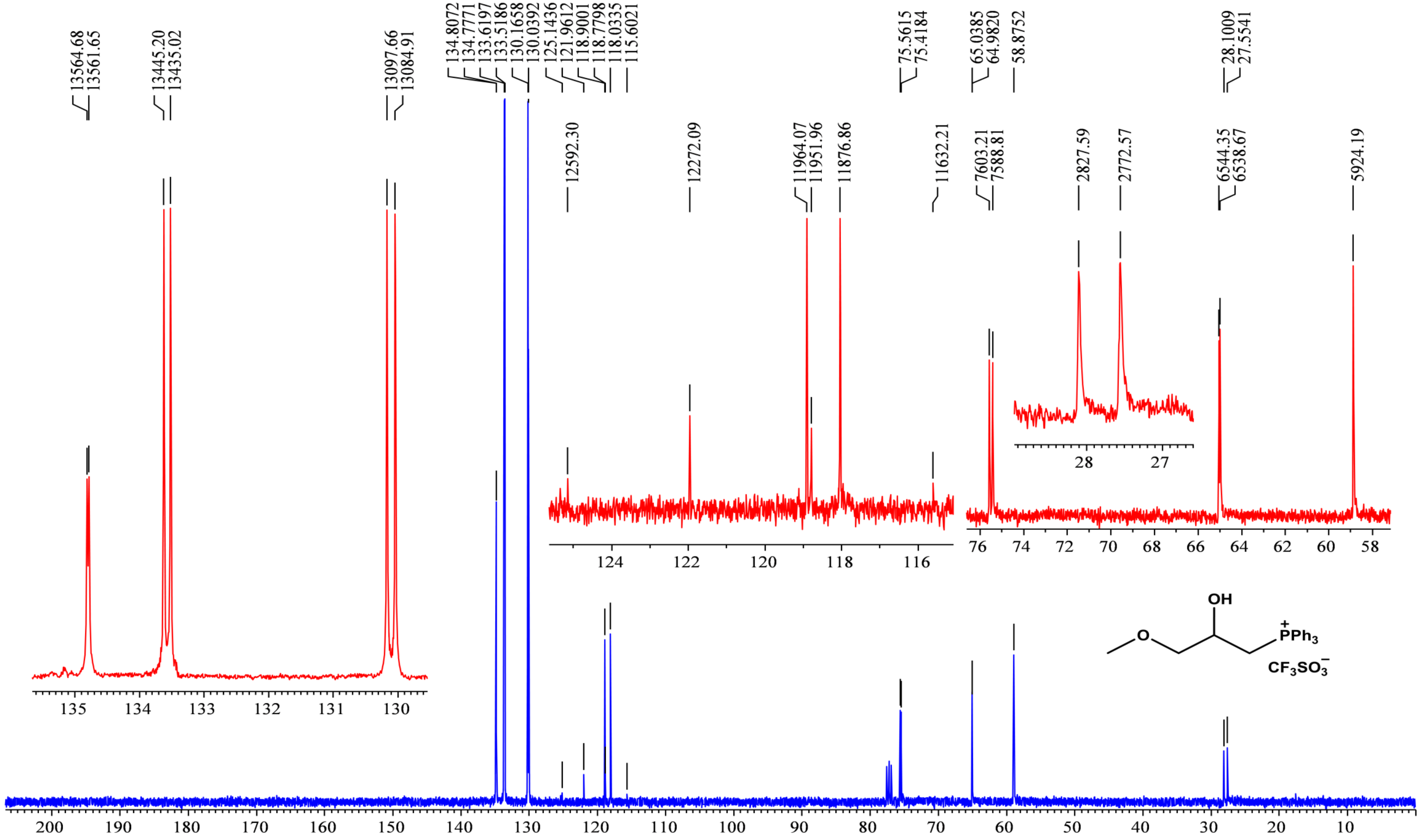

Figure 68. ^{13}C-{^{1}H} NMR spectrum (100.6 MHz, $CDCl_3$) of compound (**3f**).

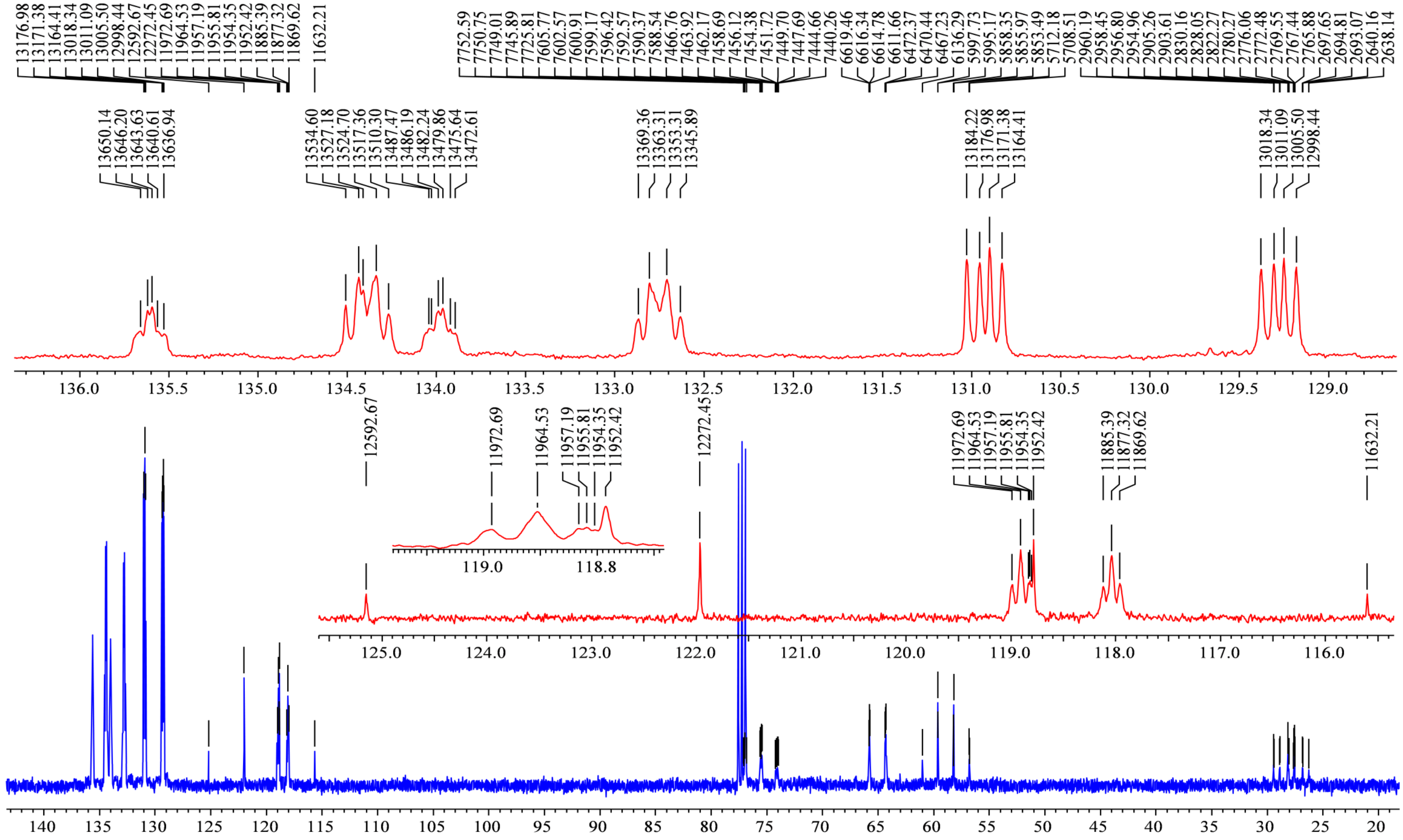

Figure 69. ^{13}C NMR spectrum (100.6 MHz, $CDCl_3$) of compound (**3f**).

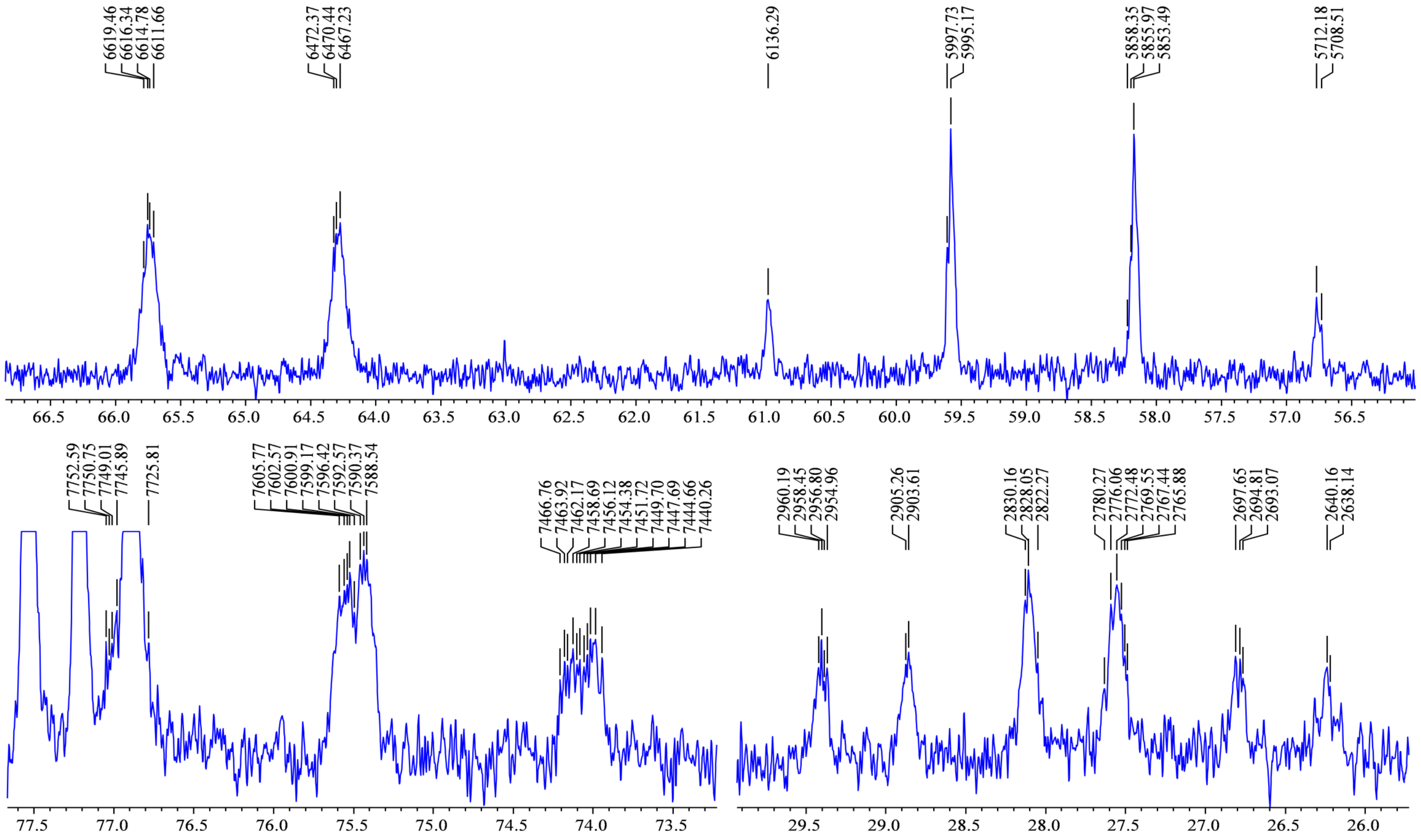

Figure 70. The 25-78 and 56-67 ppm regions of ^{13}C NMR spectrum (100.6 MHz, $CDCl_3$) of compound (**3f**).

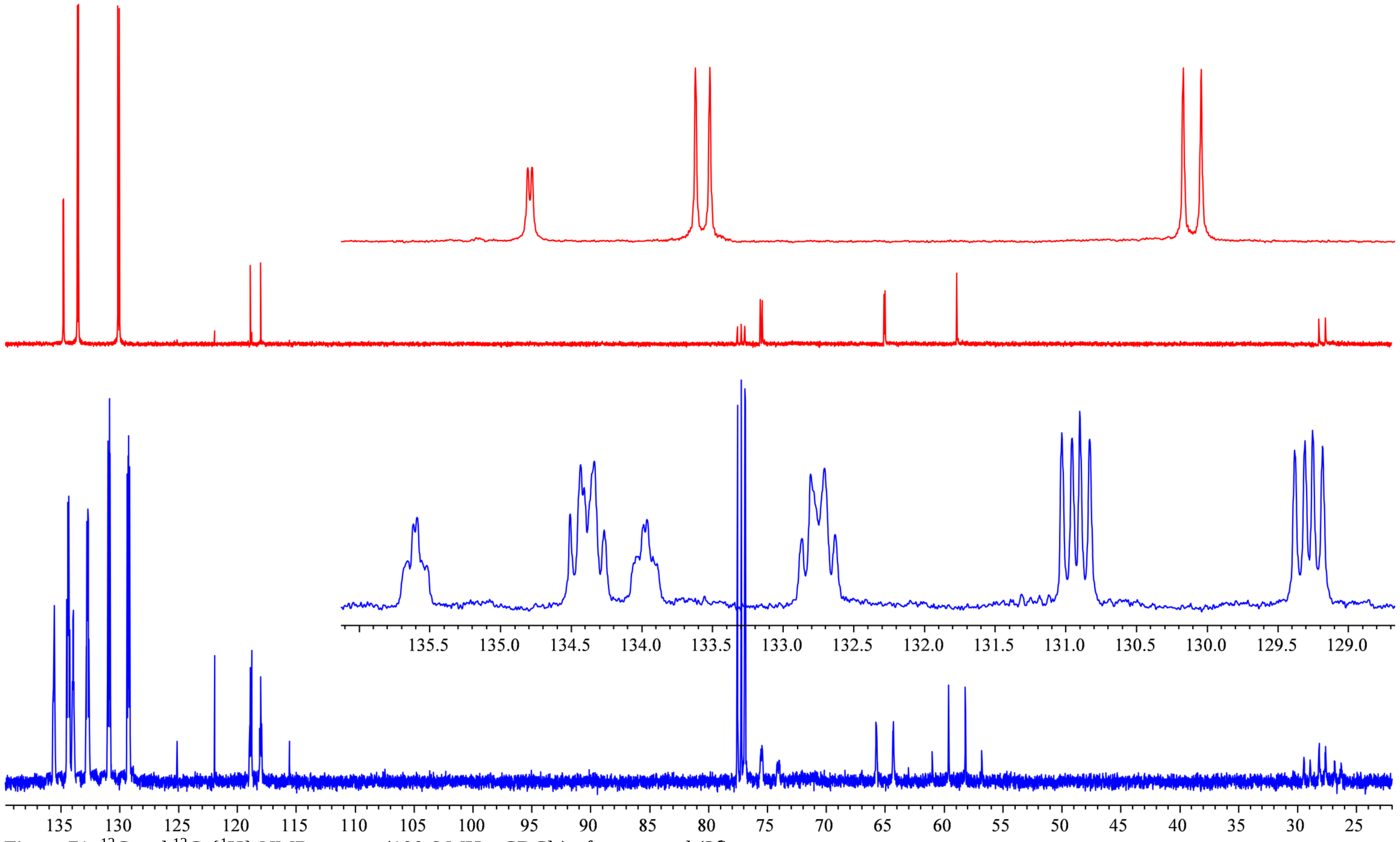

Figure 71. ^{13}C and ^{13}C-{^{1}H} NMR spectra (100.6 MHz, $CDCl_3$) of compound (**3f**).

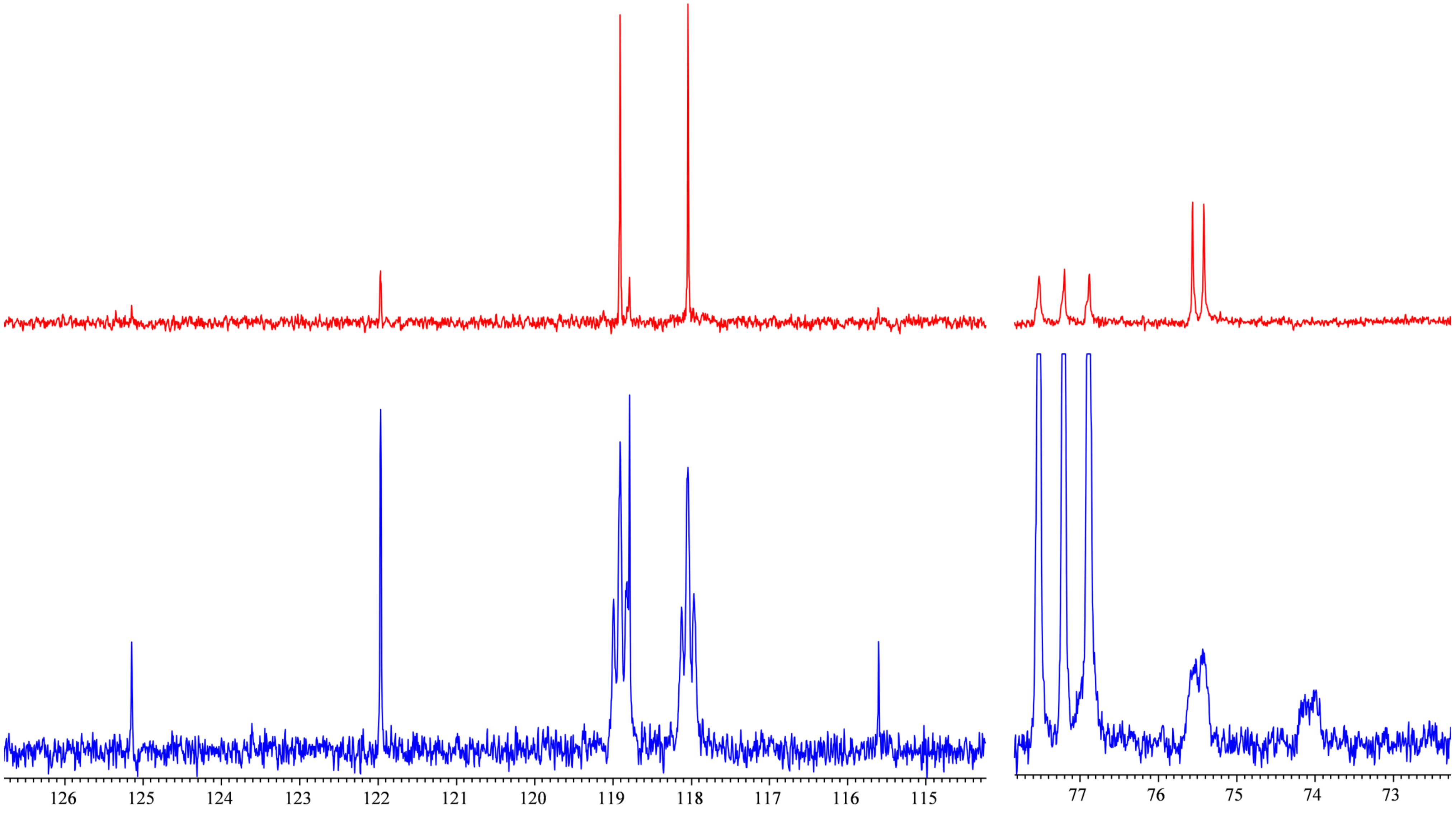

Figure 72. The 72-78 and 114-127 ppm regions of $^{13}C-\{^{1}H\}$ and ^{13}C NMR spectra (100.6 MHz, $CDCl_3$) of compound (**3f**).

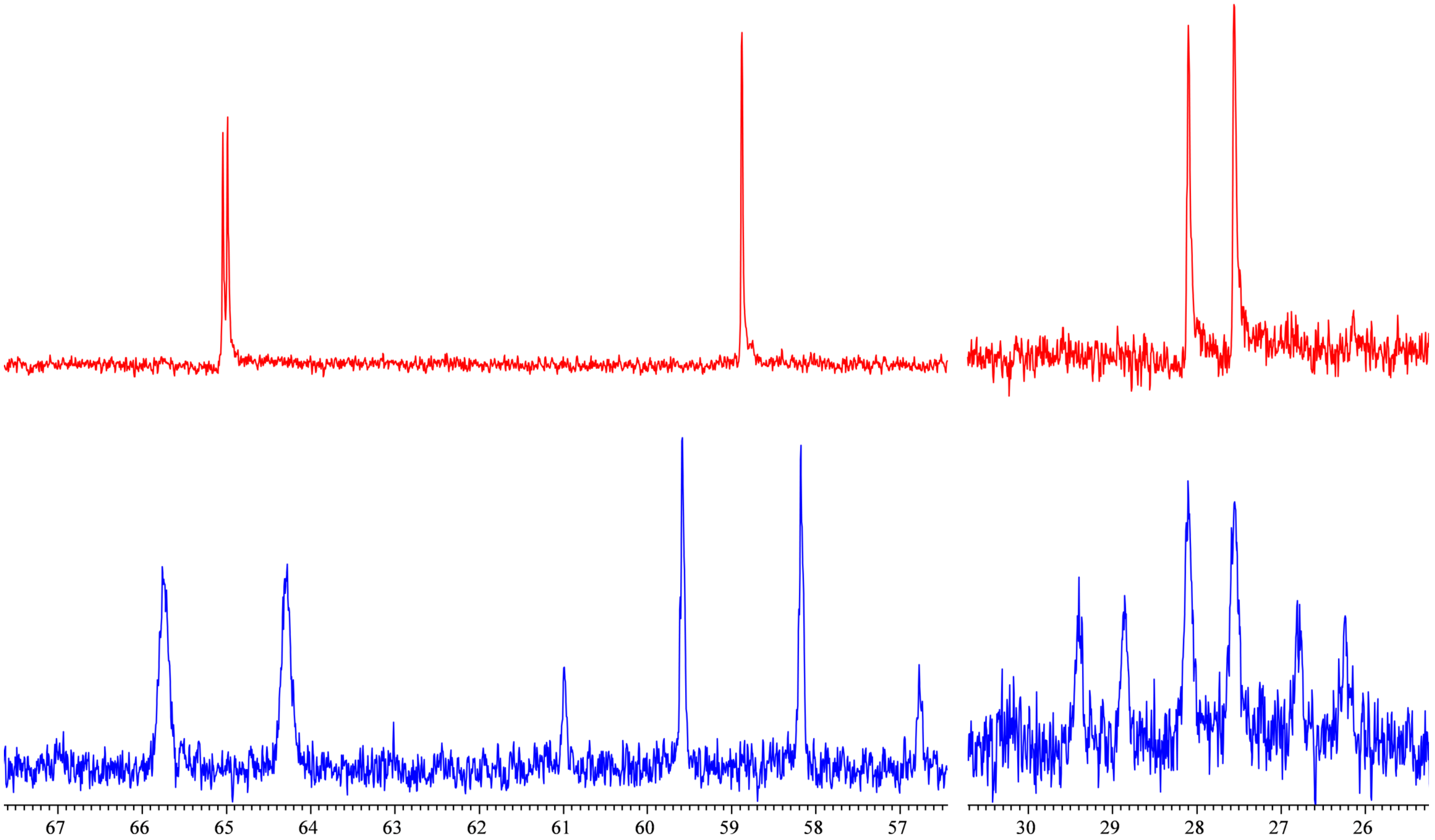

Figure 73. The 25-31 and 56-68 ppm regions of ^{13}C-{^{1}H} and ^{13}C NMR spectra (100.6 MHz, $CDCl_3$) of compound (**3f**).

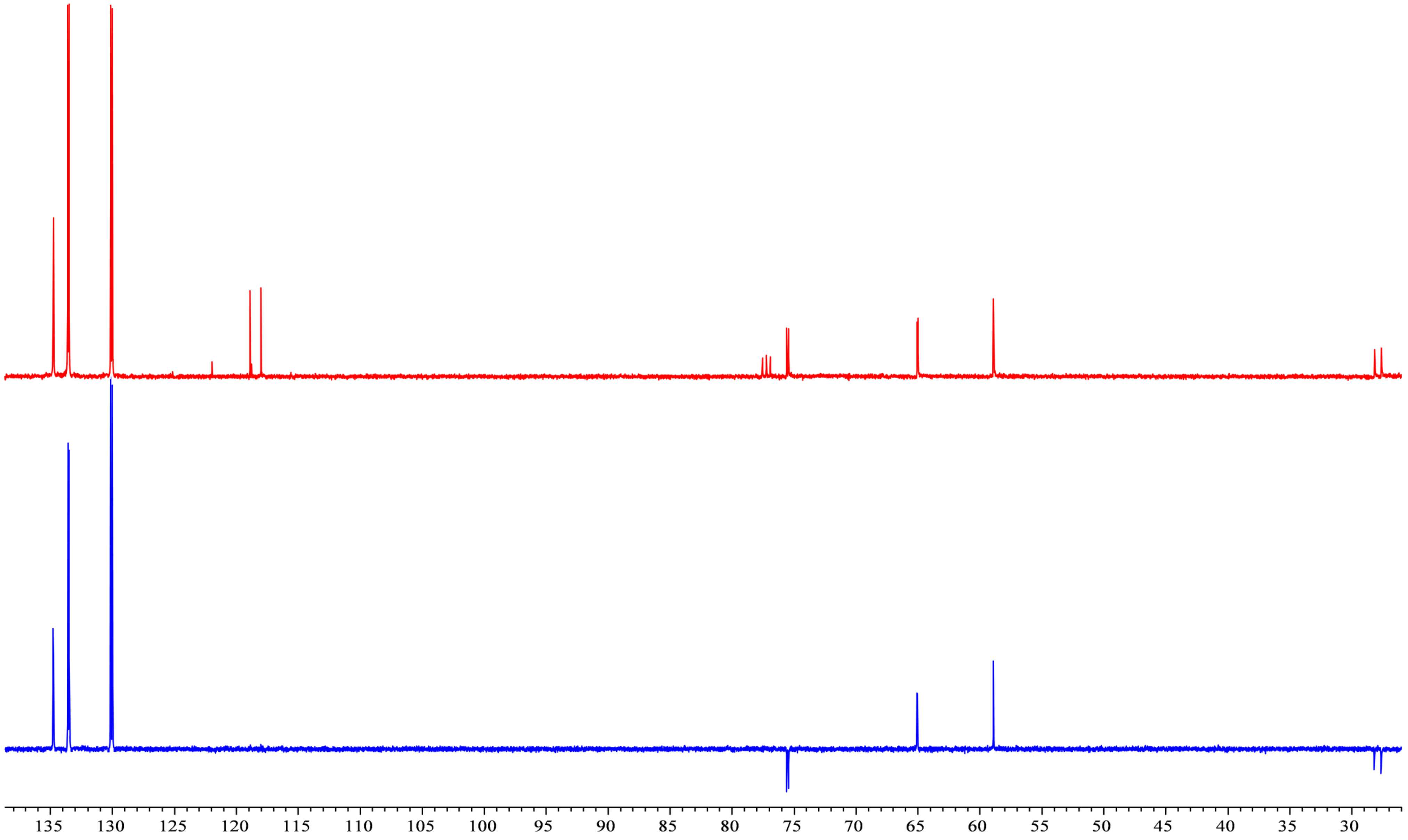

Figure 74. ^{13}C-{^{1}H} and ^{13}C-{^{1}H}-dept NMR spectra (100.6 MHz, $CDCl_3$) of compound (**3f**).

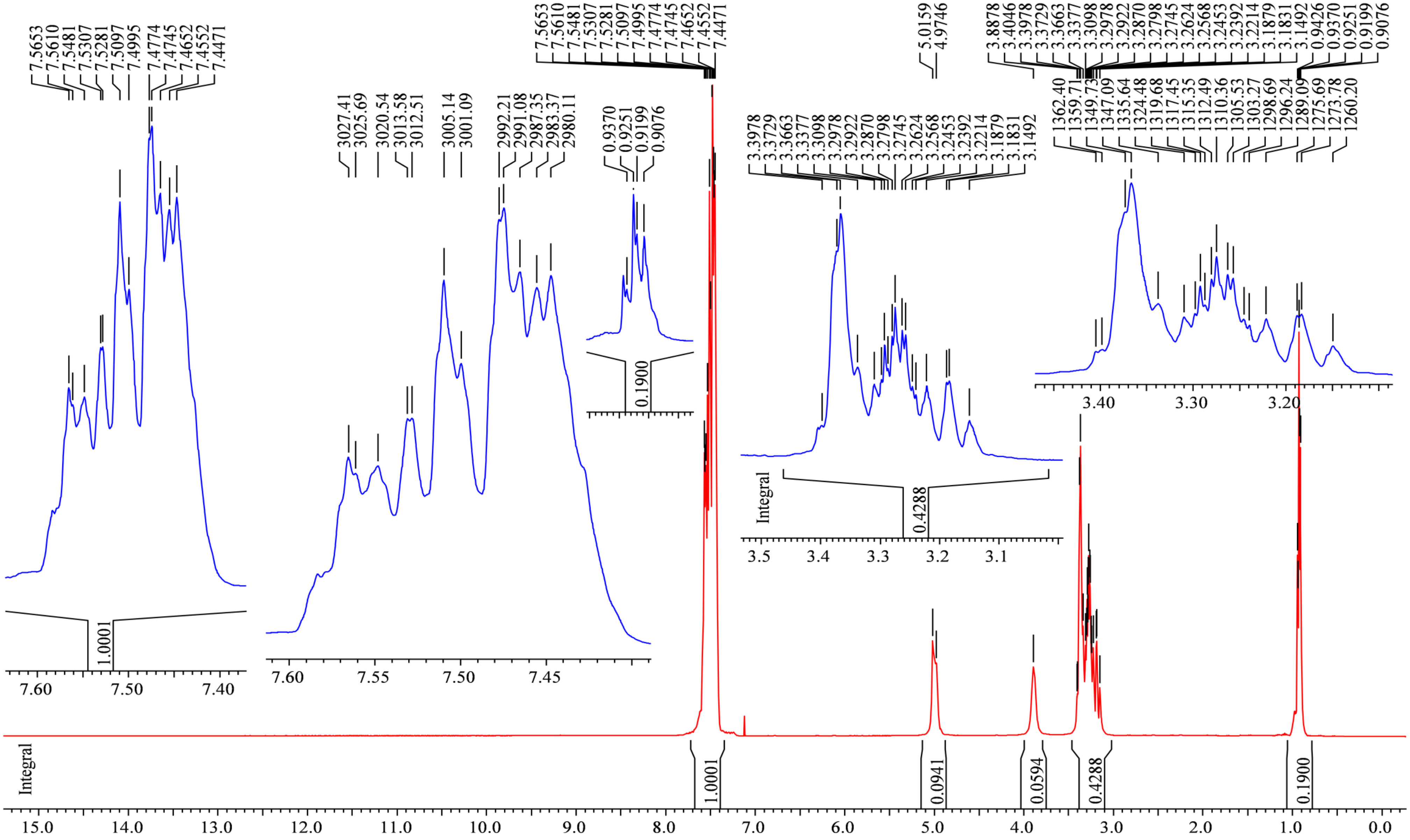

Figure 75. ^{1}H NMR spectrum (400 MHz, $CDCl_3$) of compound (**3g**).

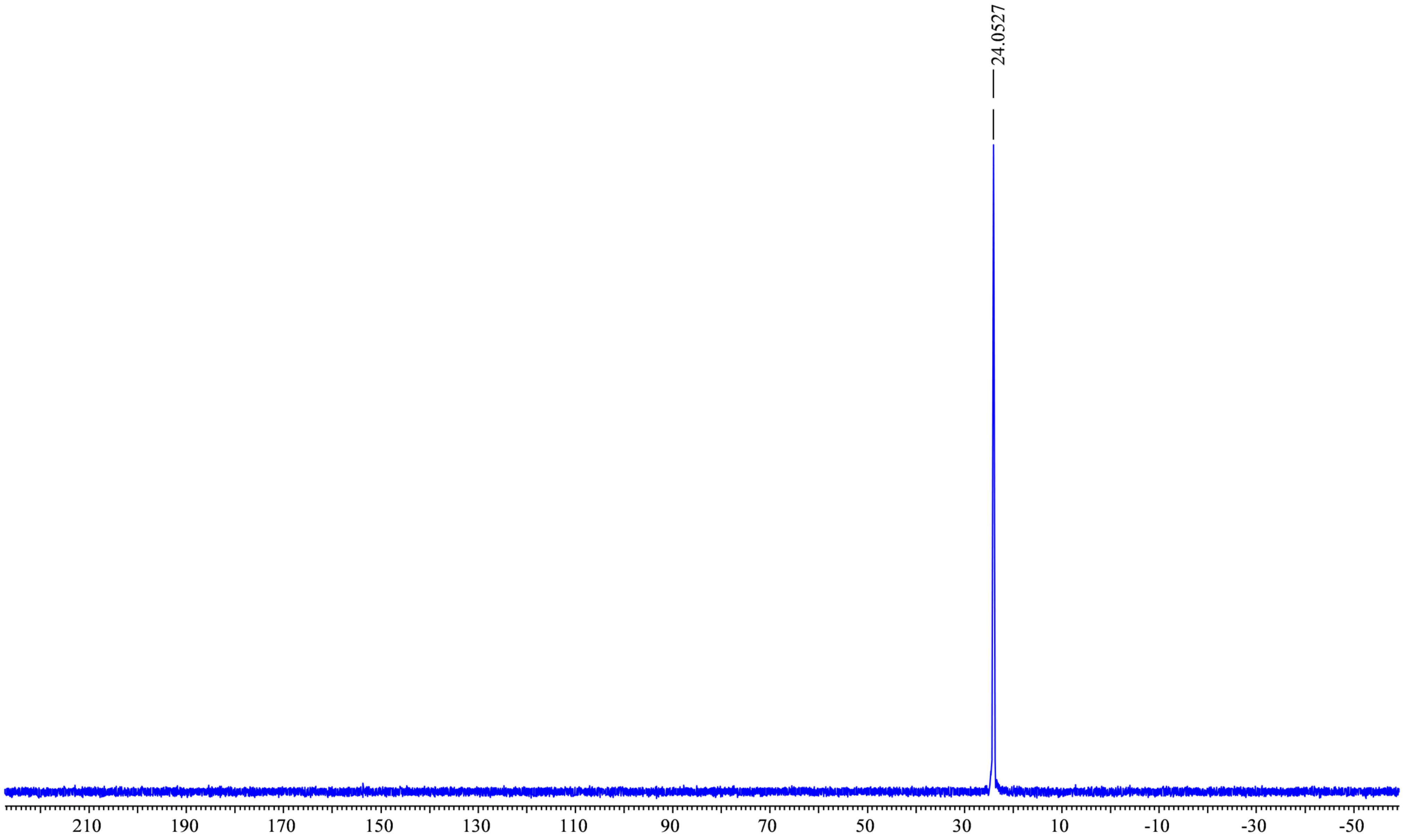

Figure 76. ^{31}P-{^{1}H} NMR spectrum (162.0 MHz, $CDCl_3$) of compound (**3g**).

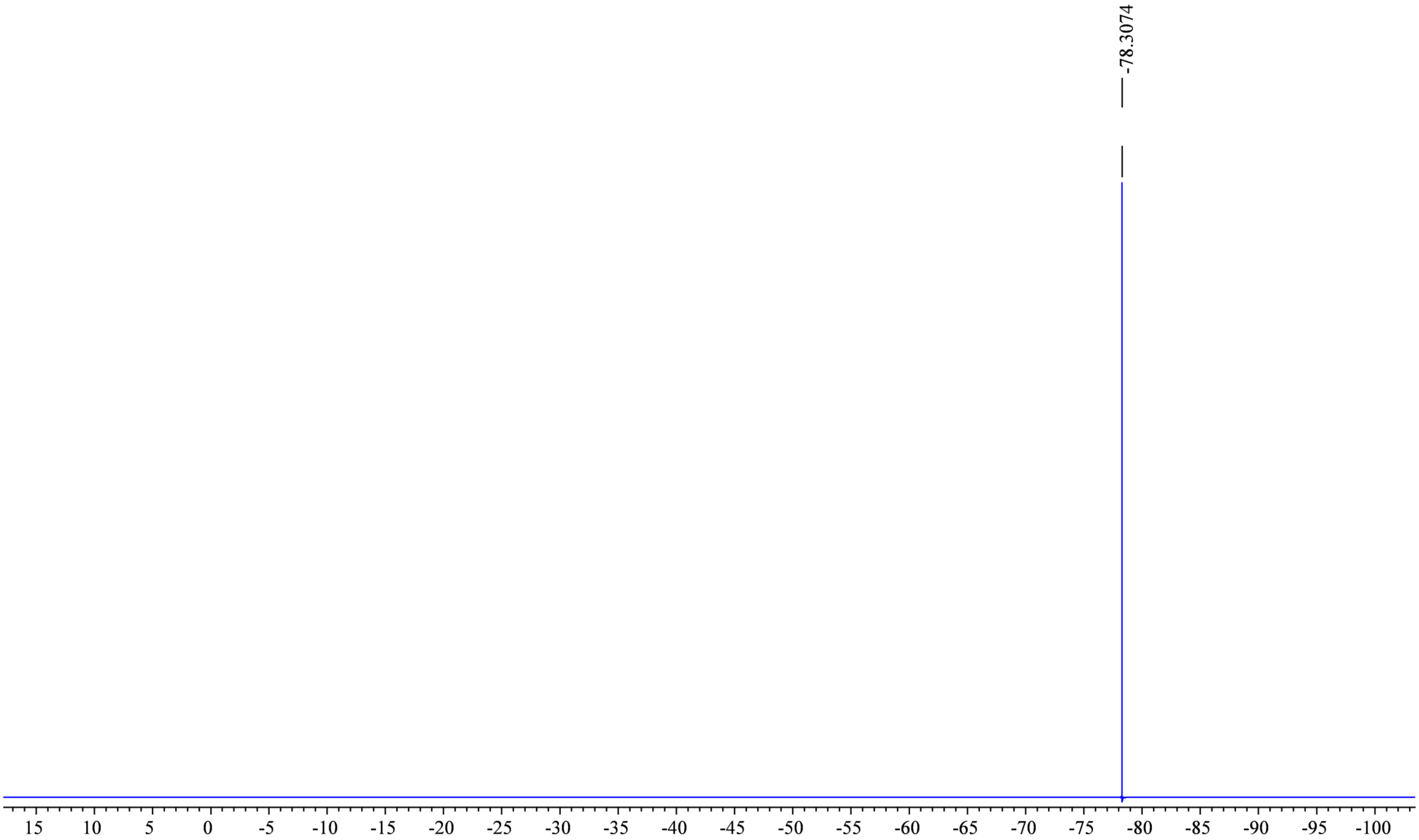

Figure 77. ^{19}F NMR spectrum (376.5 MHz, $CDCl_3$) of compound (**3g**).

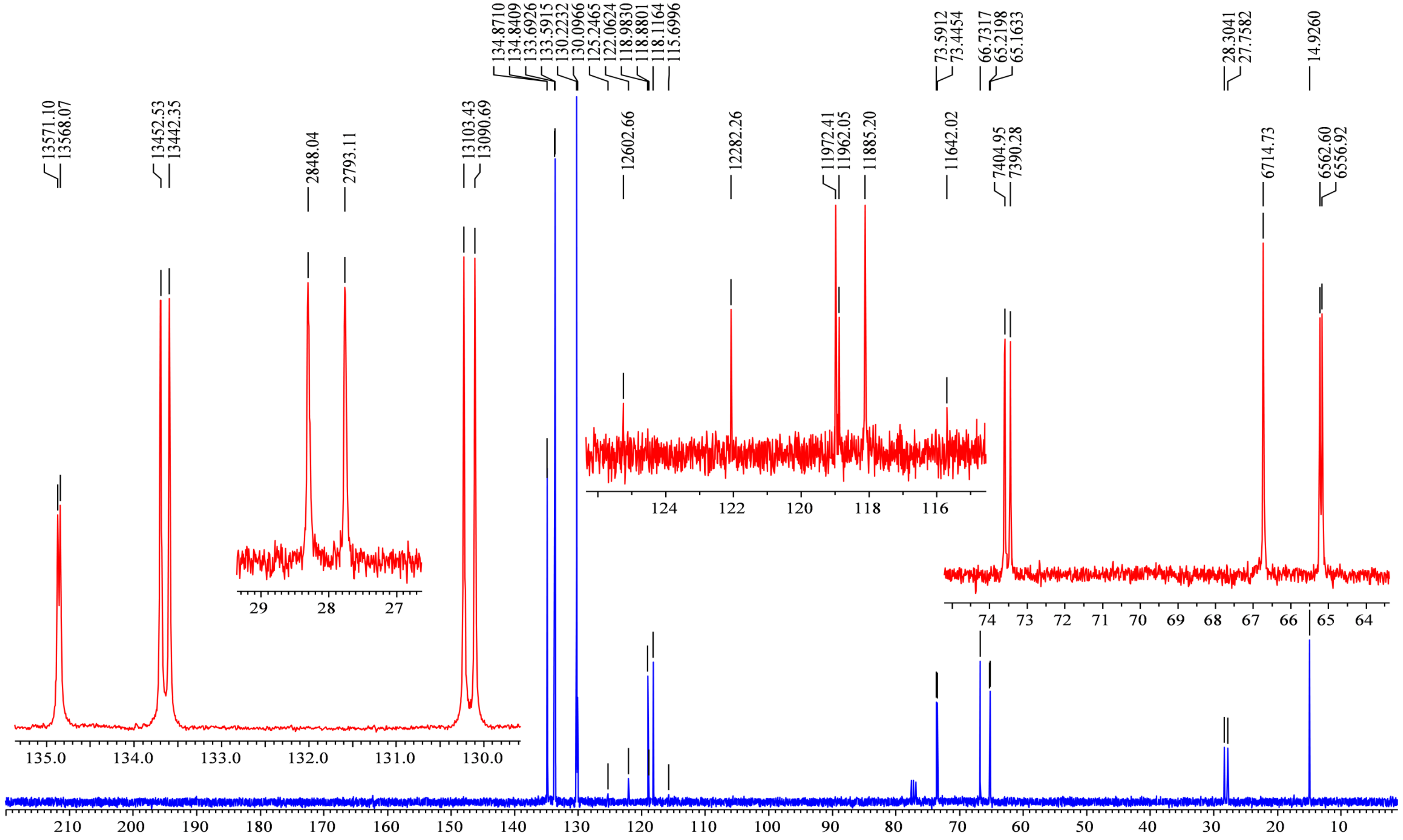

Figure 78. ^{13}C-{^{1}H} NMR spectrum (100.6 MHz, $CDCl_3$) of compound (**3g**).

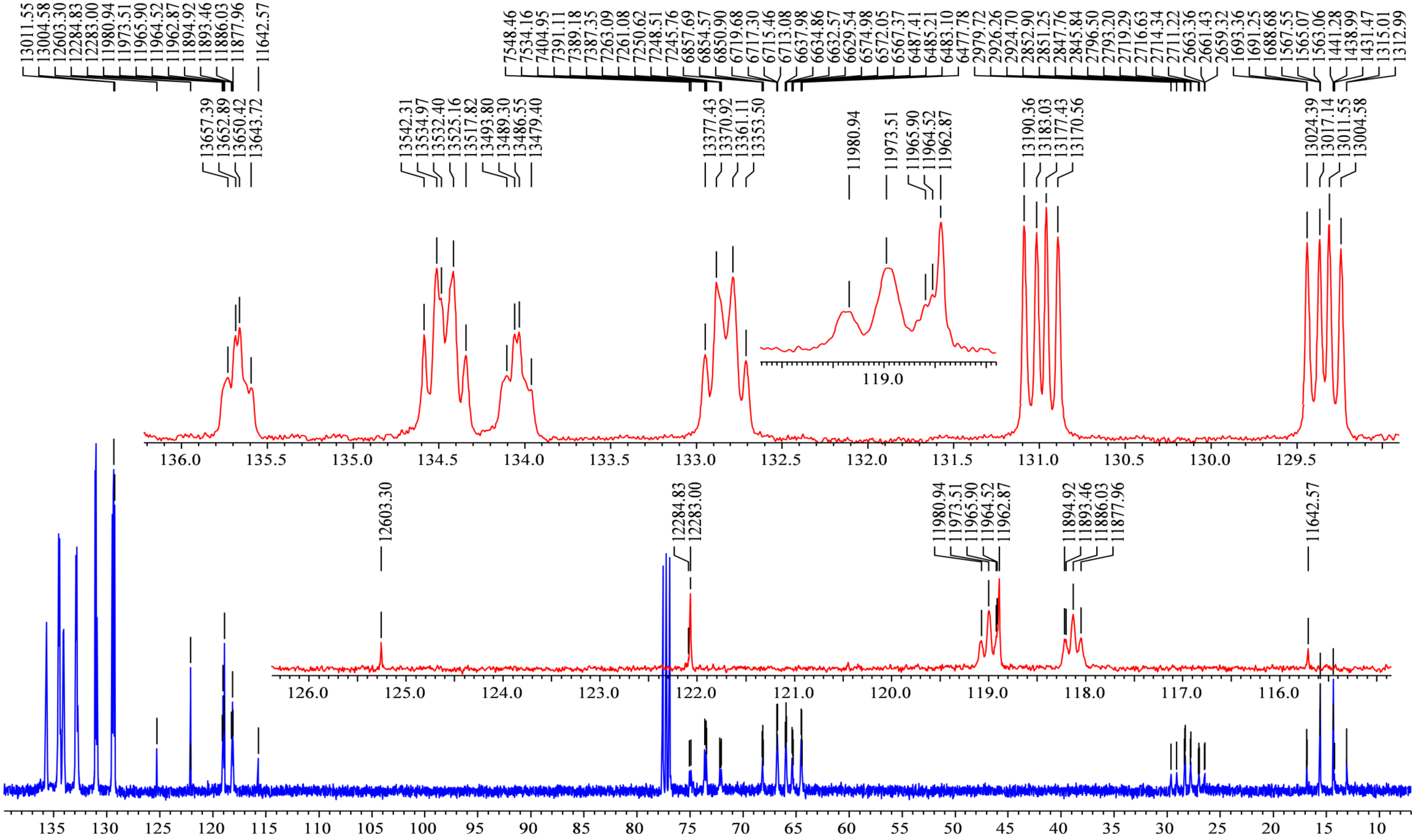

Figure 79. ^{13}C NMR spectrum (100.6 MHz, $CDCl_3$) of compound (**3g**).

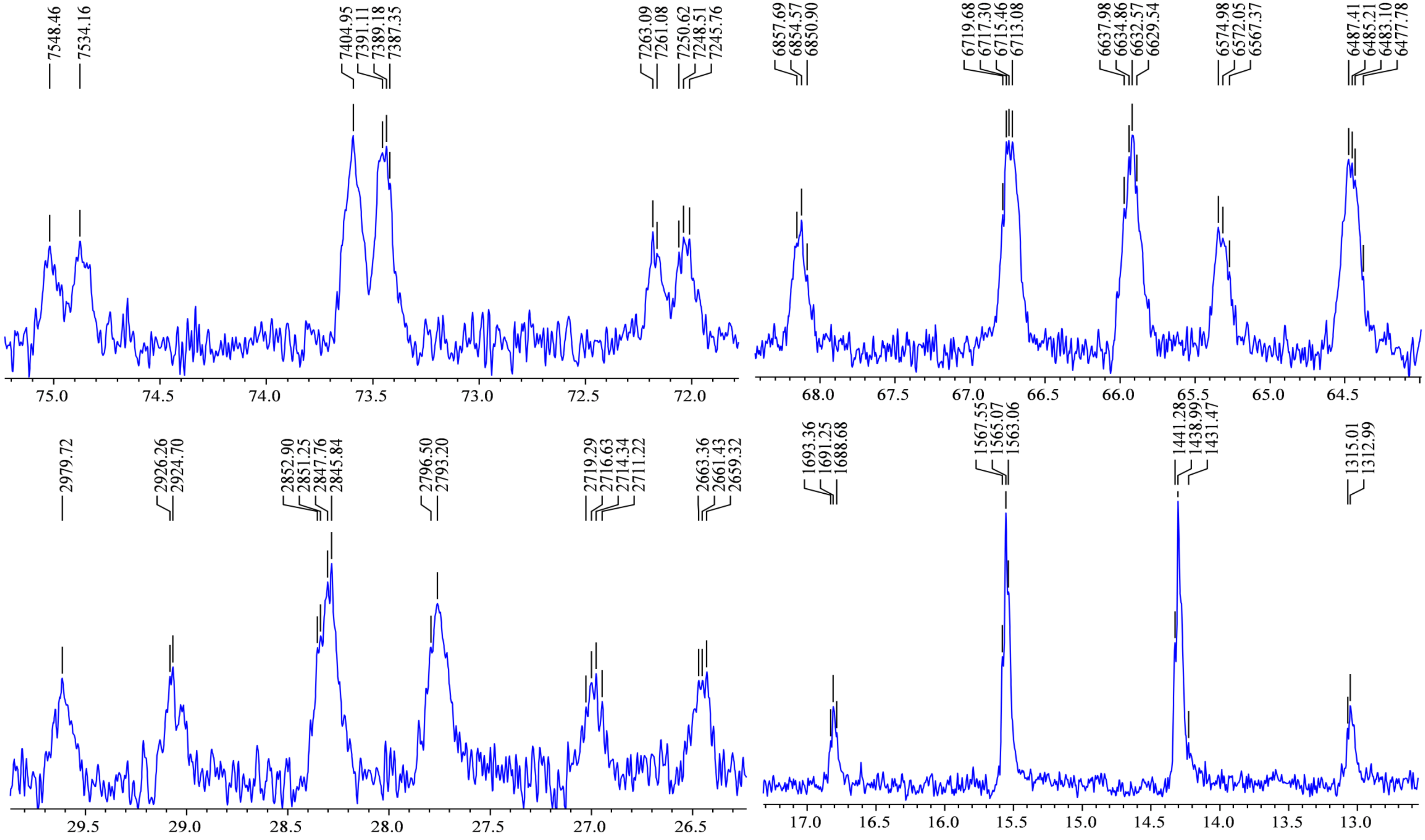

Figure 80. The 13-17, 26-30, 64-69 and 72-75 ppm regions of ^{13}C NMR spectrum (100.6 MHz, $CDCl_3$) of compound (**3g**).

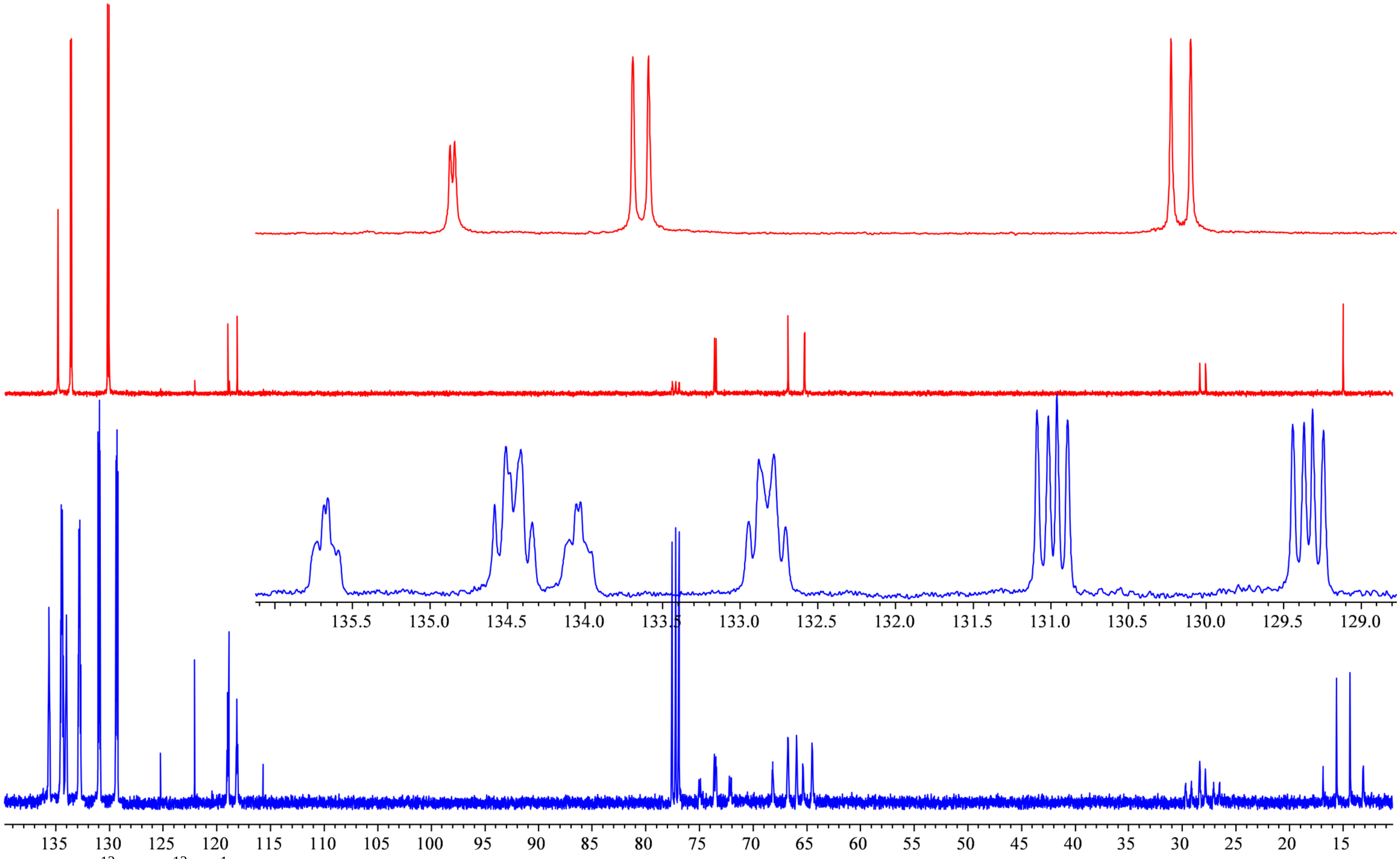

Figure 81. ^{13}C and ^{13}C-{^{1}H} NMR spectra (100.6 MHz, $CDCl_3$) of compound (**3g**).

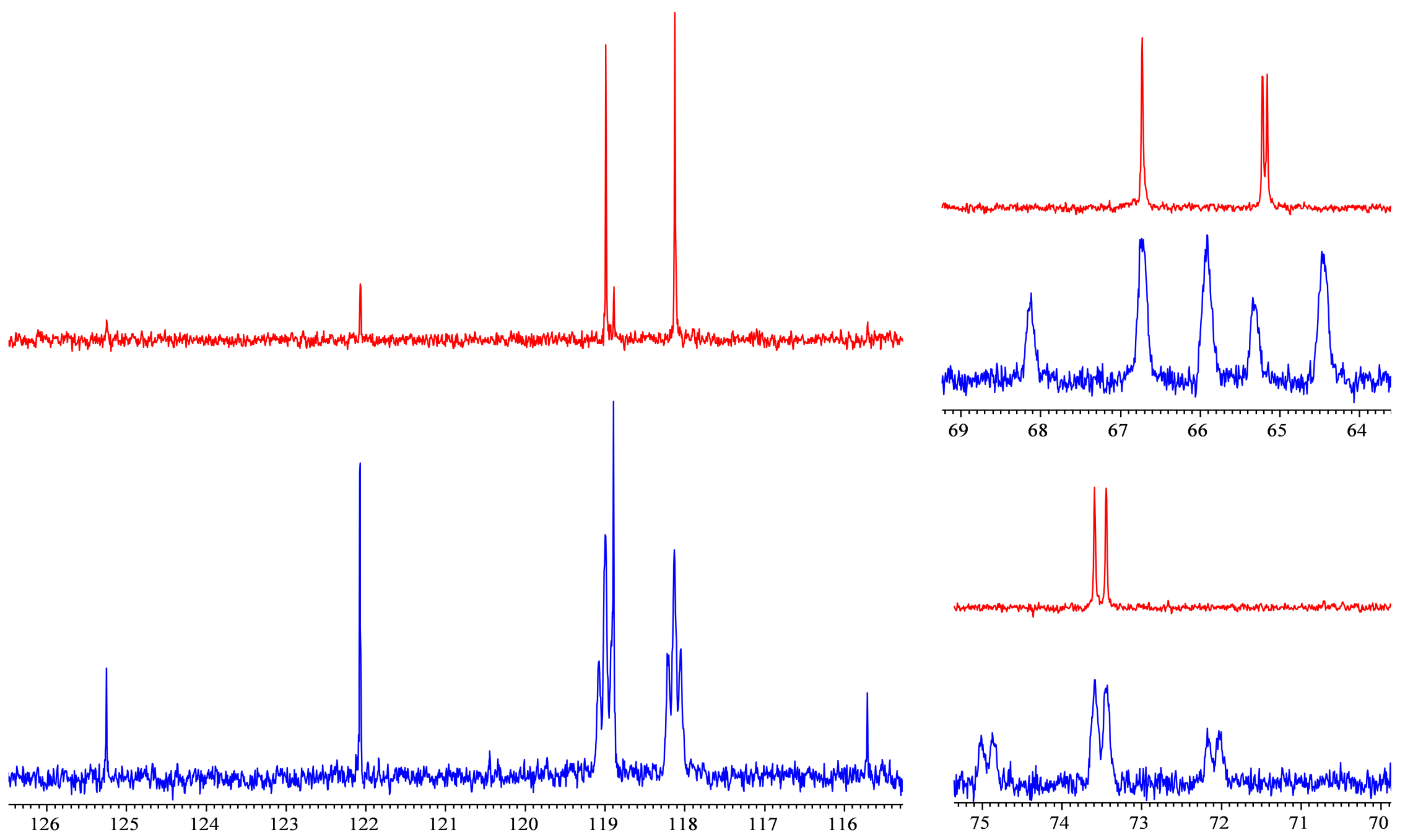

Figure 82. The 64-69, 70-75 and 115-127 ppm regions of ^{13}C-{^{1}H} and ^{13}C NMR spectra (100.6 MHz, $CDCl_3$) of compound (**3g**).

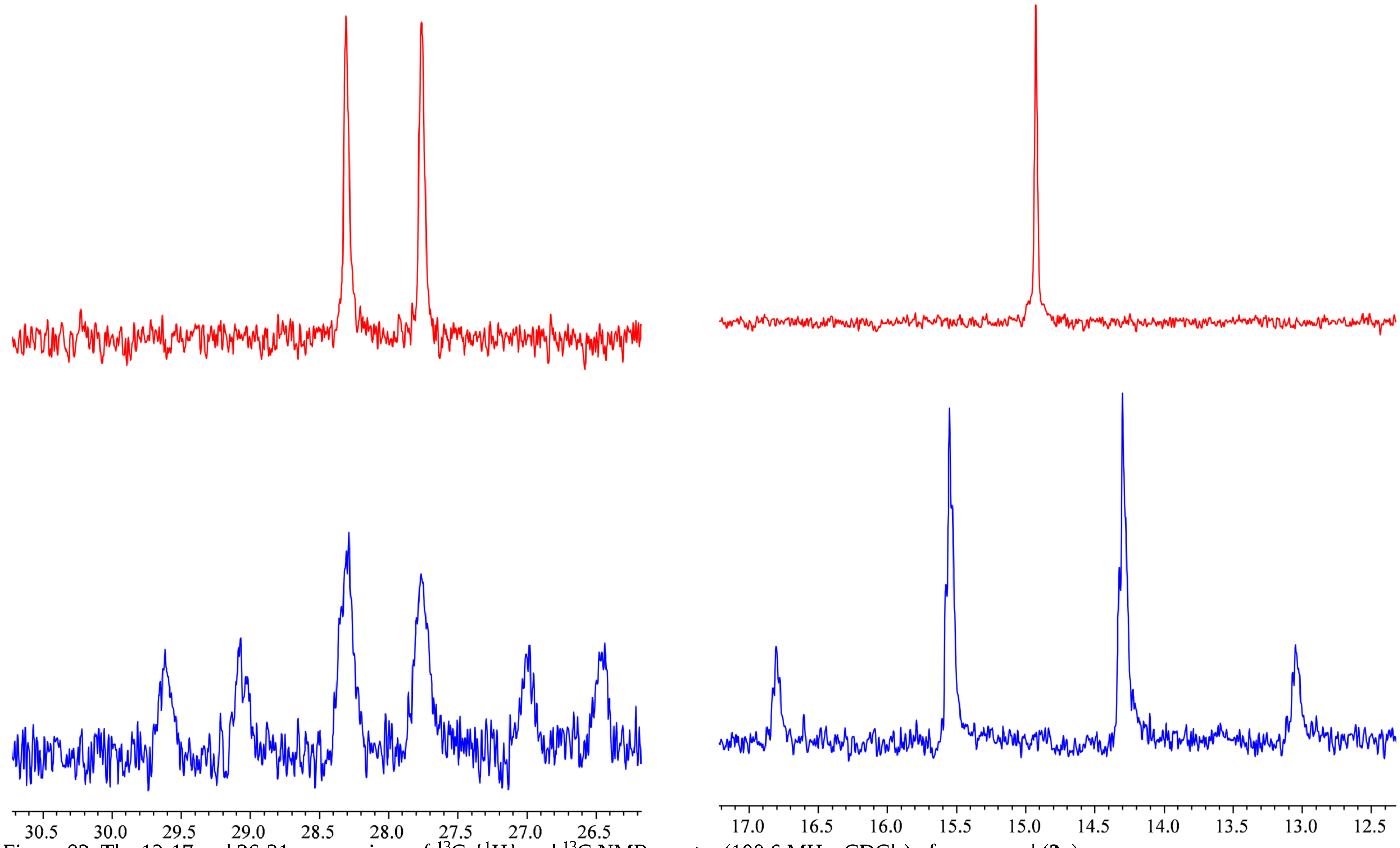

Figure 83. The 12-17 and 26-31 ppm regions of ^{13}C-{^{1}H} and ^{13}C NMR spectra (100.6 MHz, $CDCl_3$) of compound (**3g**).

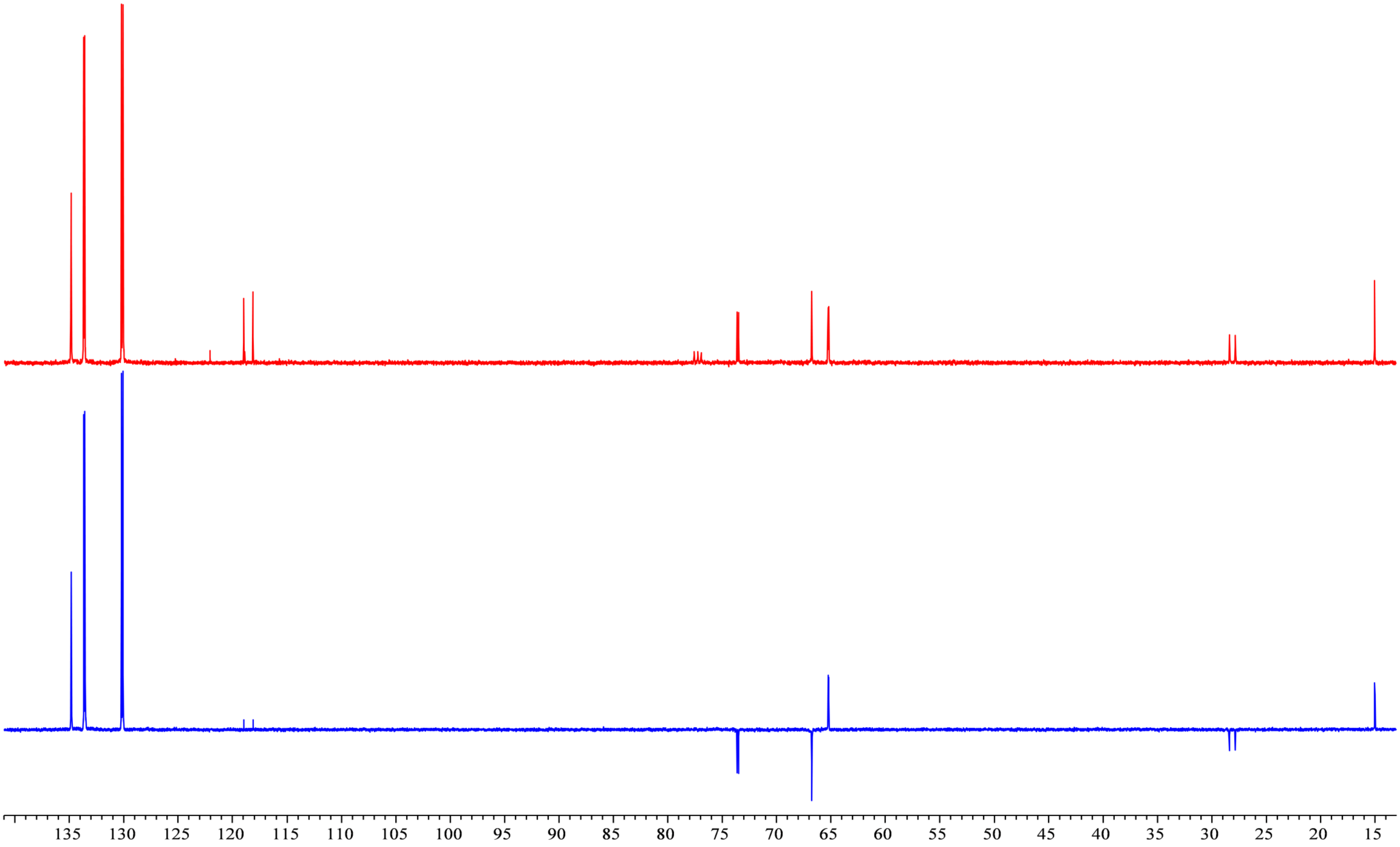

Figure 84. ^{13}C-{^{1}H} and ^{13}C-{^{1}H}-dept NMR spectra (100.6 MHz, $CDCl_3$) of compound (**3g**).

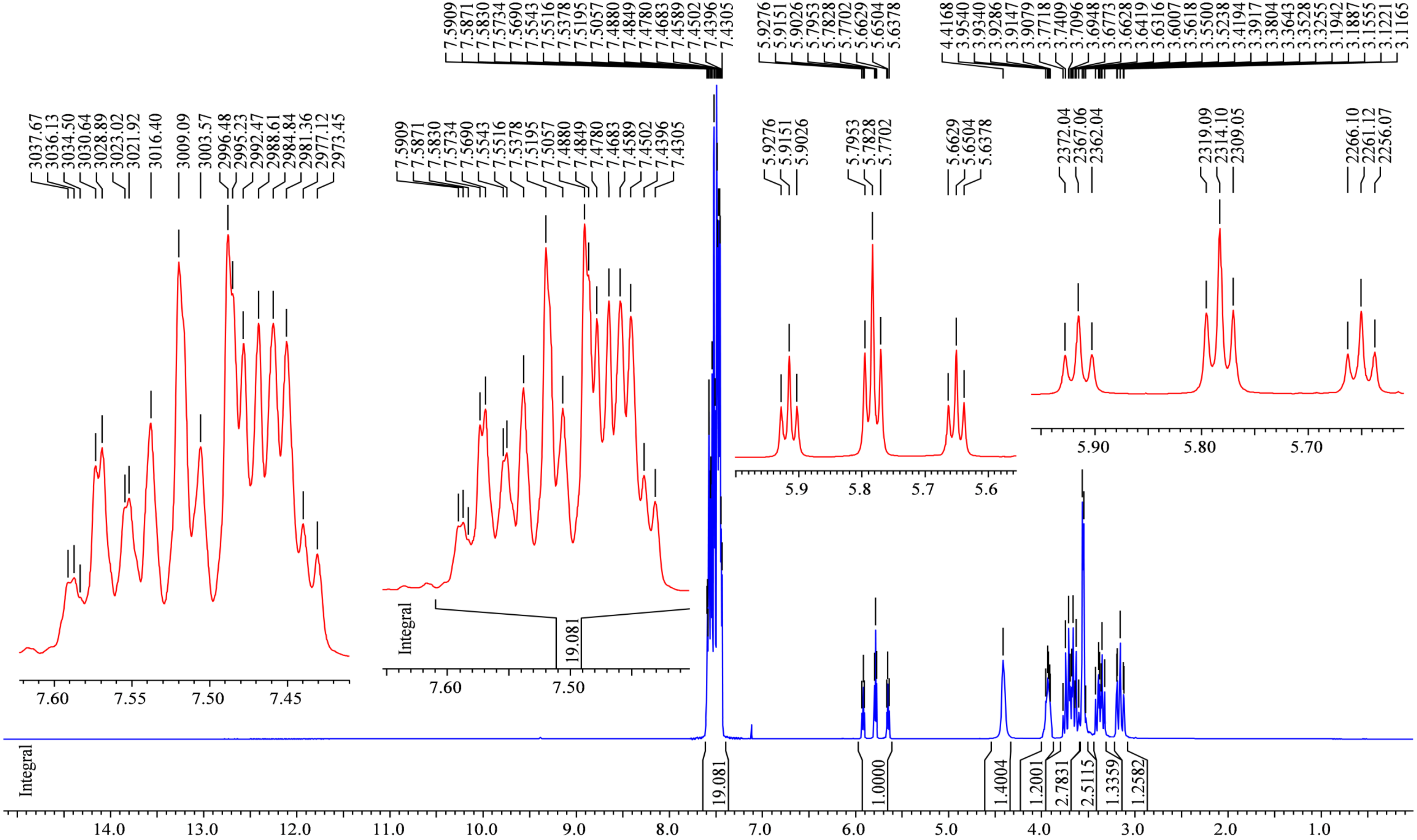

Figure 85. ^{1}H NMR spectrum (400 MHz, $CDCl_3$) of compound (**3h**).

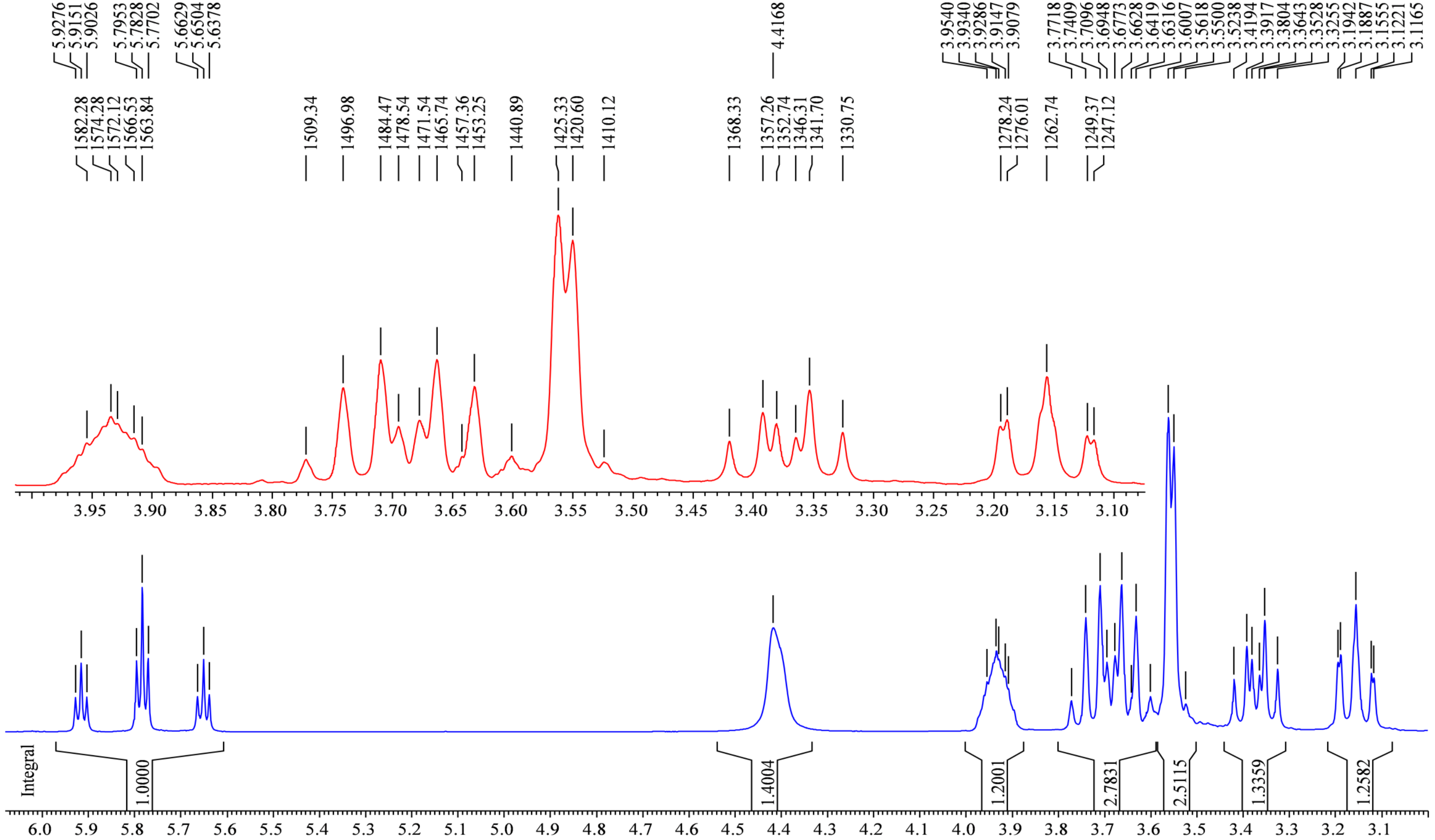

Figure 86. The 3-6 ppm region of ^{1}H NMR spectrum (400 MHz, $CDCl_3$) of compound (**3h**).

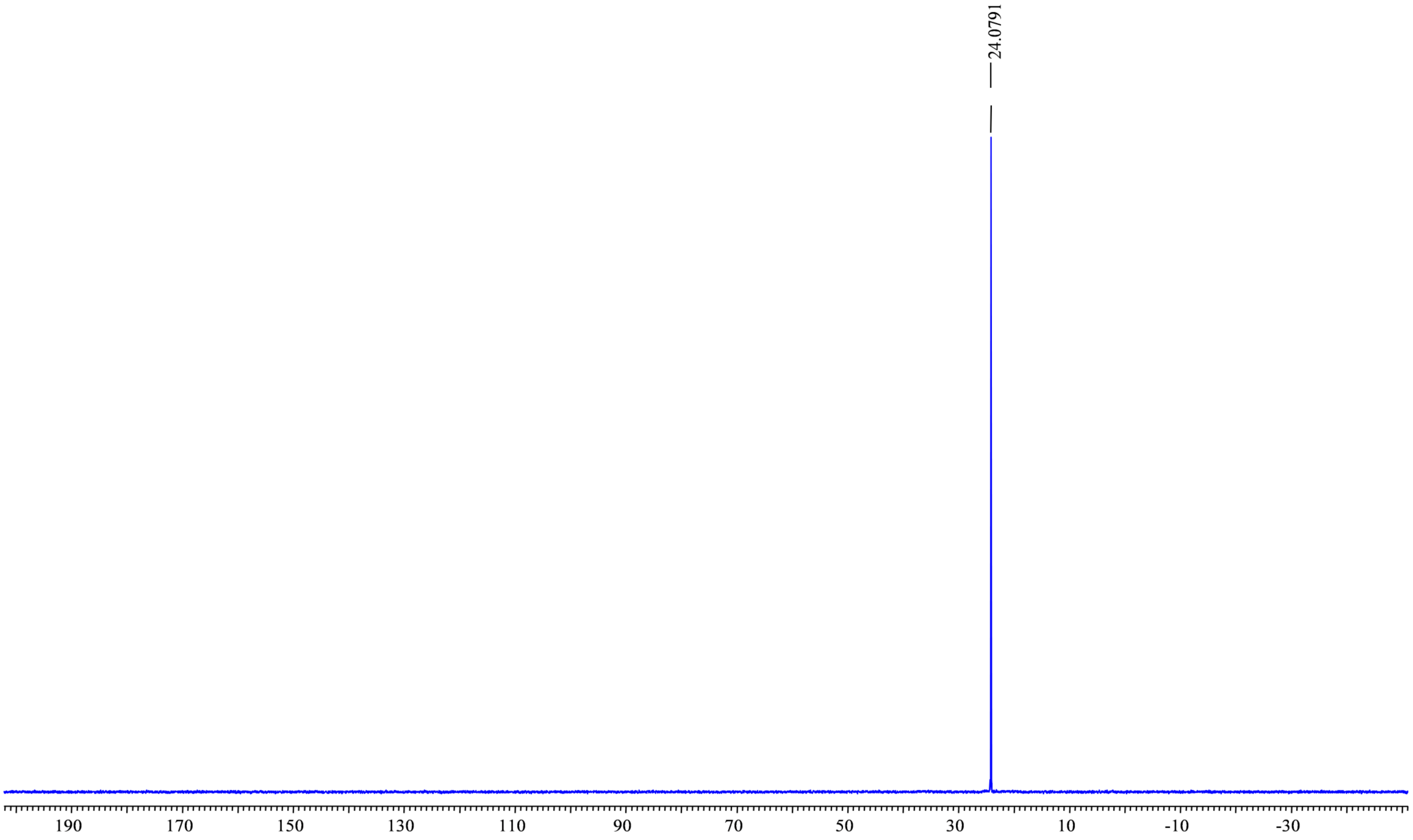

Figure 87. ^{31}P-{^{1}H} NMR spectrum (162.0 MHz, CH_3CN) of compound (**3h**).

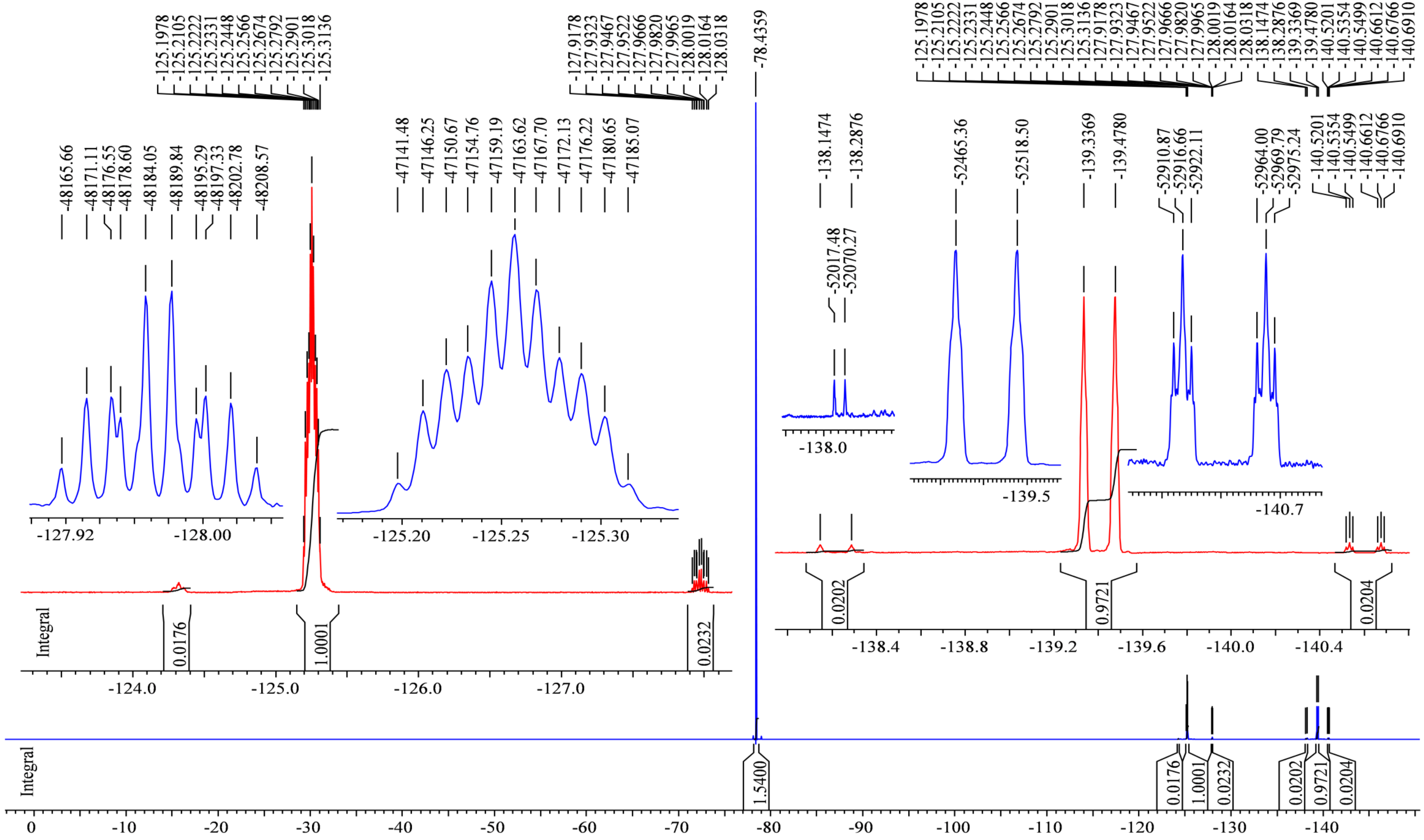

Figure 88. ^{19}F NMR spectrum (376.5 MHz, $CDCl_3$) of compound (**3h**).

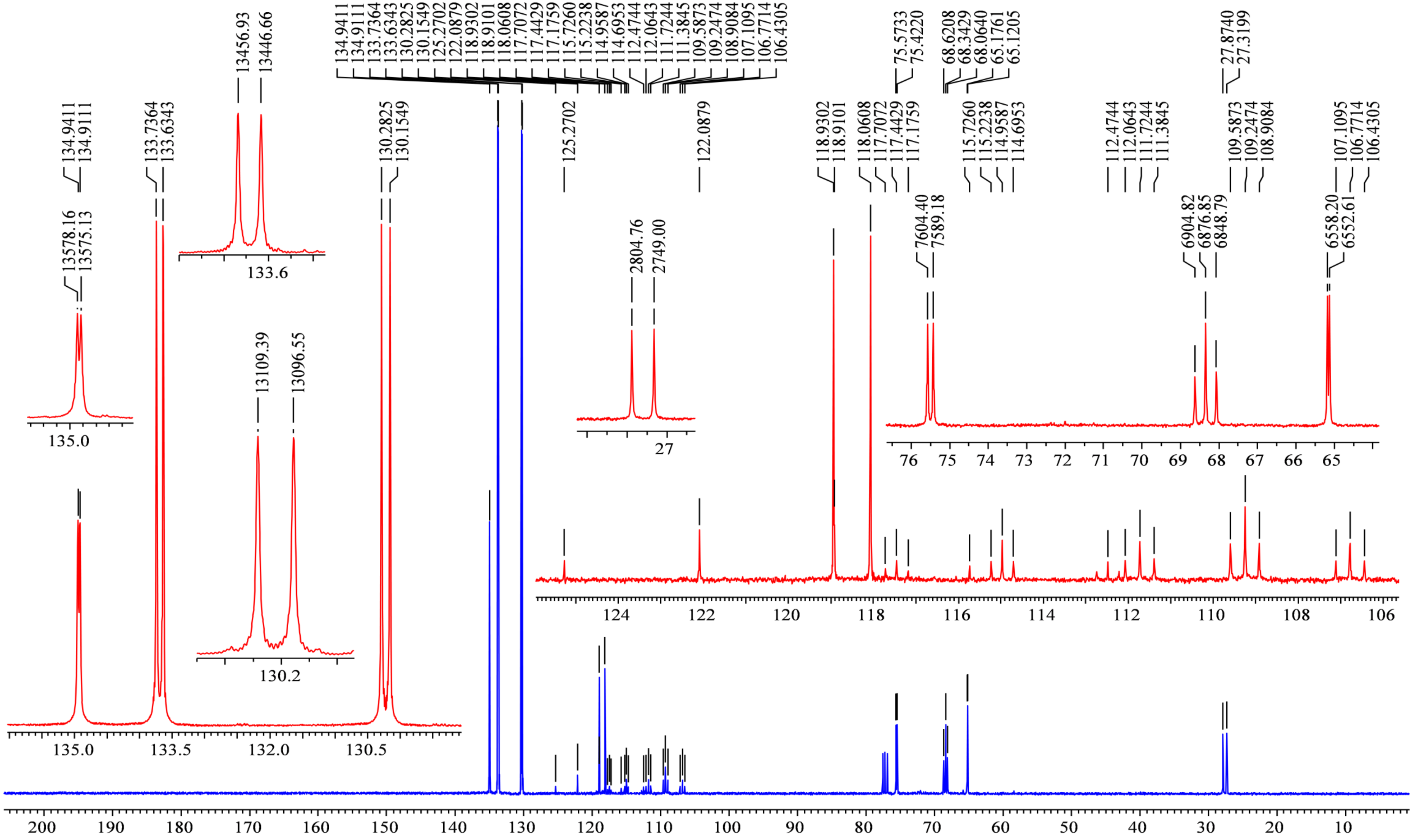

Figure 89. ^{13}C-{^{1}H} NMR spectrum (100.6 MHz, $CDCl_3$) of compound (**3h**).

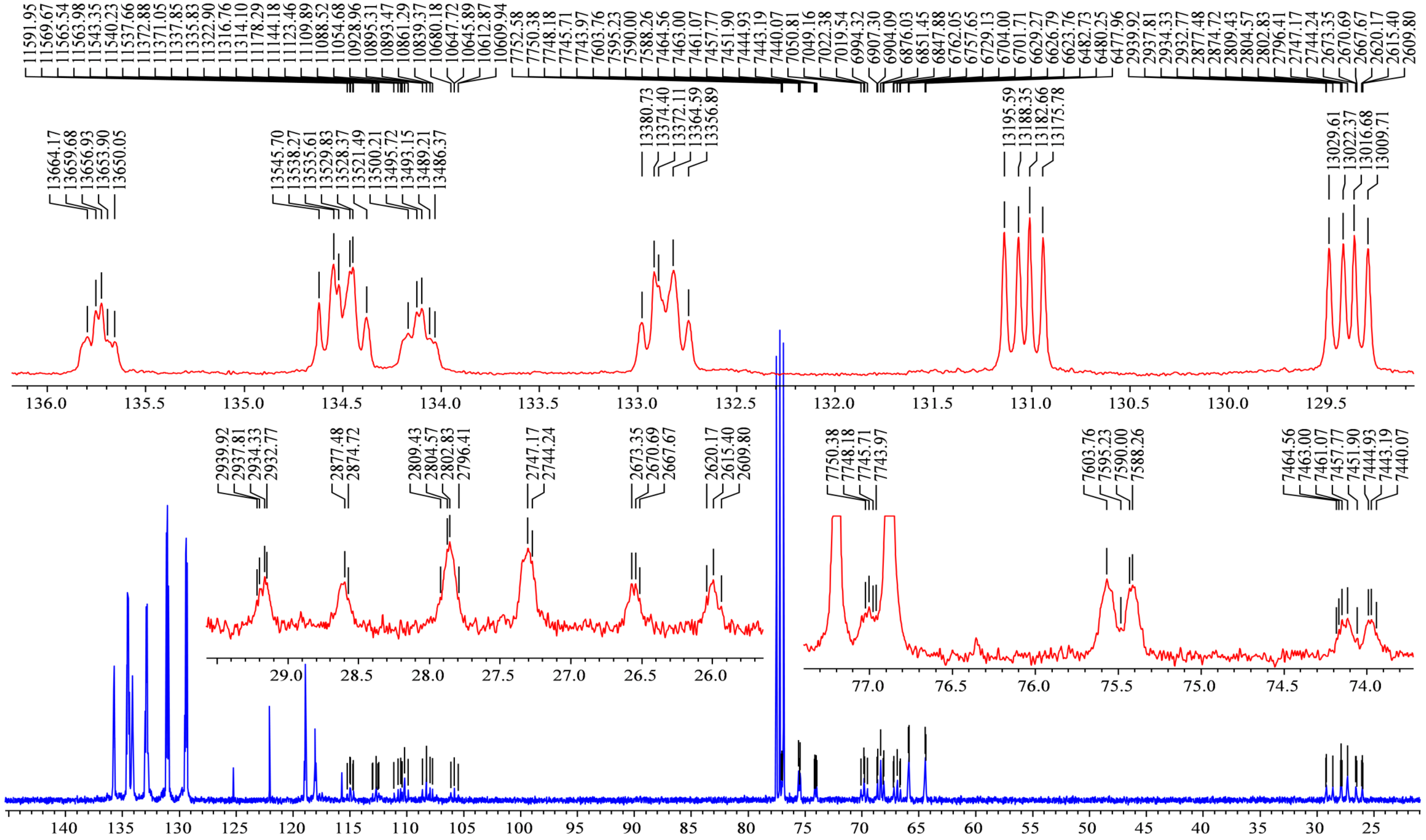

Figure 90. ^{13}C NMR spectrum (100.6 MHz, $CDCl_3$) of compound (**3h**).

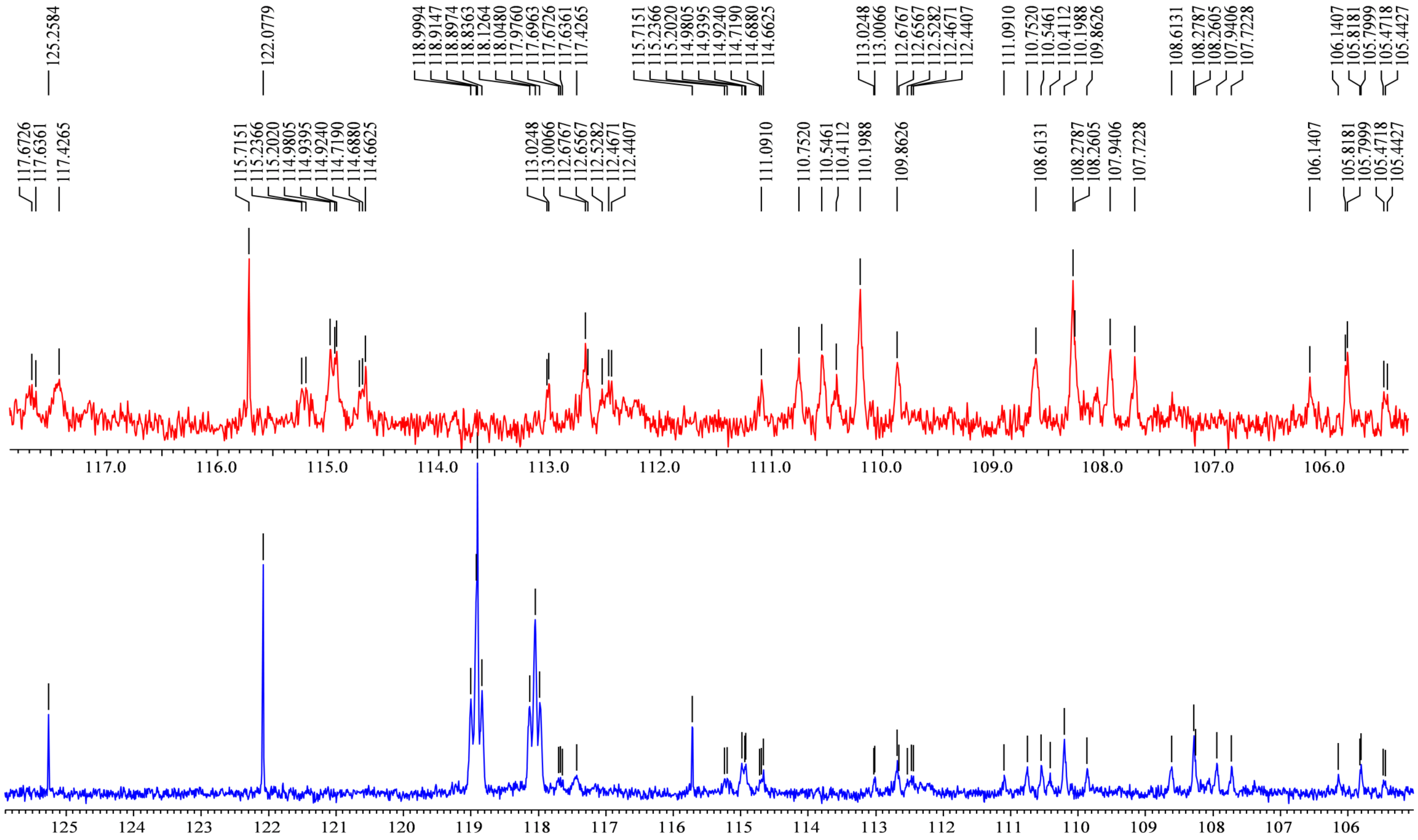

Figure 91. The 105-126 ppm region of ^{13}C NMR spectrum (100.6 MHz, $CDCl_3$) of compound (**3h**).

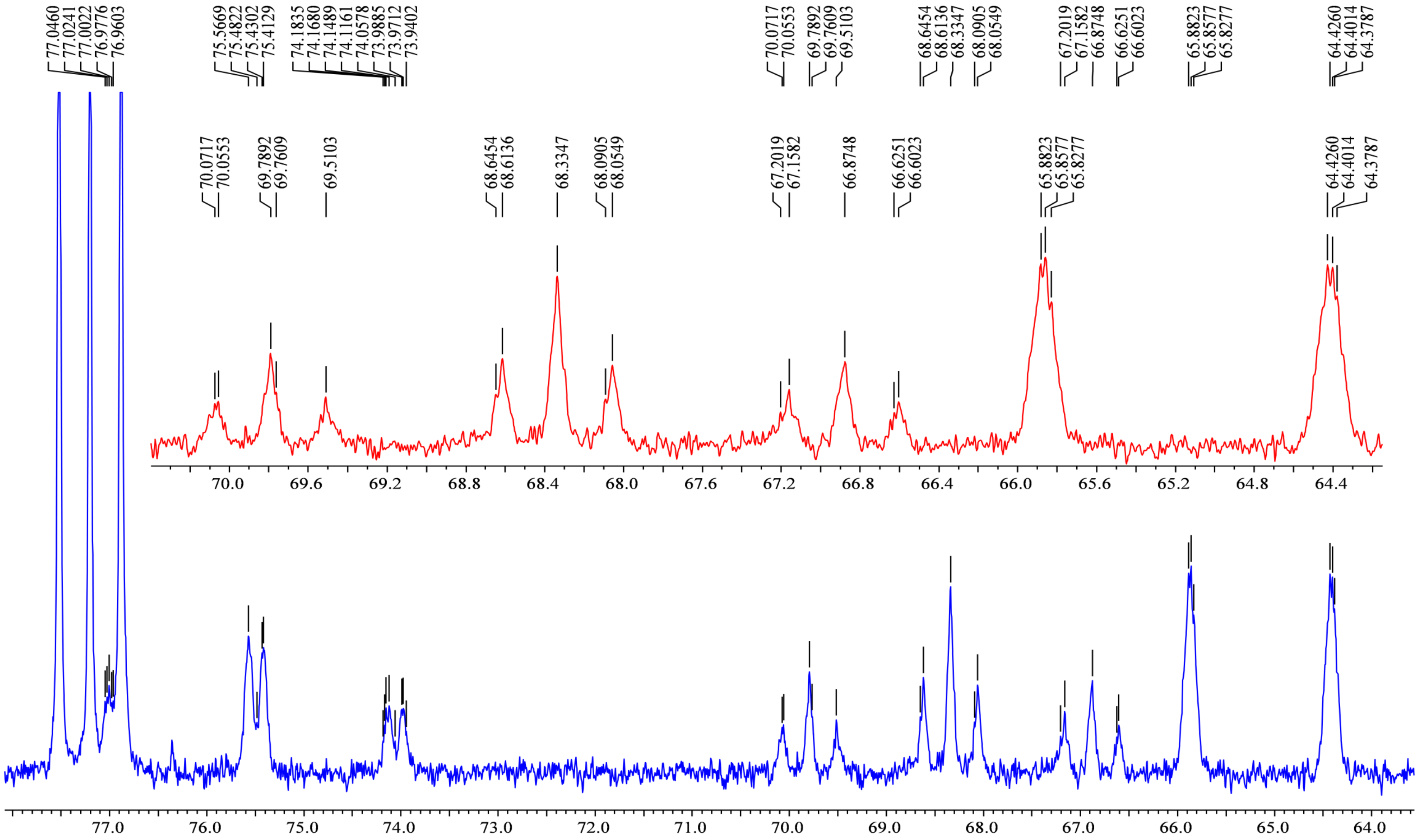

Figure 92. The 63-79 ppm region of ^{13}C NMR spectrum (100.6 MHz, $CDCl_3$) of compound (**3h**).

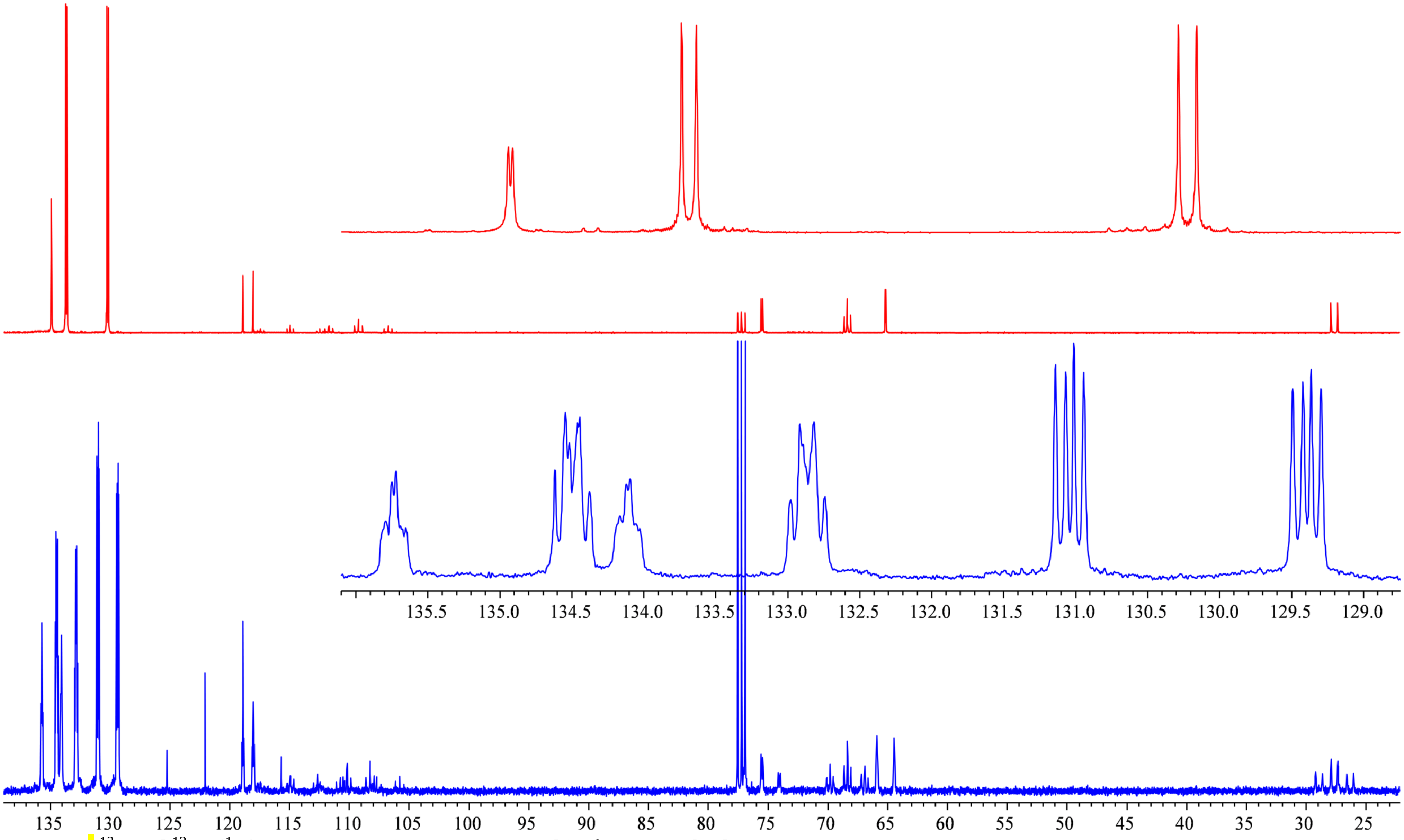

Figure 93. ^{13}C and ^{13}C -{^{1}H} NMR spectra (100.6 MHz, $CDCl_3$) of compound (**3h**).

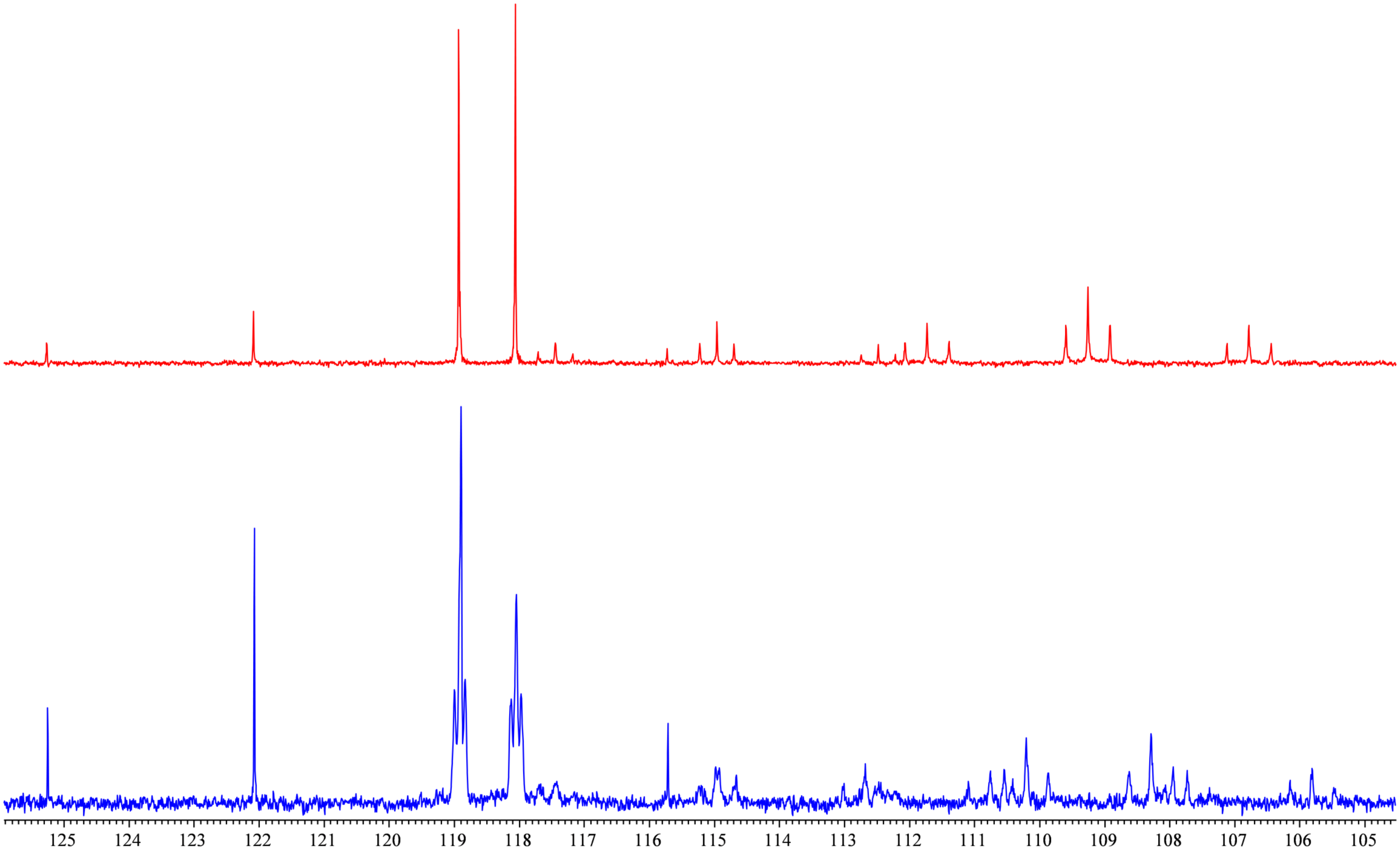

Figure 94. The 104-126 ppm region of ^{13}C-{^{1}H} and ^{13}C NMR spectra (100.6 MHz, $CDCl_3$) of compound (**3h**).

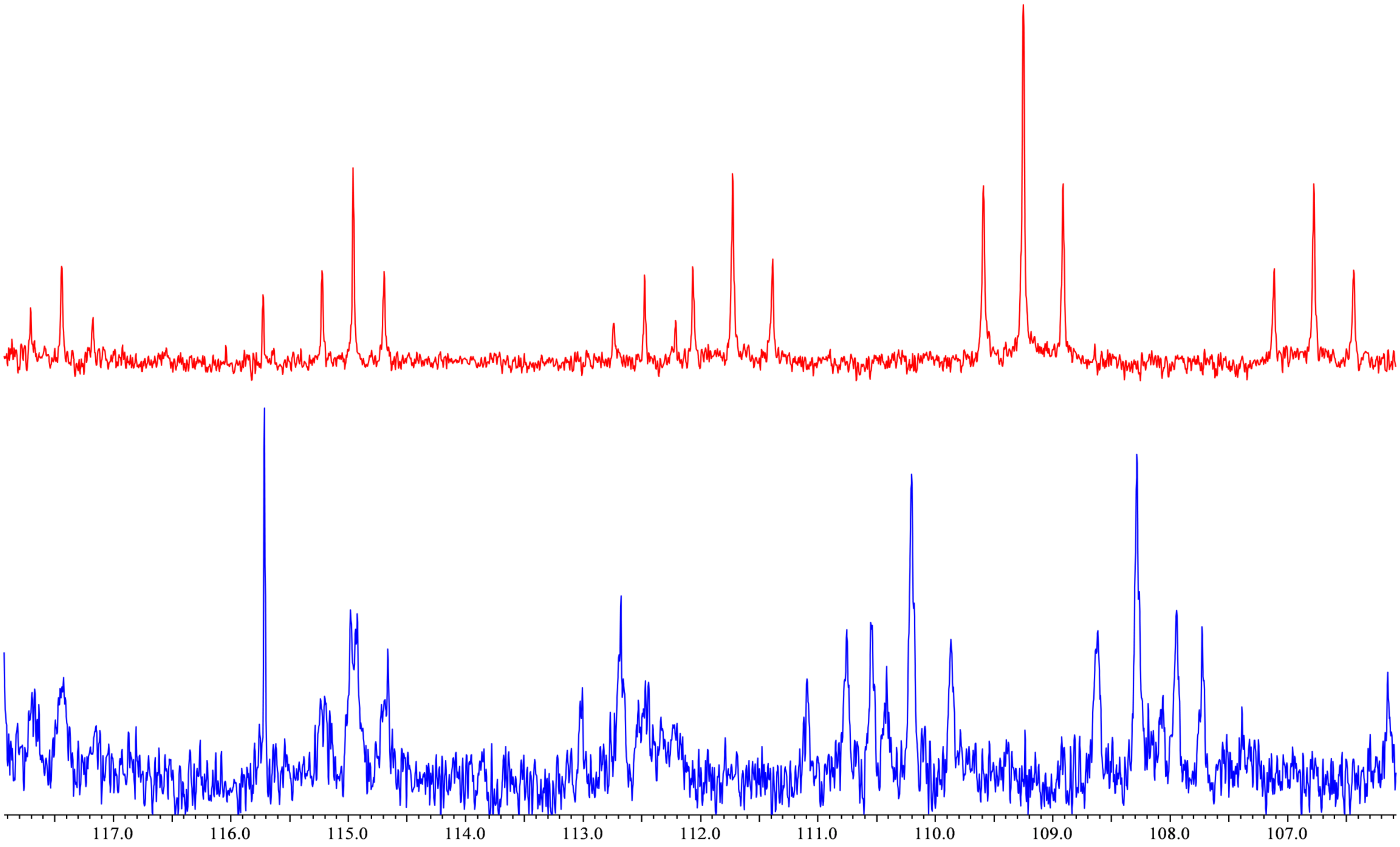

Figure 95. The 106-118 ppm region of ^{13}C-{^{1}H} and ^{13}C NMR spectrum (100.6 MHz, $CDCl_3$) of compound (**3h**).

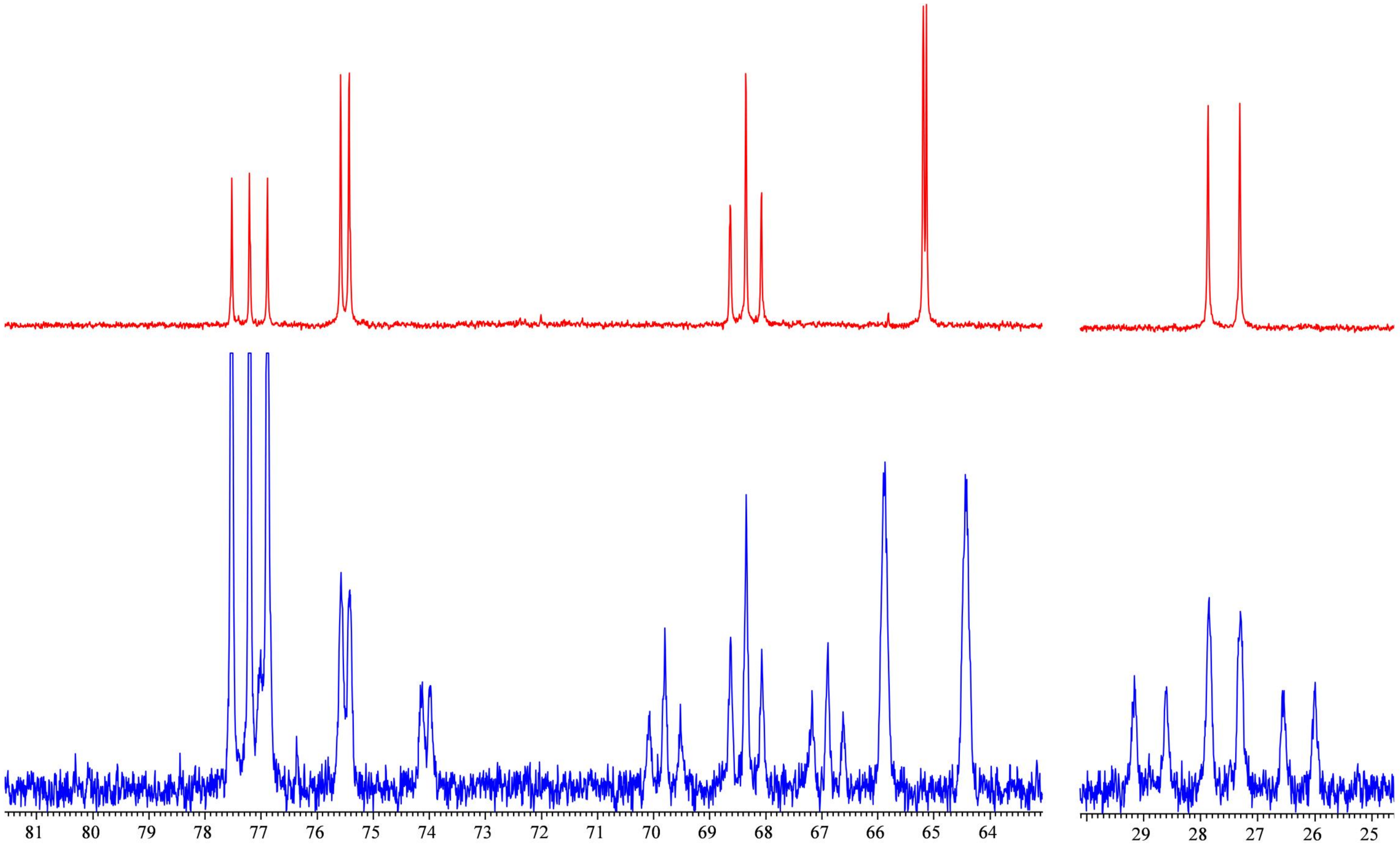

Figure 96. High-field fragments of of ^{13}C-{^{1}H} and ^{13}C NMR spectra (100.6 MHz, $CDCl_3$) of compound (**3h**).

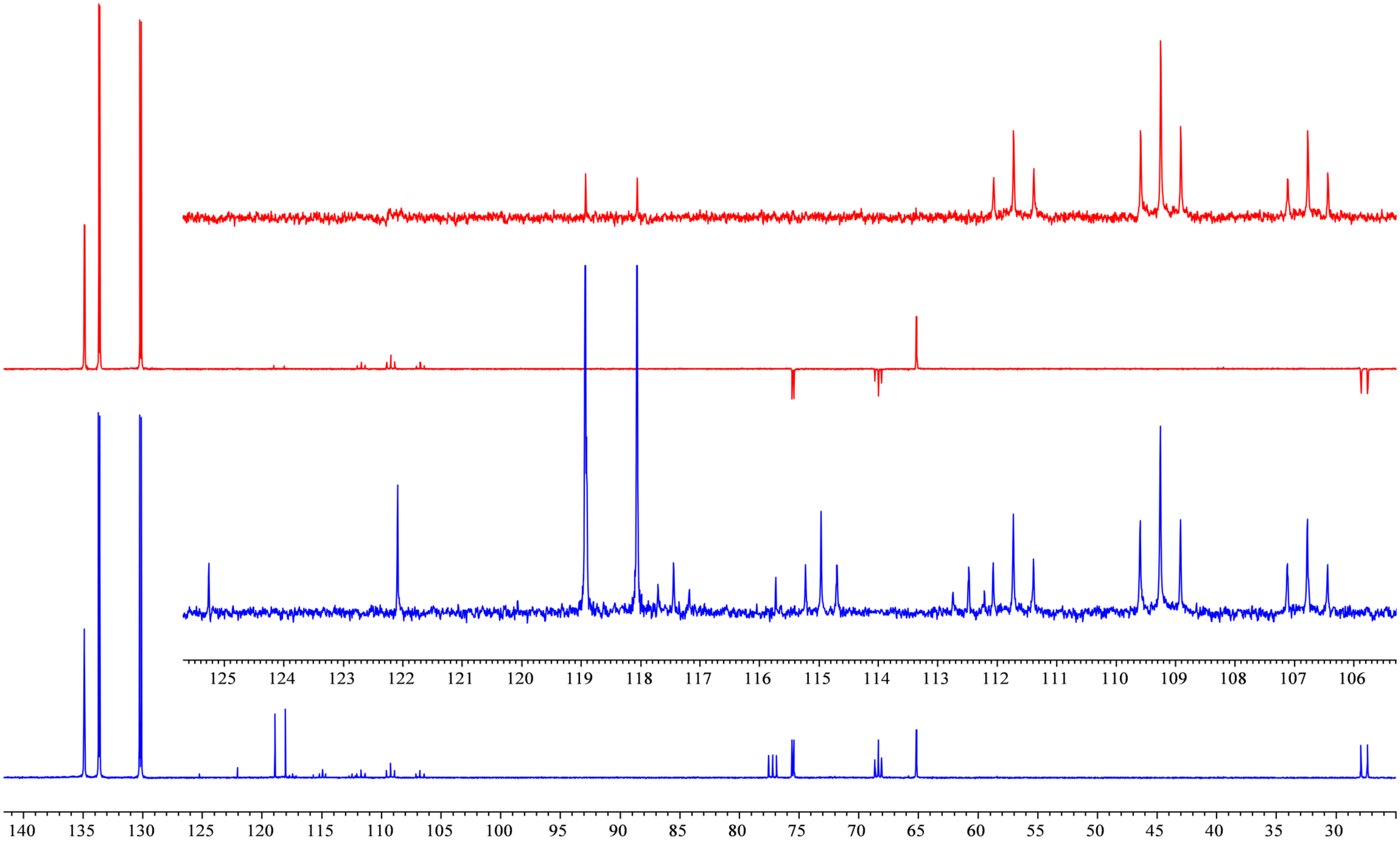

Figure 97. ^{13}C-{^{1}H} and ^{13}C-{^{1}H}-dept NMR spectra (100.6 MHz, $CDCl_3$) of compound (**3h**).

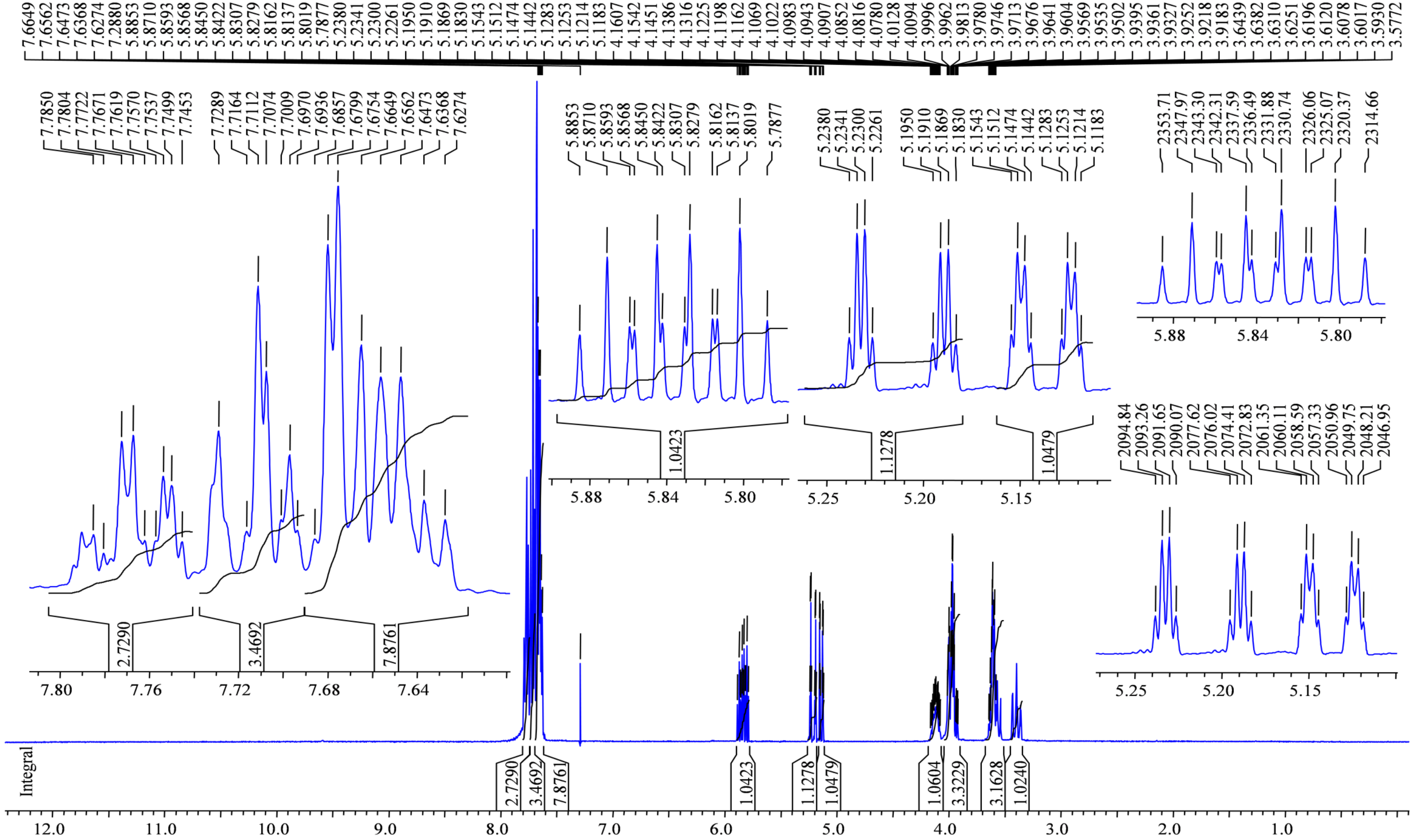

Figure 98. ^{1}H NMR spectrum (400 MHz, $CDCl_3$) of compound (**3i**).

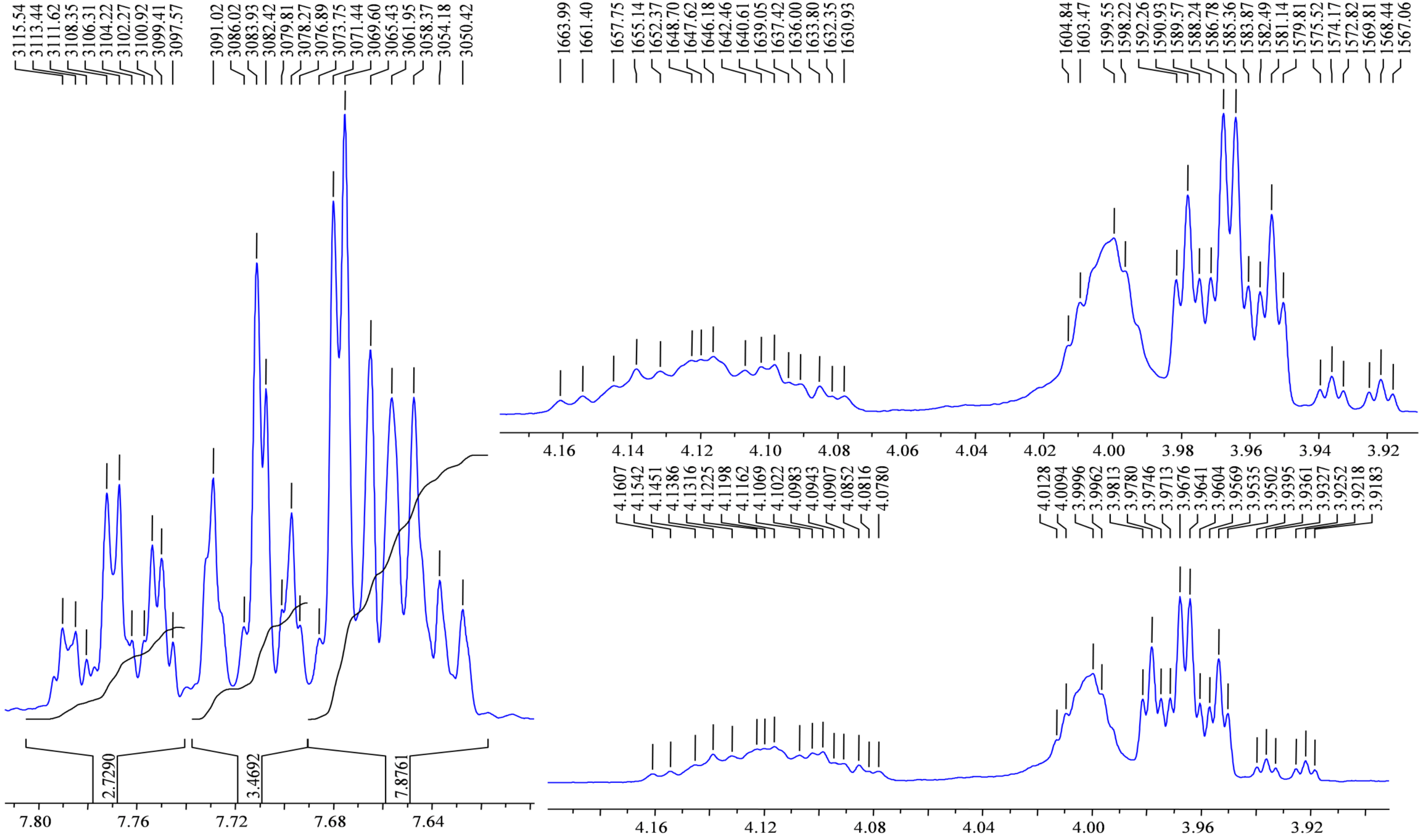

Figure 99. The 7.5-7.8 and 3.9-4.2 ppm regions of ^{1}H NMR spectrum (400 MHz, $CDCl_3$) of compound (**3i**).

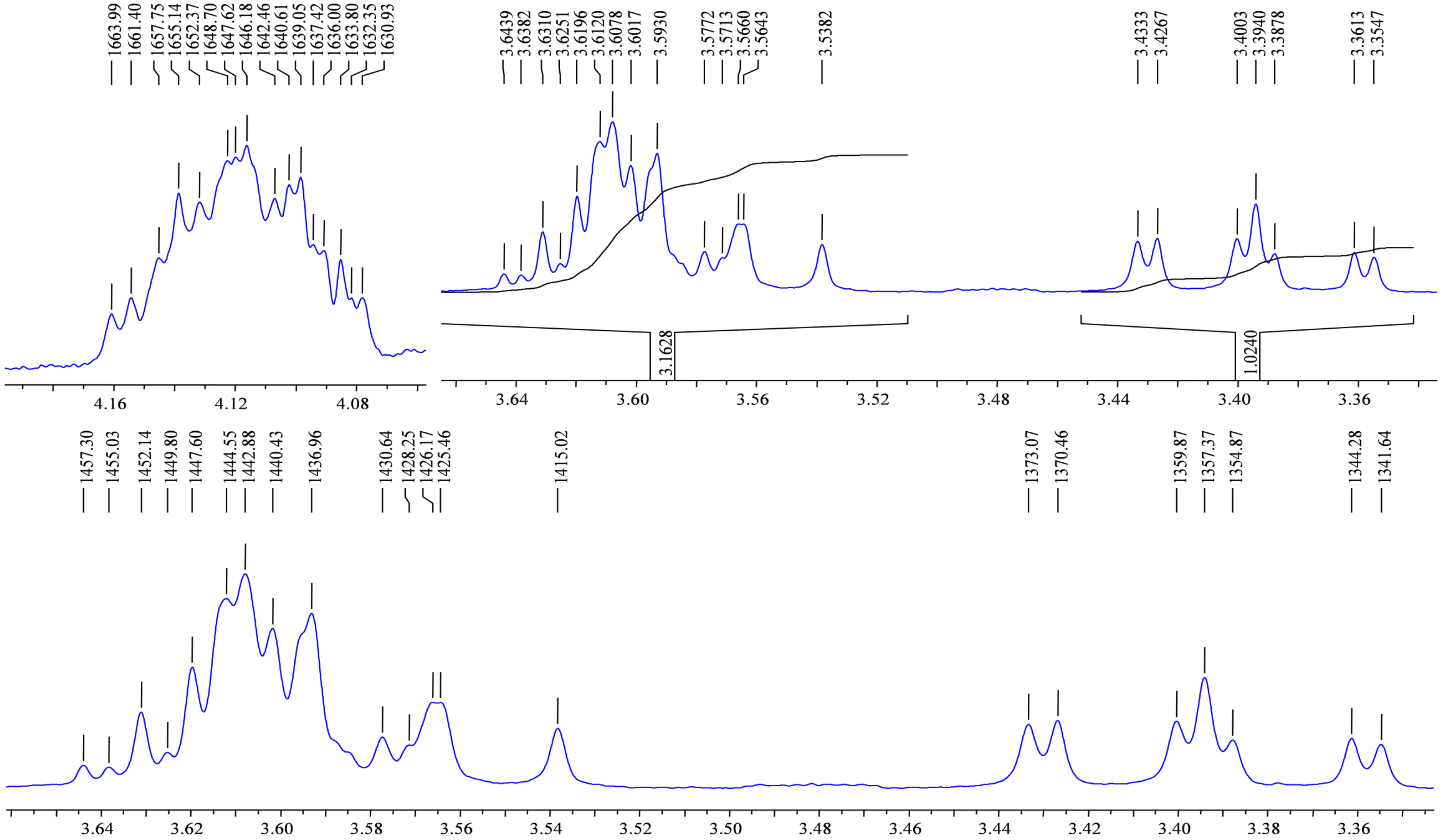

Figure 100. The 4.1-4.2 and 3.3-3.7 ppm regions of ^{1}H NMR spectrum (400 MHz, $CDCl_3$) of compound (**3i**).

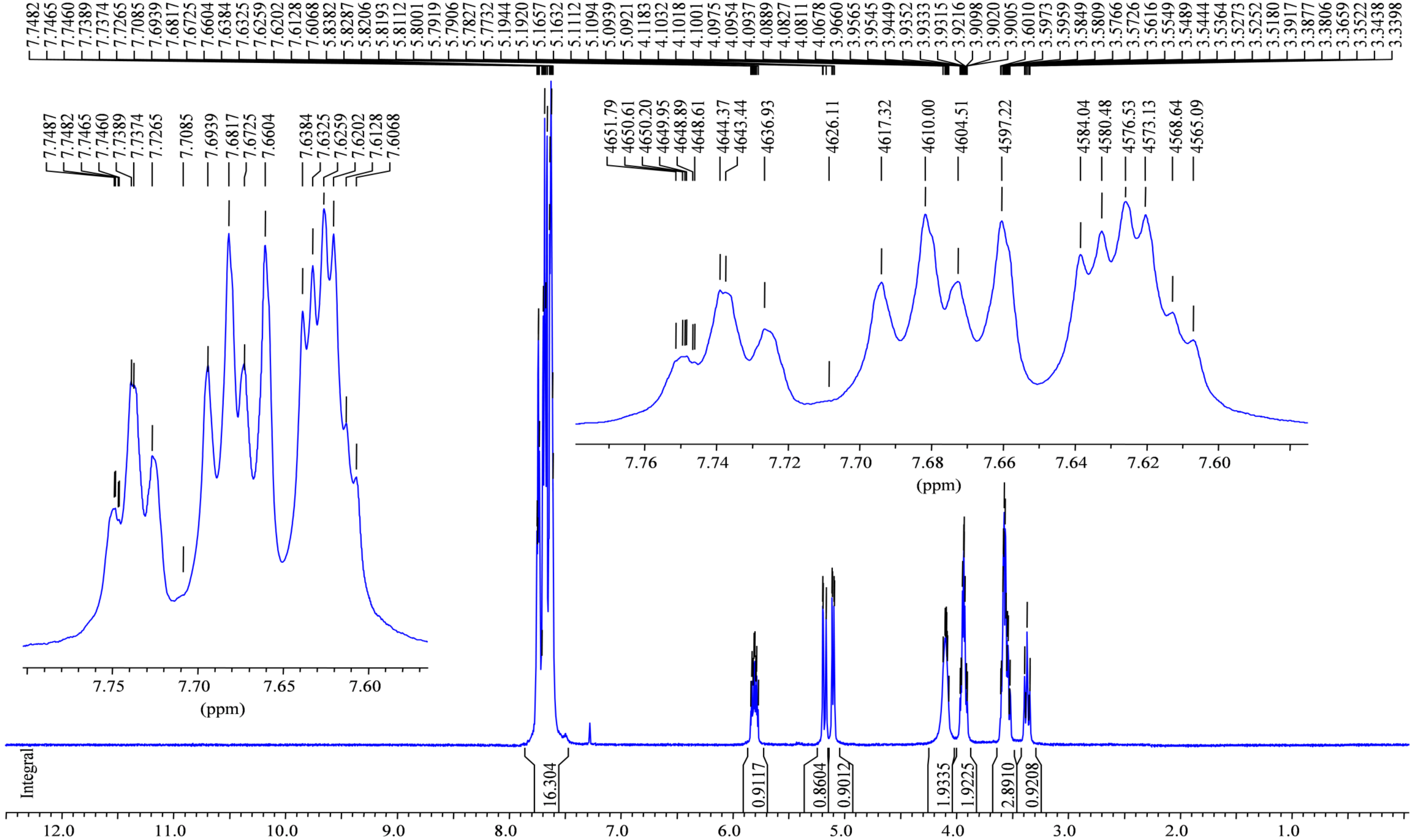

Figure 101. ^{1}H NMR spectrum (600 MHz, $CDCl_3$) of compound (**3i**).

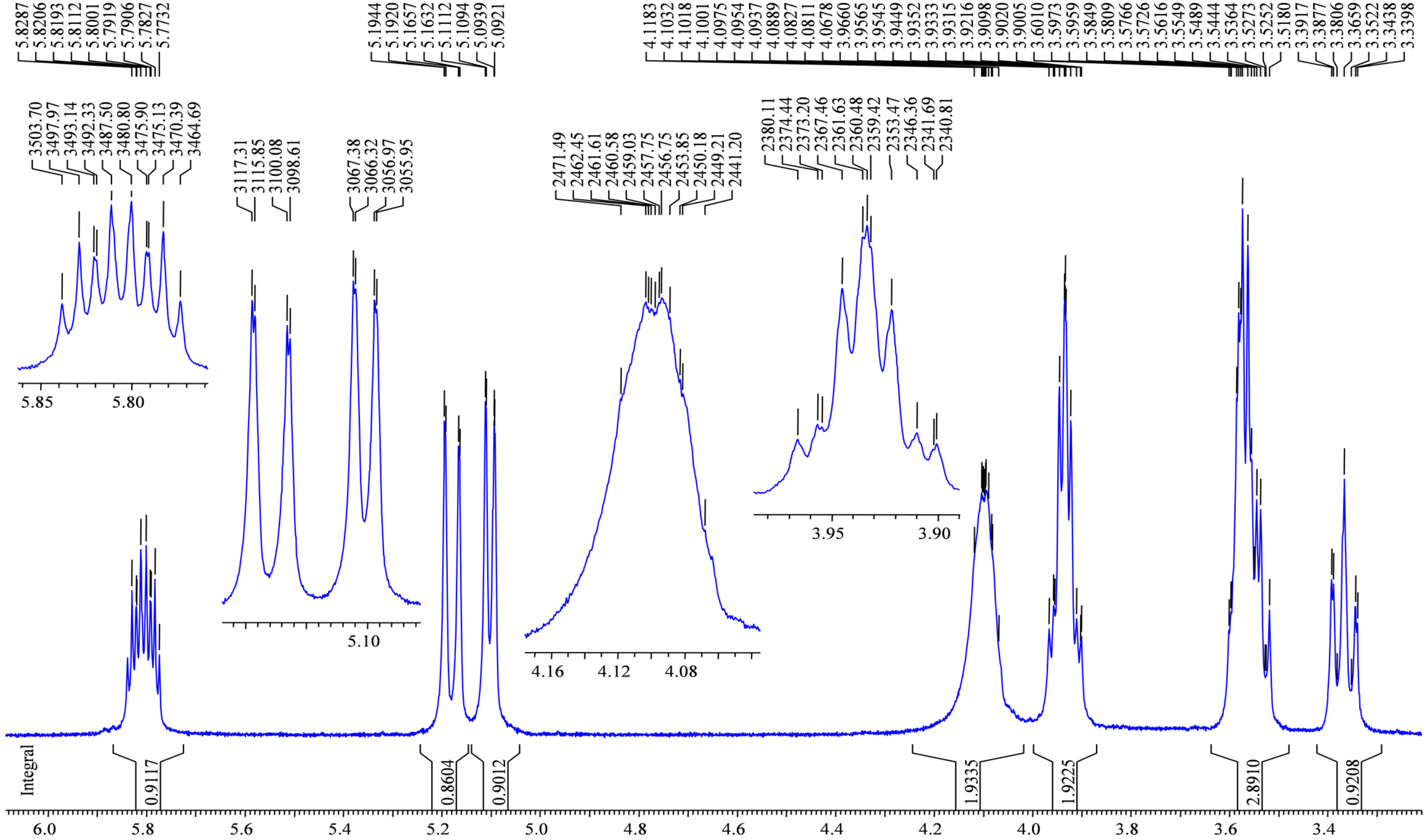
Figure 102. High-field fragment of ^{1}H NMR spectrum (600 MHz, $CDCl_3$) of compound (**3i**).

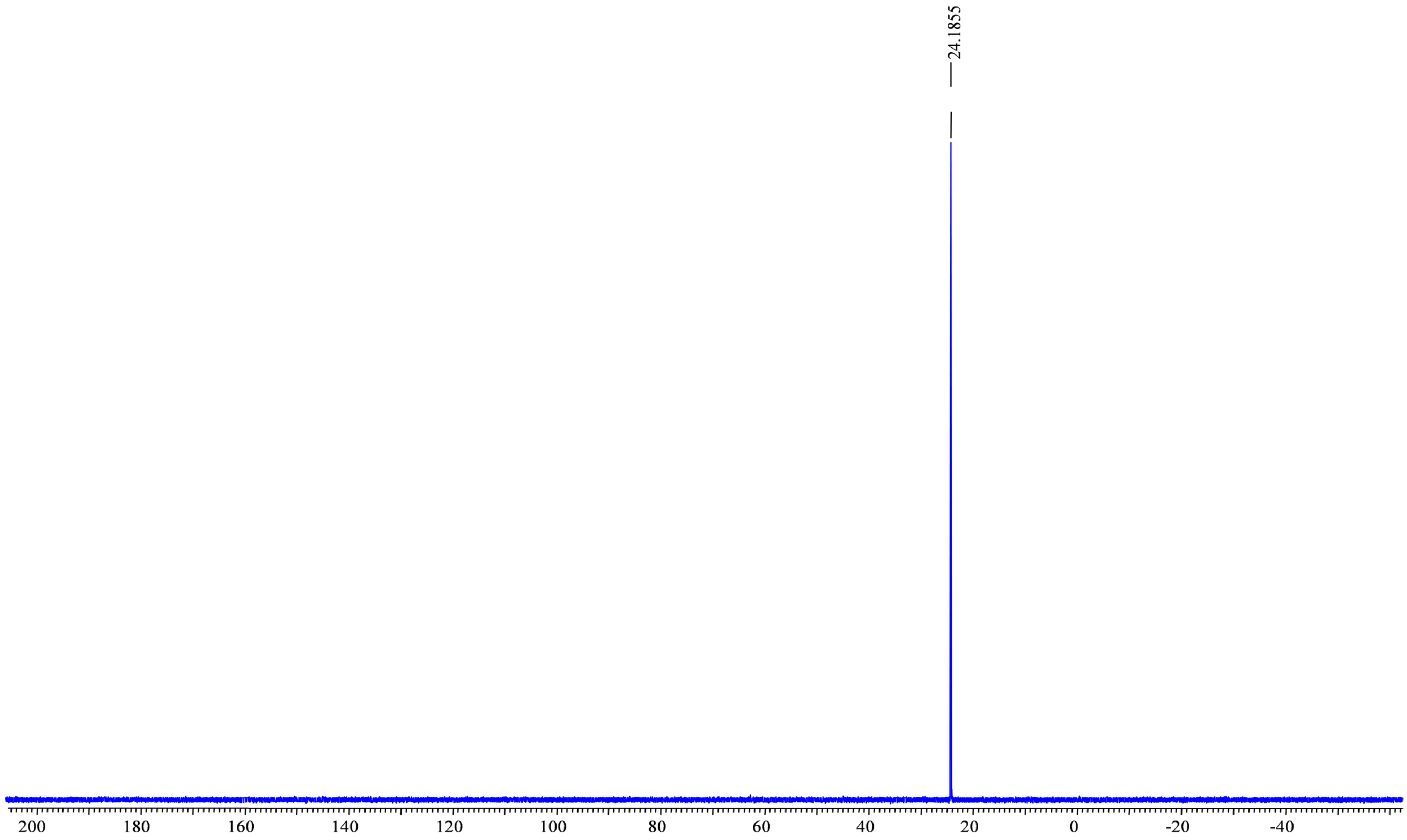

24.1855
200
180
160
140
120
100
80
60
40
20
0
-20
-40

Figure 103. $^{31}P-\{^{1}H\}$ NMR spectrum (242.94 MHz, $CDCl_3$) of compound (**3i**).

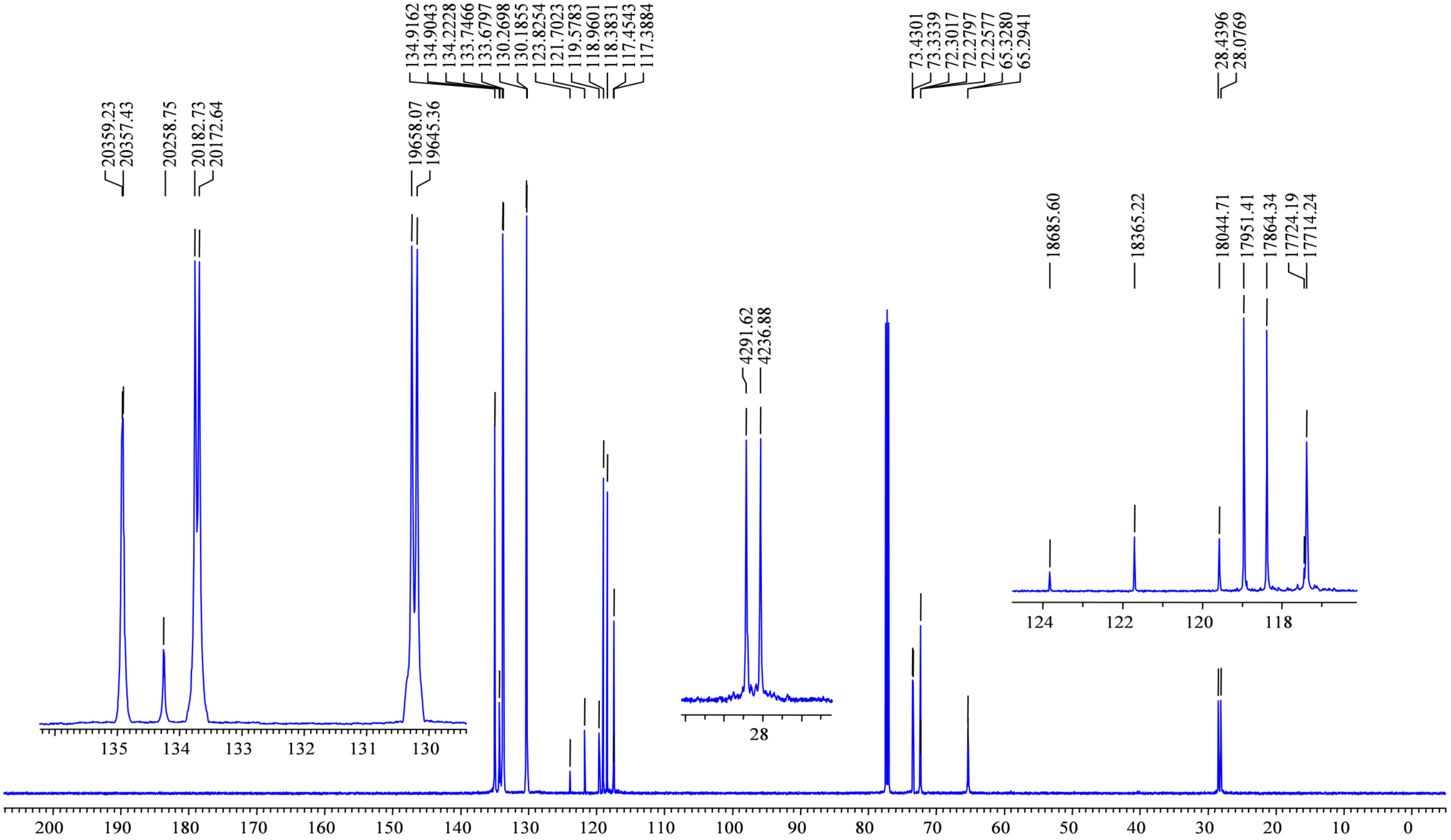

Figure 104. $^{13}C-\{^{1}H\}$ NMR spectrum (150.9 MHz, $CDCl_3$) of compound (**3i**).

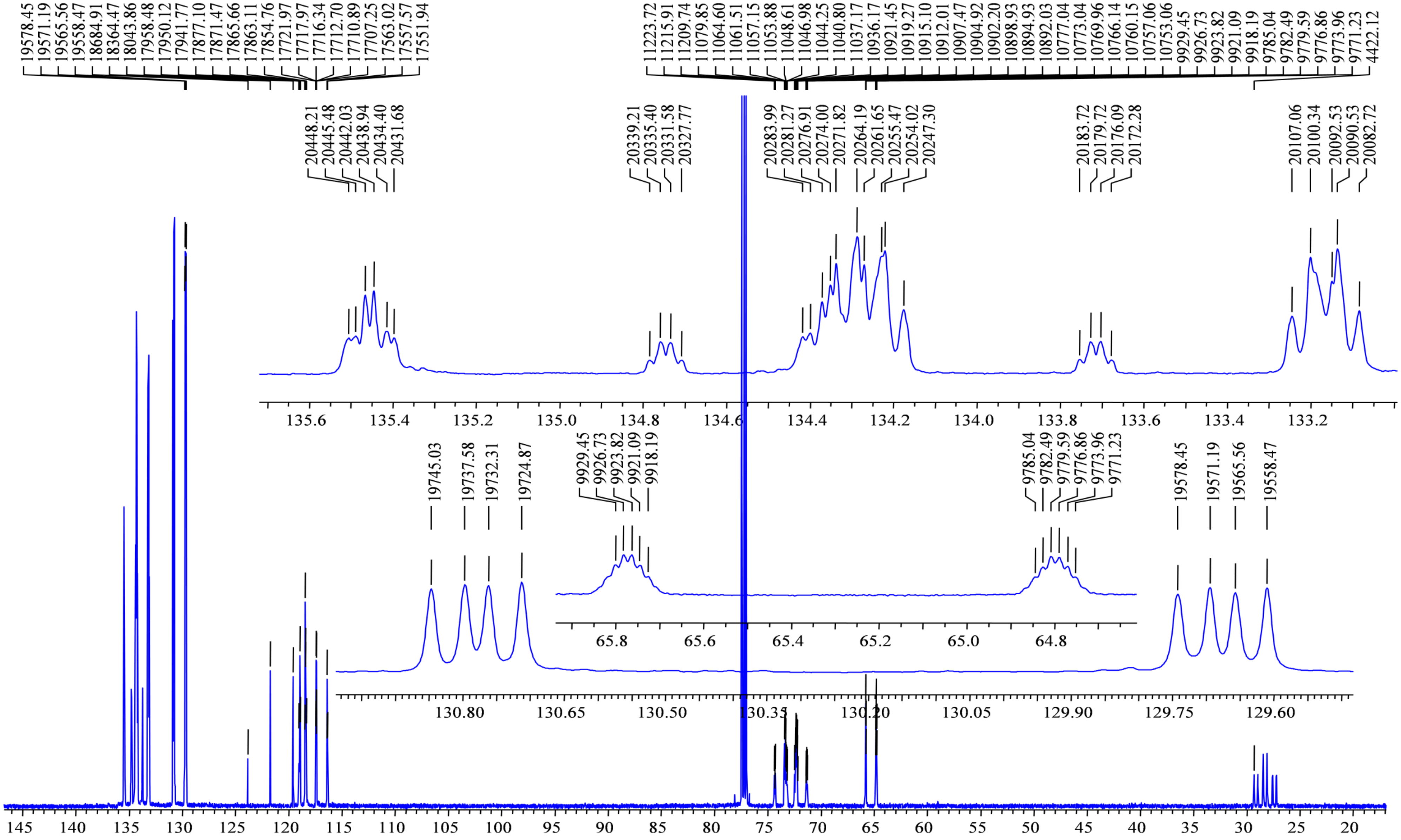

Figure 105. ^{13}C NMR spectrum (150.9 MHz, $CDCl_3$) of compound (**3i**).

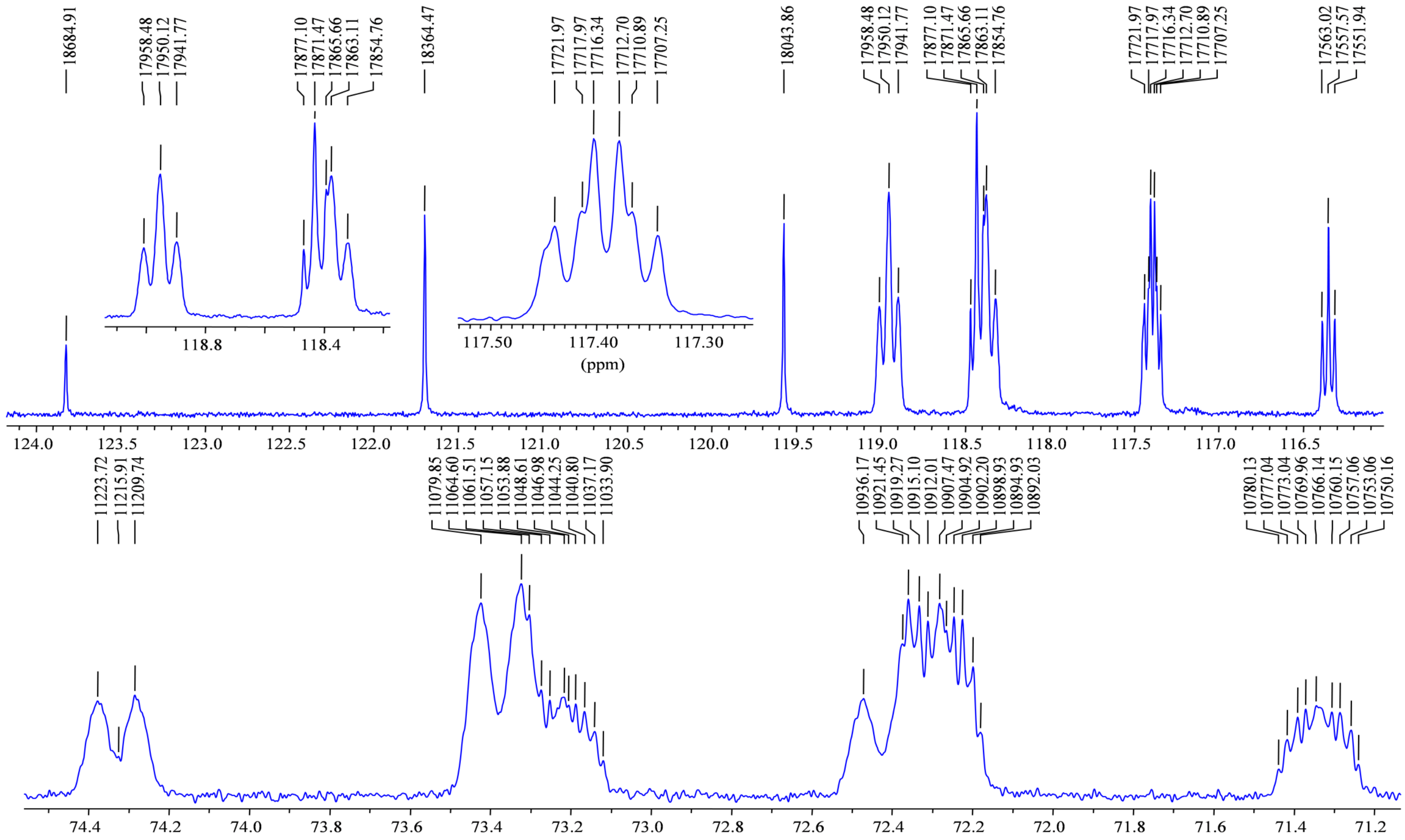

Figure 106. The 72-75 and 116-124 ppm regions of ^{13}C NMR spectrum (150.9 MHz, $CDCl_3$) of compound (**3i**).

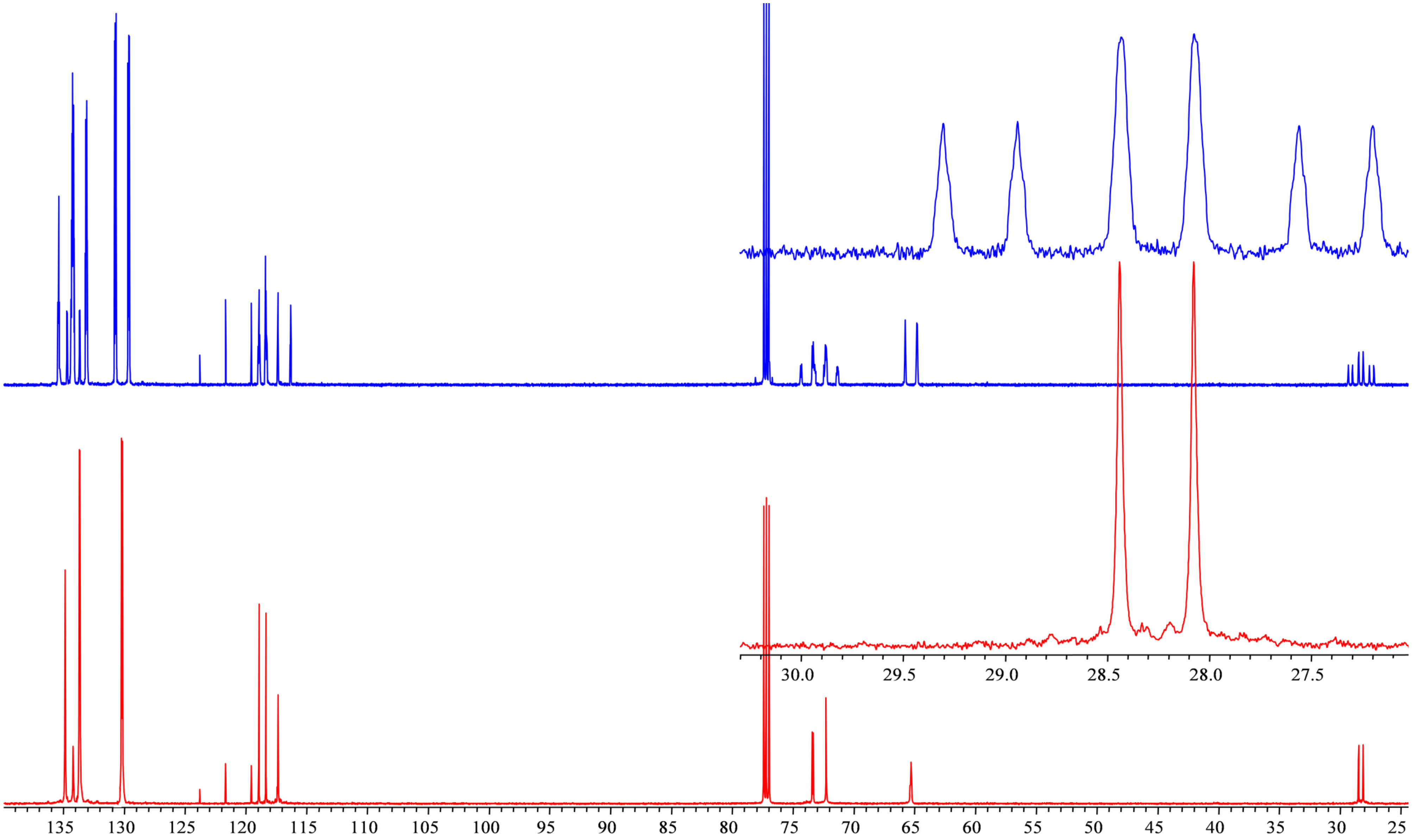

Figure 107. ^{13}C and ^{13}C-{^{1}H} NMR spectra (150.9 MHz, $CDCl_3$) of compound (**3i**).

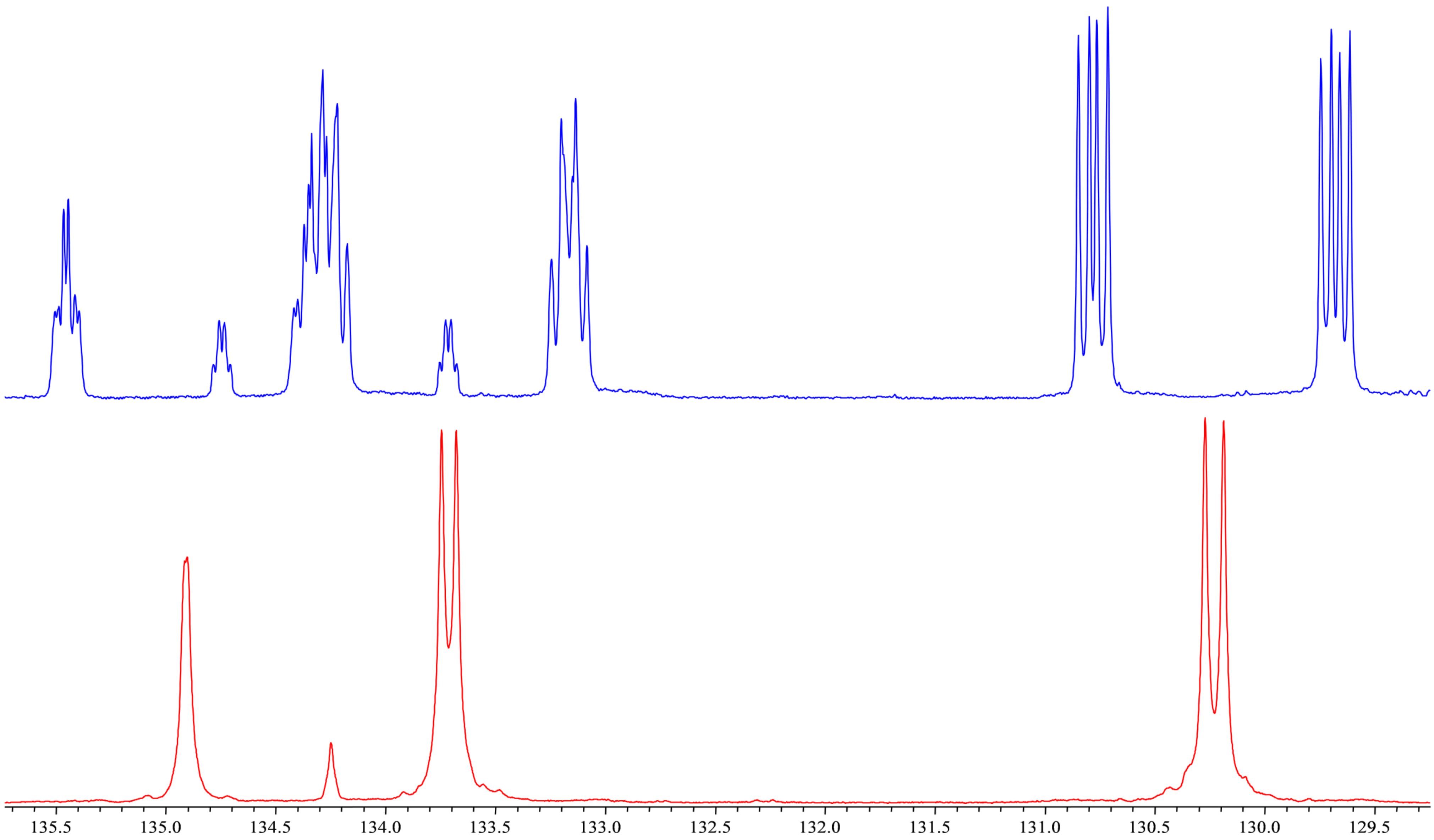

Figure 108. Aromatic carbons region of ^{13}C-{^{1}H} and ^{13}C NMR spectra (150.9 MHz, $CDCl_3$) of compound (**3i**).

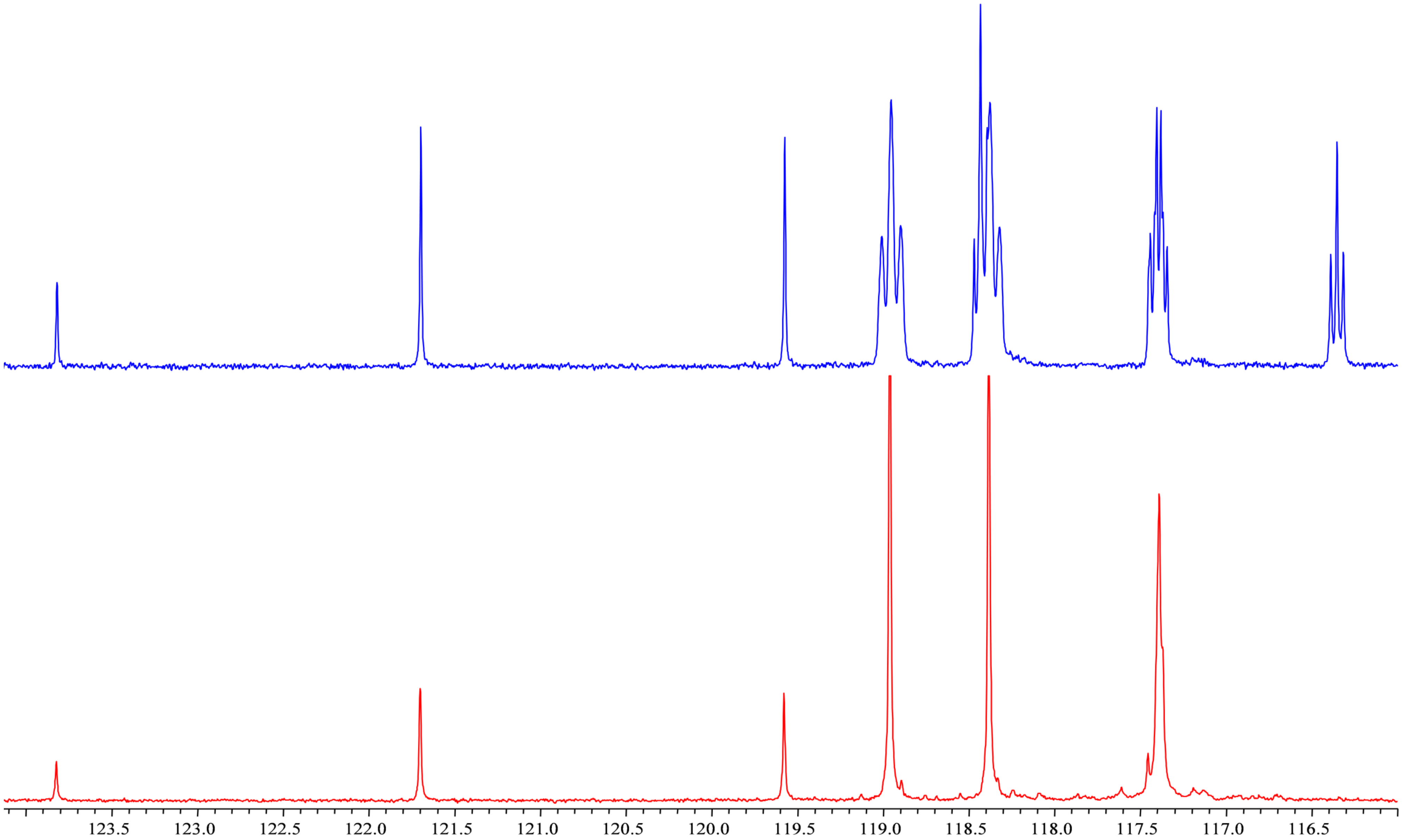

Figure 109. The 117-124 ppm region of ^{13}C-{^{1}H} and ^{13}C NMR spectra (150.9 MHz, $CDCl_3$) of compound (**3i**).

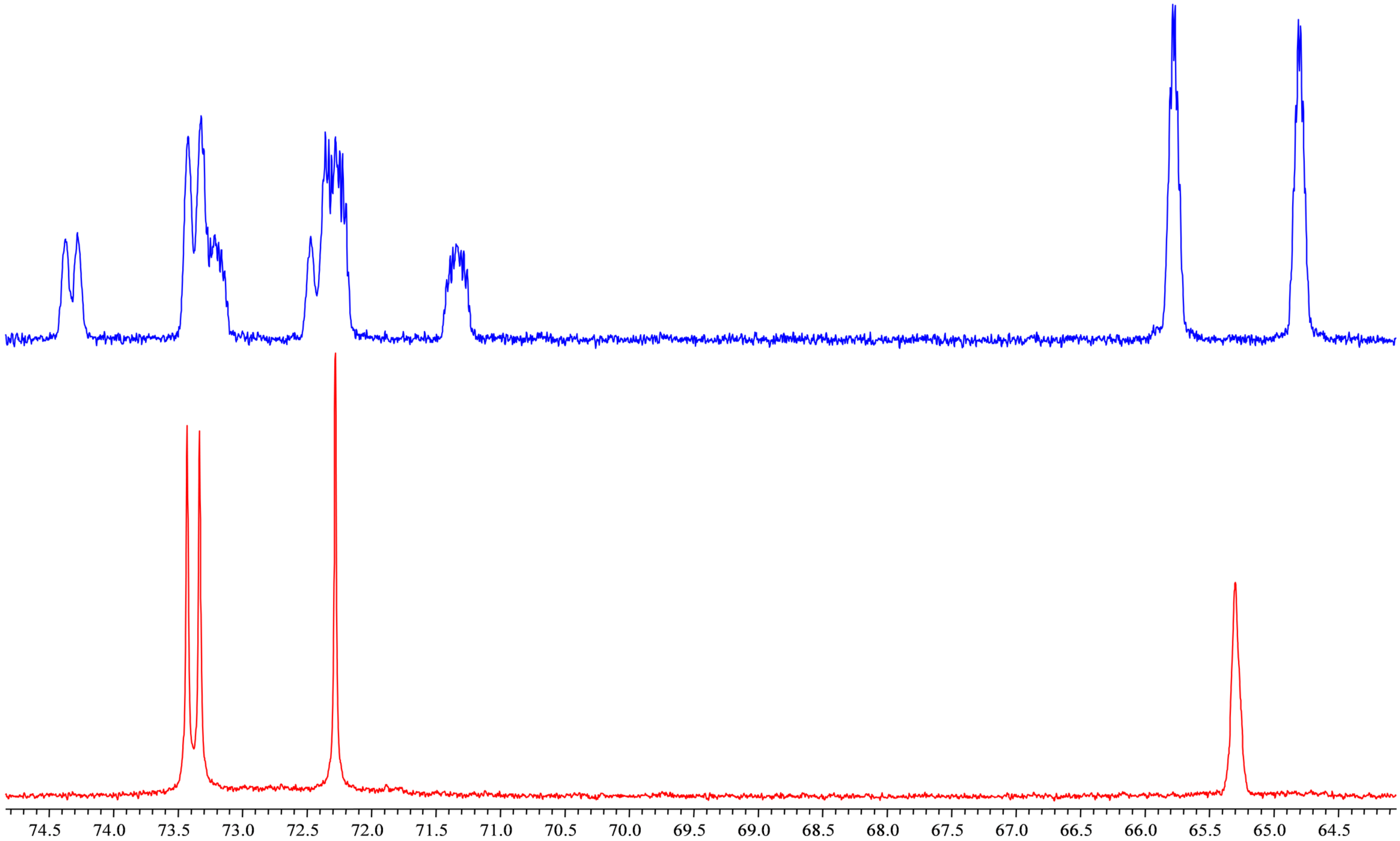

Figure 110. The 64-75 ppm region of ^{13}C-{^{1}H} and ^{13}C NMR spectra (150.9 MHz, $CDCl_3$) of compound (**3i**).

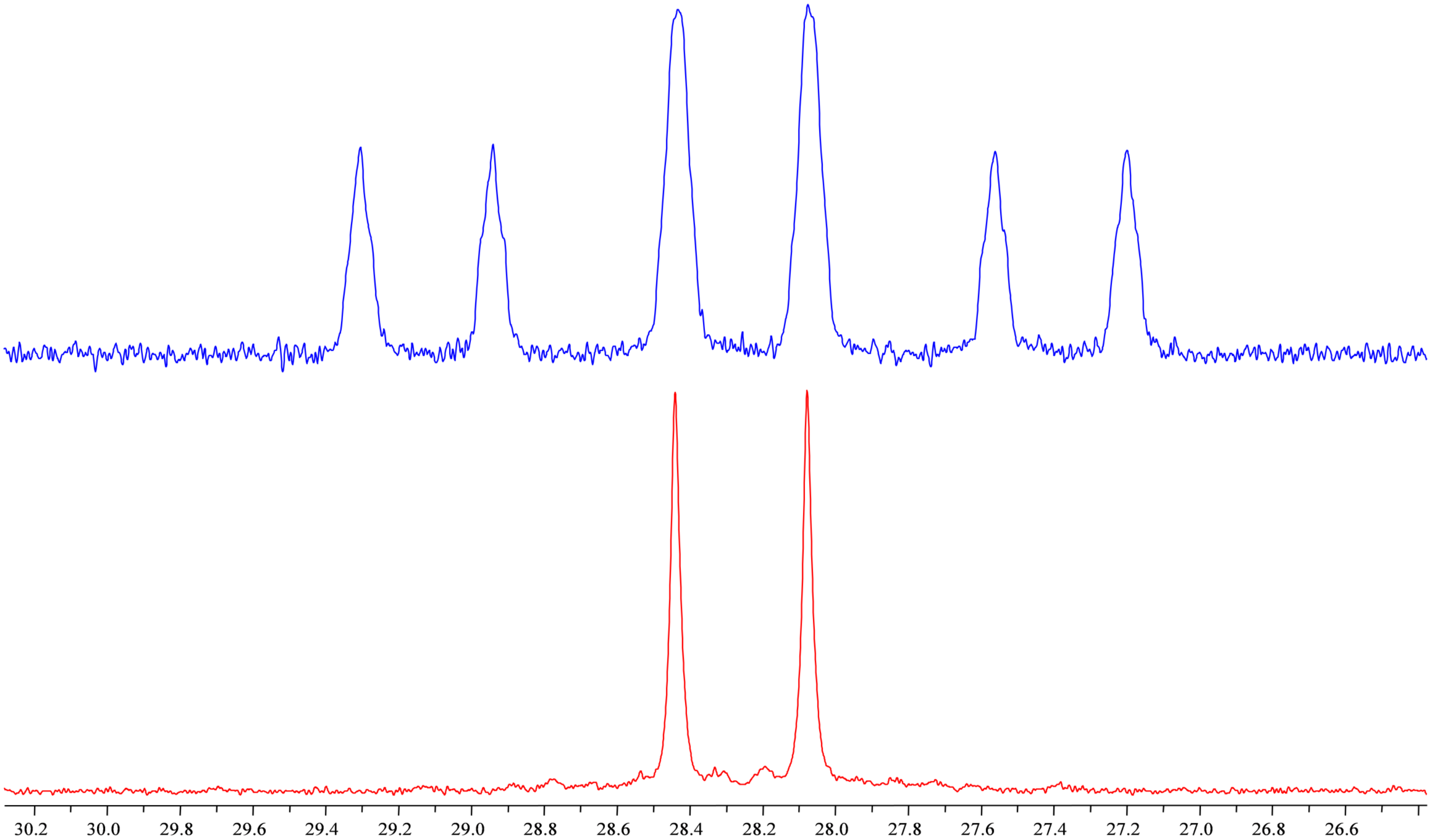

Figure 111. High-field fragment of ^{13}C-{^{1}H} and ^{13}C NMR spectra (150.9 MHz, $CDCl_3$) of compound (**3i**).

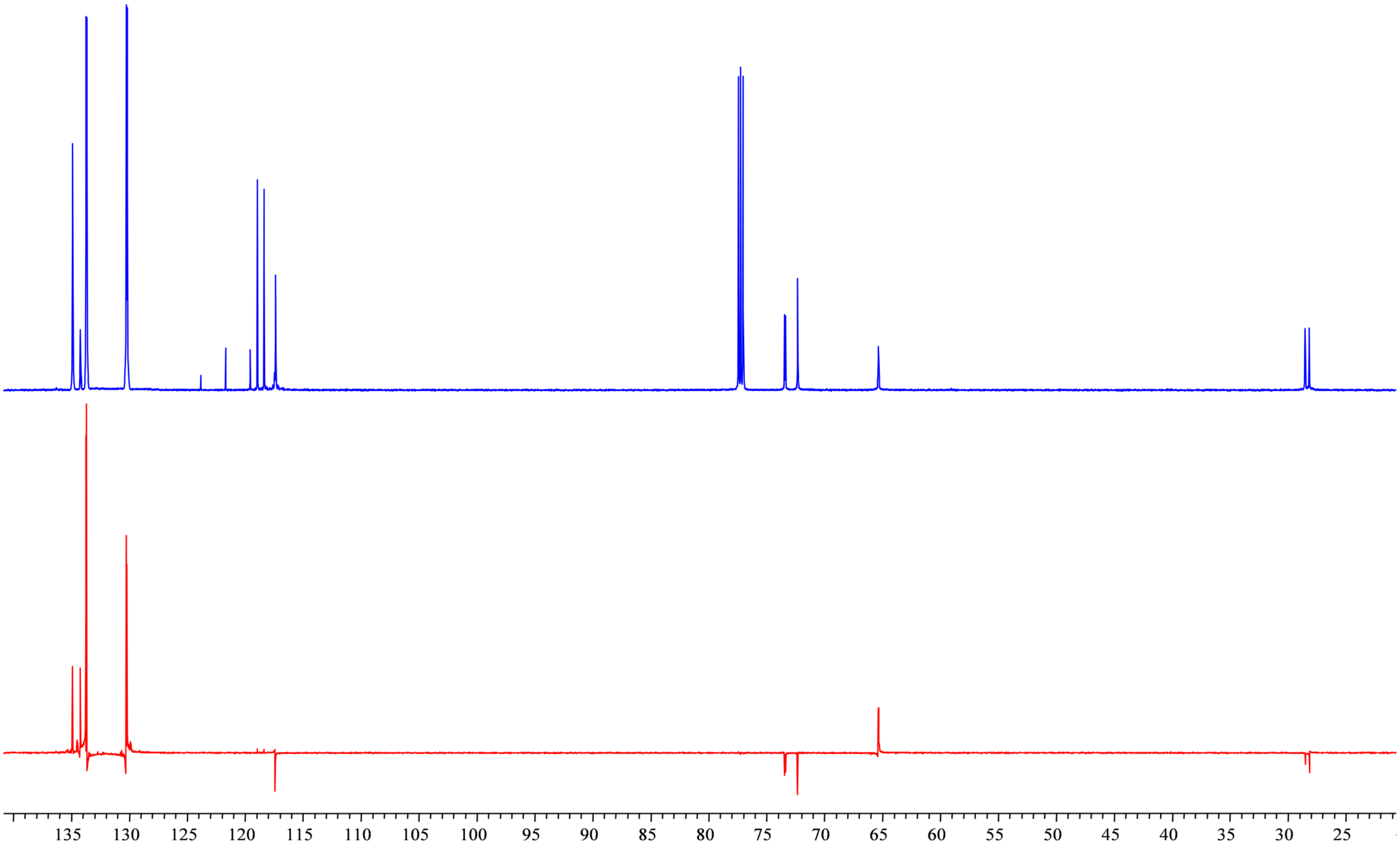

Figure 112. ^{13}C-{^{1}H} and ^{13}C-{^{1}H}-dept NMR spectra (150.9 MHz, $CDCl_3$) of compound (**3i**).

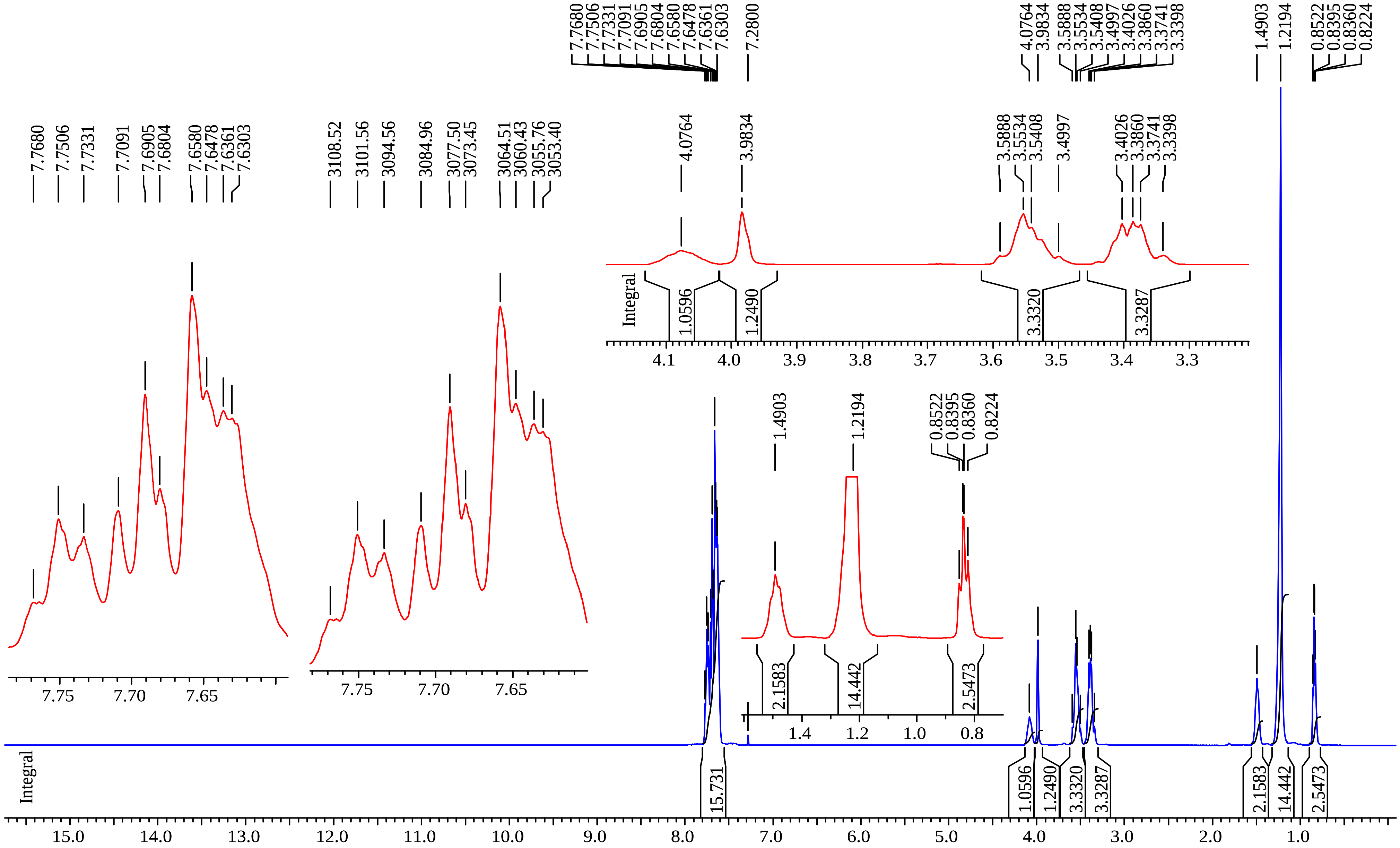

Figure 113. ^{1}H NMR spectrum (400 MHz, $CDCl_3$) of compound (**3j**).

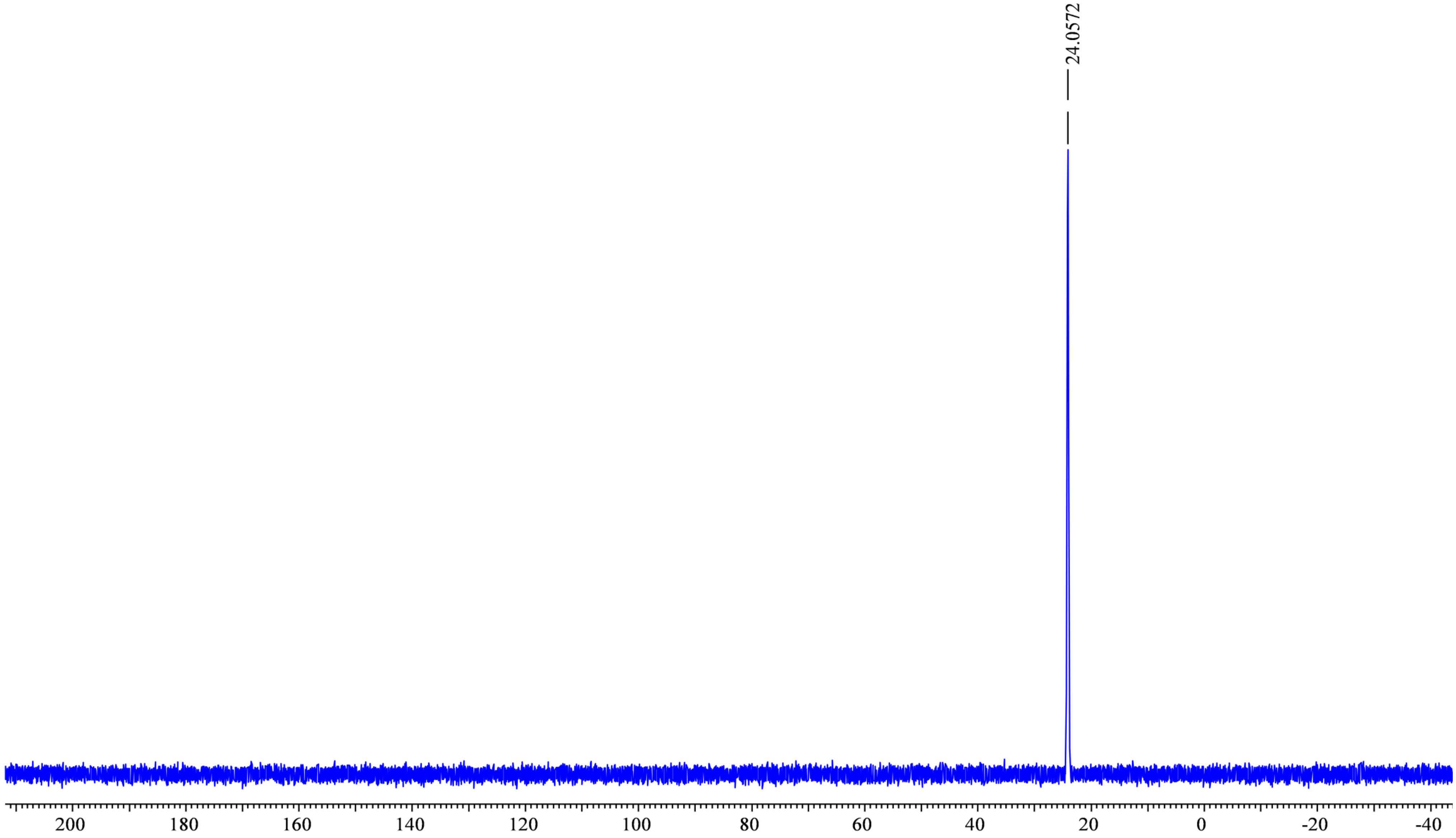

Figure 114. ^{31}P-{^{1}H} NMR spectrum (162.0 MHz, $CDCl_3$) of compound (**3j**).

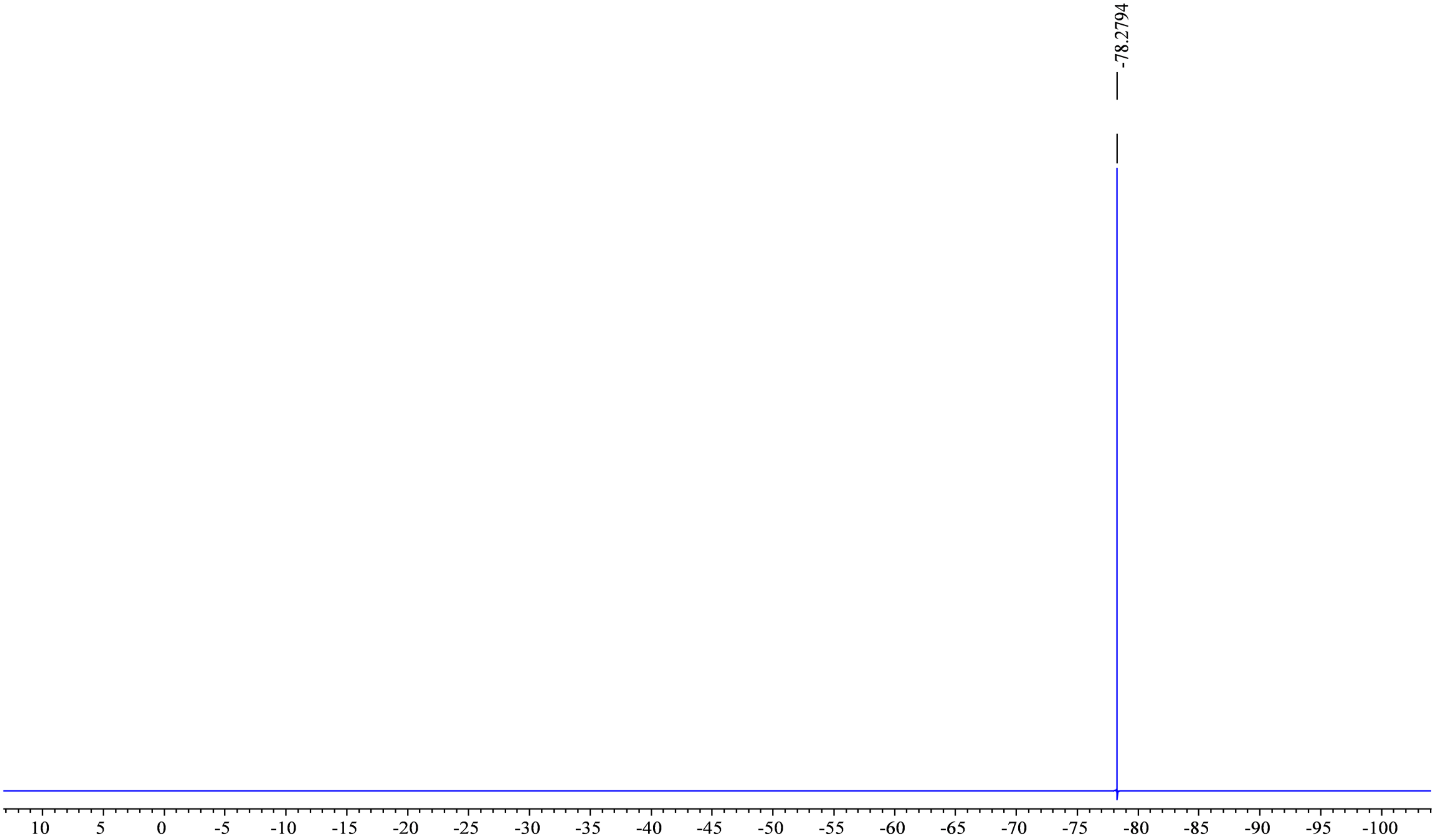

Figure 115. ^{19}F NMR spectrum (376.5 MHz, $CDCl_3$) of compound (**3j**).

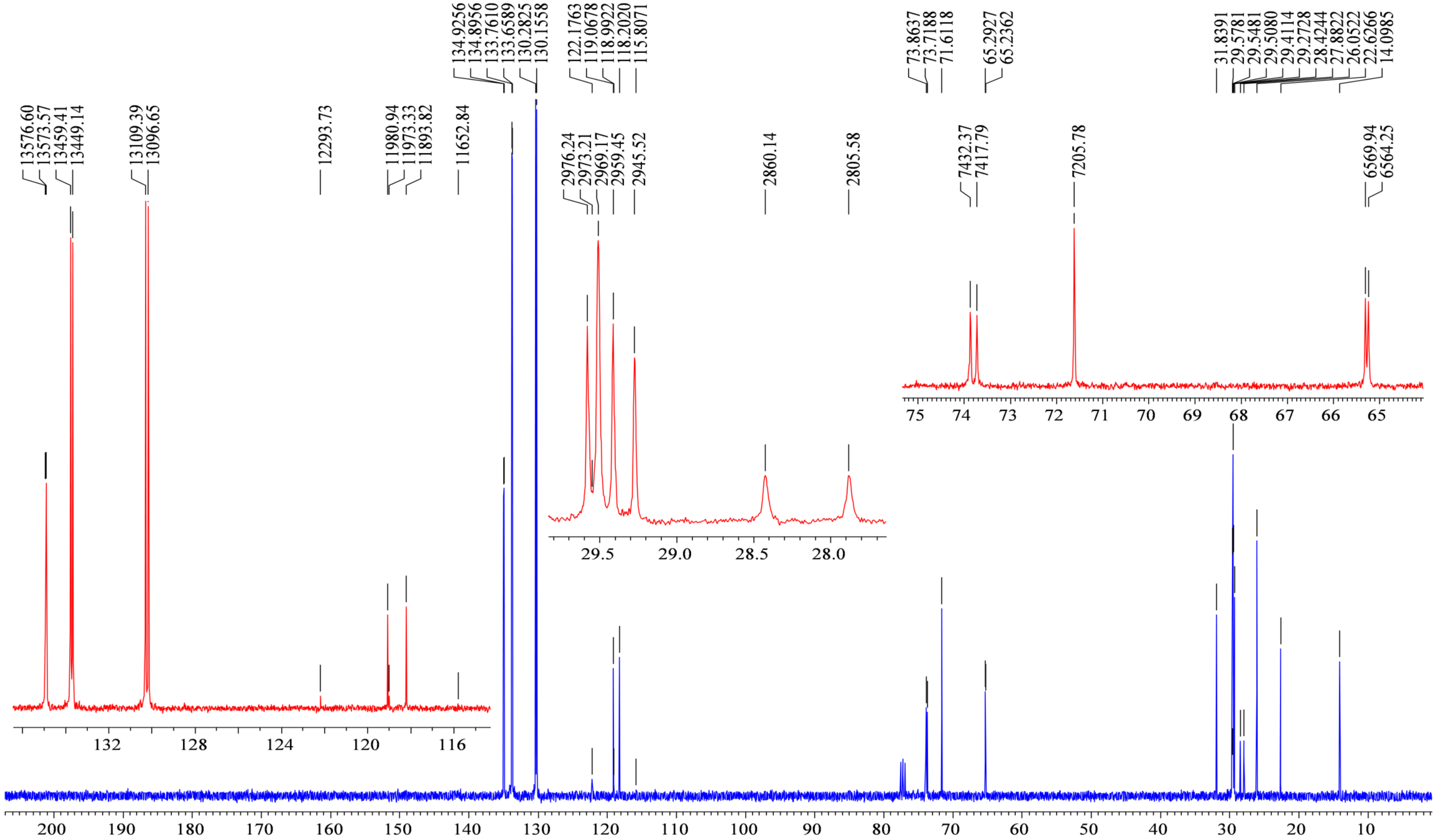

Figure 116. ^{13}C-{^{1}H} NMR spectrum (100.6 MHz, $CDCl_3$) of compound (**3j**).

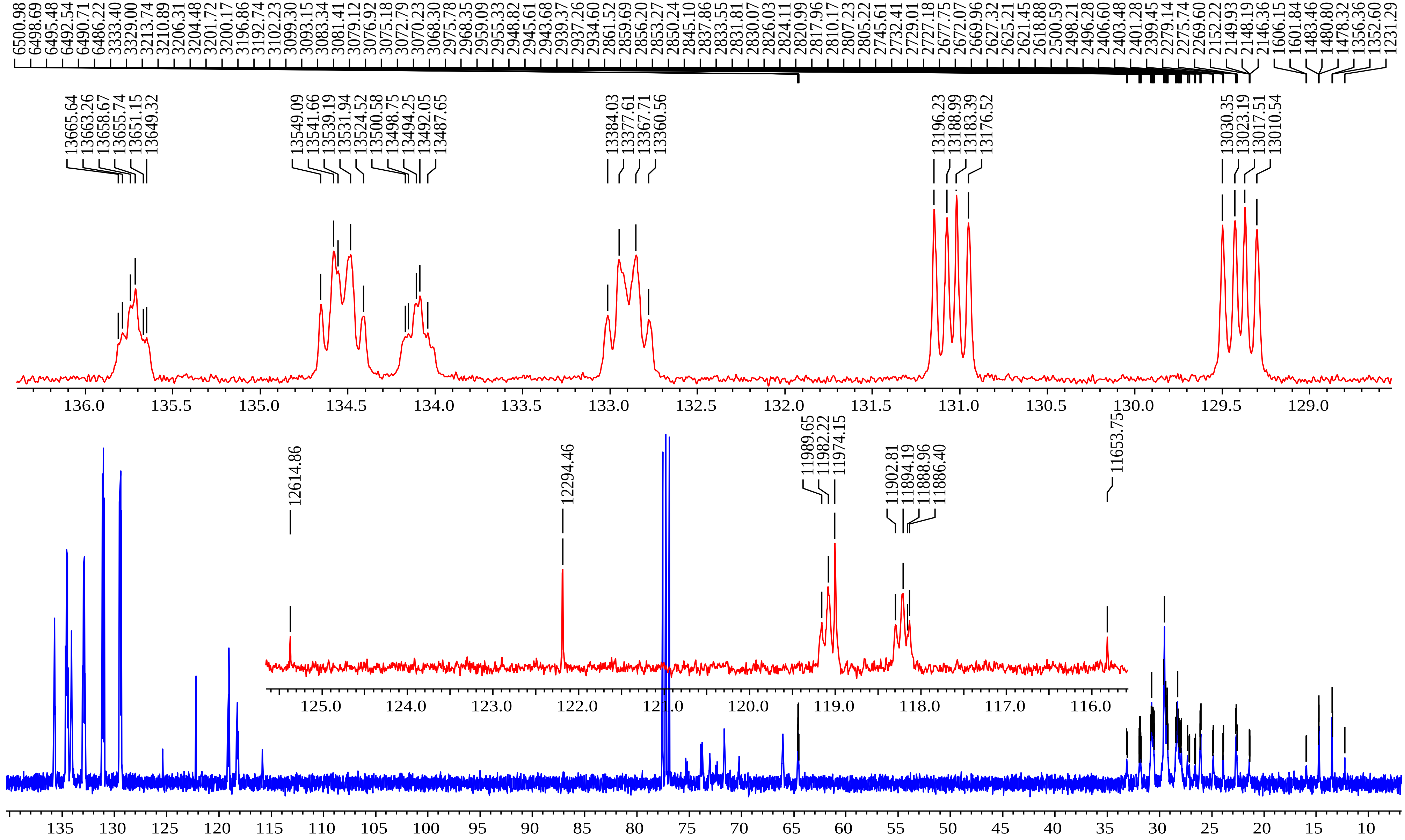

Figure 117. ^{13}C NMR spectrum (100.6 MHz, $CDCl_3$) of compound (**3j**).

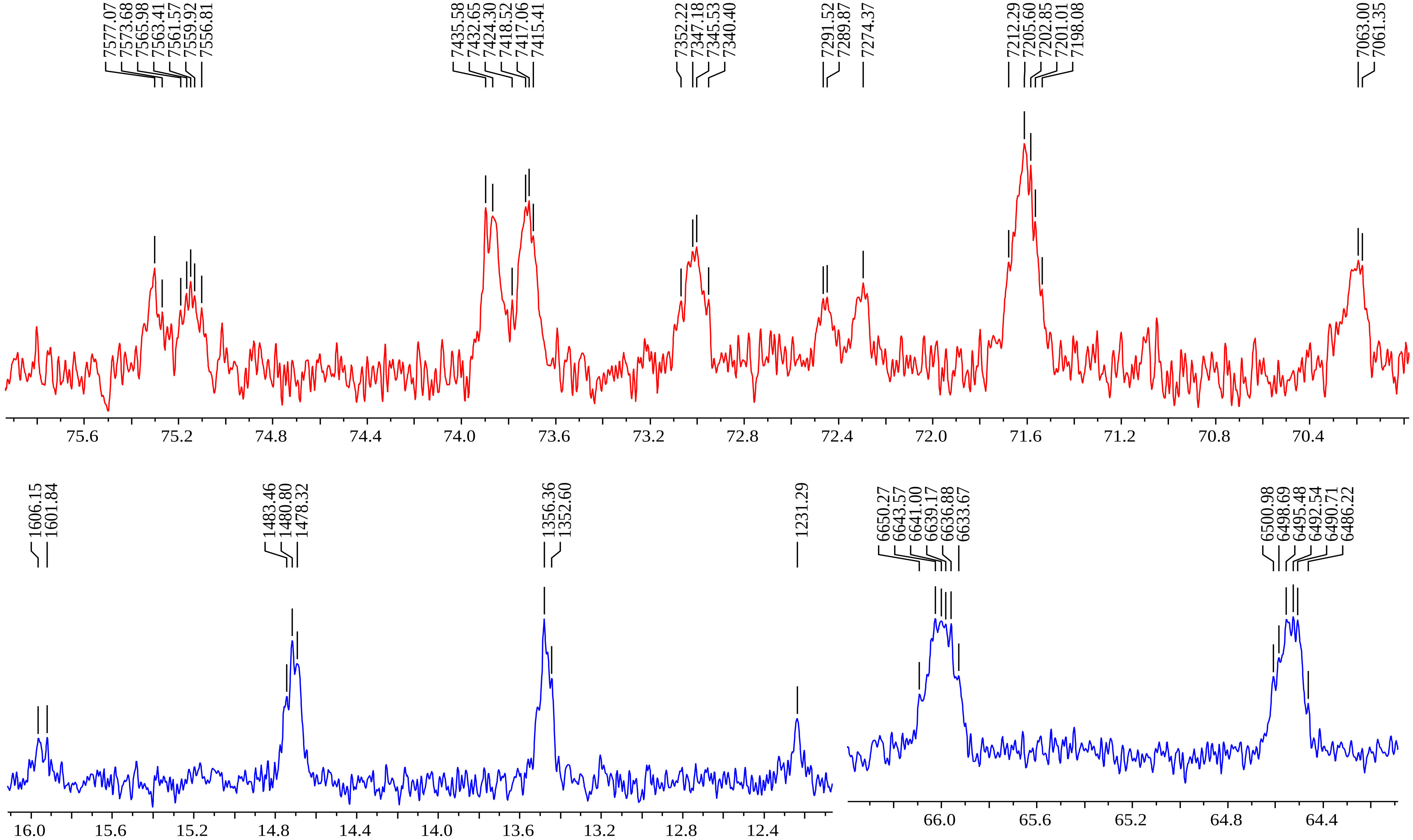

Figure 118. The 11-16, 63-67 and 70-76 ppm regions of ^{13}C NMR spectrum (100.6 MHz, $CDCl_3$) of compound (**3j**).

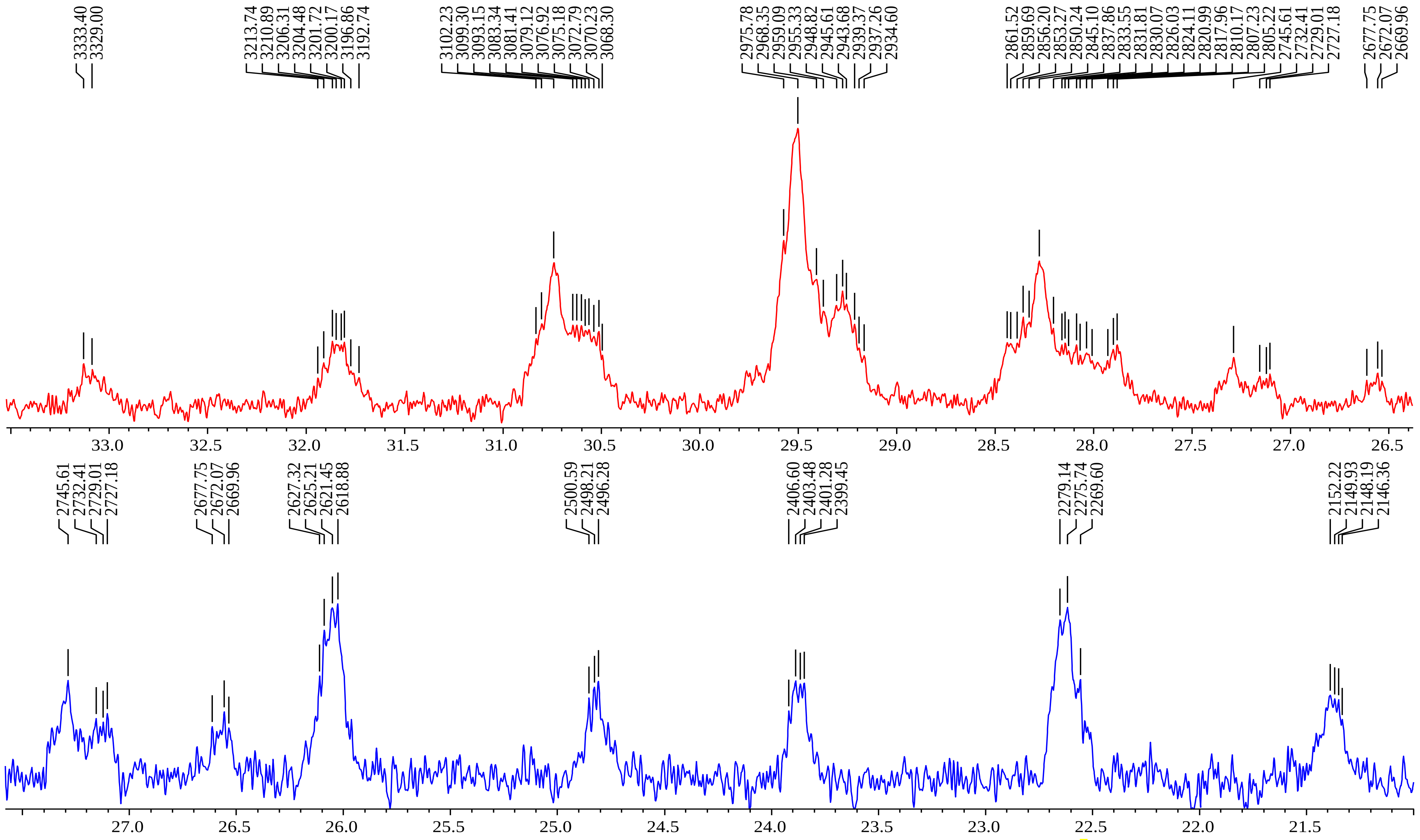

Figure 119. The 20-28 and 27-34 ppm regions of ^{13}C and ^{13}C -{^{1}H} NMR spectra (150.9 MHz, $CDCl_3$) of compound (**3j**).

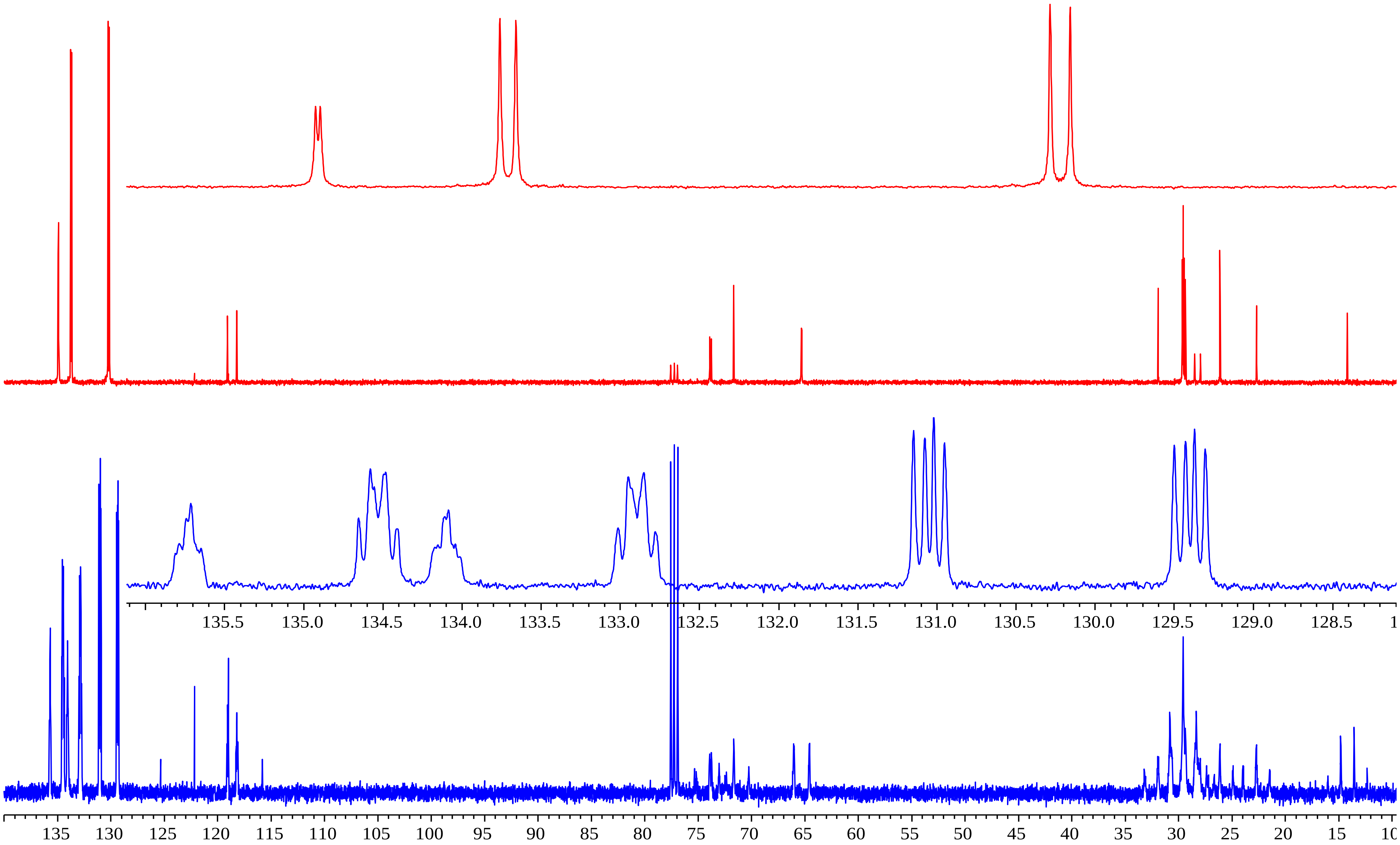

Figure 120. ^{13}C-{^{1}H} and ^{13}C NMR spectra (100.6 MHz, $CDCl_3$) of compound (**3j**).

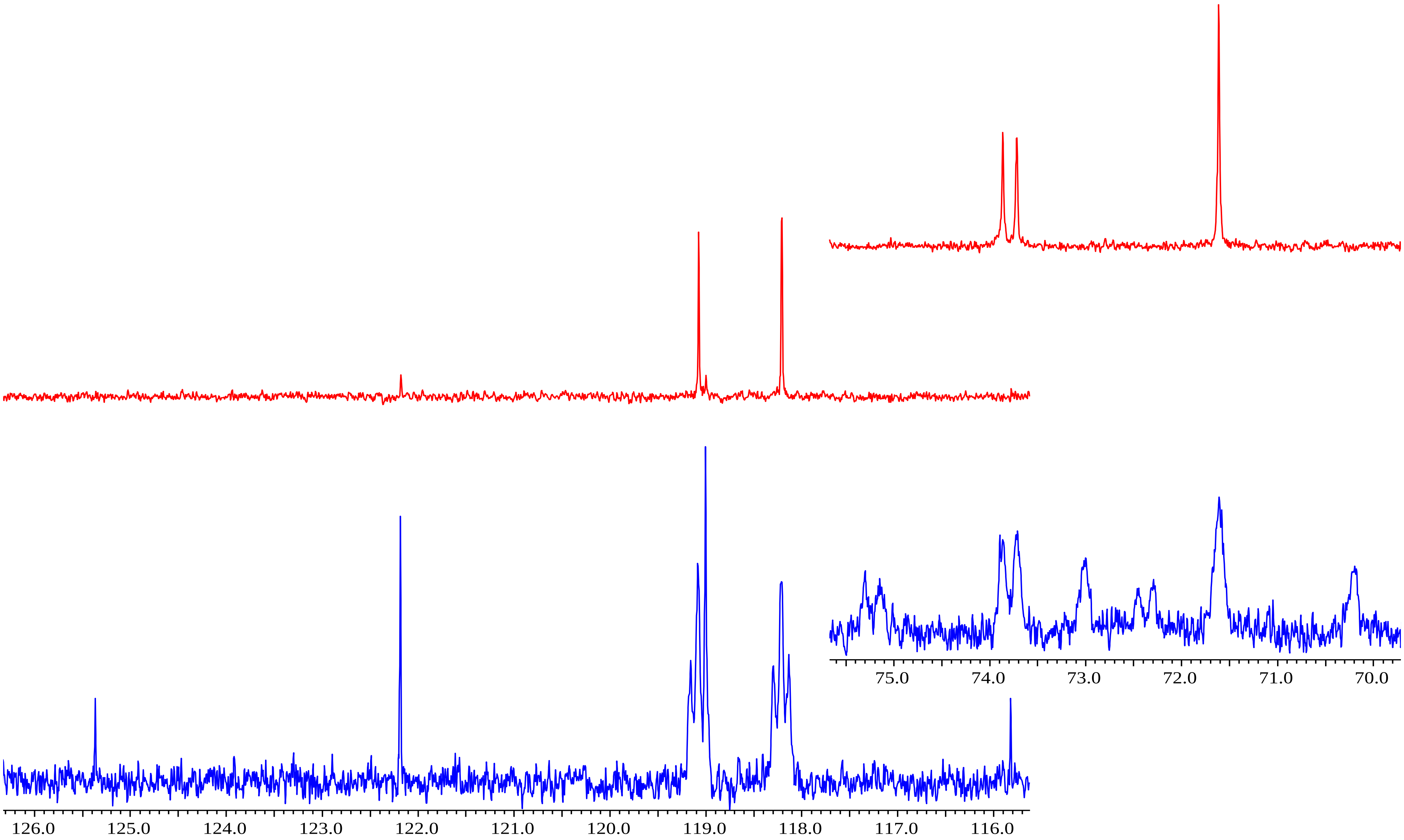

Figure 121. The 70-76 and 115-126 ppm regions of ^{13}C-{^{1}H} and ^{13}C NMR spectra (150.9 MHz, $CDCl_3$) of compound (**3j**).

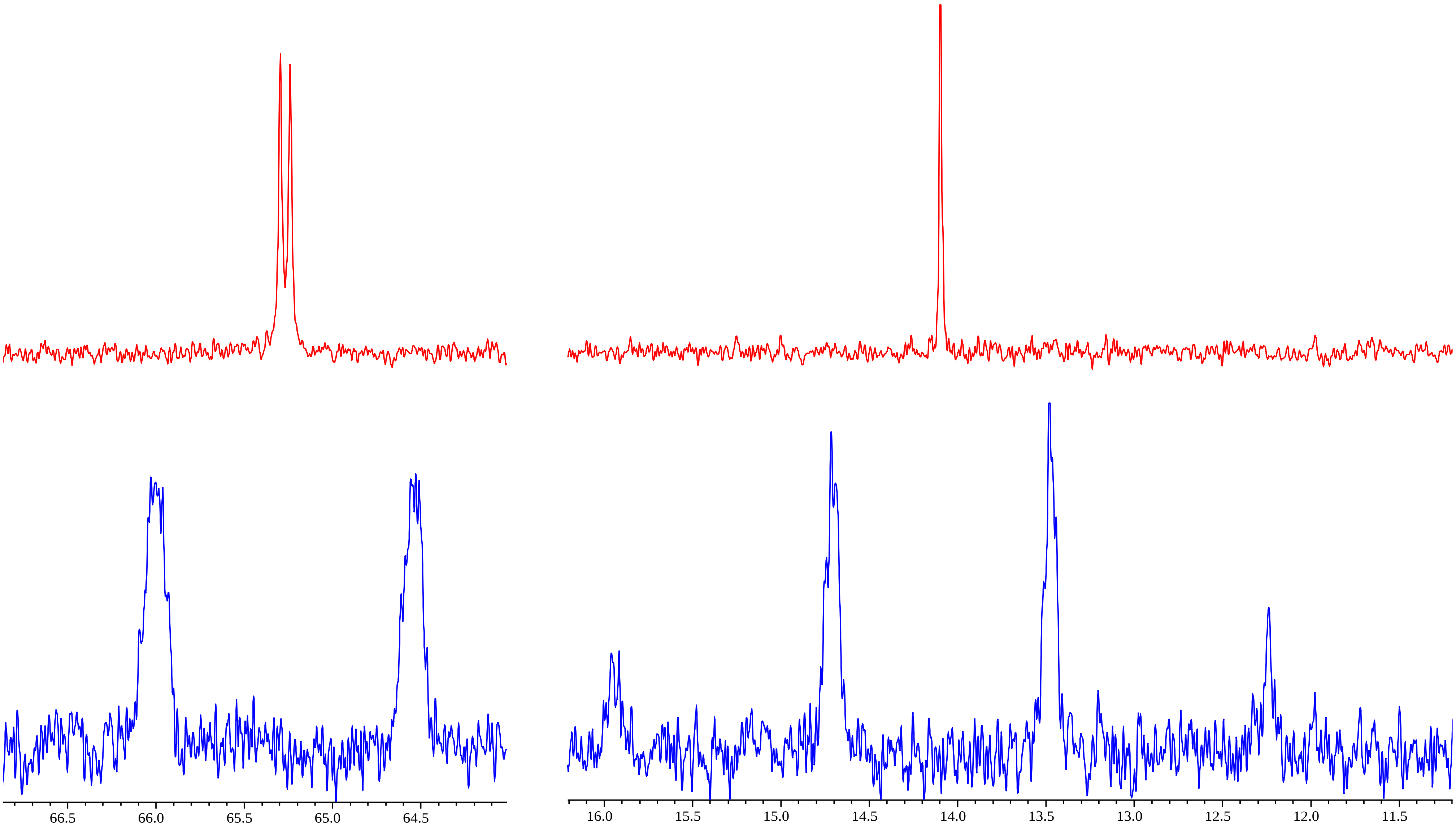

Figure 122. The 11-17 and 64-67 ppm regions of ^{13}C-{^{1}H} and ^{13}C NMR spectra (100.6 MHz, $CDCl_3$) of compound (**3j**).

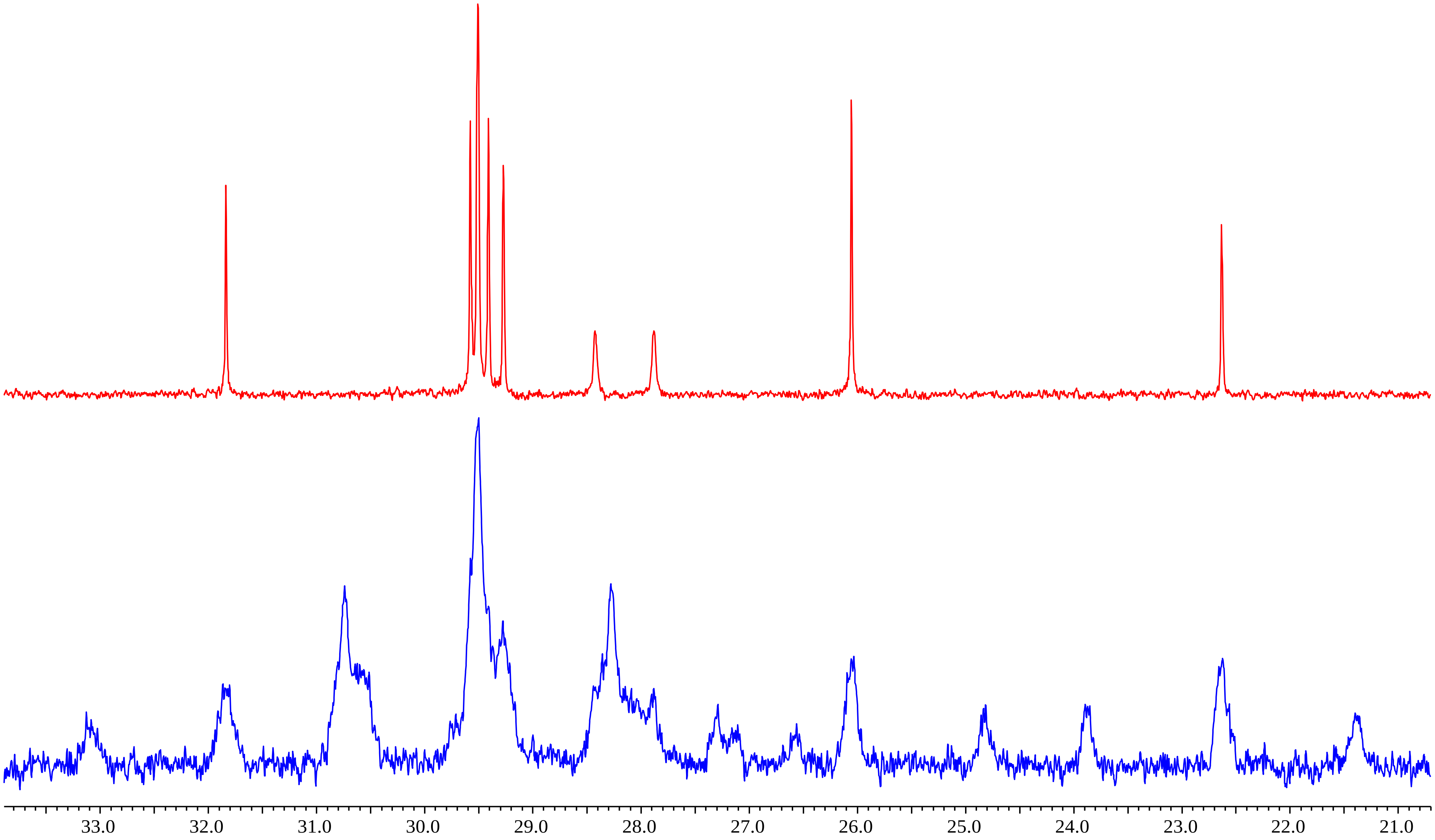

Figure 123. High-field fragment of ^{13}C-{^{1}H} and ^{13}C NMR spectra (100.6 MHz, $CDCl_3$) of compound (**3j**).

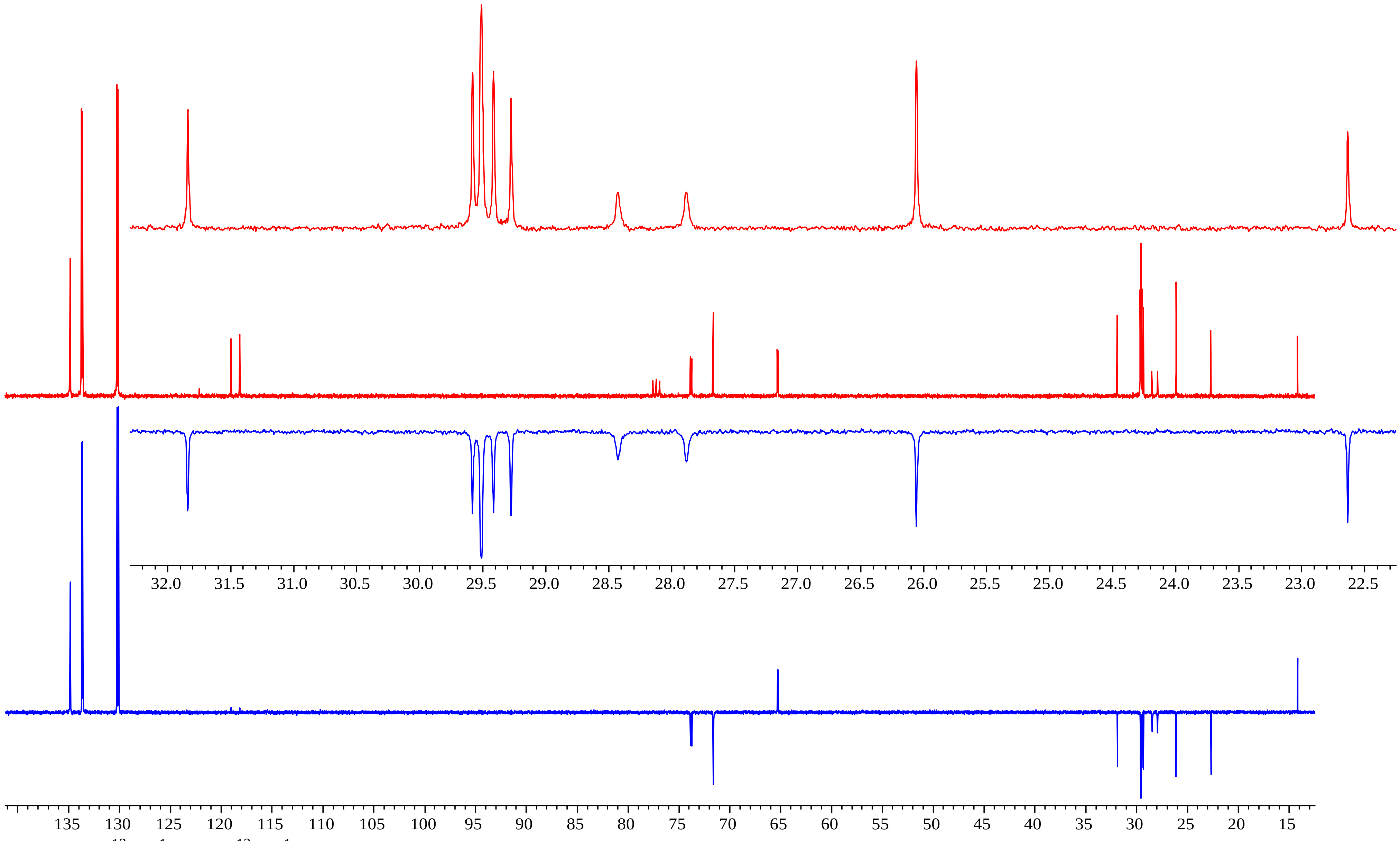

Figure 124. ^{13}C-{^{1}H} and ^{13}C-{^{1}H}-dept NMR spectra (100.6 MHz, $CDCl_3$) of compound (**3j**).

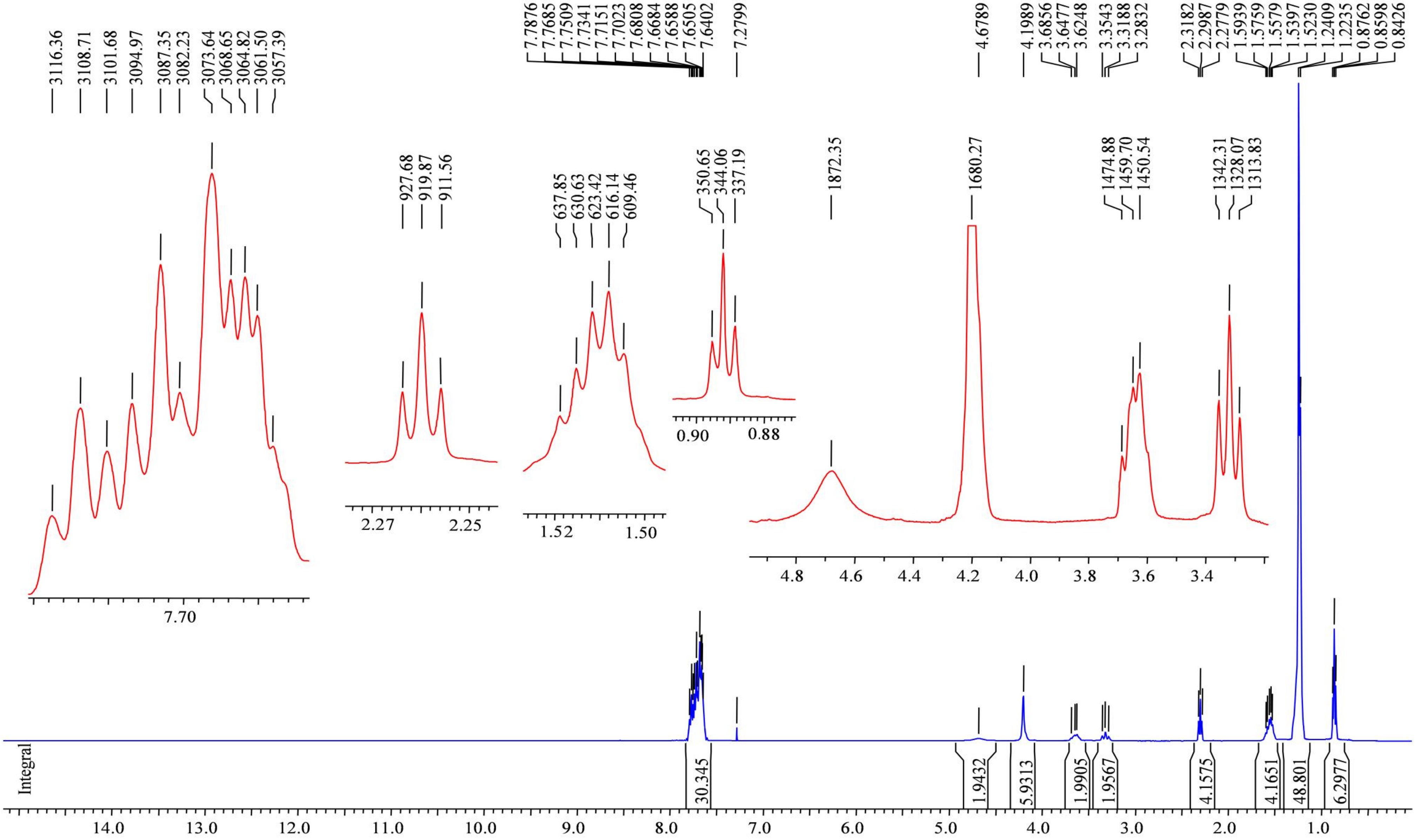

Figure 125. ^{1}H NMR spectrum (400 MHz, $CDCl_3$) of compound (**3k**).

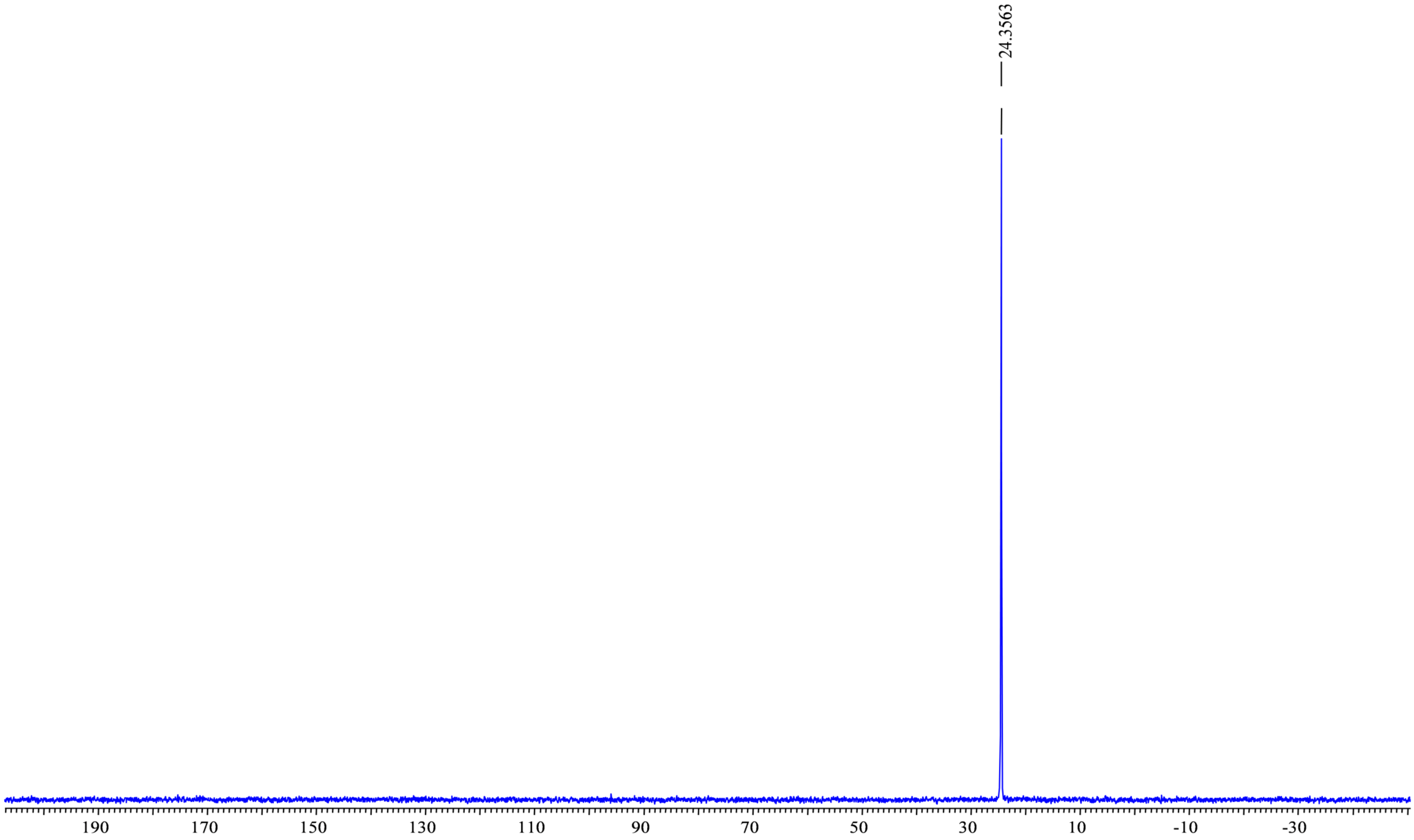

Figure 126. ^{31}P-{^{1}H} NMR spectrum (162.0 MHz, $CDCl_3$) of compound (**3k**).

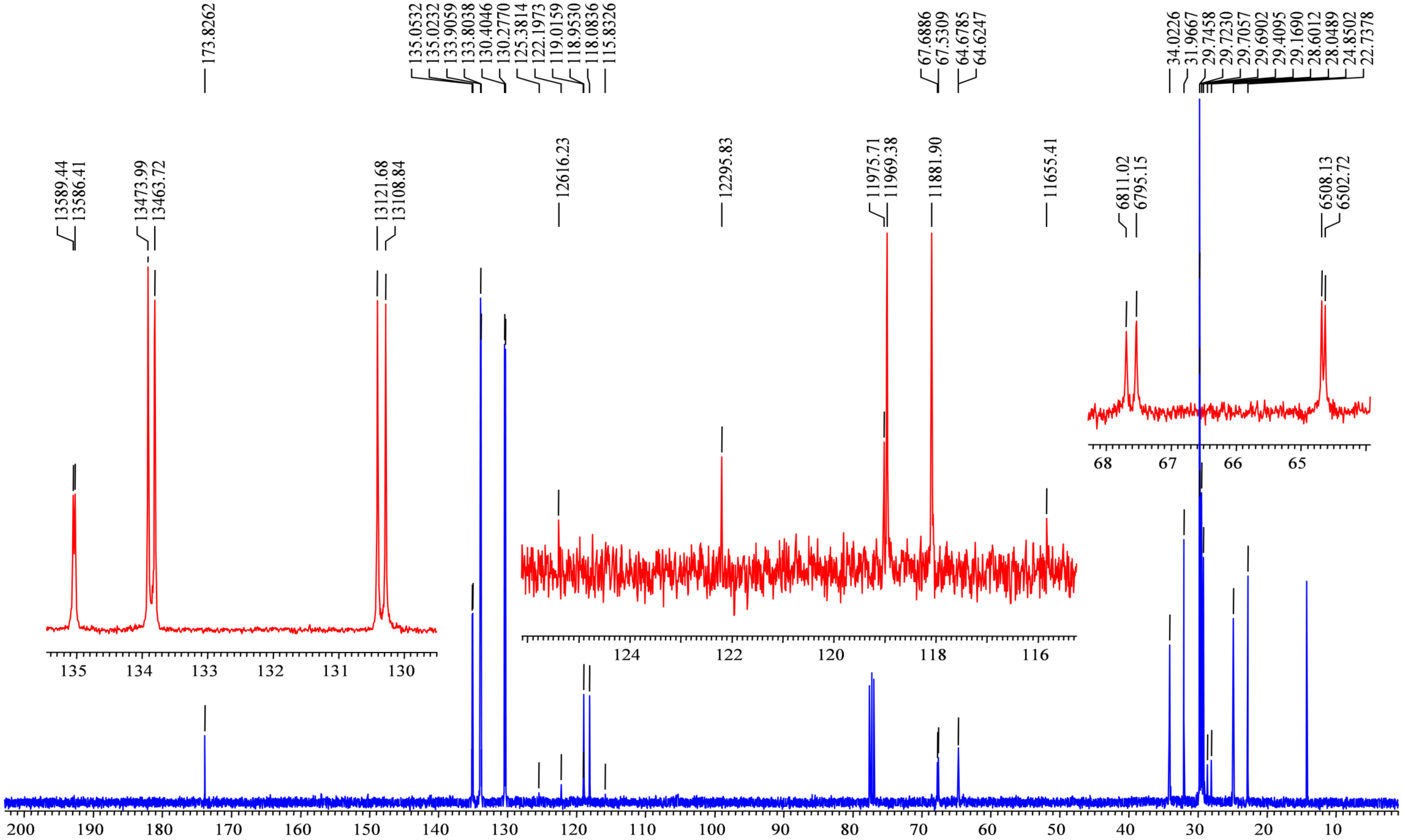

Figure 127. ^{13}C-{^{1}H} NMR spectrum (100.6 MHz, $CDCl_3$) of compound (**3k**).

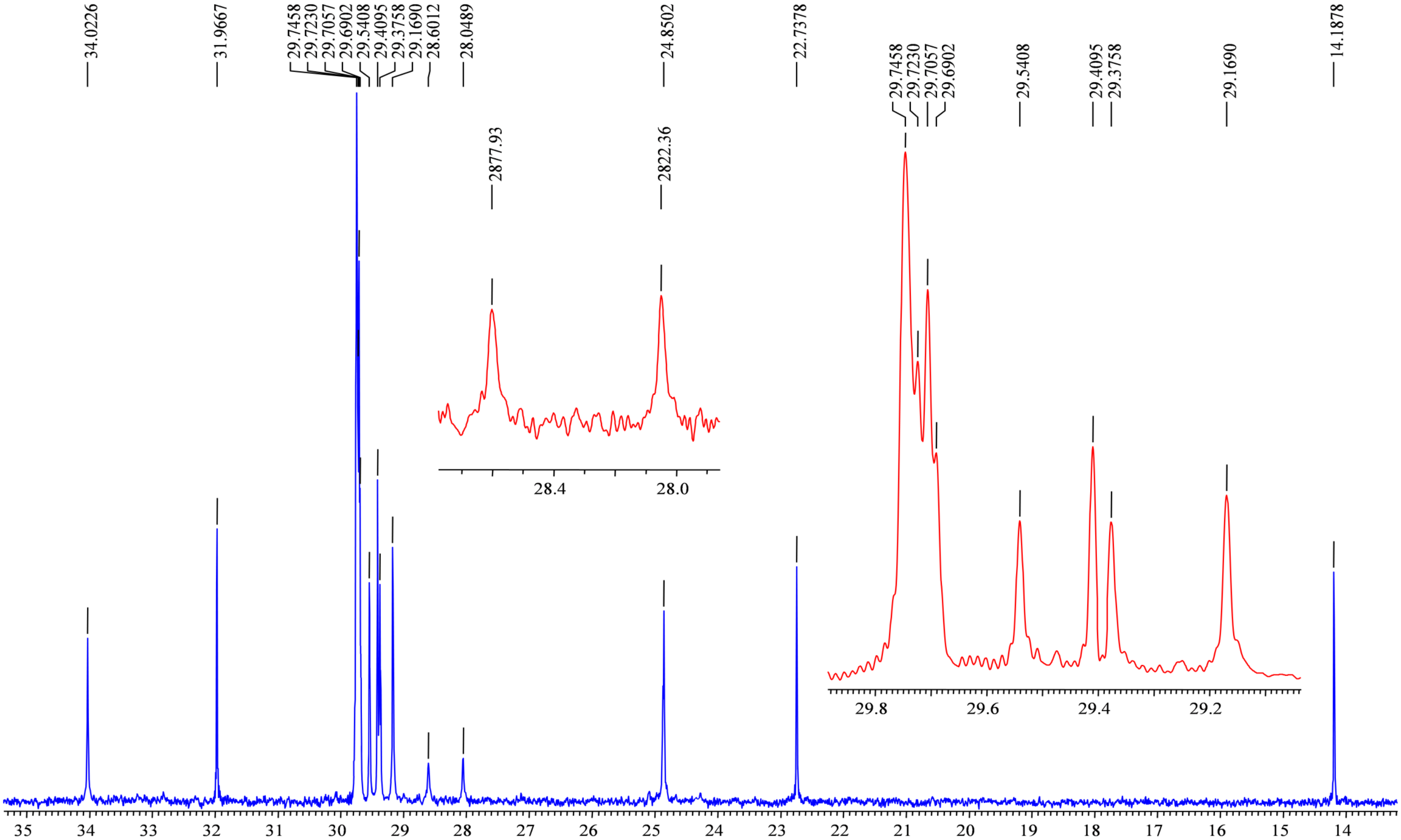

Figure 128. High-field fragment of ^{13}C-{^{1}H} NMR spectrum (100.6 MHz, $CDCl_3$) of compound (**3k**).

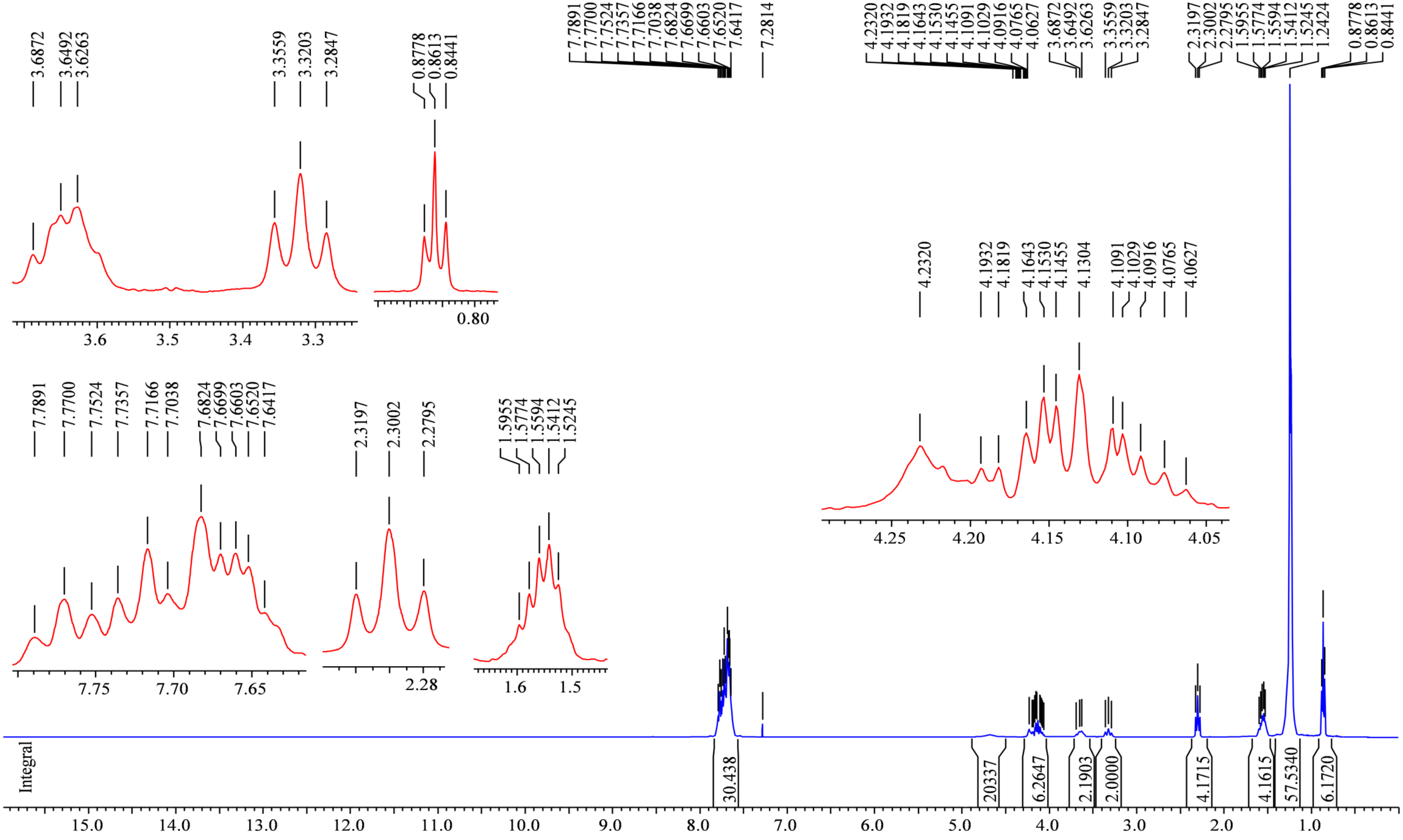

Figure 129. ^{1}H NMR spectrum (400 MHz, $CDCl_3$) of compound (**3l**).

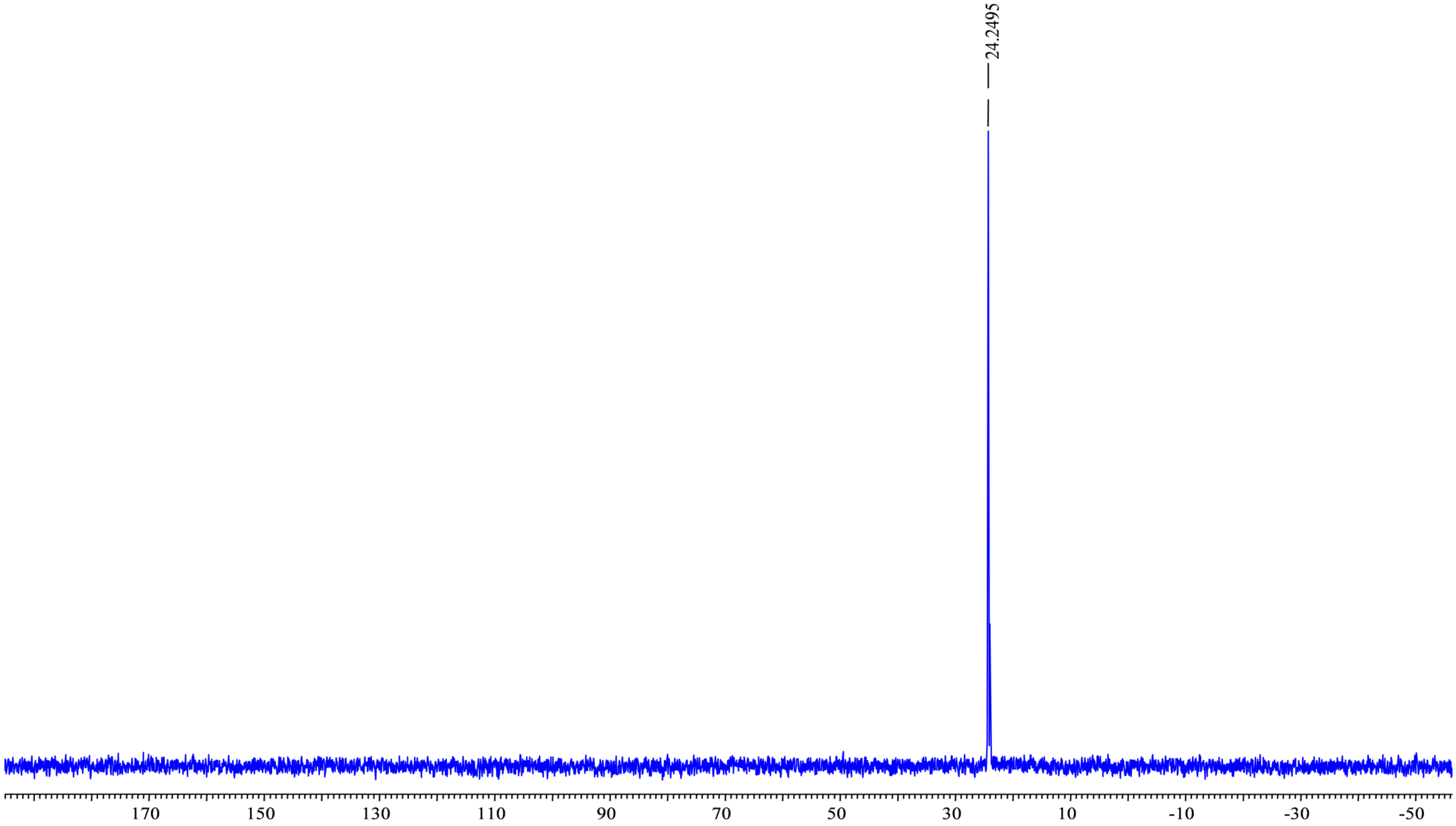

Figure 130. ^{31}P-{^{1}H} NMR spectrum (162.0 MHz, $CDCl_3$) of compound (**3l**).

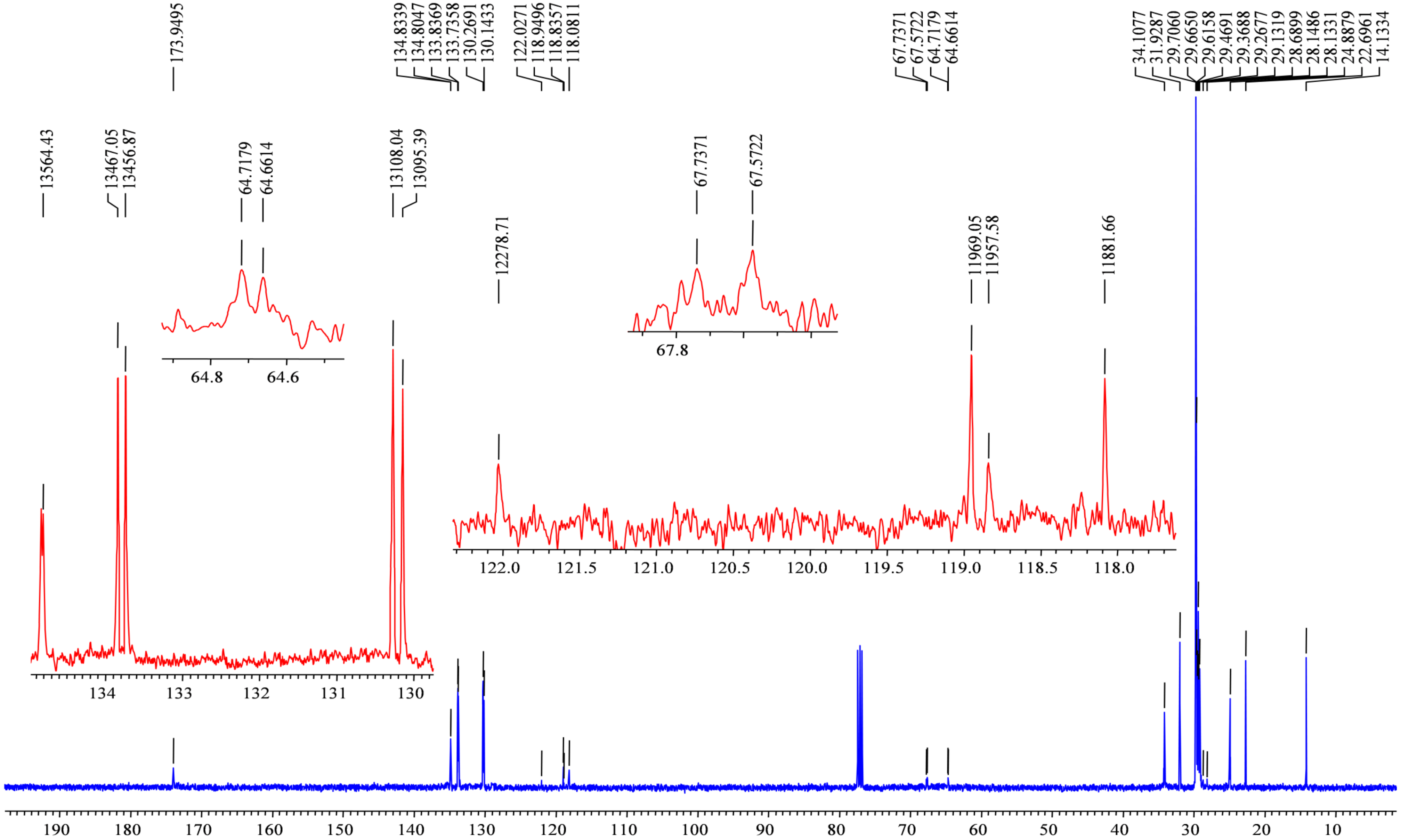

Figure 131. ^{13}C-{^{1}H} NMR spectrum (100.6 MHz, $CDCl_3$) of compound (**3l**)..

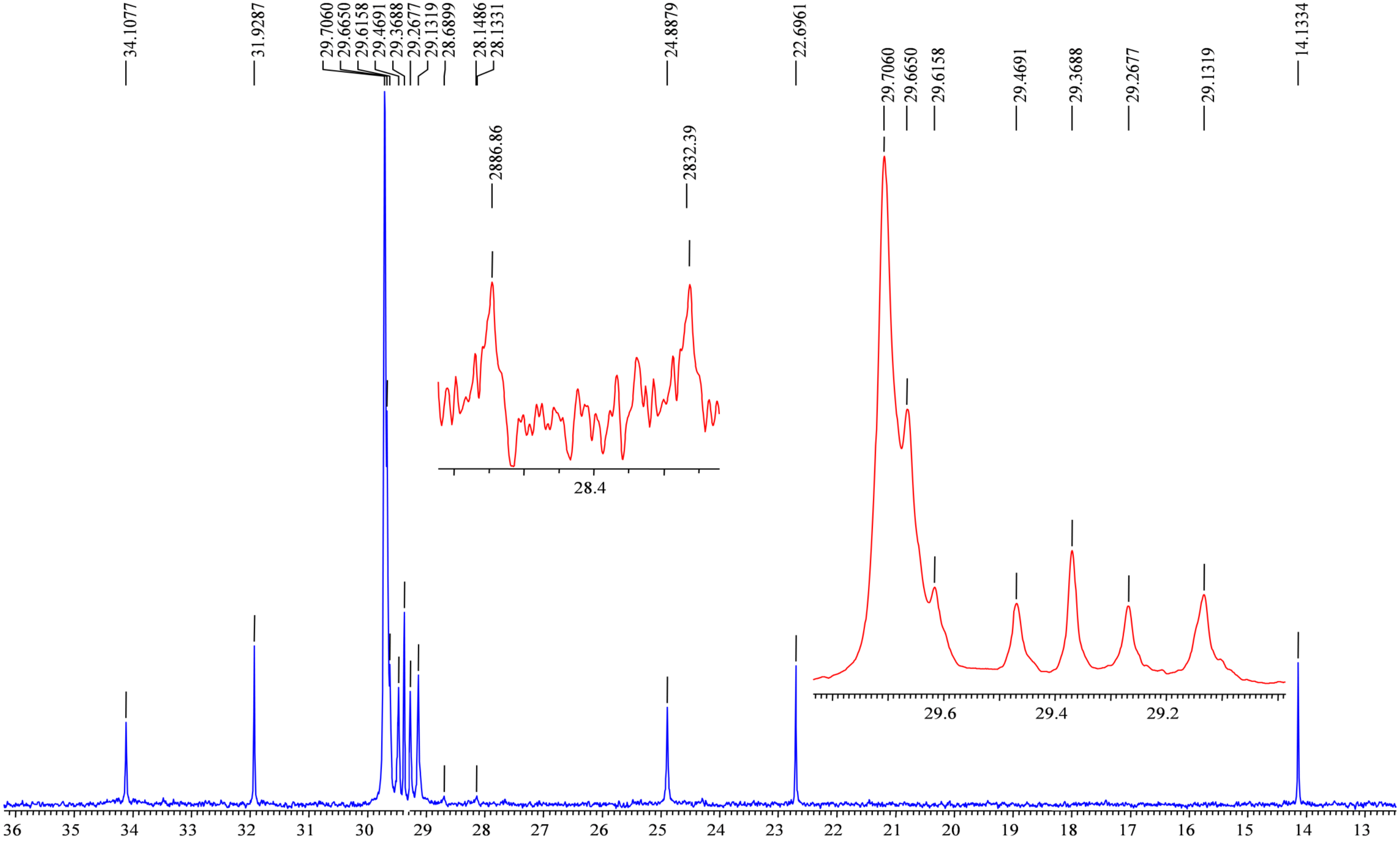

Figure 132. High-field fragment of ^{13}C-{^1H} NMR spectrum (100.6 MHz, $CDCl_3$) of compound (**3l**).

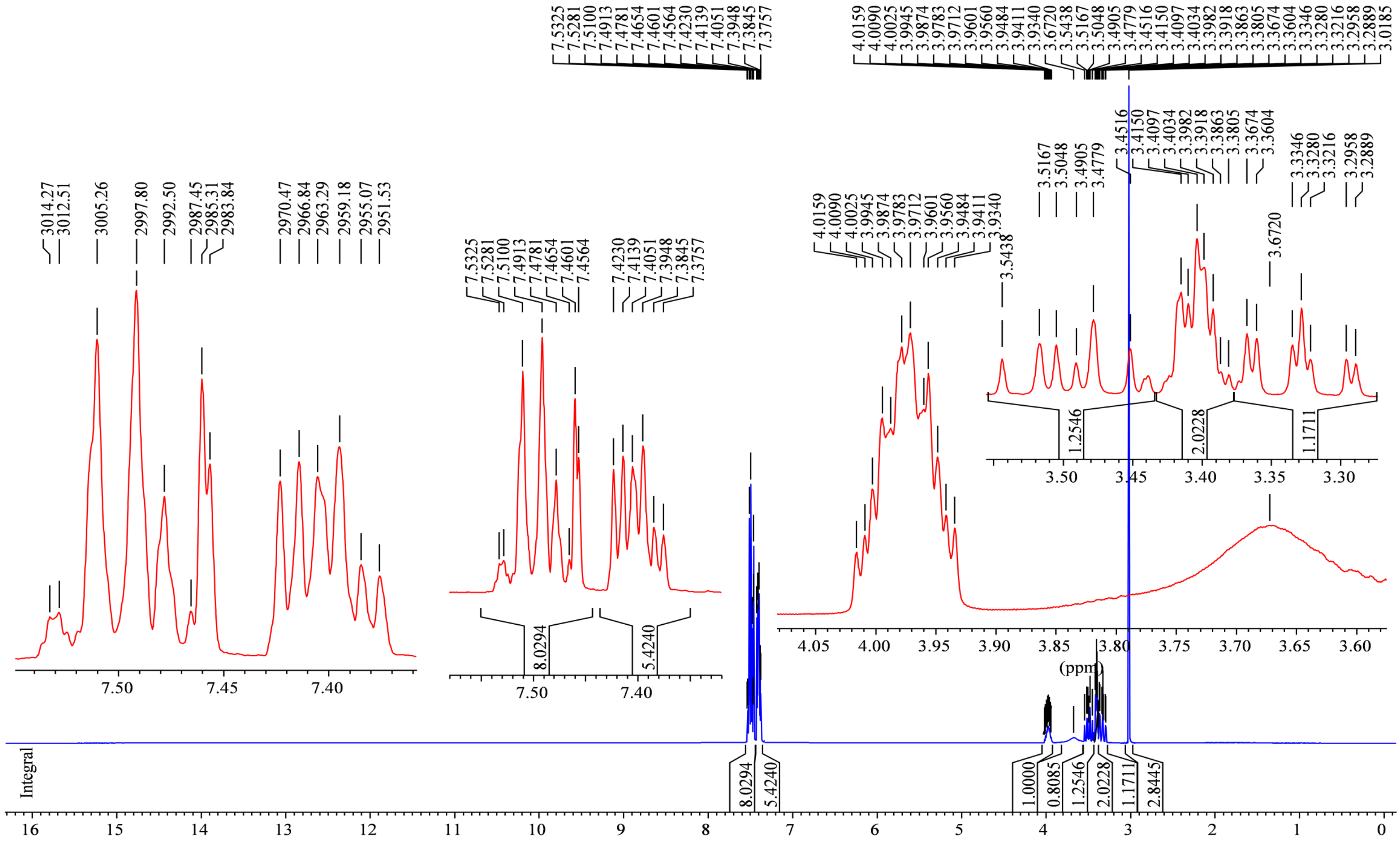

Figure 133. ^{1}H NMR spectrum (400 MHz, $CDCl_3$) of compound (**4a**).

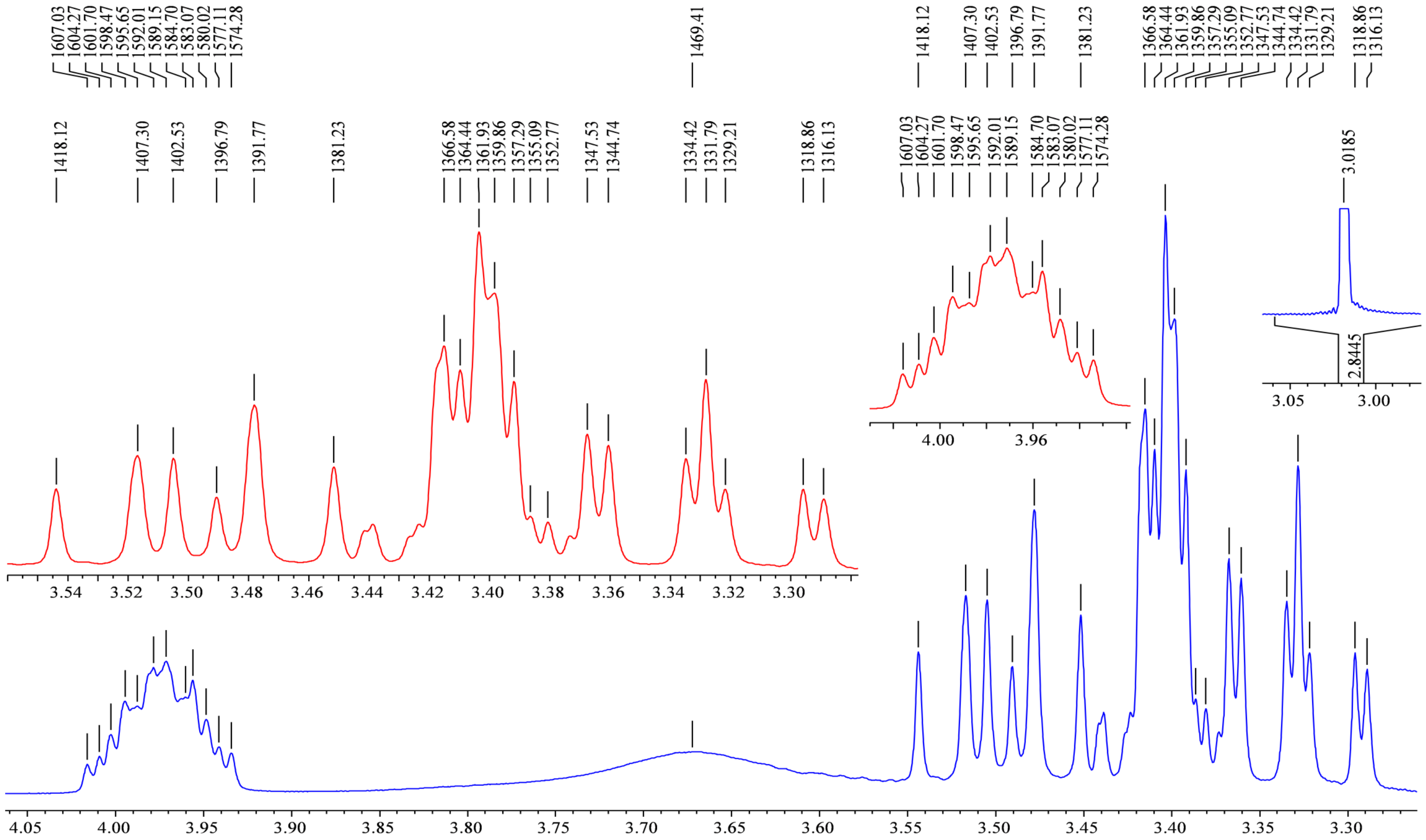

Figure 134. The fragment of ^{1}H NMR spectrum (400 MHz, $CDCl_3$) of compound (**4a**).

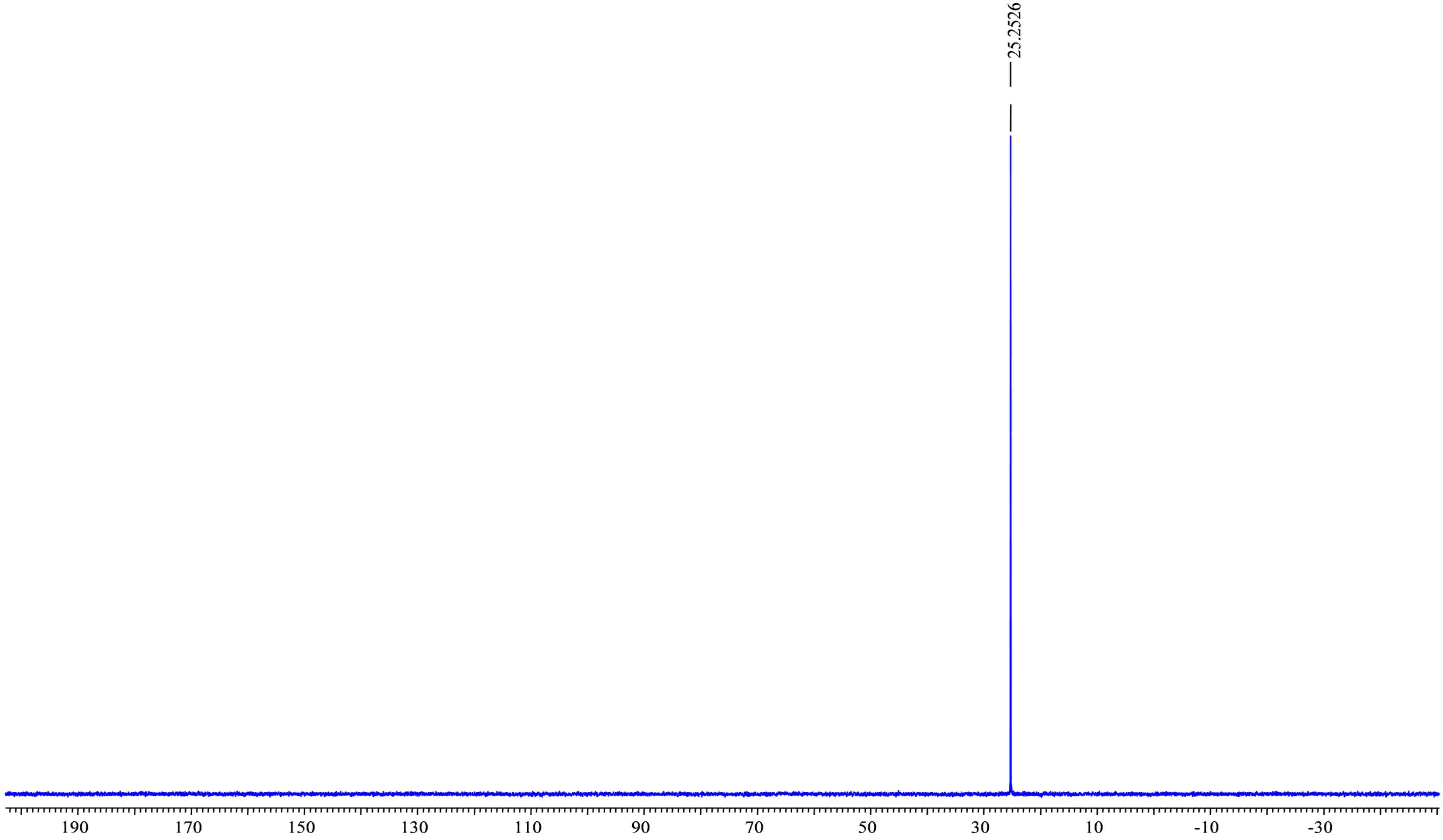

Figure 135. ^{31}P-{^{1}H} NMR spectrum (162.0 MHz, CH_3CN) of compound (**4a**).

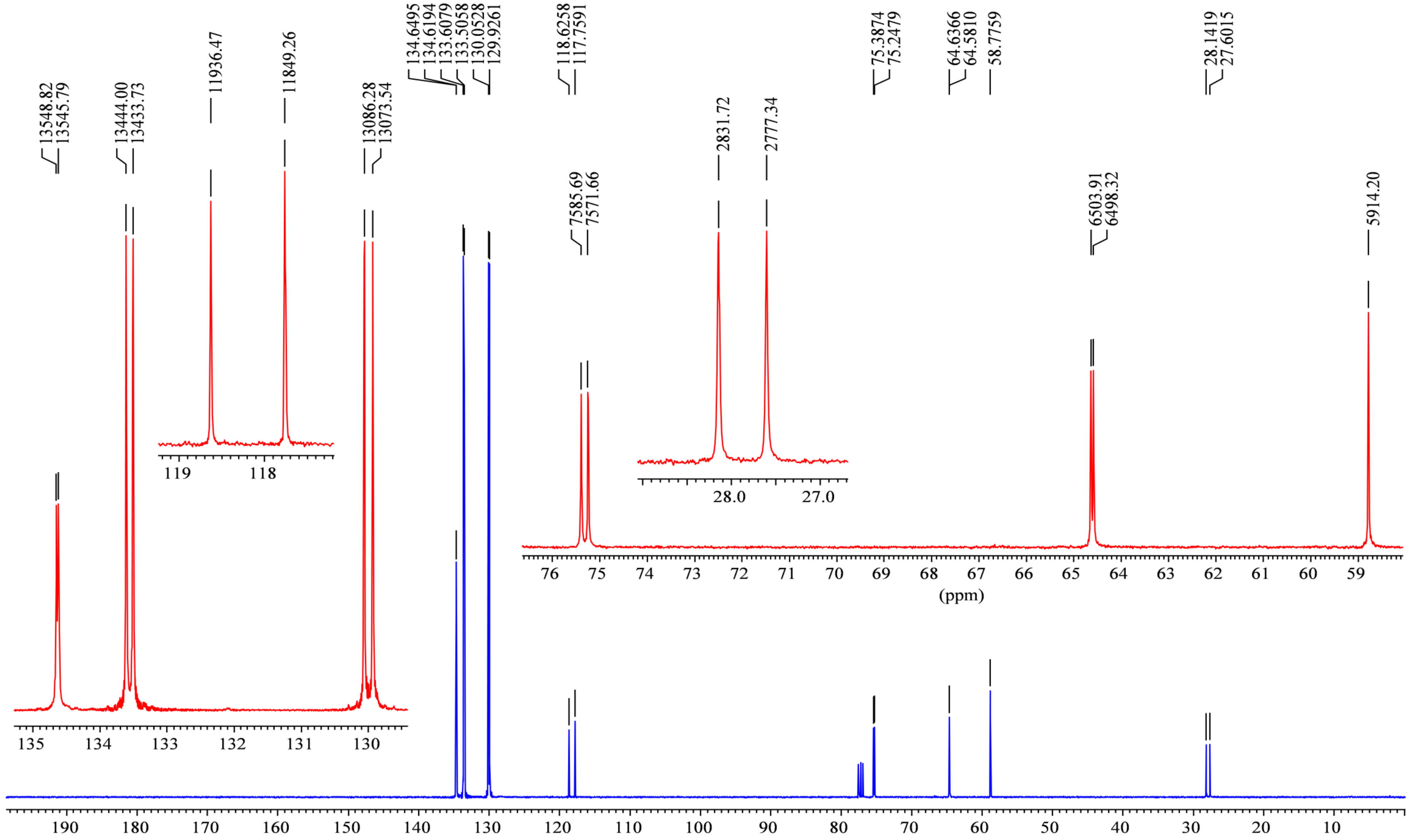

Figure 136. ^{13}C-{^{1}H} NMR spectrum (100.6 MHz, $CDCl_3$) of compound (**4a**).

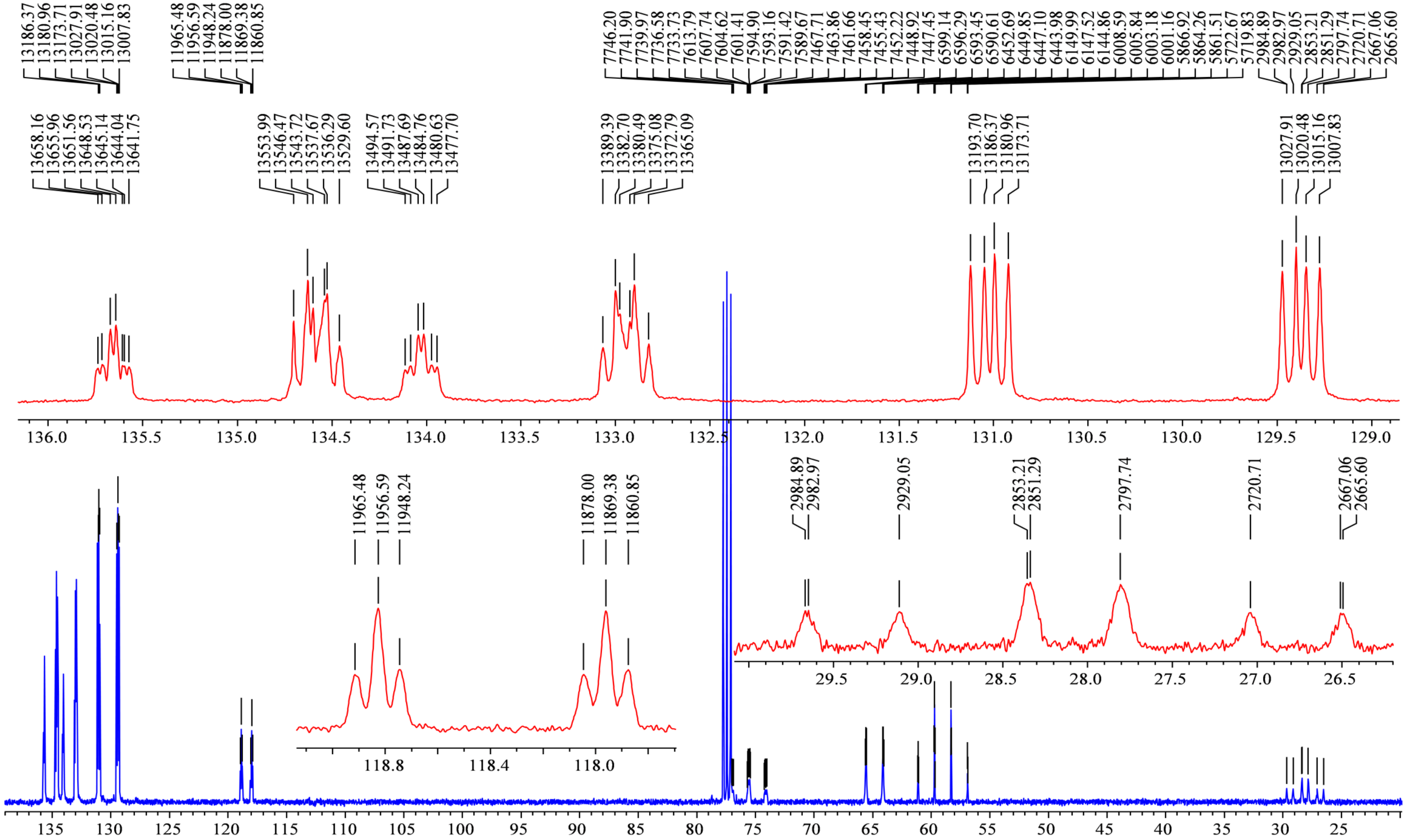

Figure 137. ^{13}C NMR spectrum (100.6 MHz, $CDCl_3$) of compound (**4a**).

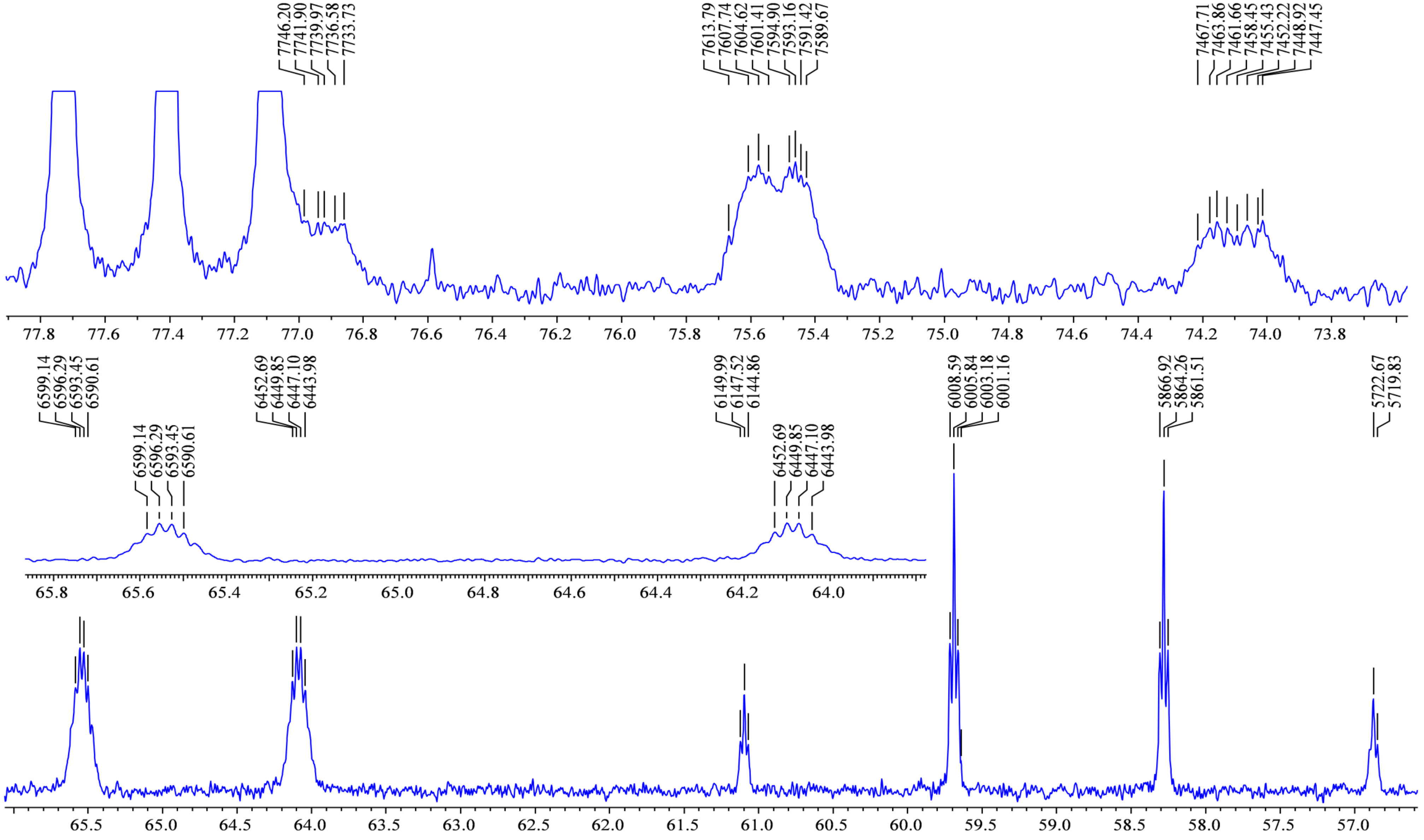

Figure 138. The 56-66 ppm region of ^{13}C NMR spectrum (100.6 MHz, $CDCl_3$) of compound (**4a**).

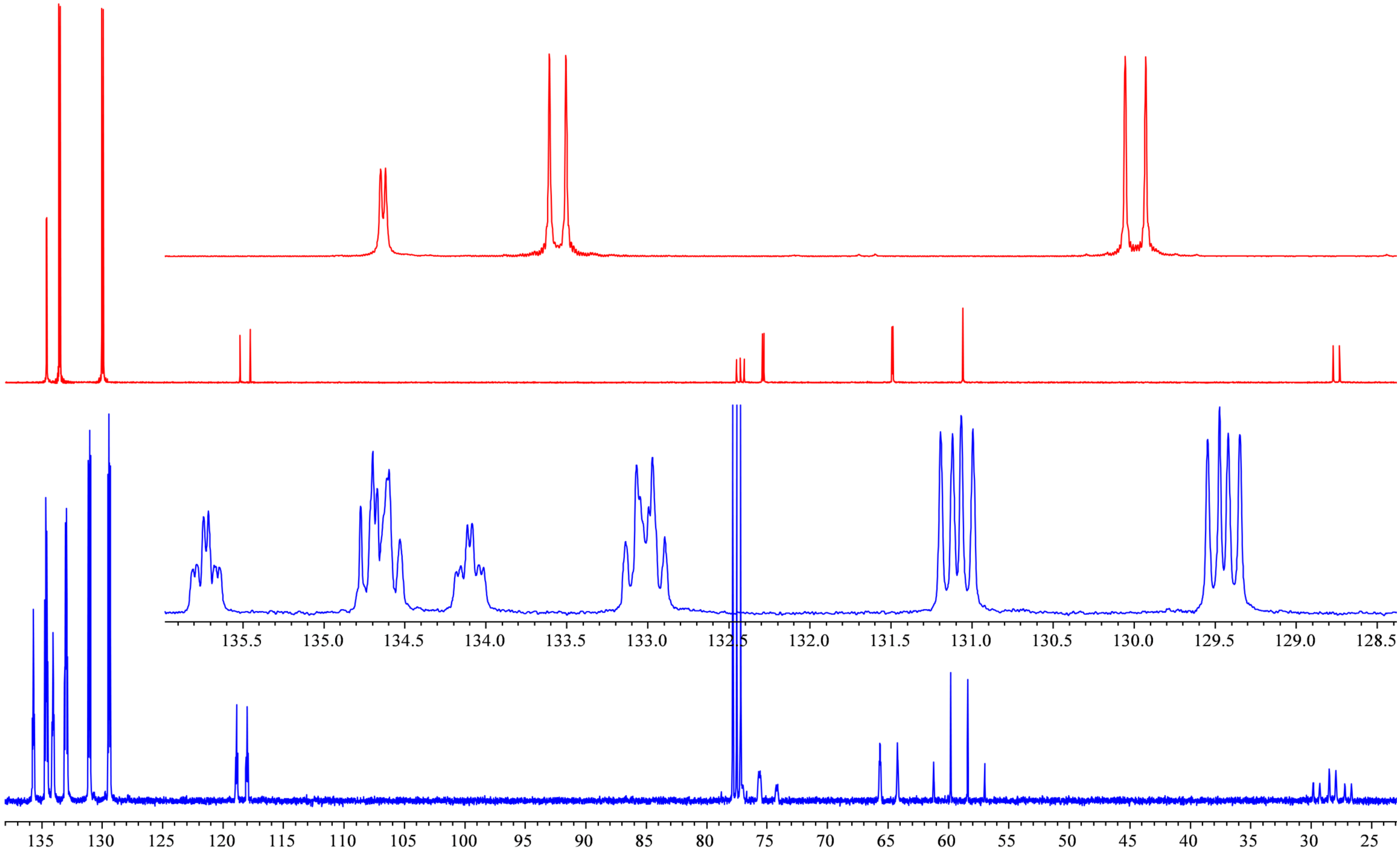

Figure 139. ^{13}C and ^{13}C -{^{1}H} NMR spectra (100.6 MHz, $CDCl_3$) of compound (**4a**).

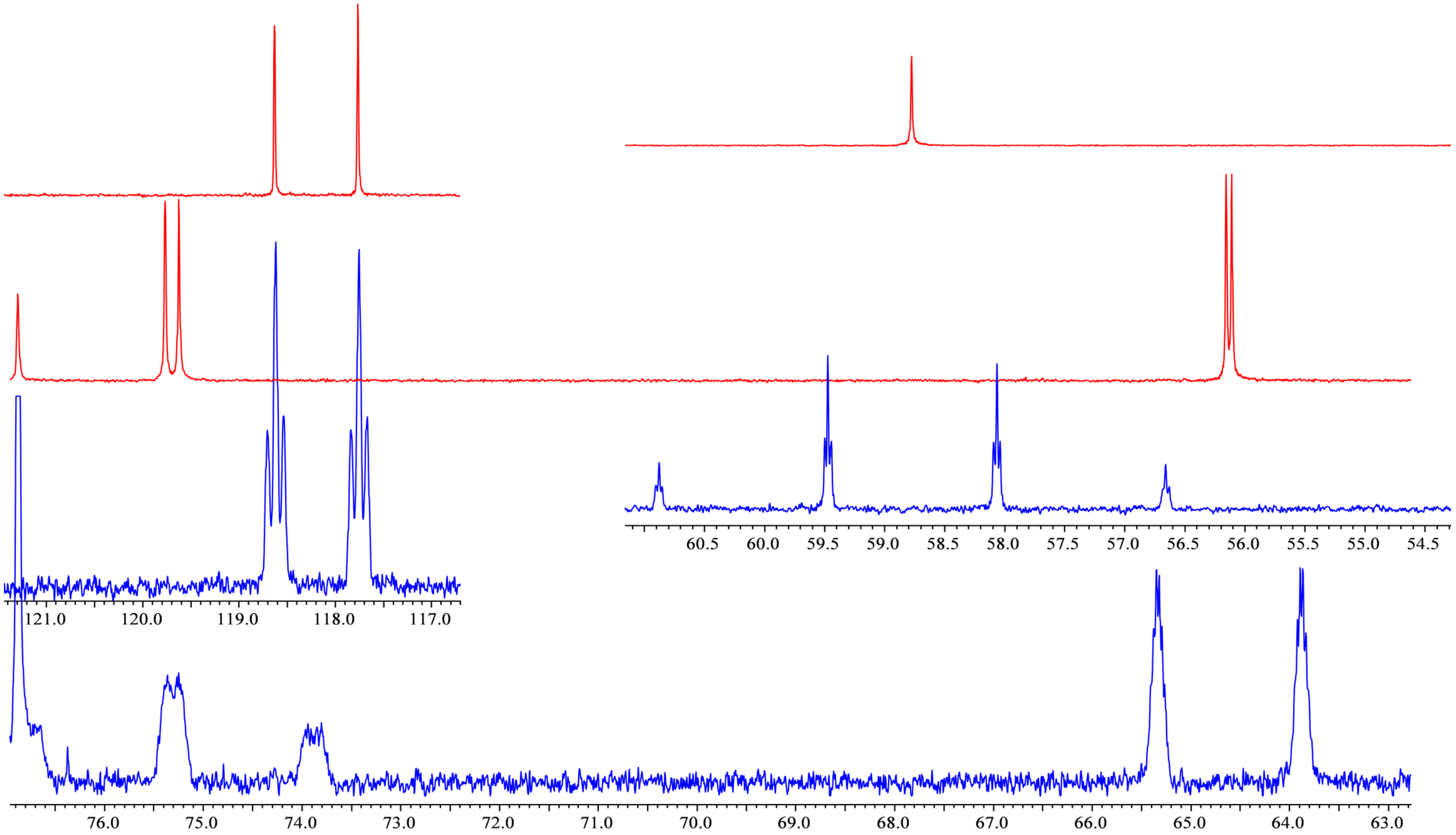

Figure 140. The 104-126 ppm region of ^{13}C-{^{1}H} and ^{13}C NMR spectra (100.6 MHz, $CDCl_3$) of compound (**4a**).

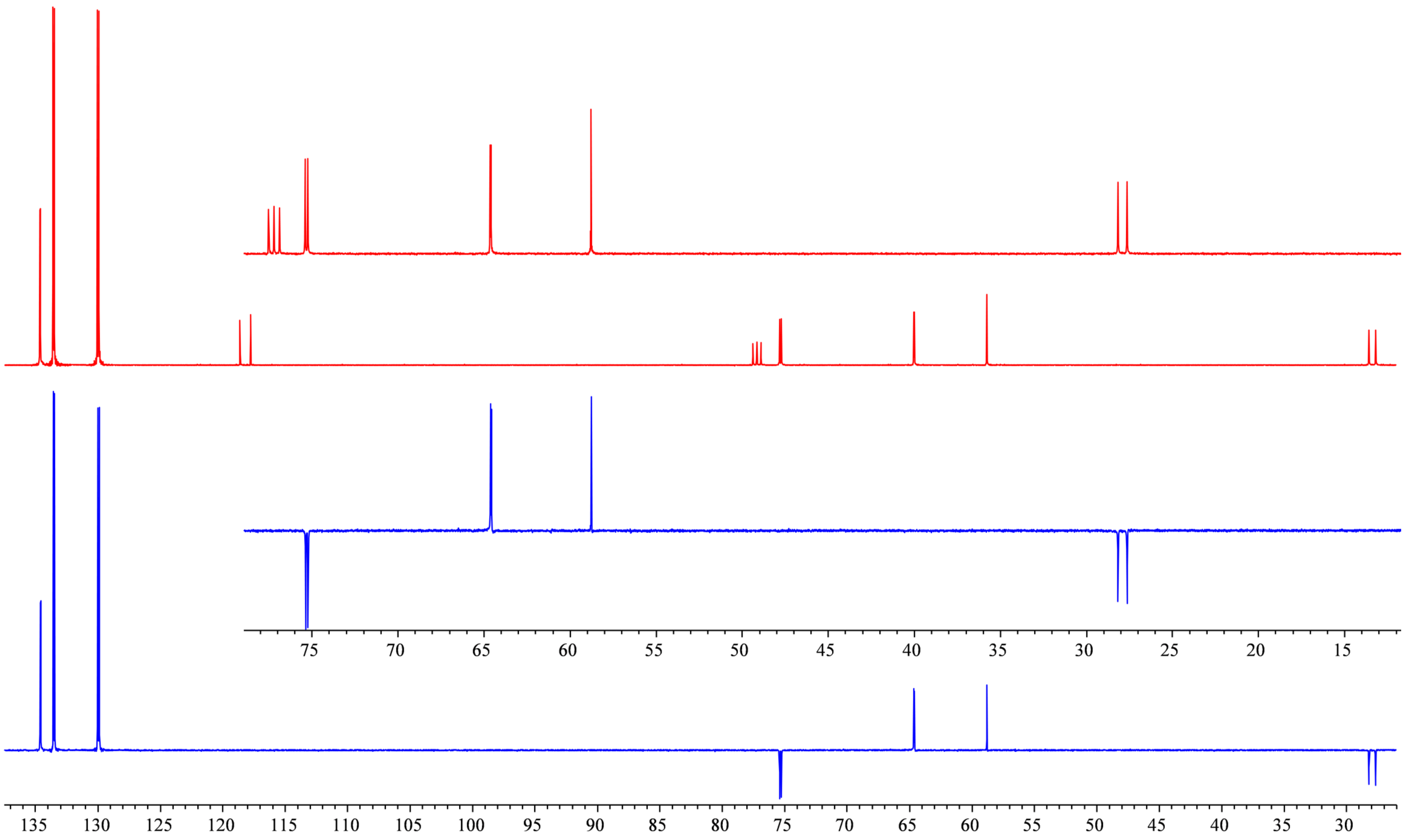

Figure 141. ^{13}C-{^{1}H} and ^{13}C-{^{1}H}-dept NMR spectra (100.6 MHz, $CDCl_3$) of compound (**4a**).

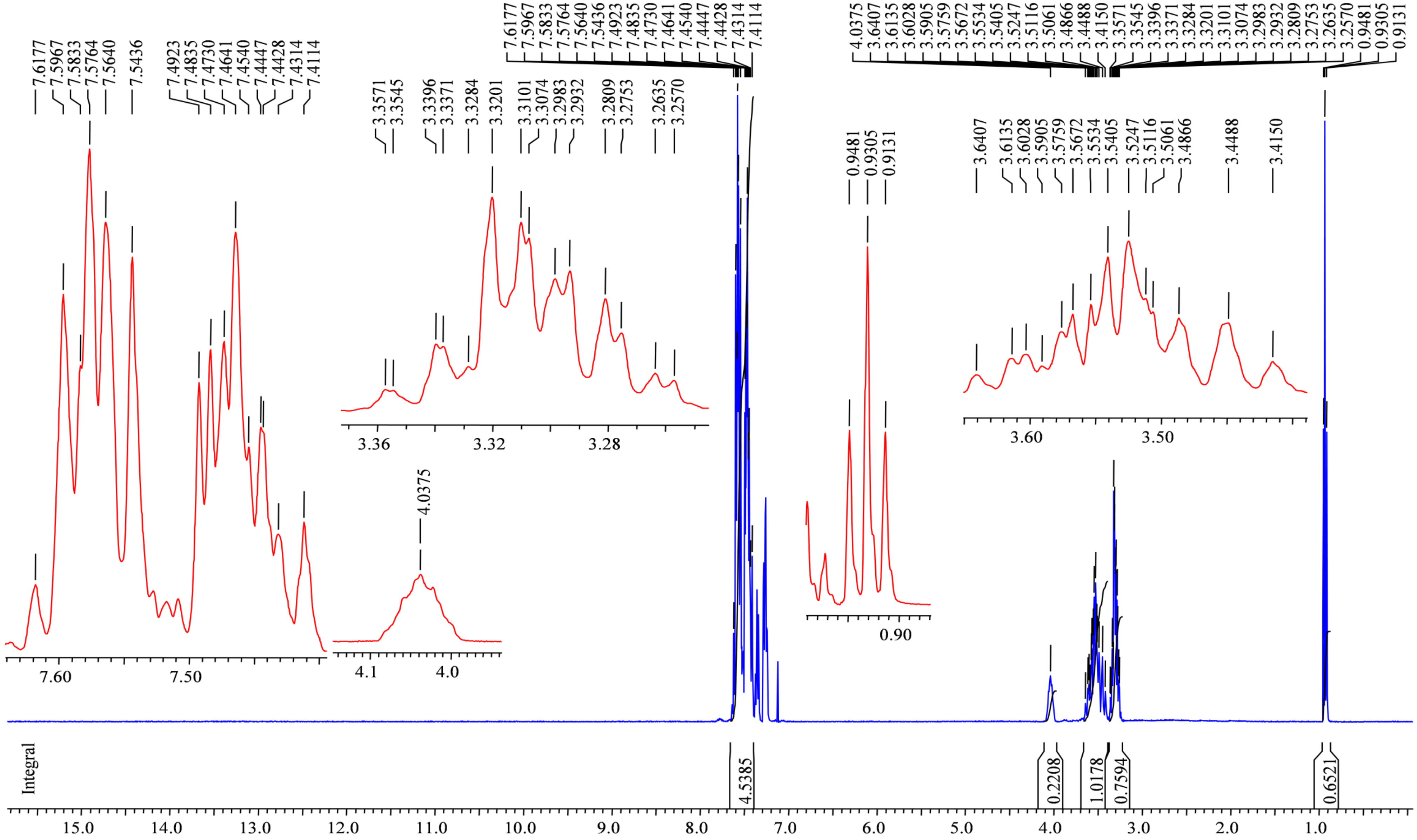

Figure 142. ^{1}H NMR spectrum (400 MHz, $CDCl_3$) of compound (**4b**).

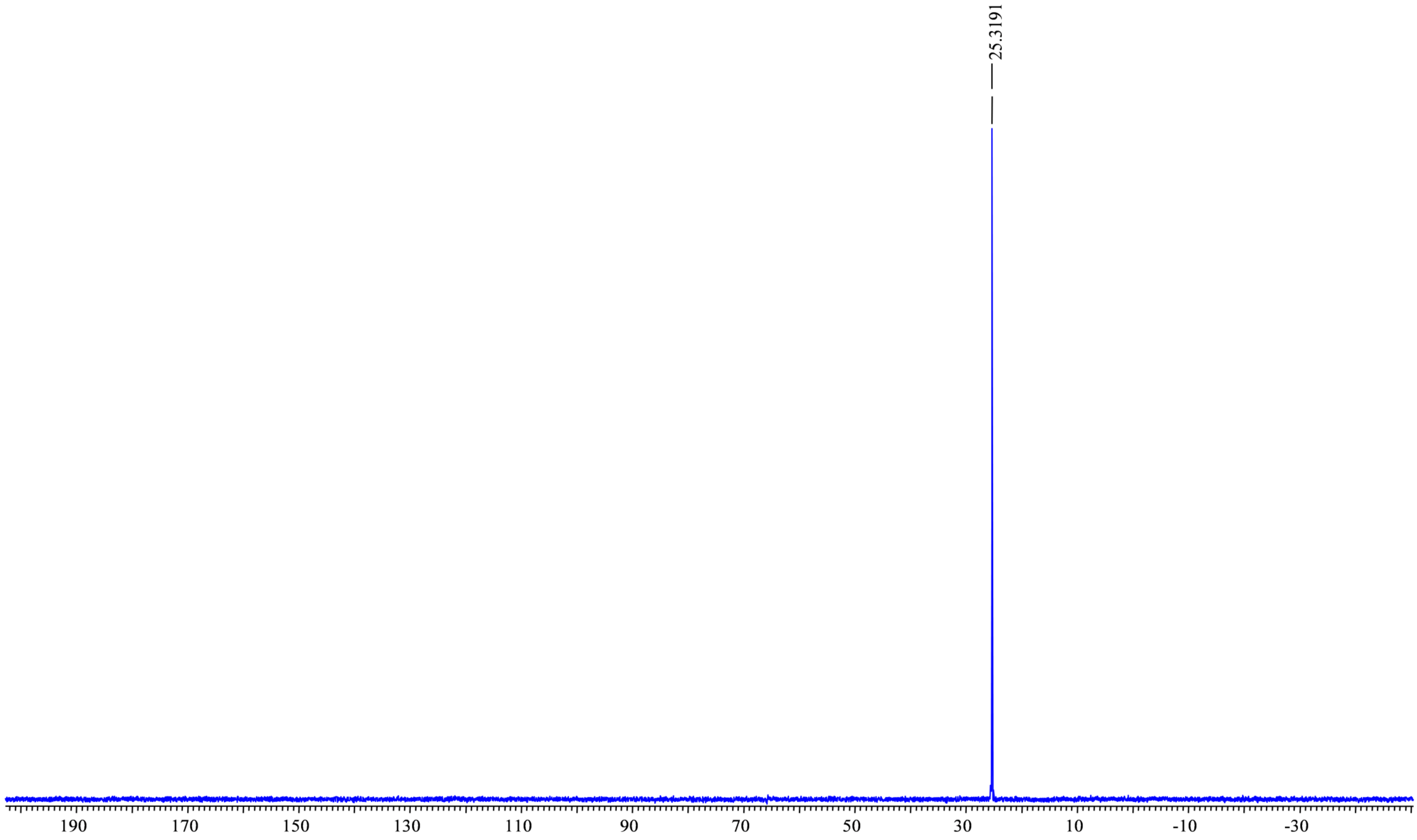

Figure 143. ^{31}P-{^{1}H} NMR spectrum (162.0 MHz, CH_3CN) of compound (**4b**).

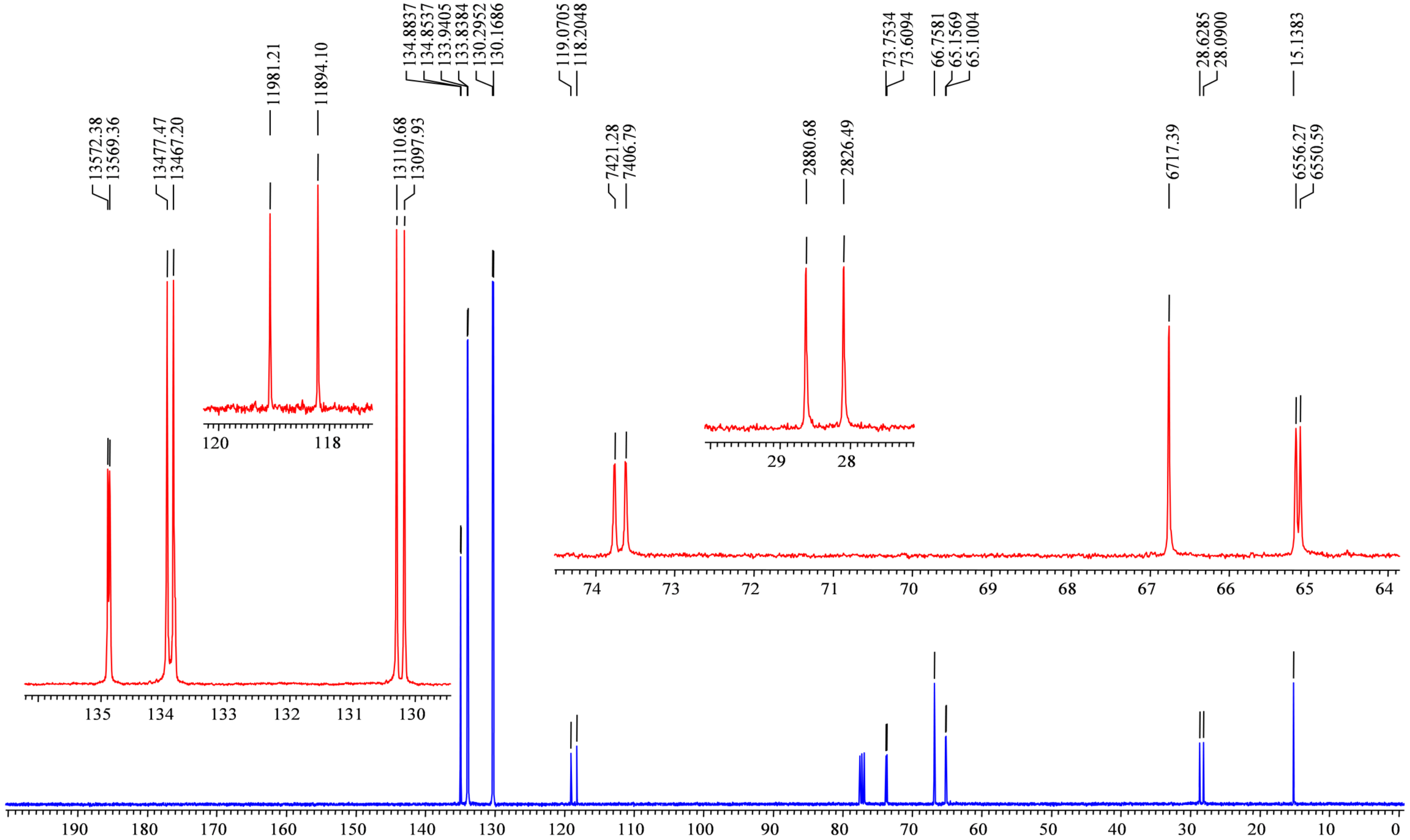

Figure 144. ^{13}C-{^{1}H} NMR spectrum (100.6 MHz, $CDCl_3$) of compound (**4b**).

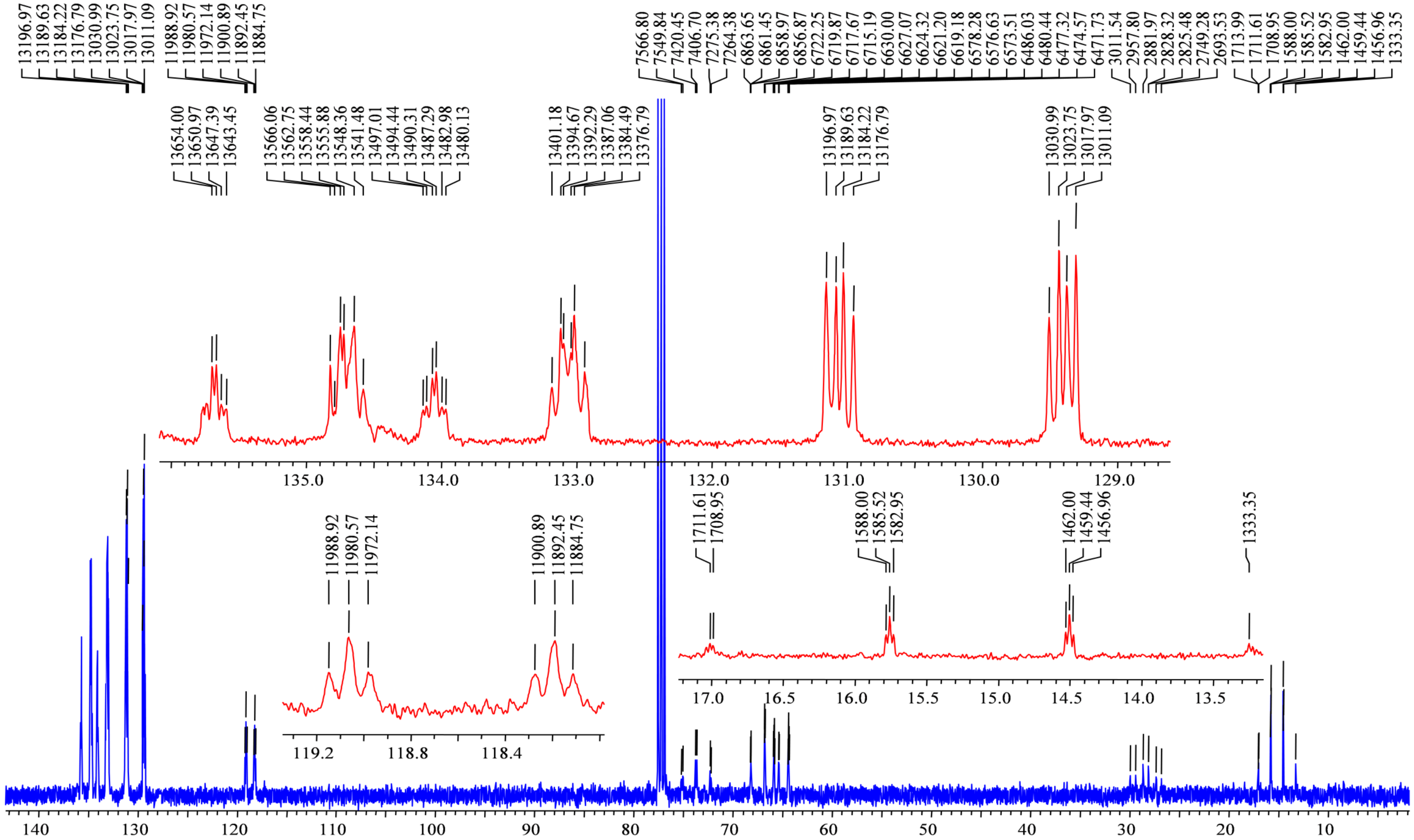

Figure 145. ^{13}C NMR spectrum (100.6 MHz, $CDCl_3$) of compound (**4b**).

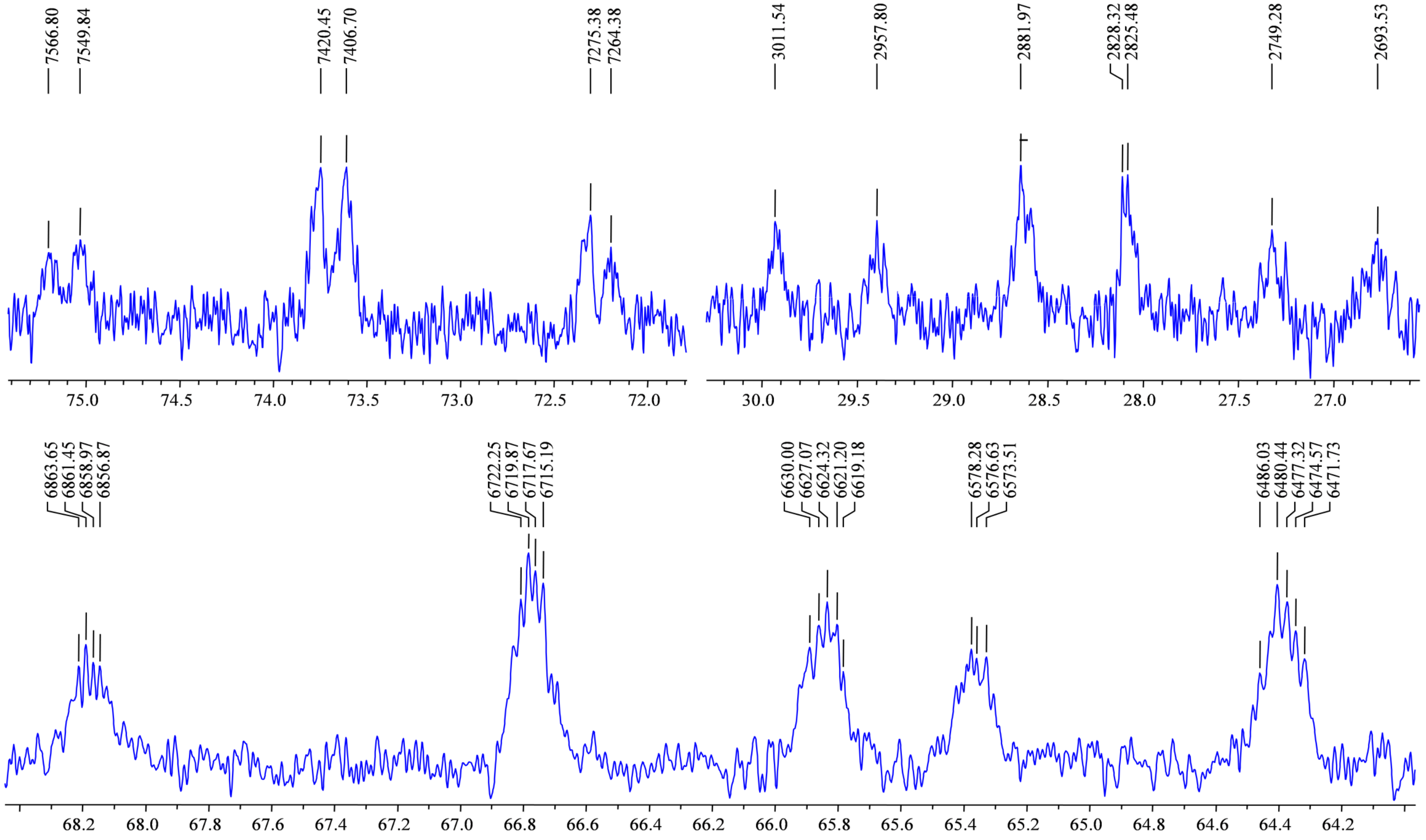

Figure 146. The 26-30, 64-69 and 72-76 ppm regions of ^{13}C NMR spectrum (100.6 MHz, $CDCl_3$) of compound (**4b**).

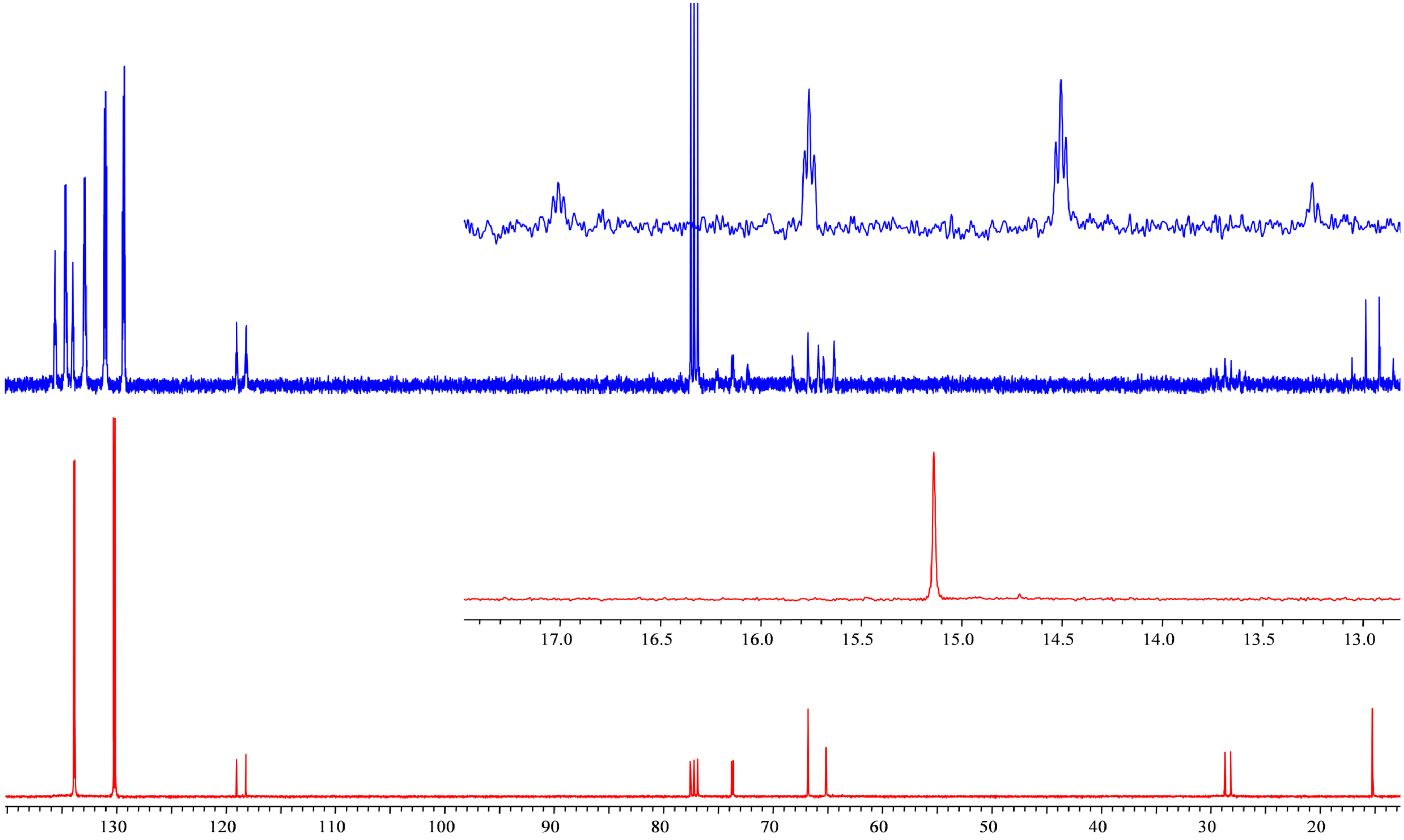

Figure 147. ^{13}C and $^{13}C-\{^1H\}$ NMR spectra (100.6 MHz, $CDCl_3$) of compound (**4b**).

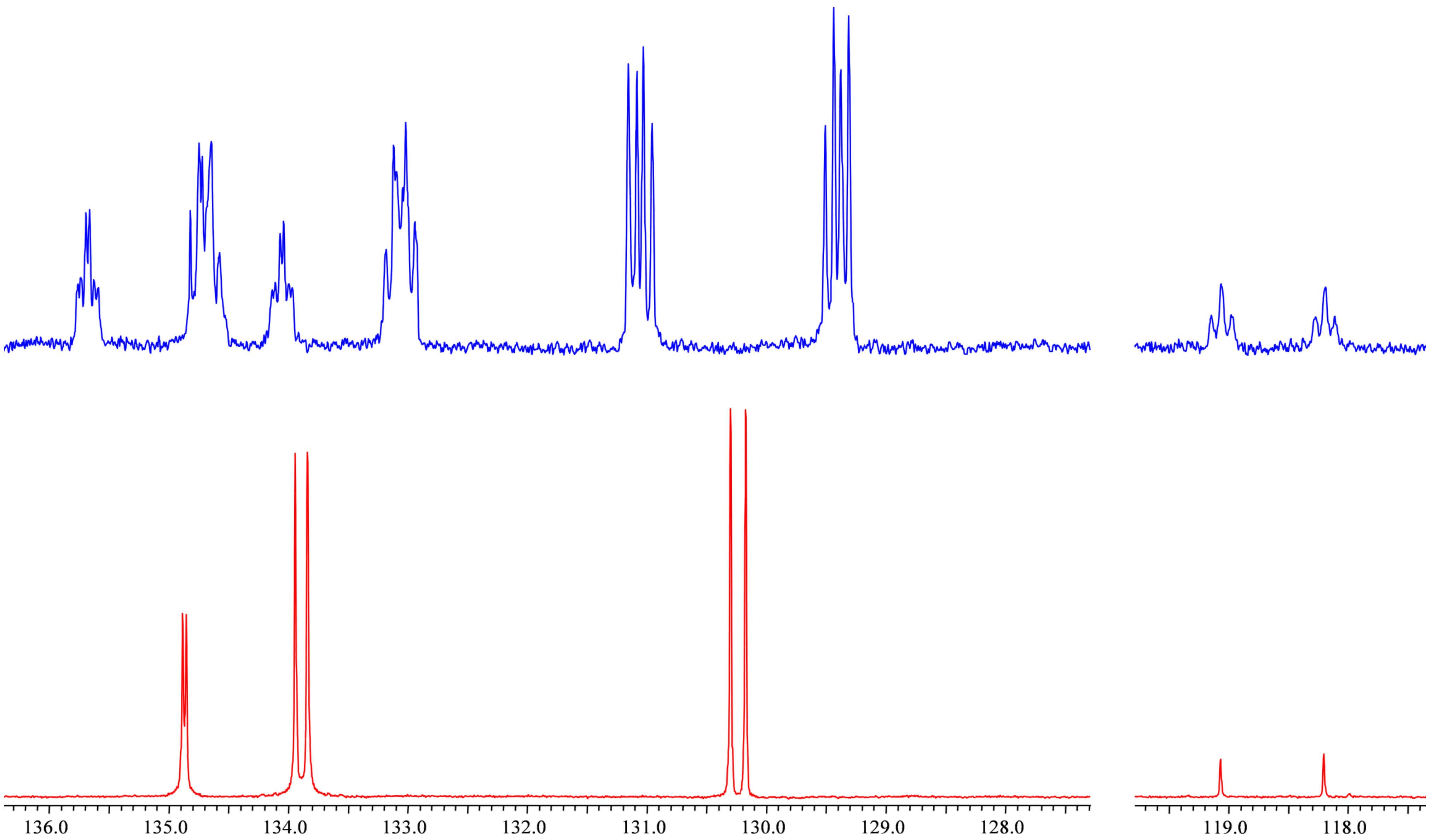

Figure 148. The 117-136 ppm region of ^{13}C-{^{1}H} and ^{13}C NMR spectra (100.6 MHz, $CDCl_3$) of compound (**4b**).

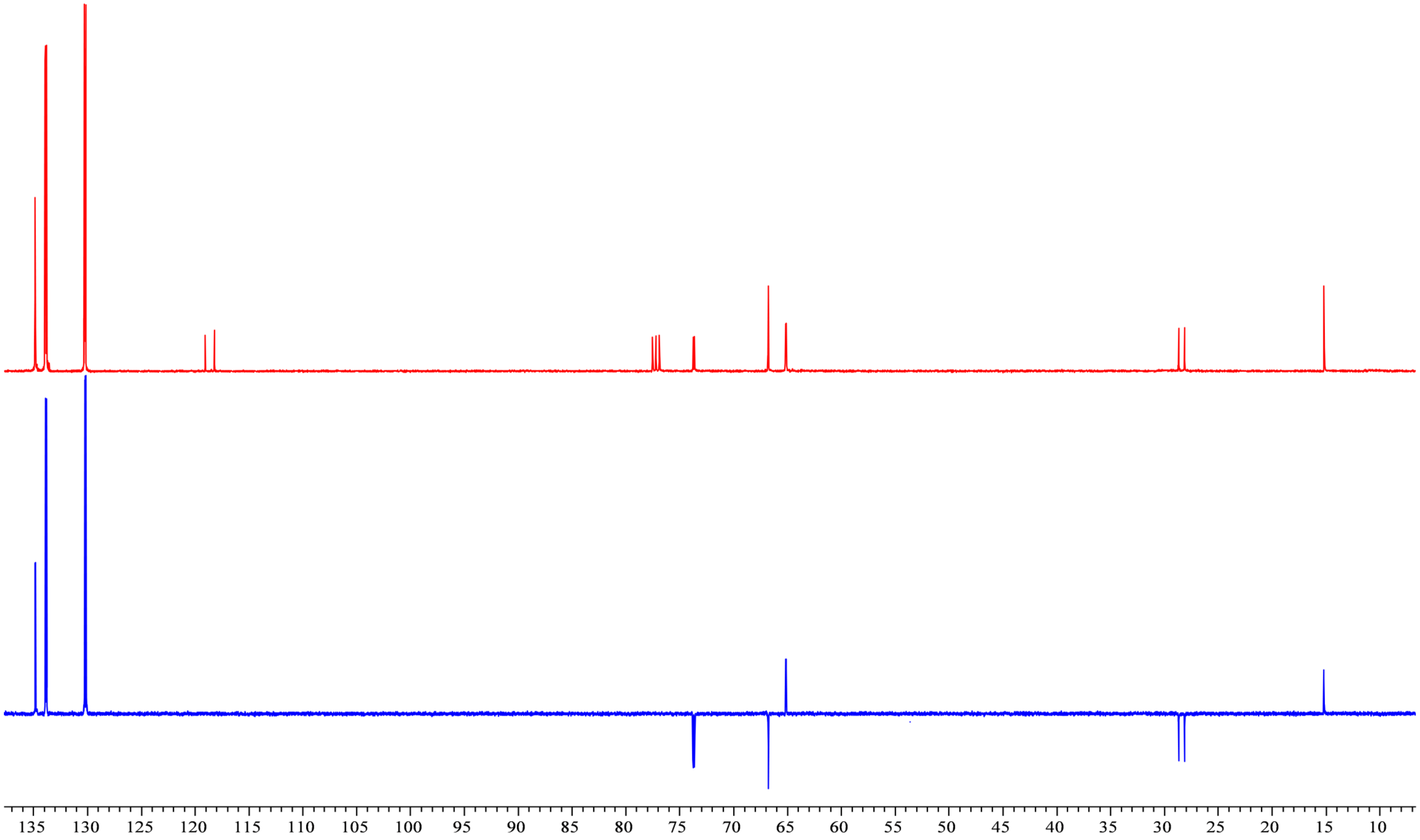

Figure 149. ^{13}C-{^{1}H} and ^{13}C-{^{1}H}-dept NMR spectra (100.6 MHz, $CDCl_3$) of compound (**4b**).

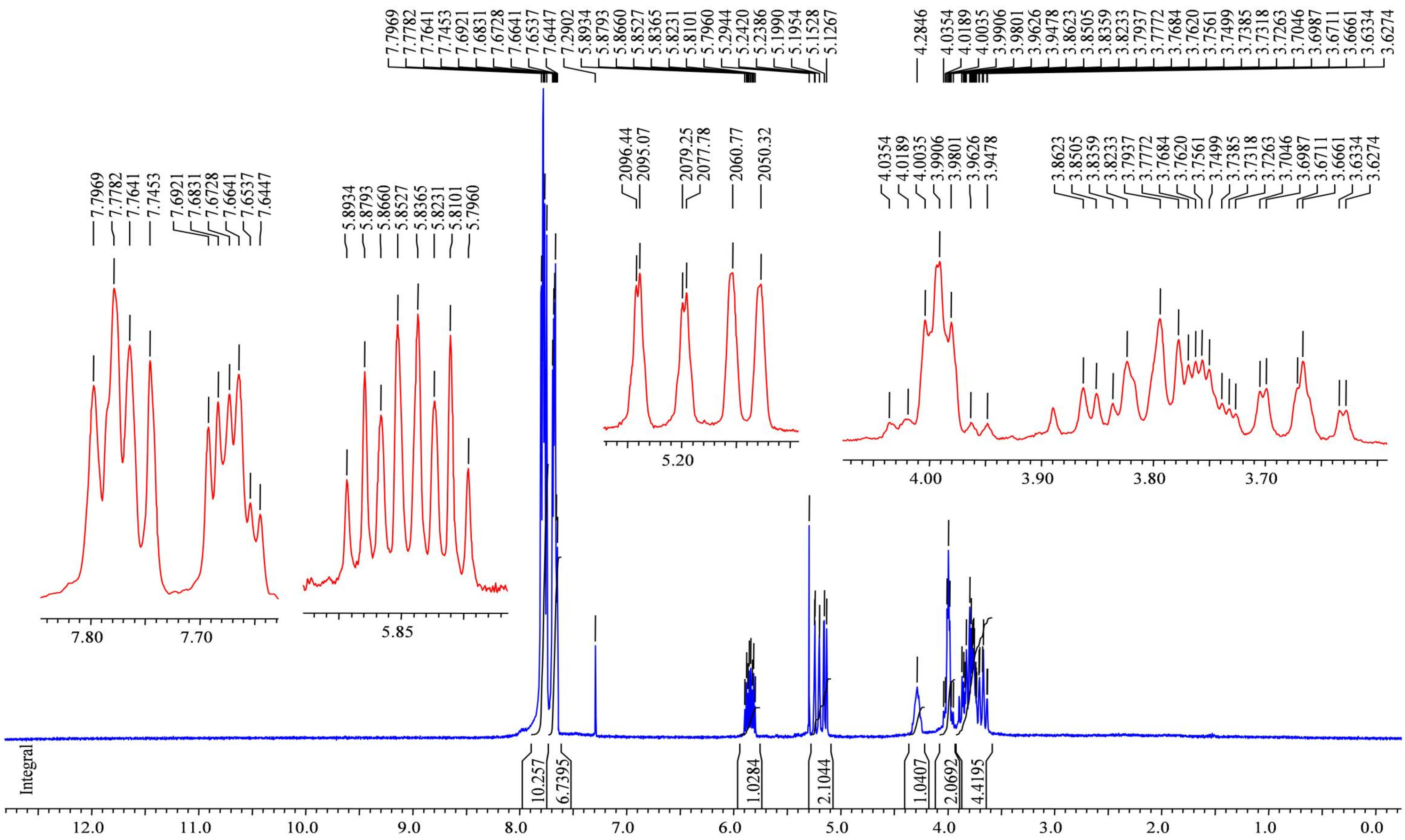

Figure 150. ^{1}H NMR spectrum (400 MHz, $CDCl_3$) of compound (**4c**).

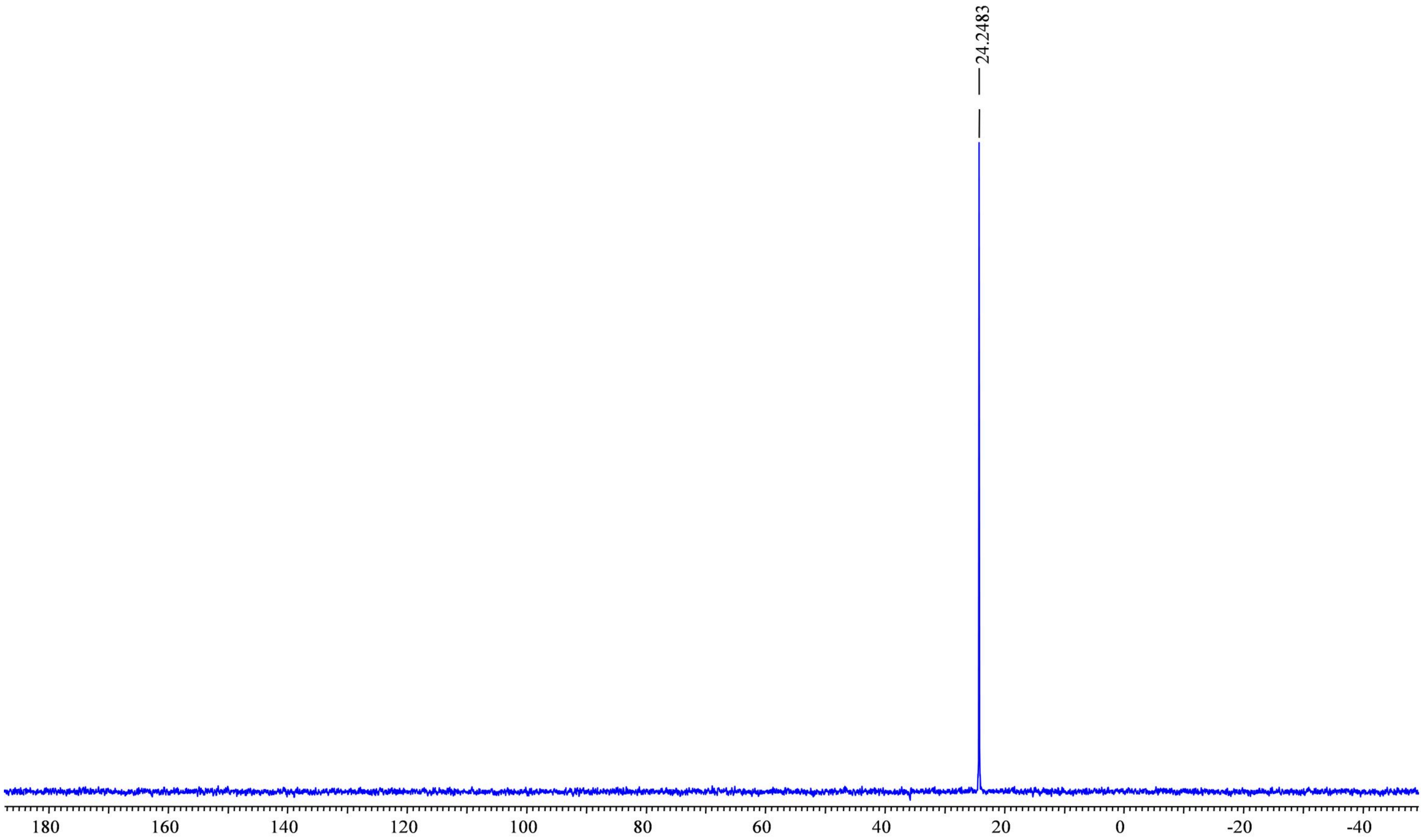

Figure 151. ^{31}P-{^{1}H} NMR spectrum (162.0 MHz, CD_3CN) of compound (**4c**).

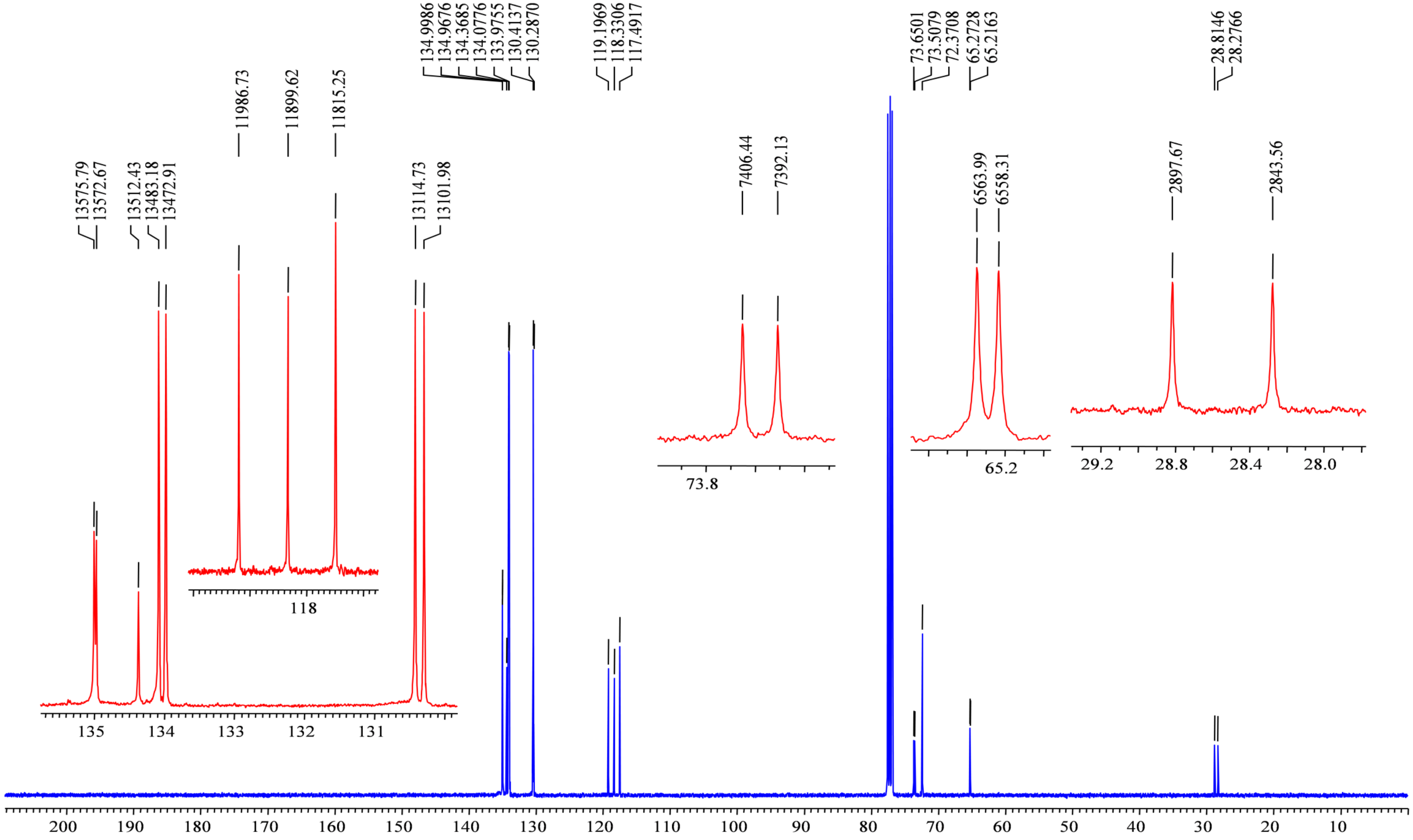

Figure 152. ^{13}C-{^1H} NMR spectrum (100.6 MHz, $CDCl_3$) of compound (**4c**) .

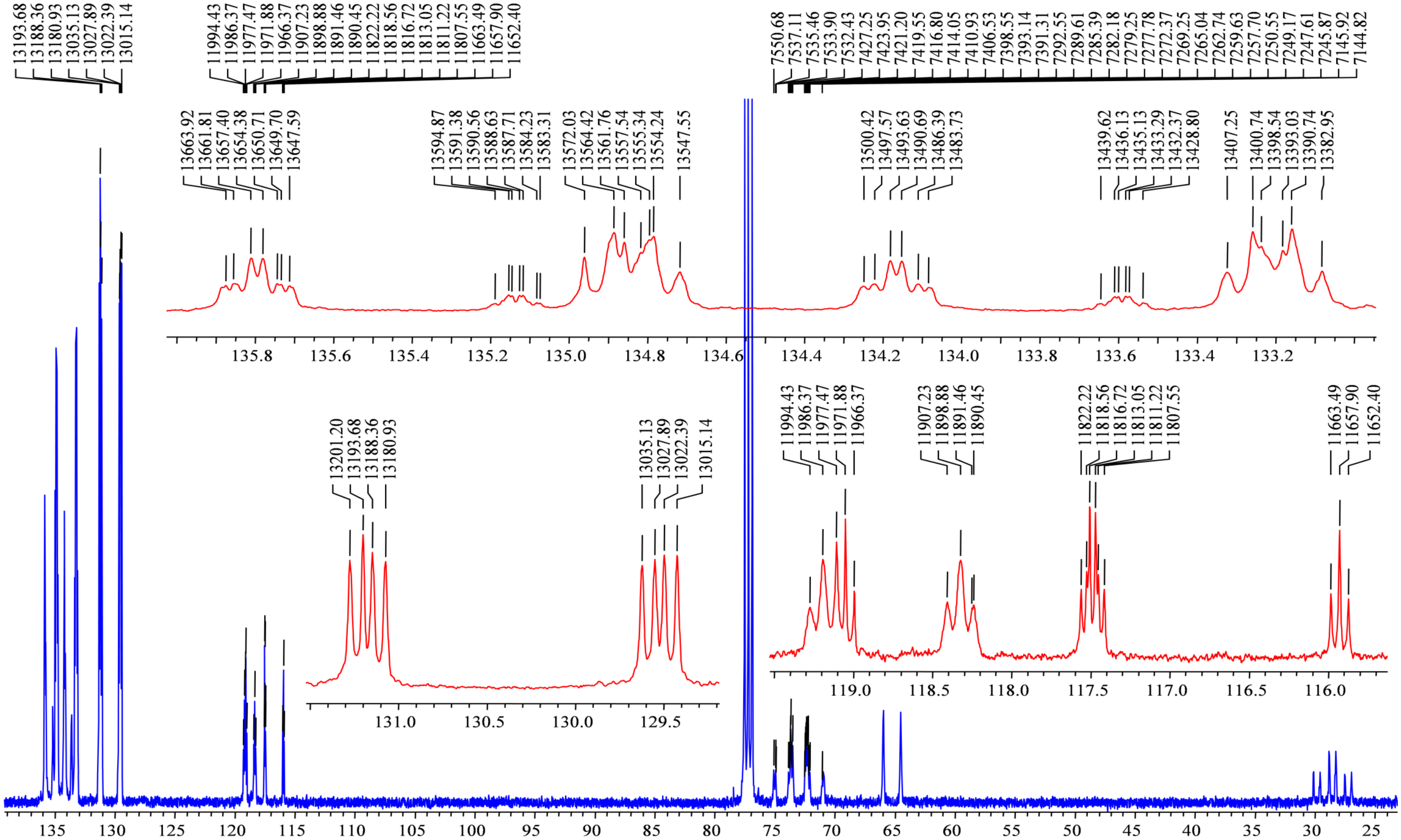

Figure 153. ^{13}C NMR spectrum (100.6 MHz, $CDCl_3$) of compound (**4c**).

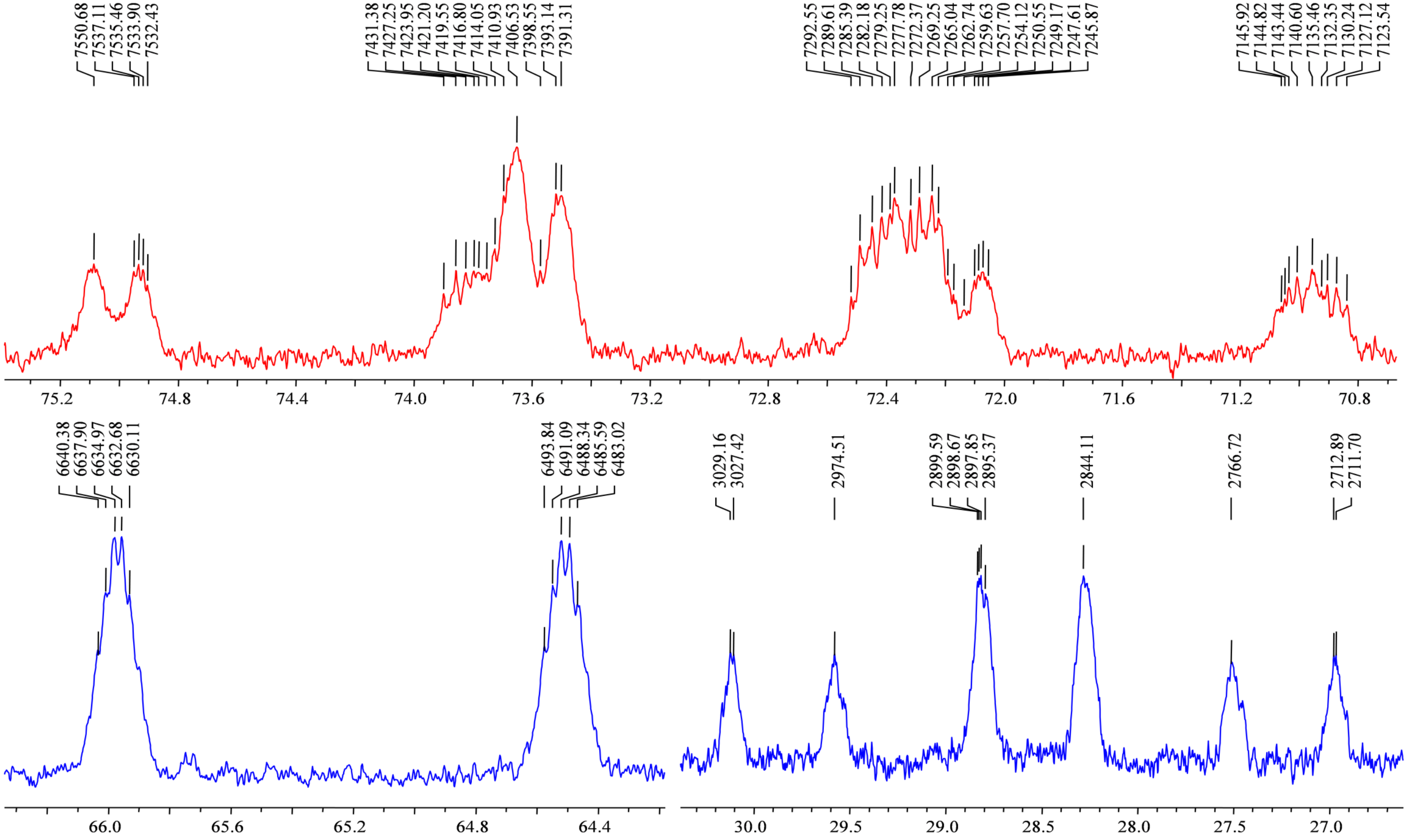

Figure 154. High-field fragments of ^{13}C NMR spectrum (100.6 MHz, $CDCl_3$) of compound (**4c**).

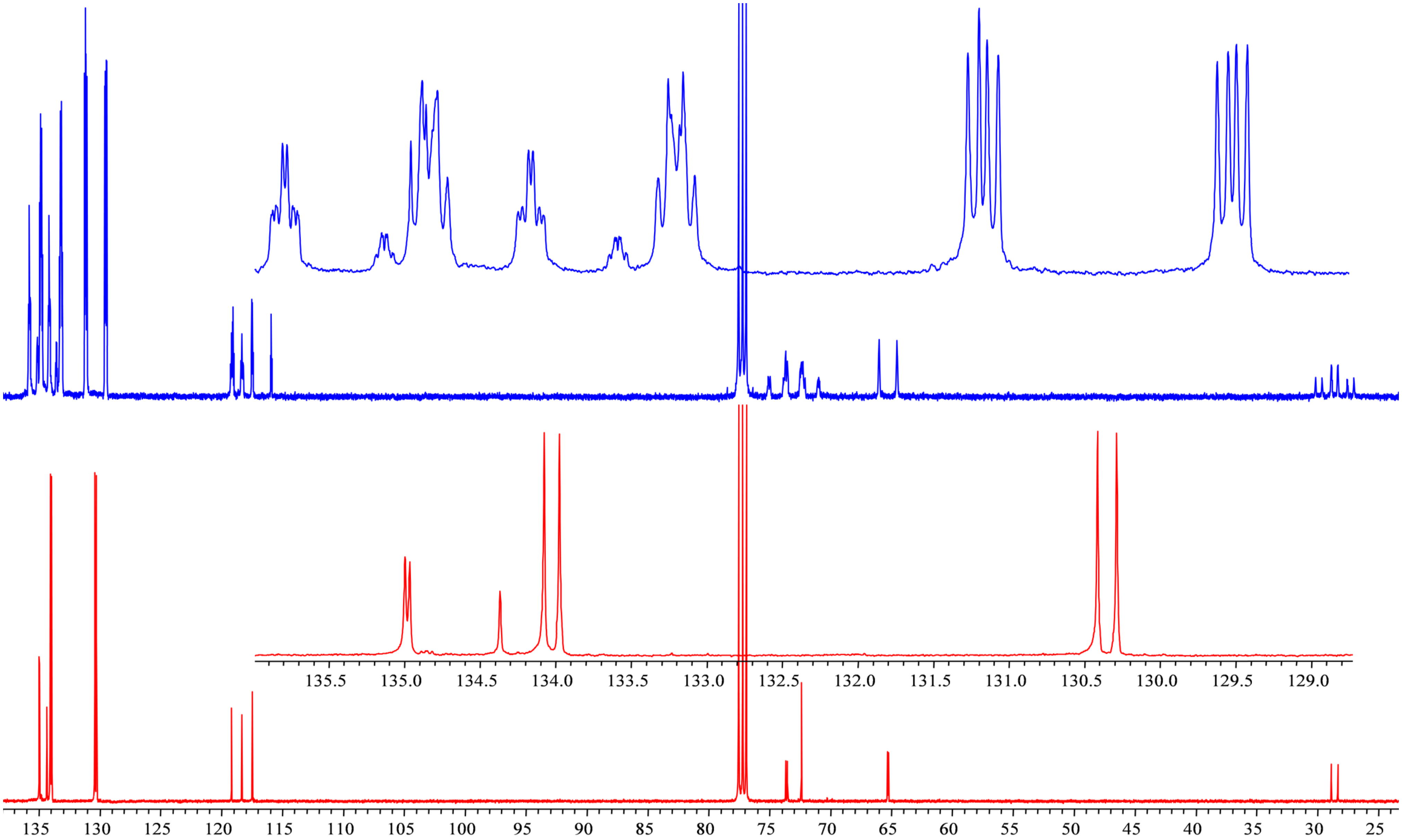

Figure 155. ^{13}C and ^{13}C -{^{1}H} NMR spectra (100.6 MHz, $CDCl_3$) of compound (**4c**).

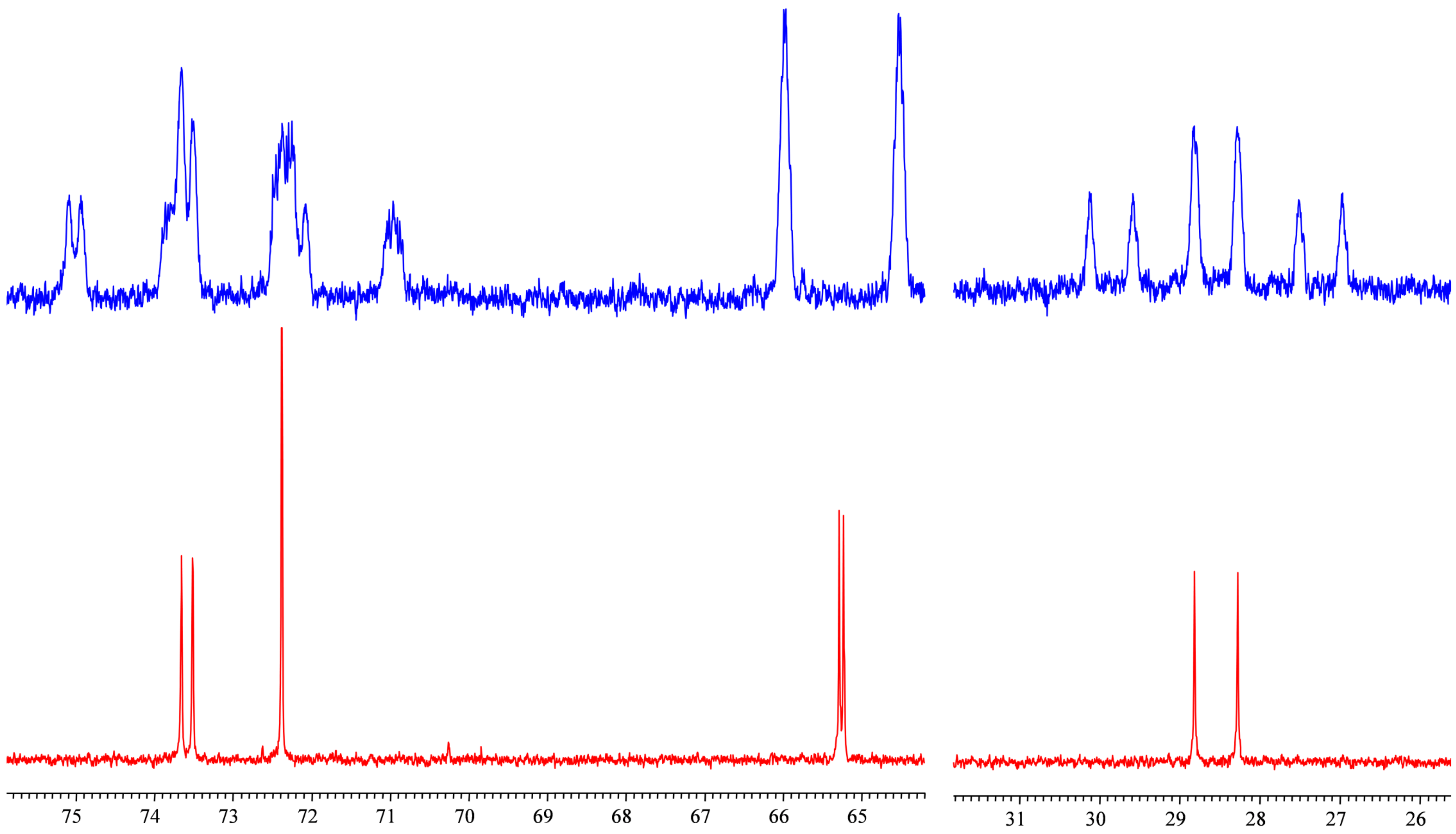

Figure 156. High-field region of ^{13}C and $^{13}C-\{^{1}H\}$ NMR spectra (100.6 MHz, $CDCl_3$) of compound (**4c**).

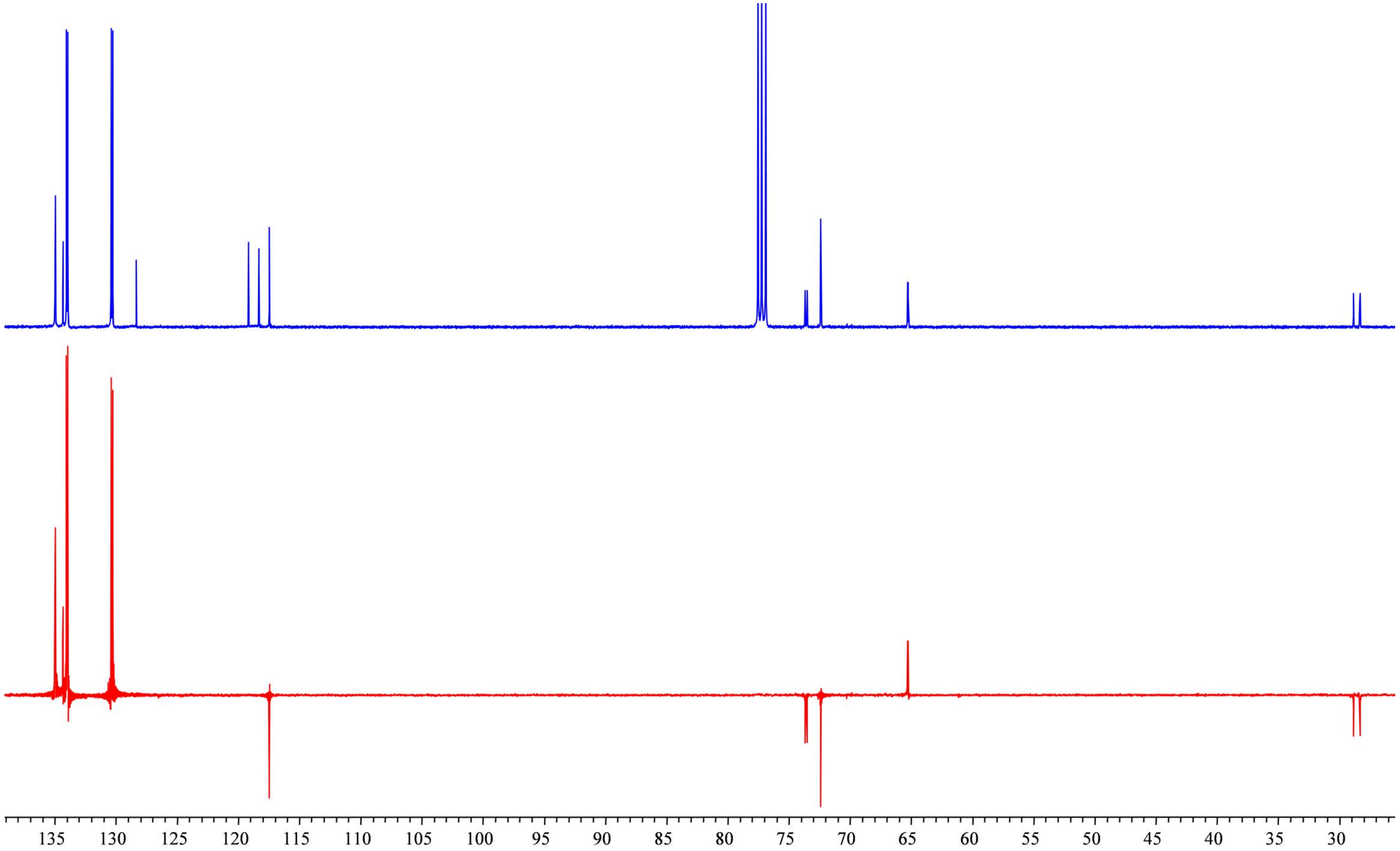

Figure 157. ^{13}C-{^{1}H} and ^{13}C-{^{1}H}-dept NMR spectra (100.6 MHz, $CDCl_3$) of compound (**4c**) .

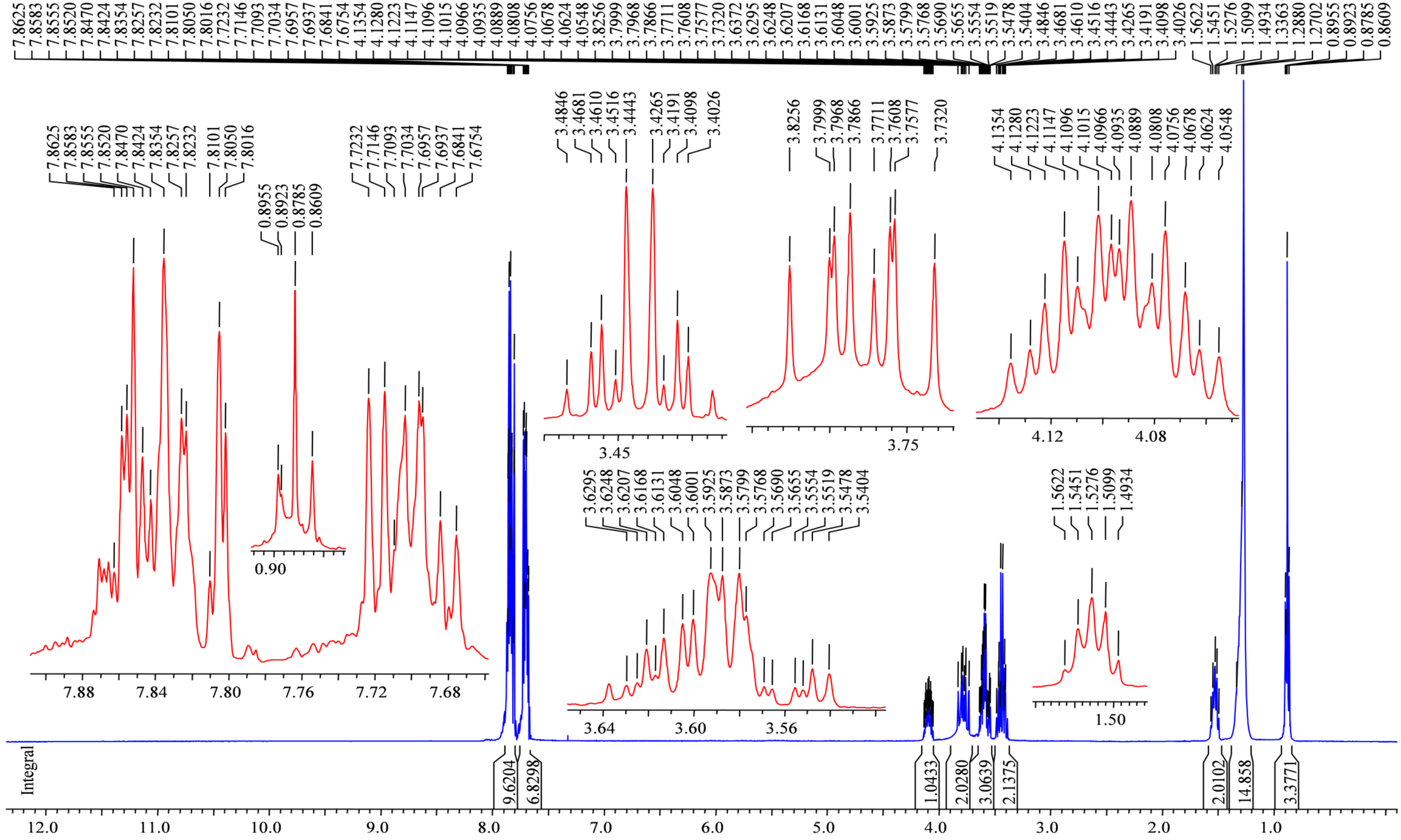

Figure 158. ^{1}H NMR spectrum (400 MHz, $CDCl_3$) of compound (**4d**).

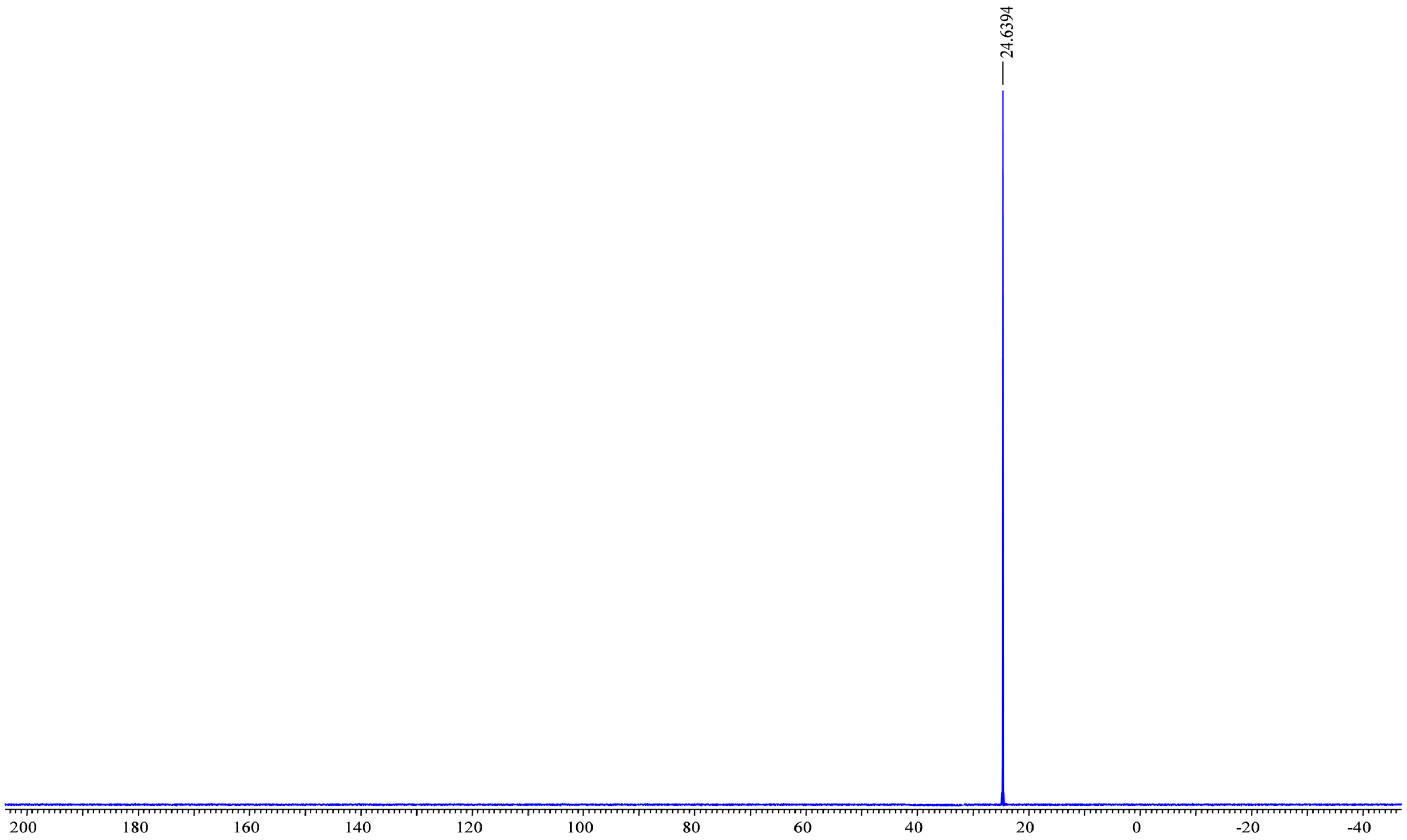

Figure 159. ^{31}P-{^{1}H} NMR spectrum (162.0 MHz, CD_3CN) of compound (**4d**).

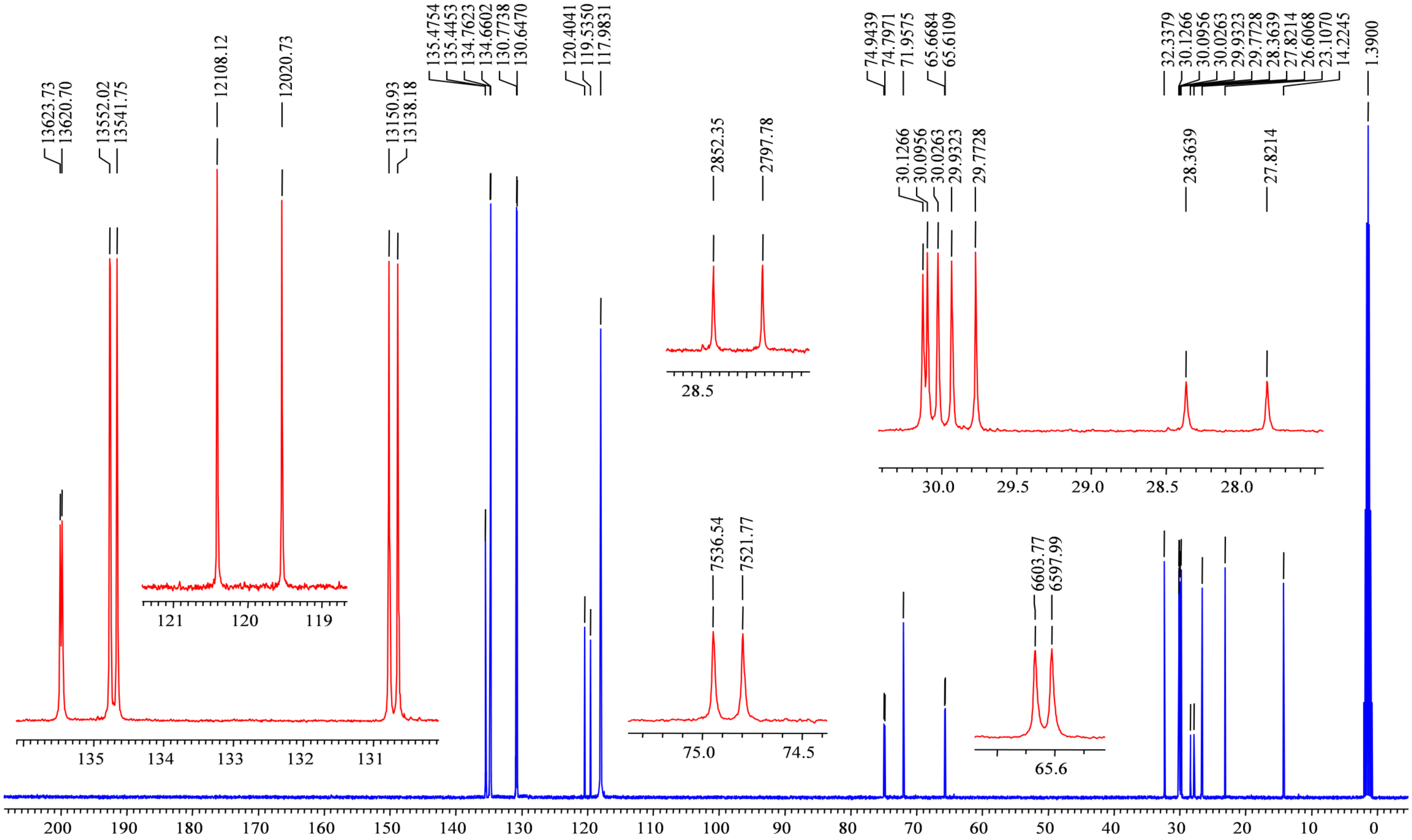

Figure 160. ^{13}C-{^{1}H} NMR spectrum (100.6 MHz, CD_3CN) of compound (**4d**).

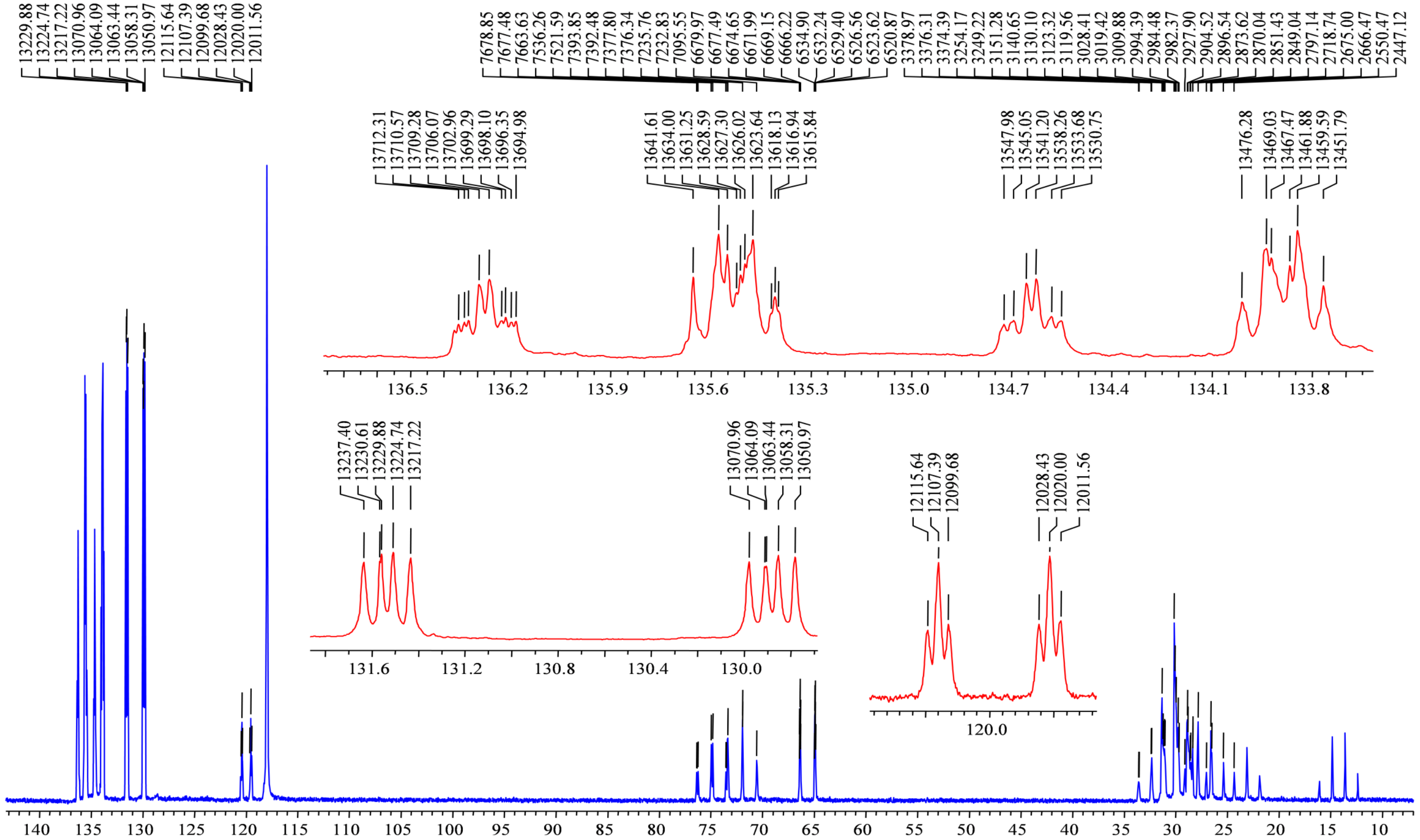

Figure 161. ^{13}C NMR spectrum (100.6 MHz, CD_3CN) of compound (**4d**) .

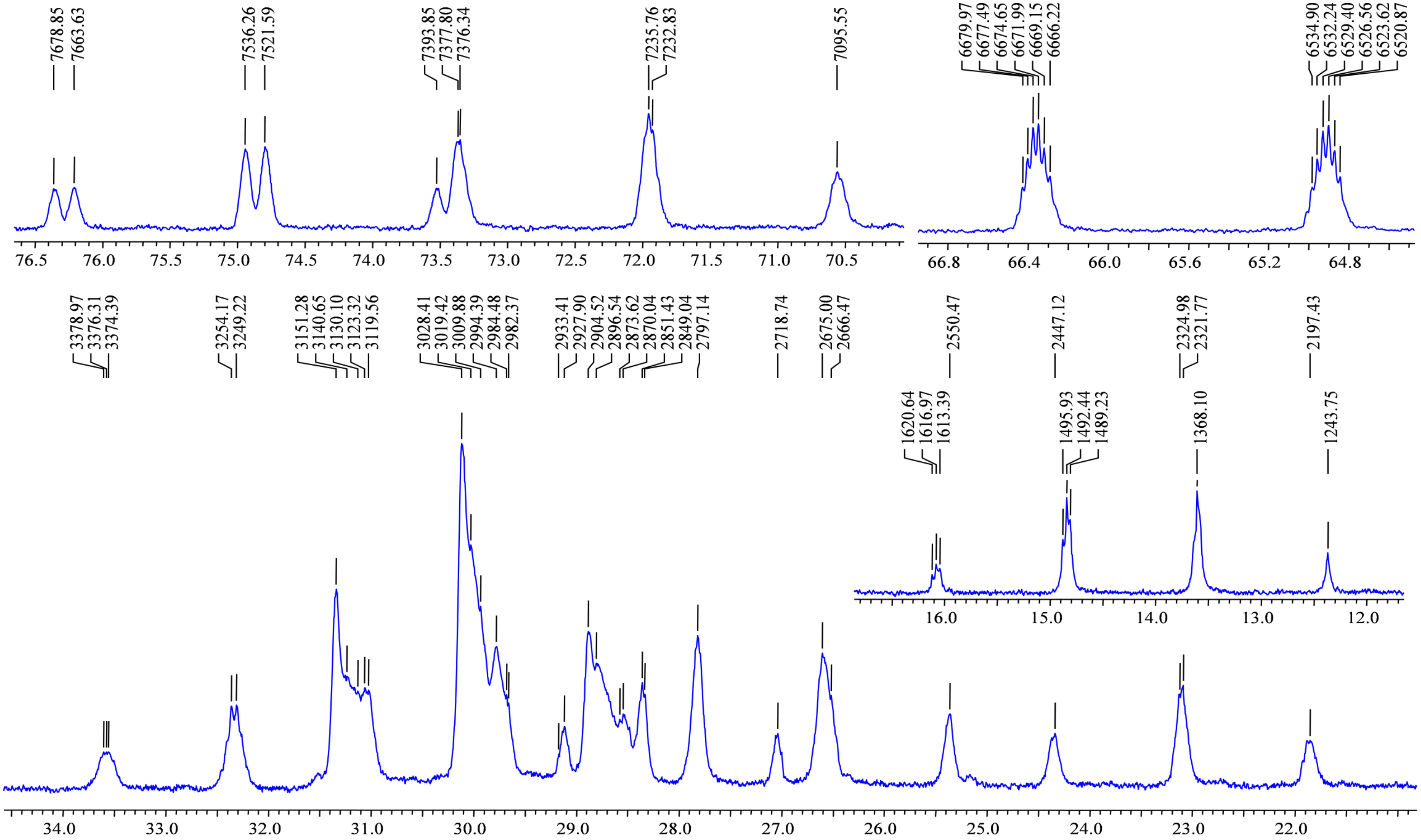

Figure 162. High-field fragments of ^{13}C NMR spectrum (100.6 MHz, CD_3CN) of compound (**4d**).

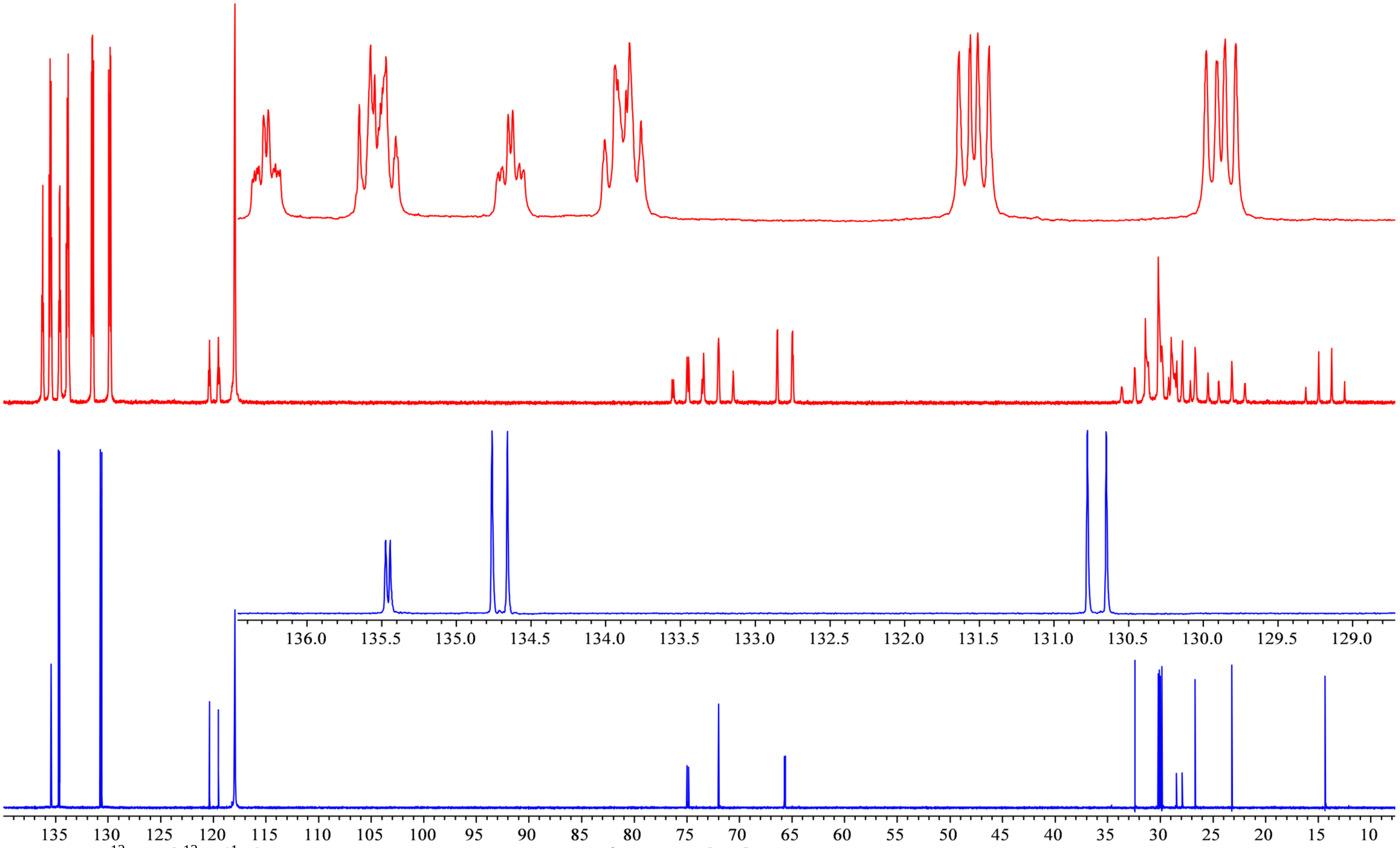

Figure 163. ^{13}C and ^{13}C-{^{1}H} NMR spectra (100.6 MHz, CD_3CN) of compound (**4d**).

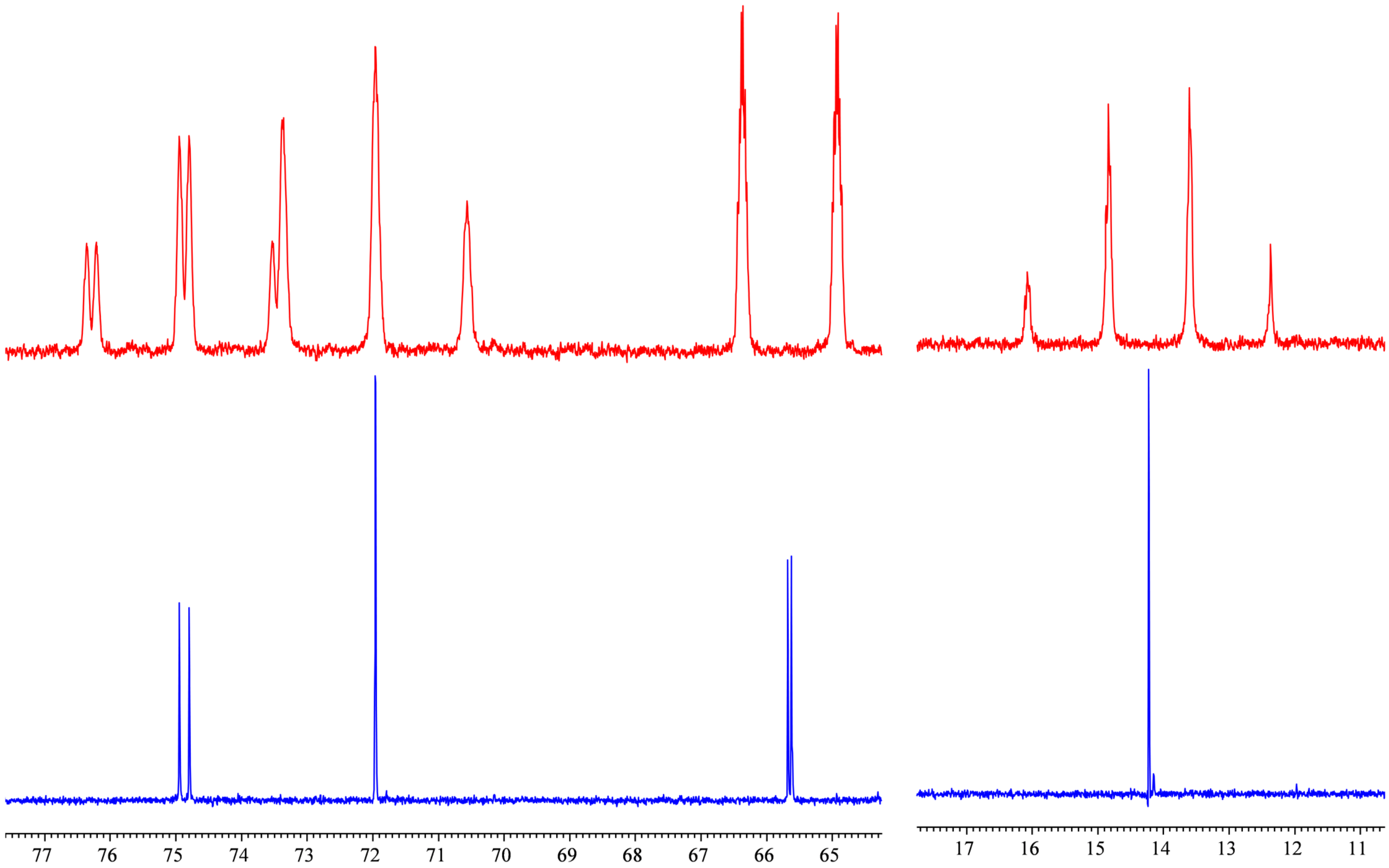

Figure 164. ^{13}C and ^{13}C-{^{1}H} NMR spectra (100.6 MHz, CD_3CN) of compound (**4d**).

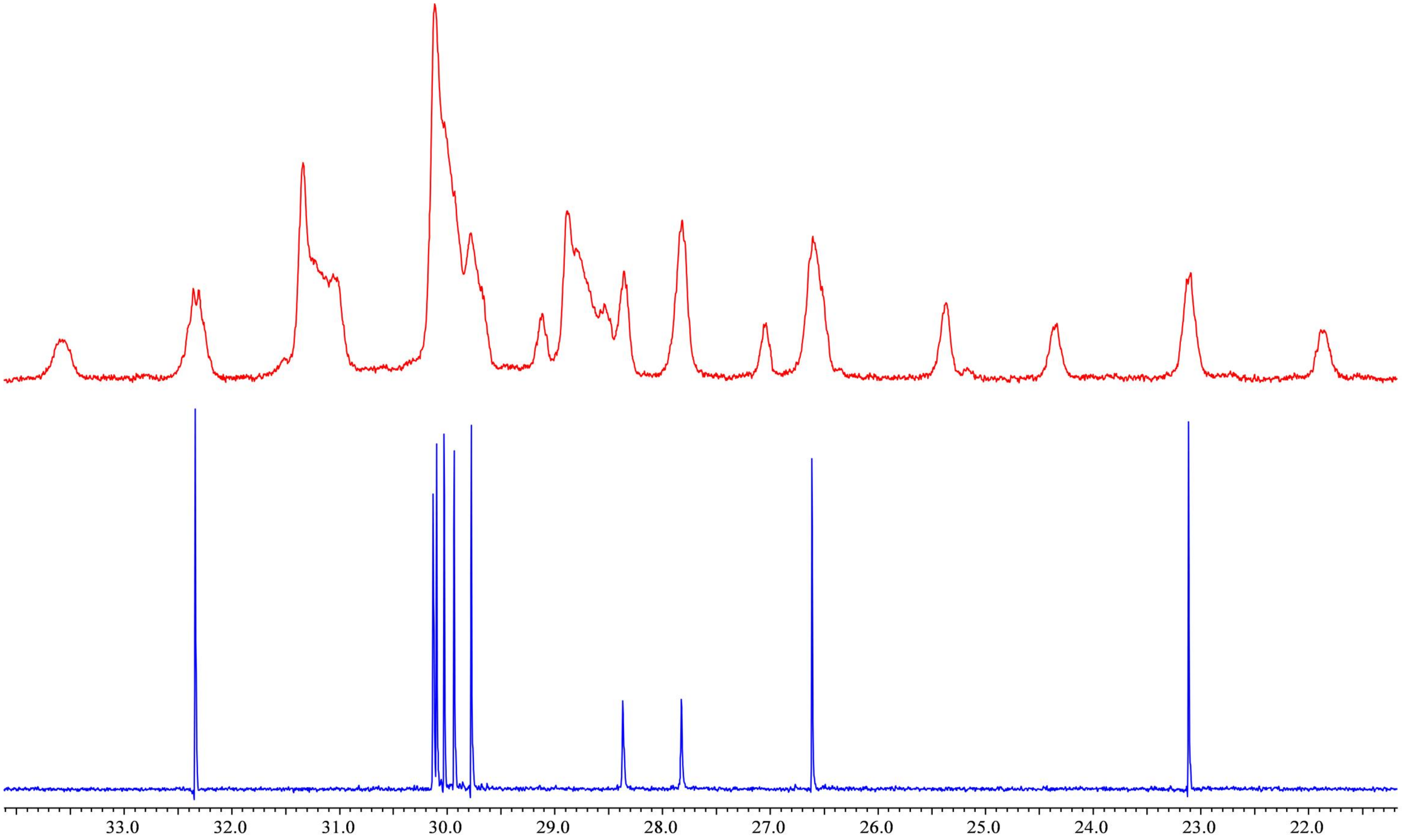

Figure 165. High-field region of $^{13}C-\{^{1}H\}$ and ^{13}C NMR spectra (100.6 MHz, CD_3CN) of compound (**4d**).

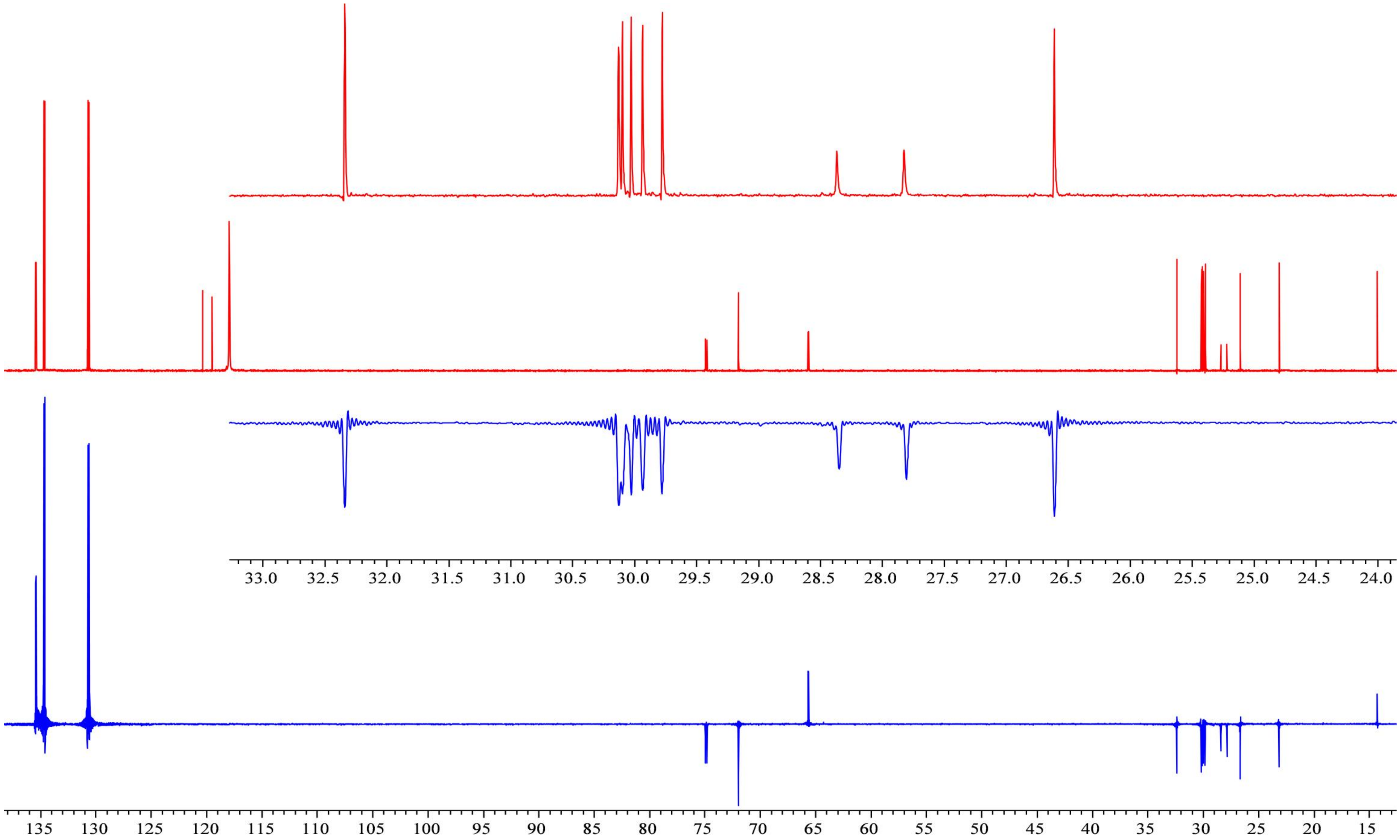

Figure 166. ^{13}C-{^{1}H} and ^{13}C-{^{1}H}-dept NMR spectra (100.6 MHz, CD_3CN) of compound (**4d**).

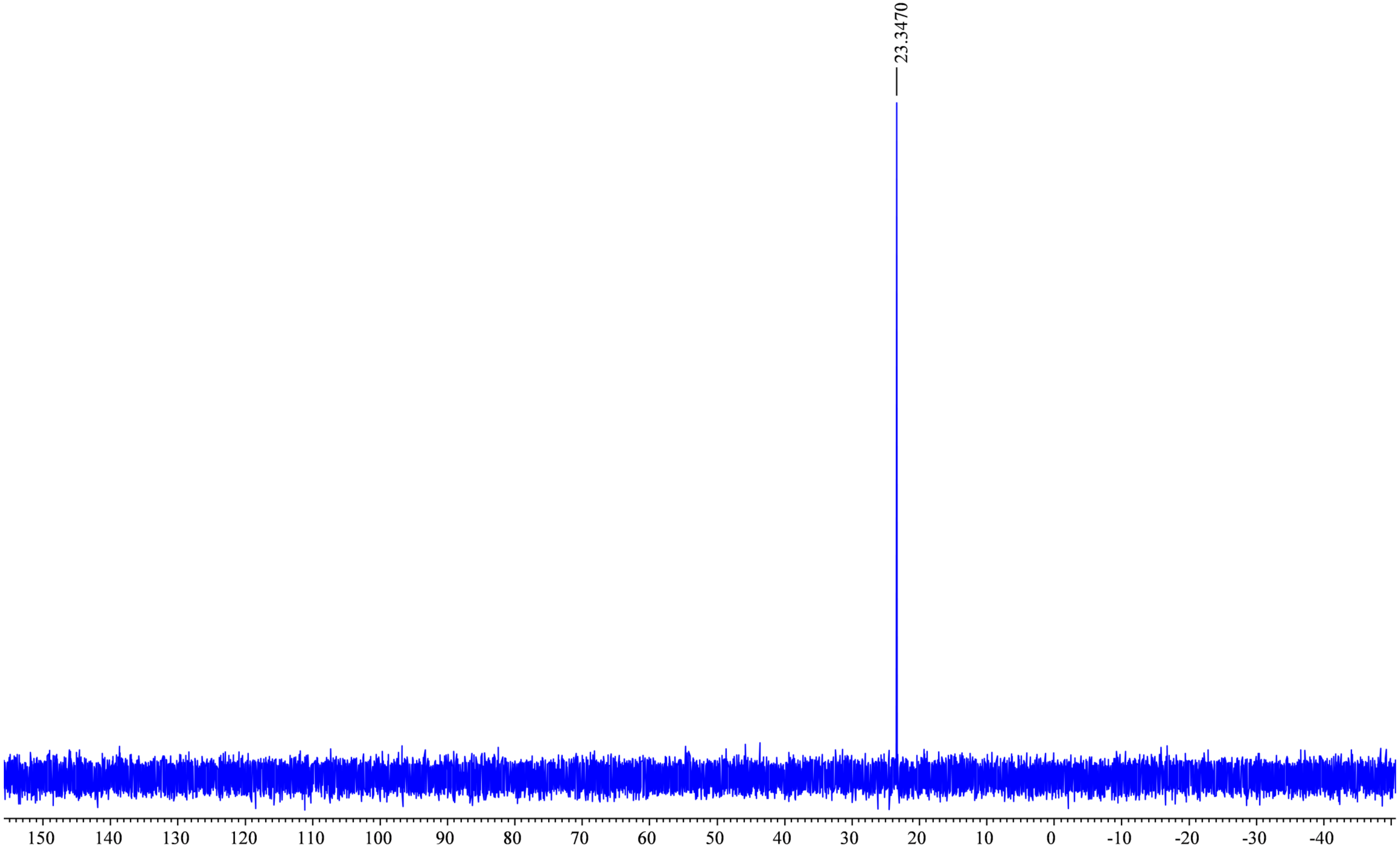

Figure 167. ^{31}P-{^{1}H} NMR spectrum (242.94 MHz, $CDCl_3$ + 10% MeCN) of compound (**7**).

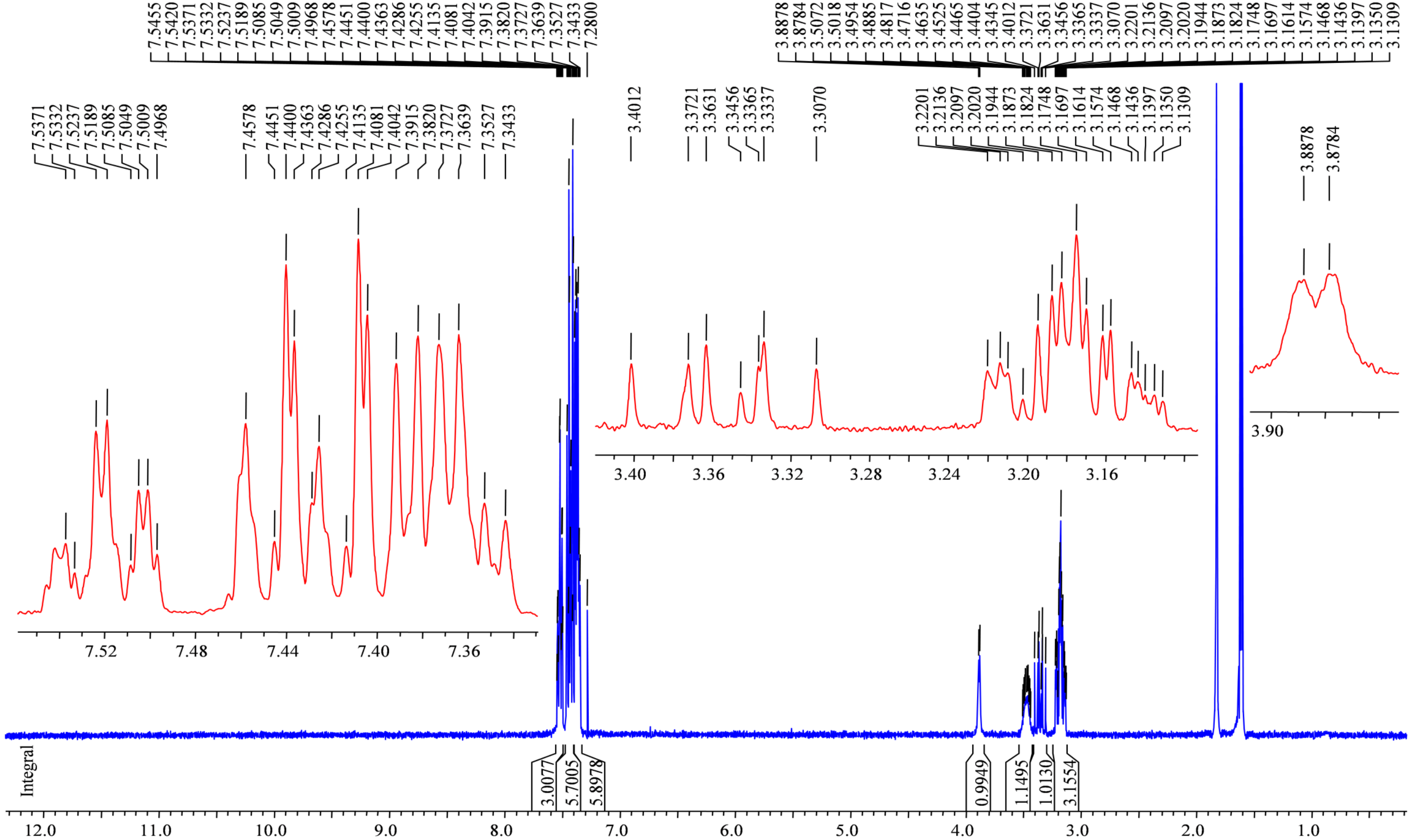

Figure 168. ^{1}H NMR spectrum (242.94 MHz, CD_3CN) of compound (**7**).

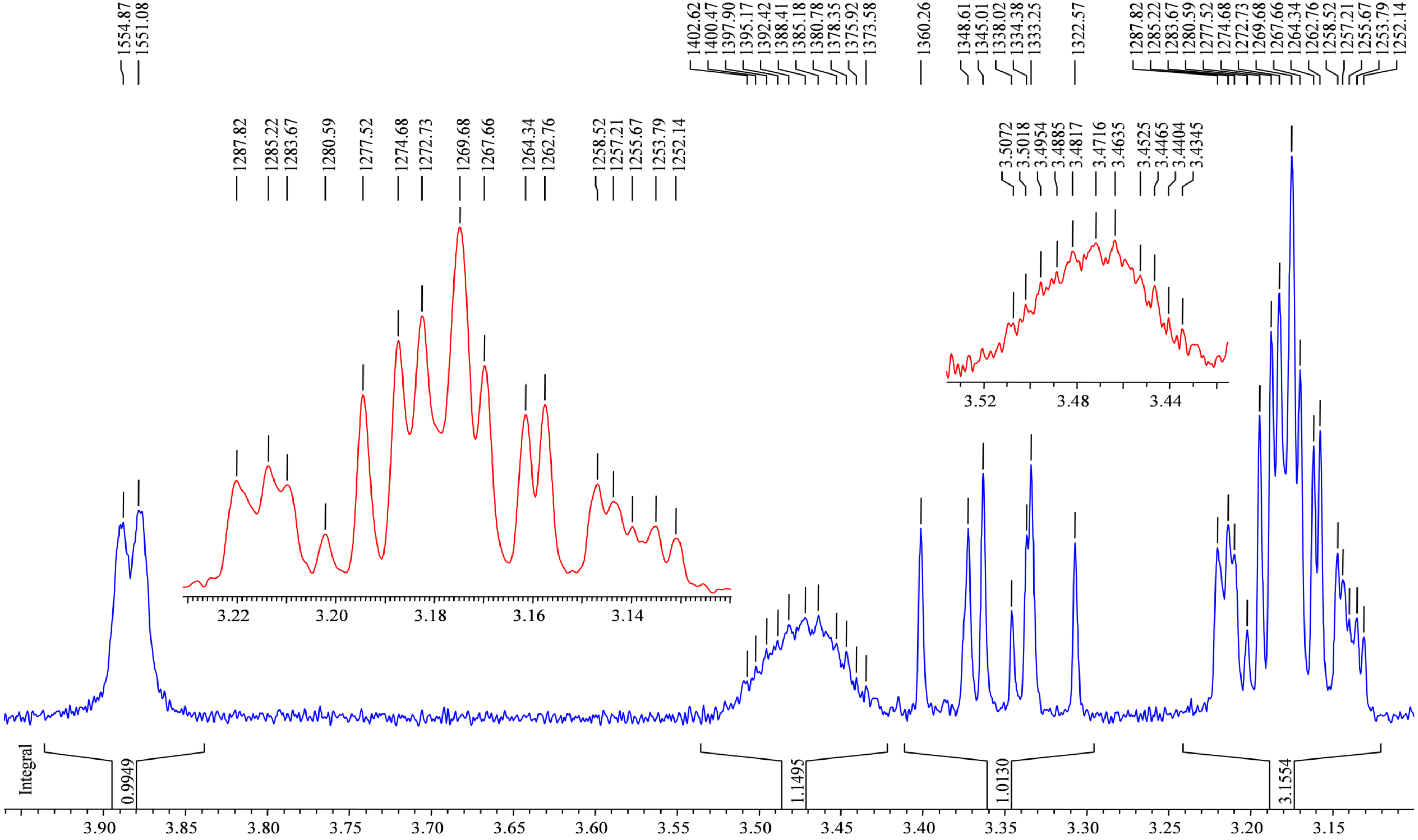

Figure 169. High-field fragment of ^{1}H NMR spectrum (242.94 MHz, CD_3CN) of compound (**7**).

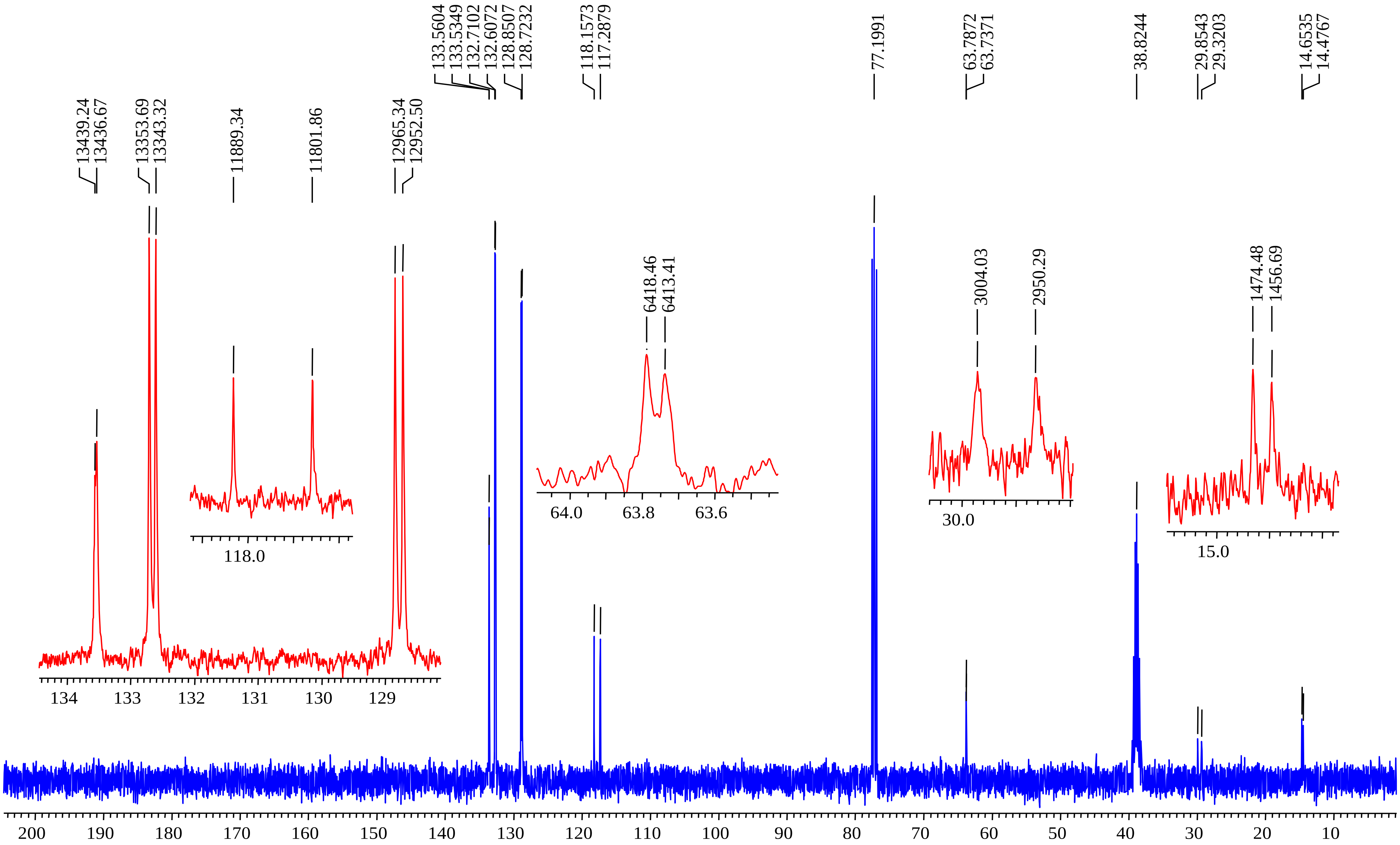

Figure 170. ^{13}C-{^{1}H} NMR spectrum (100.6 MHz, $CDCl_3$ + 20% DMSO-d_6) of compound (**7**).

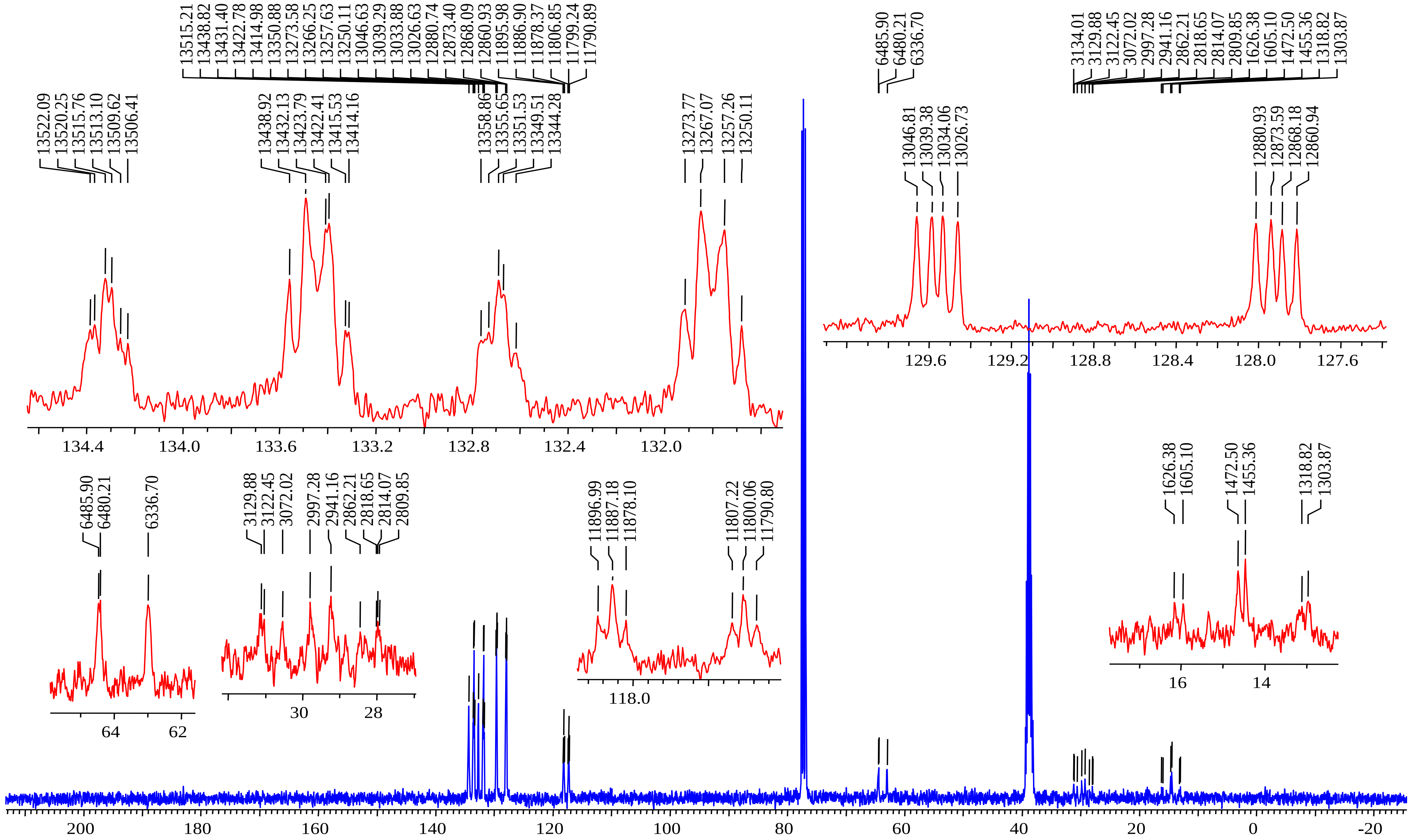

Figure 171. ^{13}C NMR spectrum (100.6 MHz, $CDCl_3$ + 20% DMSO-d_6) of compound (**7**).

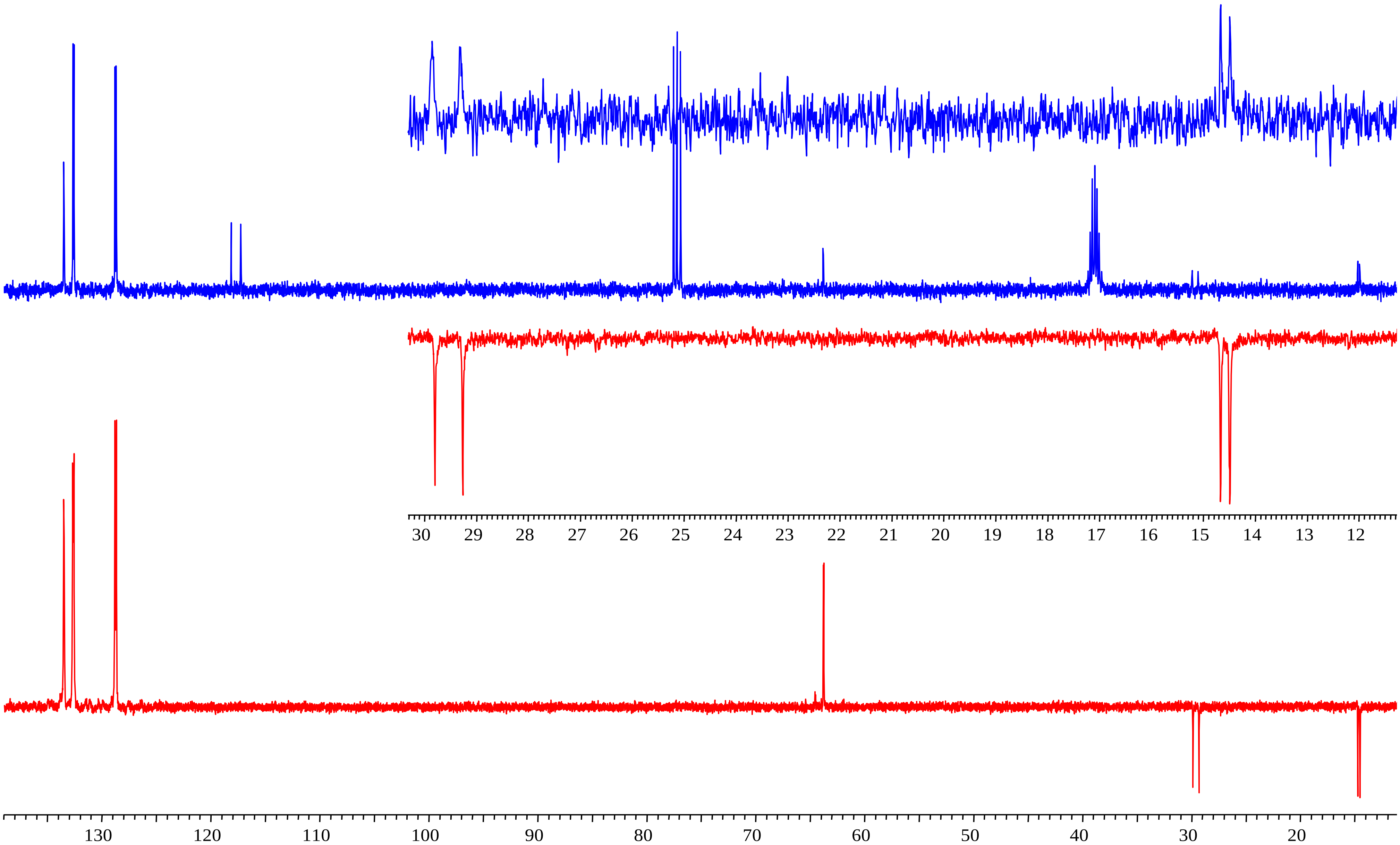

Figure 172. ^{13}C-{^{1}H} and ^{13}C-{^{1}H}-dept NMR spectra (100.6 MHz, $CDCl_3$ + 20% DMSO-d_6) of compound (**7**).

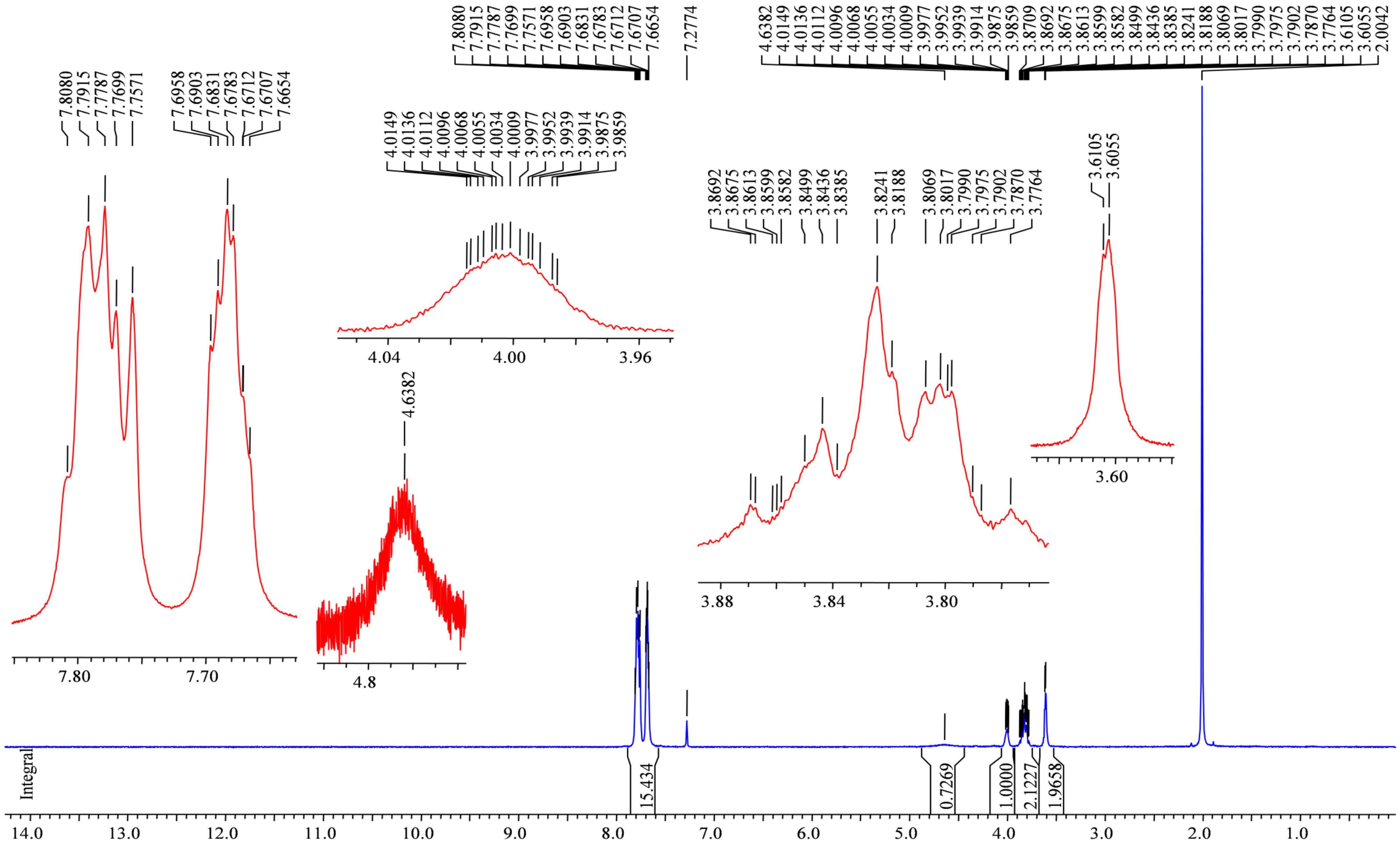

Figure 173. ^{1}H NMR spectrum (600 MHz, $CDCl_3$ + 10% MeCN) of compound (**7**).

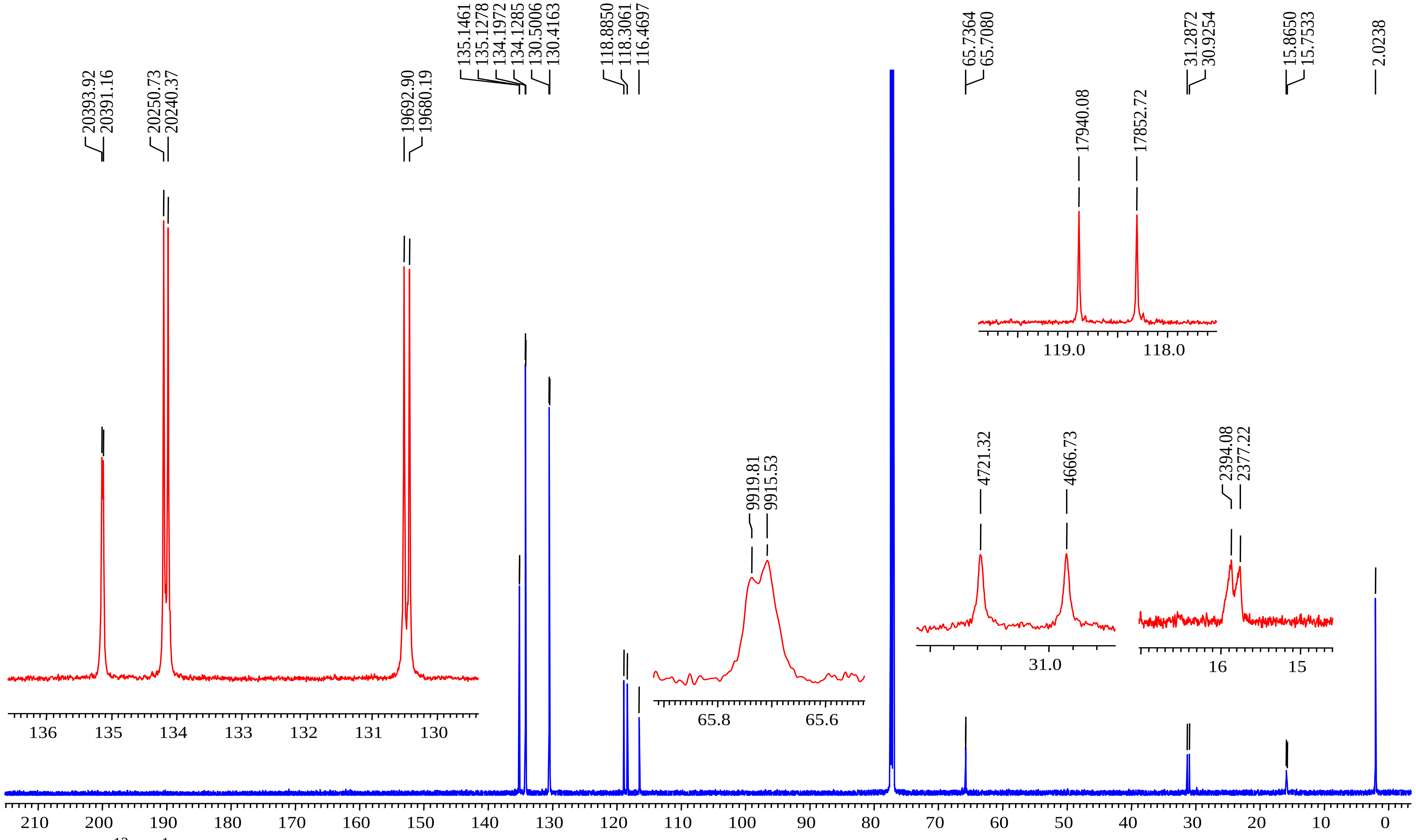

Figure 174. ^{13}C-{^{1}H} NMR spectrum (150.9 MHz, $CDCl_3$ + 10% MeCN) of compound (**7**).

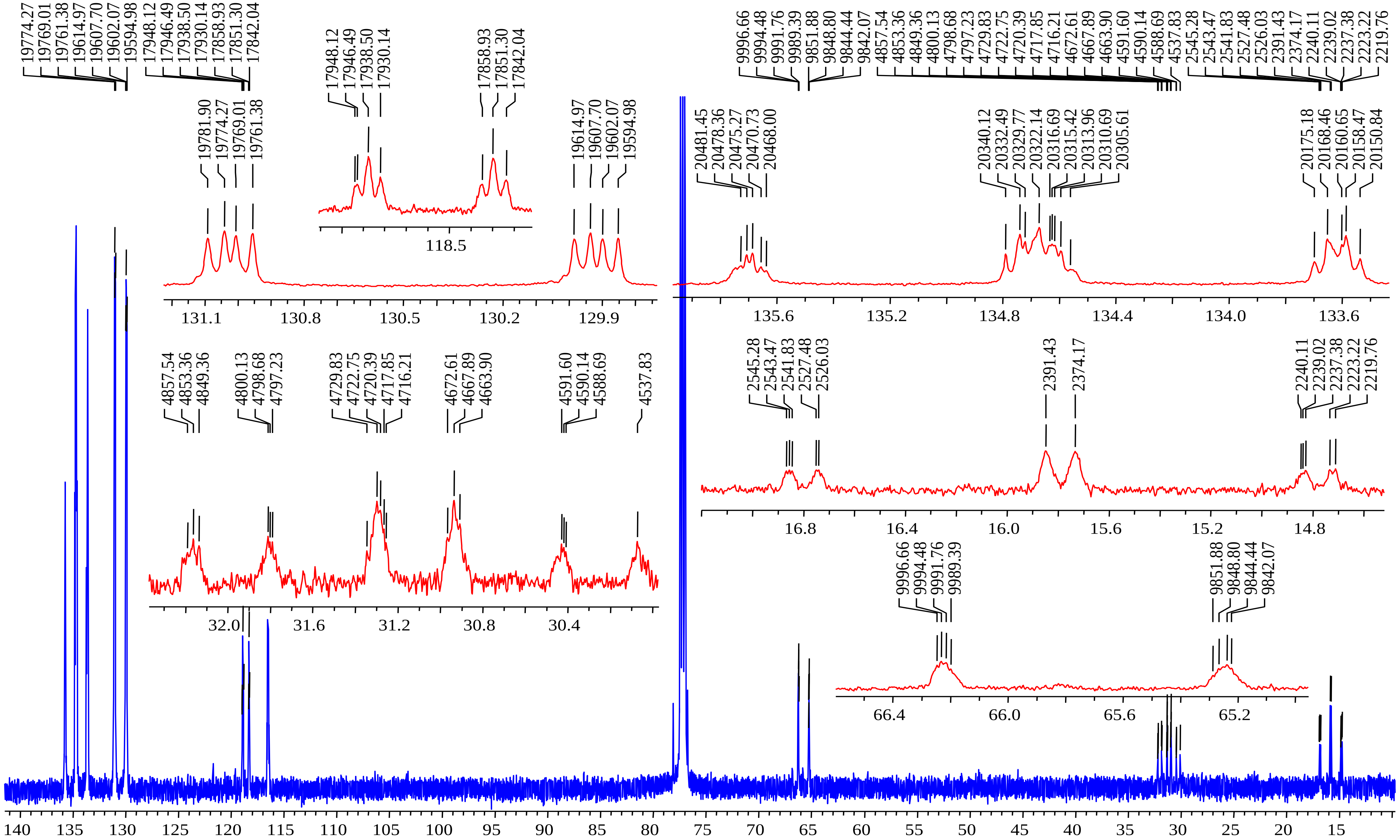

Figure 175. ^{13}C NMR spectrum (150.9 MHz, $CDCl_3$ + 10% MeCN) of compound (**7**).

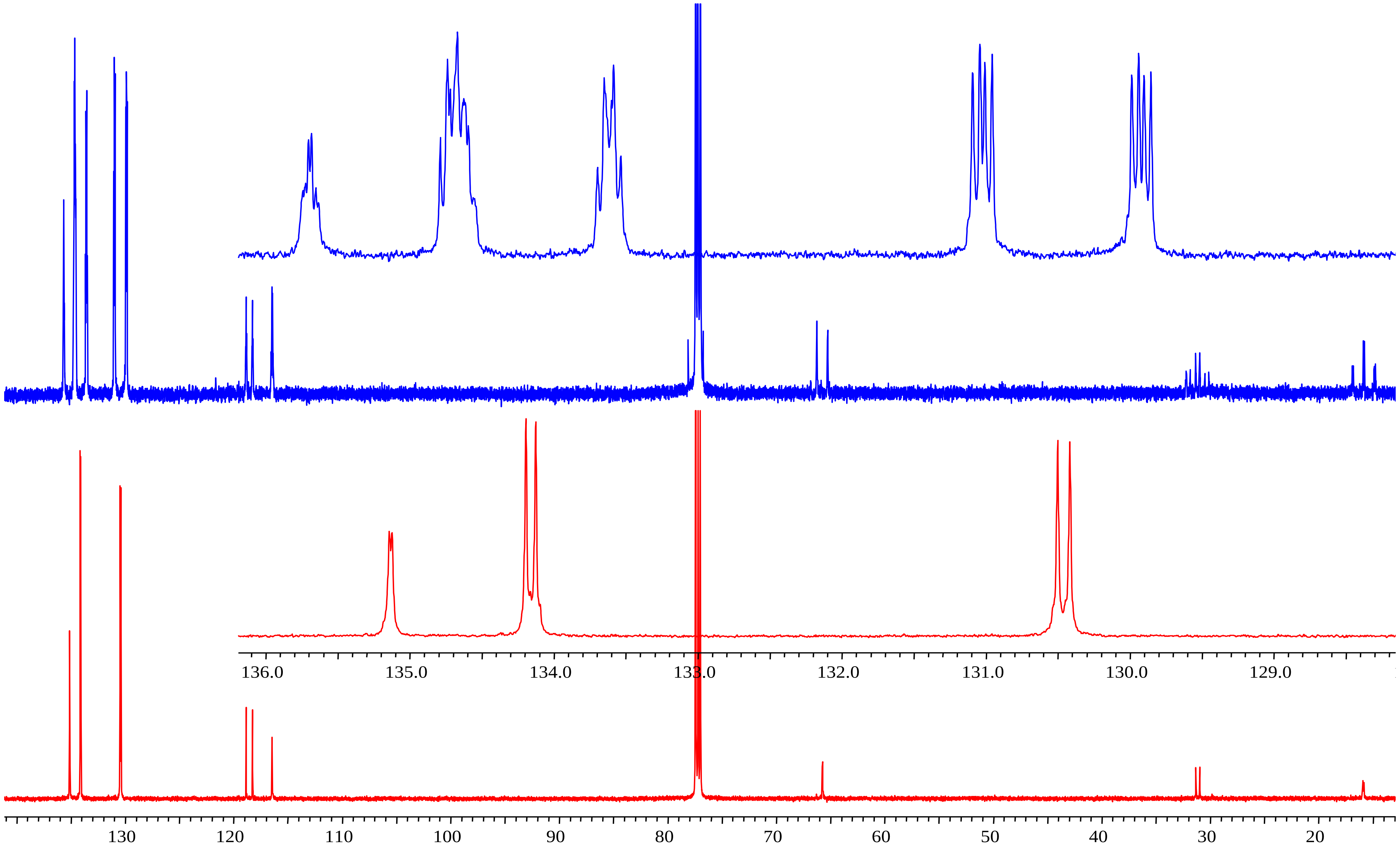

Figure 176. ^{13}C and ^{13}C-{^{1}H} NMR spectra (150.9 MHz, $CDCl_3$ + 10% MeCN) of compound (**7**).

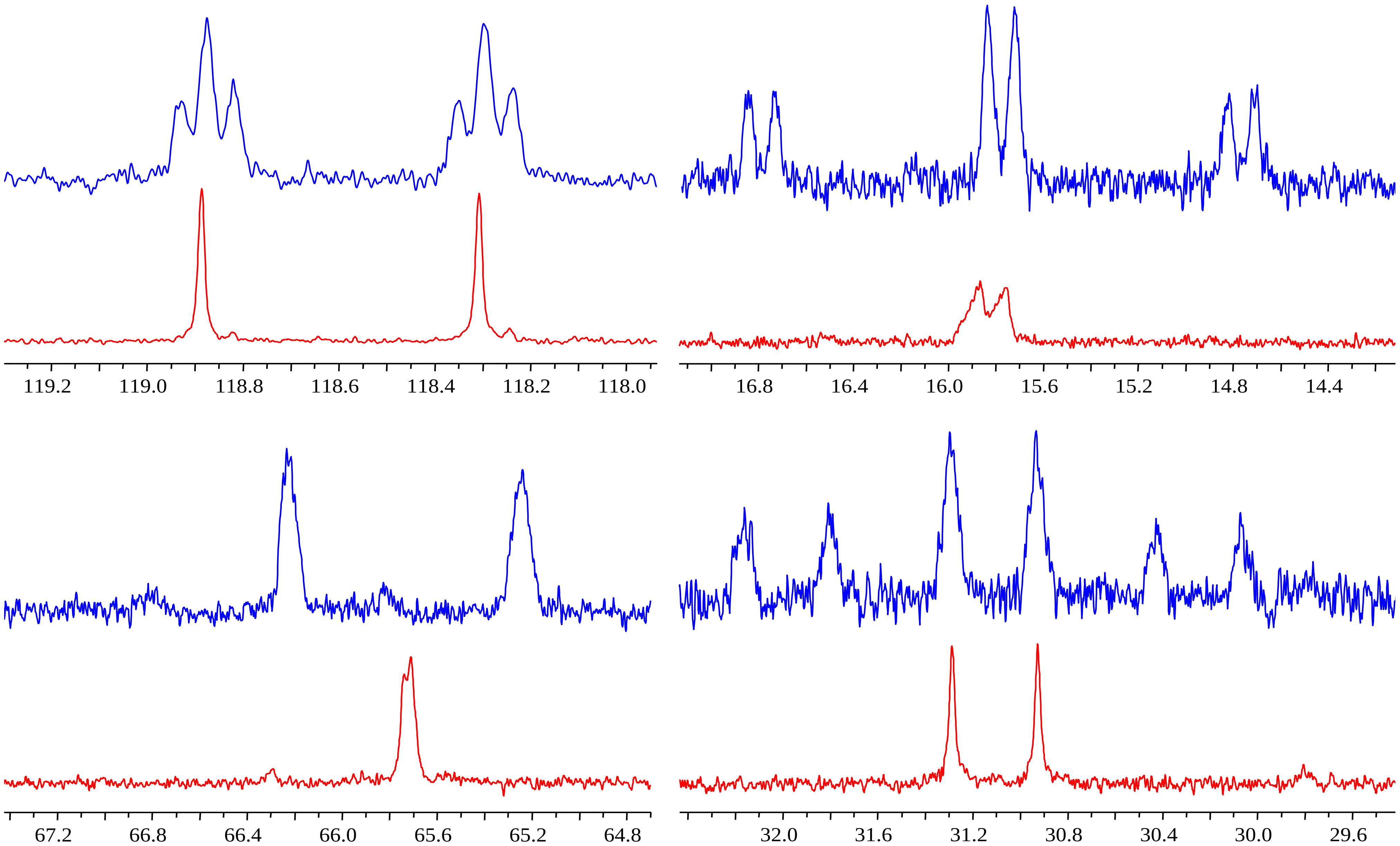

Figure 177. Fragments of ^{13}C and ^{13}C-{^{1}H} NMR spectra (150.9 MHz, $CDCl_3$ + 10% MeCN) of compound (**7**).

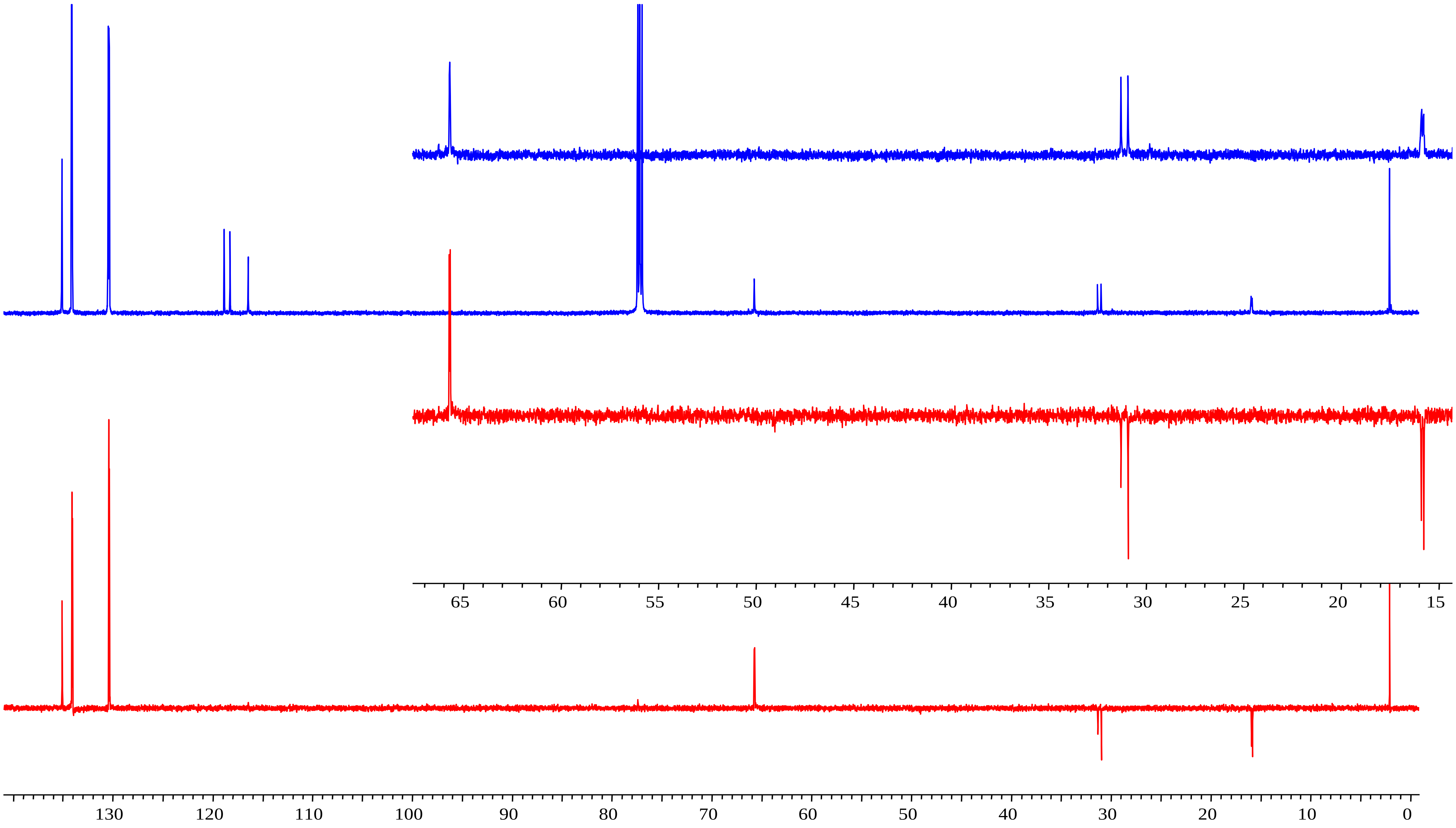

Figure 178. ^{13}C-{^{1}H} and ^{13}C-{^{1}H}-dept NMR spectra (150.9 MHz, $CDCl_3$ + 10% MeCN) of compound (**7**).
